# Supplementary material for: Looking at the Pathogenesis of the Rabies Lyssavirus Strain Pasteur Vaccins through a Prism of the Disorder-Based Bioinformatics
Source: Biomolecules. 2022 Oct 7;12(10):1436. doi: 10.3390/biom12101436 (PMC9599798; doi:10.3390/biom12101436)
Supplement: Supplementary file 1 [file biomolecules-12-01436-s001.zip › biomolecules-1917533-supplementary.pdf]

Supplementary Materials

Looking at the Pathogenesis of the Rabies Lyssavirus Strain Pasteur Vaccins through a Prism of the Disorder-Based Bioinformatics

Surya Dhulipala 1 and Vladimir Uversky 1,2,3,*

[1] Department of Molecular Medicine, Morsani College of Medicine, University of South Florida, Tampa, FL 33612, USA

[2] USF Health Byrd Alzheimer's Research Institute, Morsani College of Medicine, University of South Florida, Tampa, FL 33612, USA

[3] Protein Research Group, Institute for Biological Instrumentation of the Russian Academy of Sciences, Federal Research Center "Pushchino Scientific Center for Biological Research of the Russian Academy of Sciences", 142290 Pushchino, Moscow Region, Russia

[*] Correspondence: vuversky@usf.edu

Supplementary Figure S1. Functional disorder in human proteins interacting with the RABV P-protein. For each protein, an amino acid sequence in FASTA format is shown followed by the disorder profile generated by RIDAO, D^{2}P^2-generated functional disorder profile, modeled 3D structure generated by AlphaFold, and STRING-based protein-protein interaction network.

DYNLL1 (UniProt ID: P63167)

>sp|P63167|DYL1_HUMAN Dynein light chain 1, cytoplasmic OS=Homo sapiens OX=9606 GN=DYNLL1 PE=1 SV=1

MCDRKAVIKNADMSEEMQQDSVECATQALEKYNIEKDIAAHIKKEFDKKYNPTWHCIV GRNFGSYVTHETKHFIYFYLGQVAILLFKSG

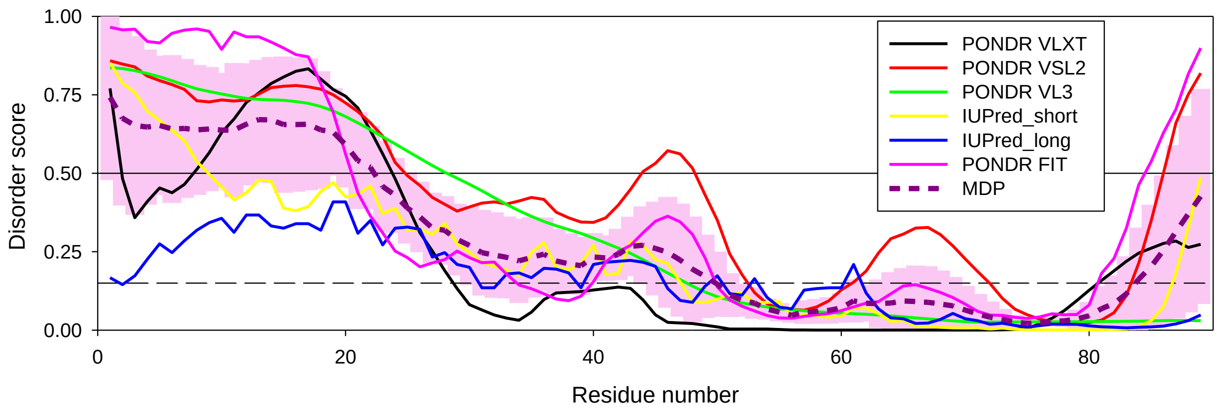

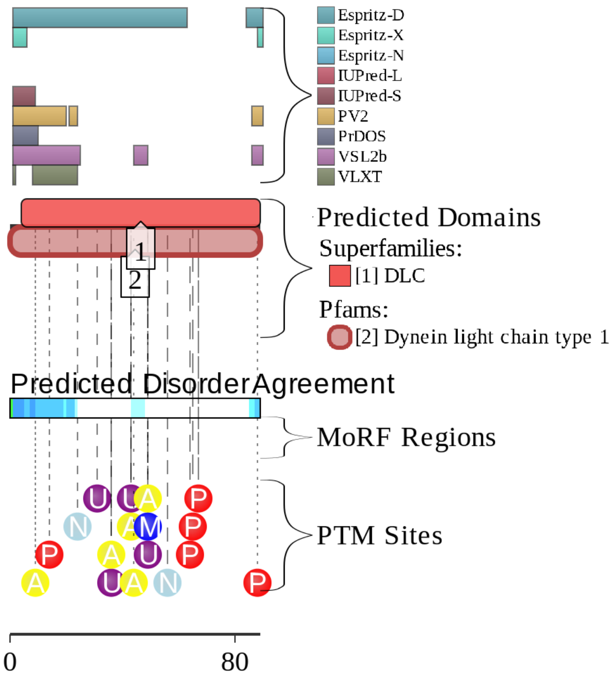

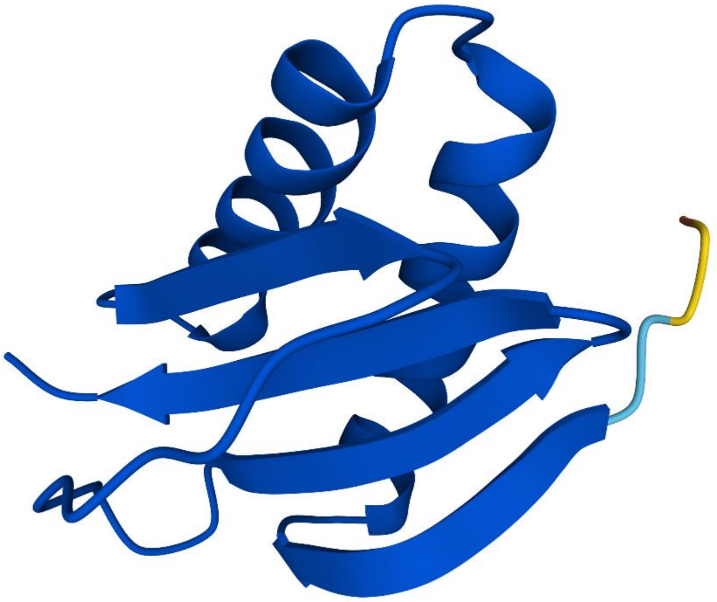

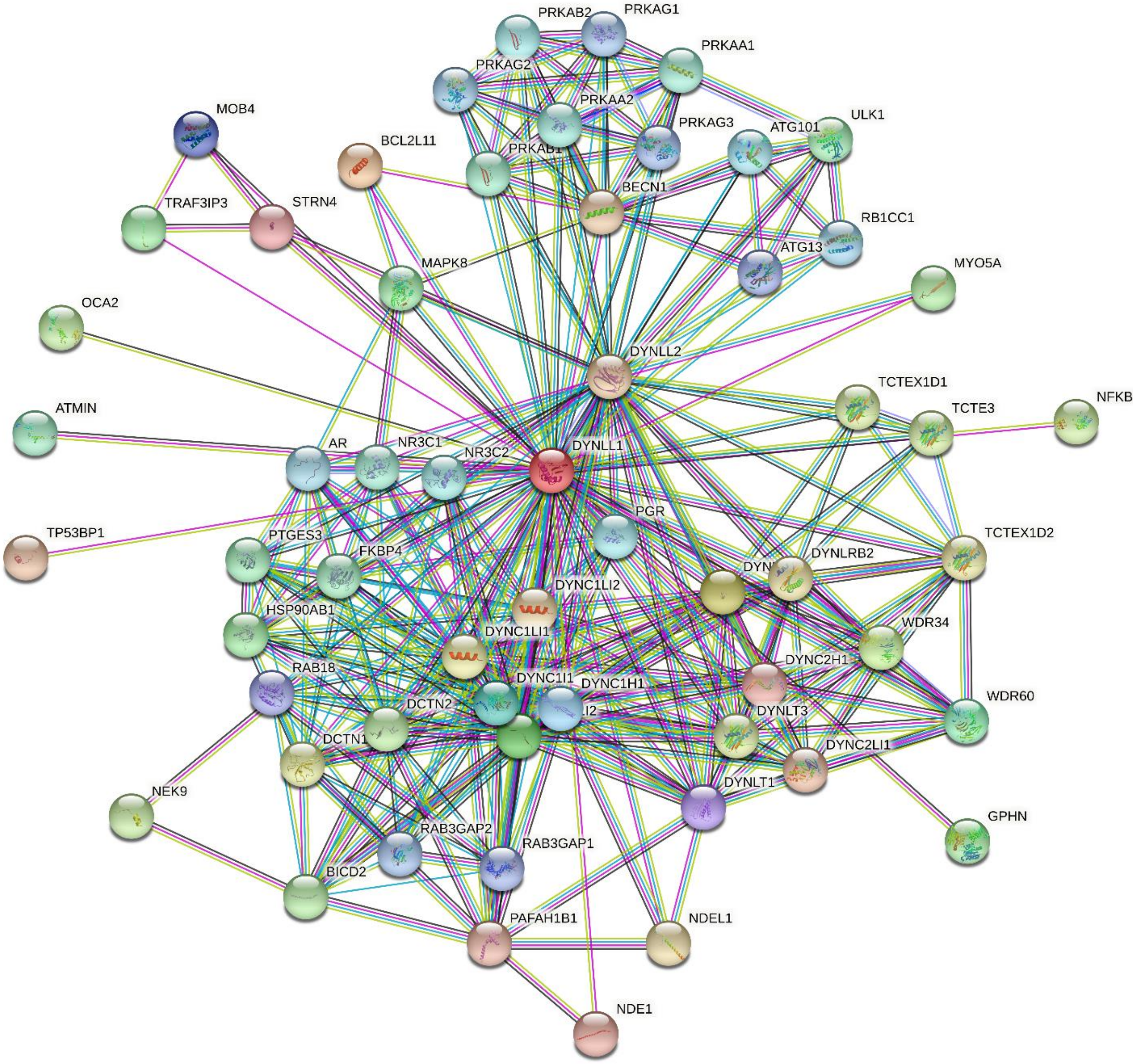

minimum required interaction score: highest confidence (0.900)
number of nodes: 58
number of edges: 338
average node degree: 11.7
avg. local clustering coefficient: 0.828
expected number of edges: 68
PPI enrichment p-value: < 1.0e-16

DYNLL2 (UniProt ID: Q96FJ2)

>sp|Q96FJ2|DYL2_HUMAN Dynein light chain 2, cytoplasmic OS=Homo sapiens OX=9606 GN=DYNLL2 PE=1 SV=1

MSDRKAVIKNADMSEDMQQDAVDCATQAMEKYNIEKDIAAYIKKEFDKKYNPTWHCI VGRNFGSYVTHETKHFIYFYLGQVAILLFKSG

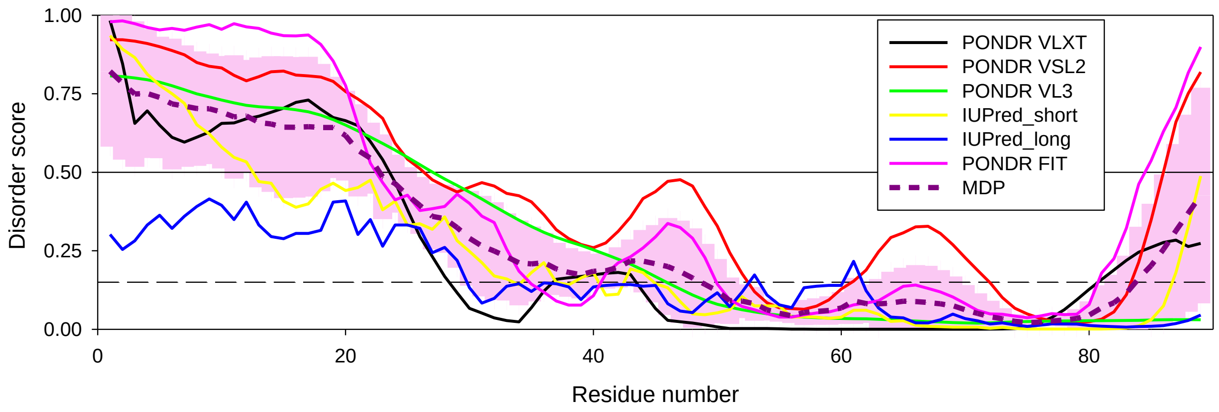

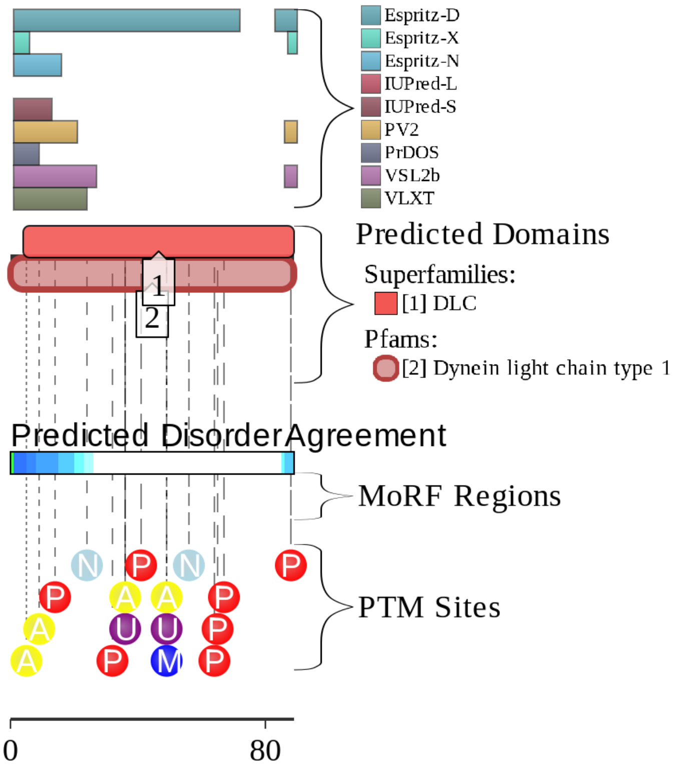

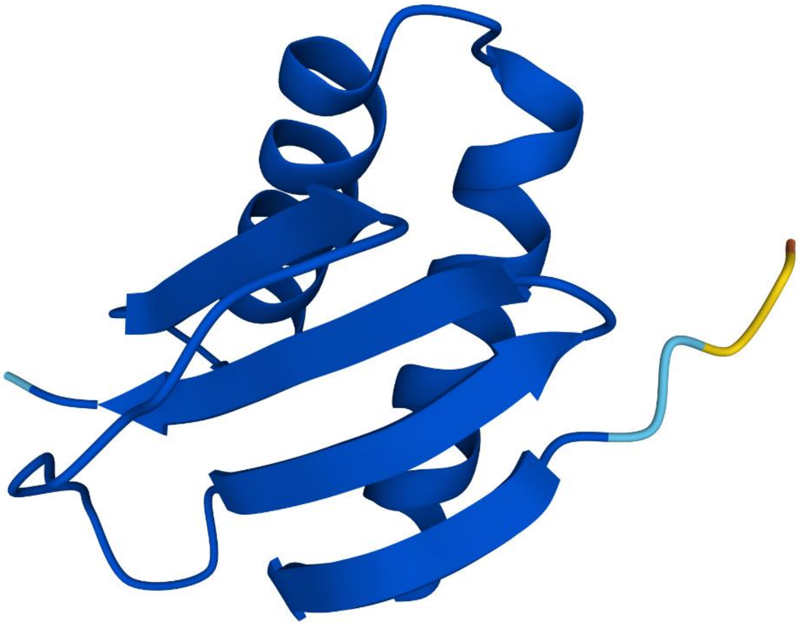

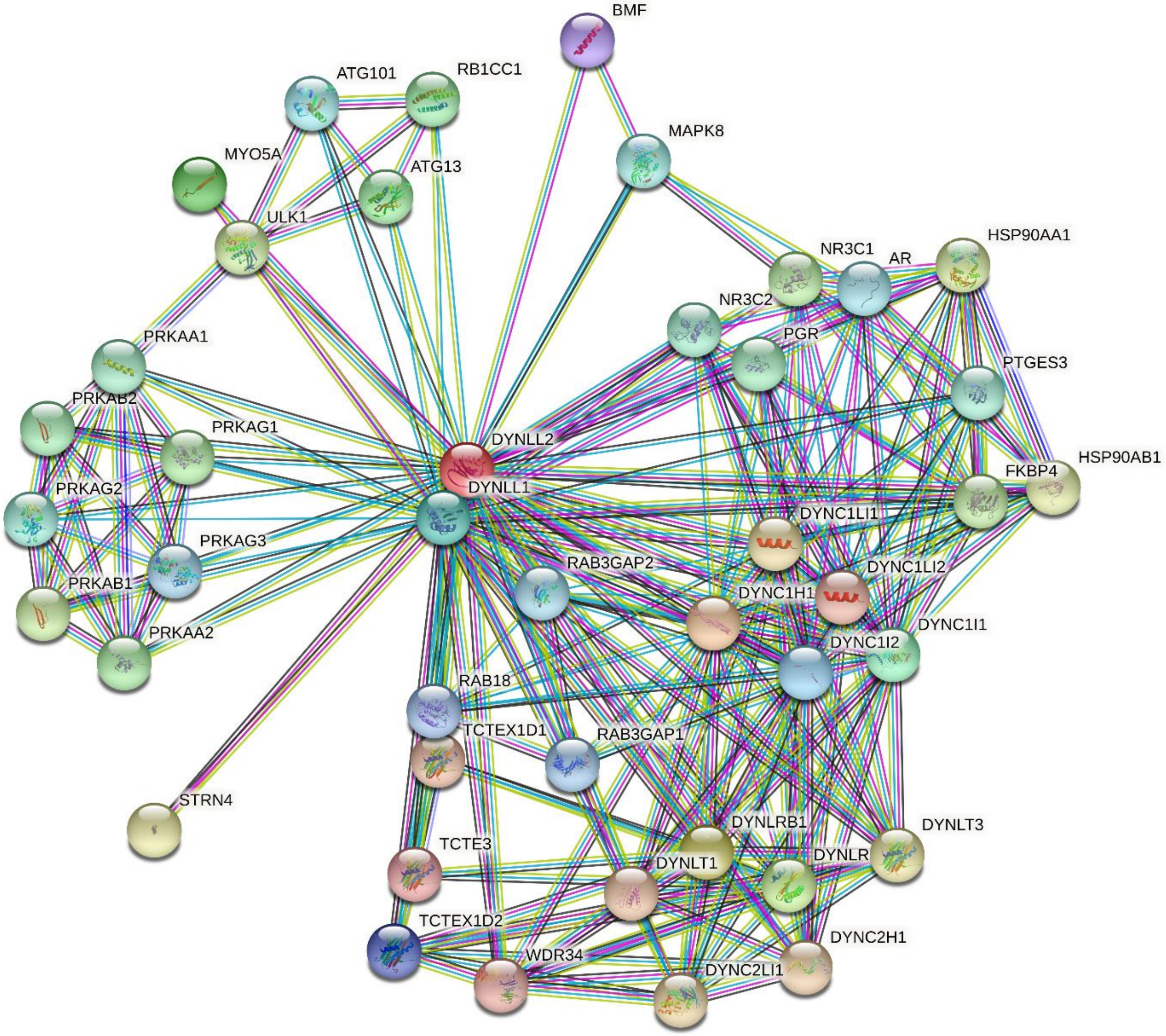

minimum required interaction score: highest confidence (0.900)
number of nodes: 43
number of edges: 257
average node degree: 12
avg. local clustering coefficient: 0.847
expected number of edges: 50
PPI enrichment p-value: < 1.0e-16

STAT1 (UniProt ID: P42224)

>sp|P42224|STAT1_HUMAN Signal transducer and activator of transcription 1-alpha/beta OS=Homo sapiens OX=9606 GN=STAT1 PE=1 SV=2

MSQWYELQQLDSKFLEQVHQLYDDSFPMEIRQYLAQWLEKQDWEHAANDVSFATIRF
HDLLSQLDDQYSRFSLENNFLLQHNIRKSKRNLQDNFQEDPIQMSMIIYSCLKEERKILEN
AQRFNQAQSGNIQSTVMLDKQKELDSKVRNVKDKVMCIEHEIKSLEDLQDEYDFKCKT
LQNREHETNGVAKSDQKQEQLLLKKMYLMLDNKRKEVVHKIIELLNVTELTQNALIND
ELVEWKRRQQSACIGGPPNACLDQLQNWFTIVAESLQQVRQQLKKLEELEQKYTYEHD
PITKNKQVLWDRTFSLFQQLIQSSFVVERQPCMPTHPQRPLVLKTGVQFTVKLRLLVKL
QELNYNLKVKVLFDKDVNERNTVKGFRKFNILGTHTKVMNMEESTNGSLAAEFRHLQL
KEQKNAGTRTNEGPLIVTEELHSLSFETQLCQPGLVIDLETTSLPVVVISNVSQLPSGWAS
ILWYNMLVAEPRNLSFFLTPPCARWAQLSEVLSWQFSSVTKRGLNVDQLNMLGEKLLG
PNASPDGLIPWTRFCKENINDKNFPFWLWIESILELIKKHLLPLWNDGCIMGFISKERERA
LLKDQQPGTFLLRFSESSREGAITFTWVERSQNGGEPDFHAVEPYTKKELSAVTFPDIIRN
YKVMAAENIPENPLKYLYPNIDKDHAFGKYYSRPKEAPEPMELDGPKGTGYIKTELISVS
EVHPSRLQTTDNLLPMSPEEFDEVSRIVGSVEFDSMMNTV

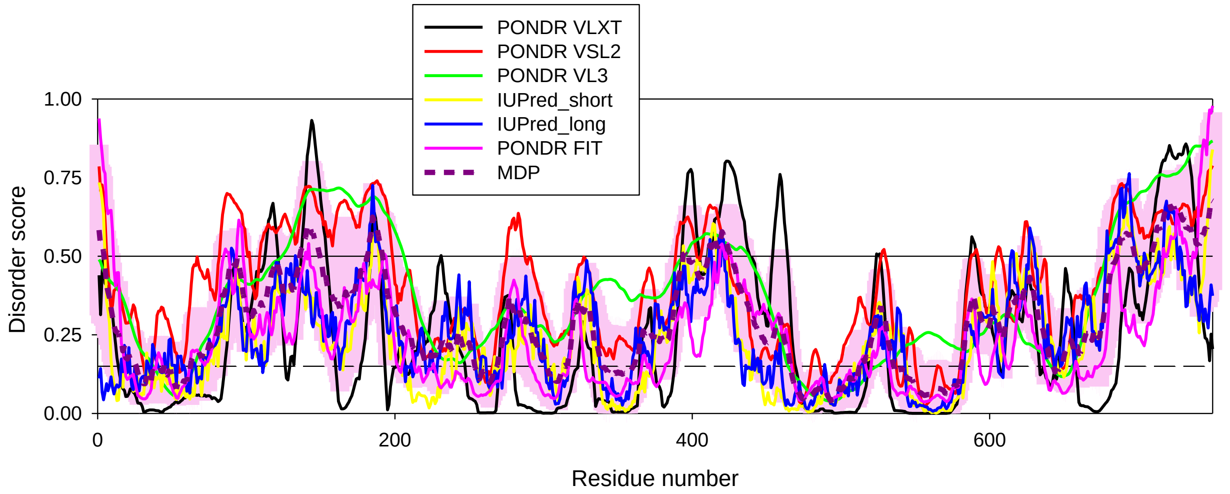

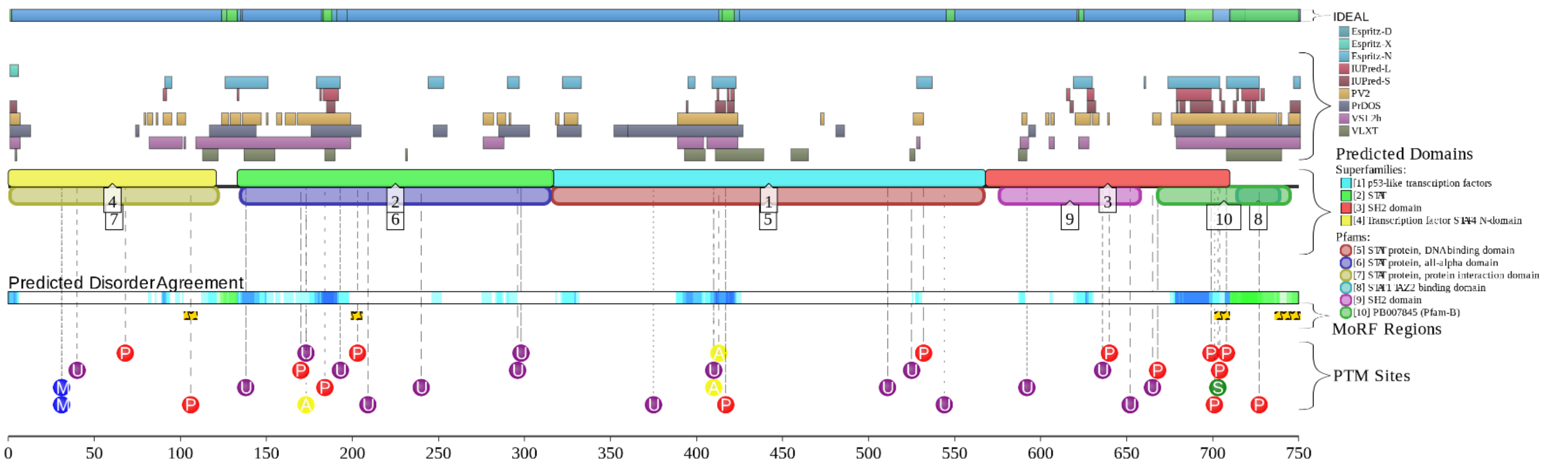

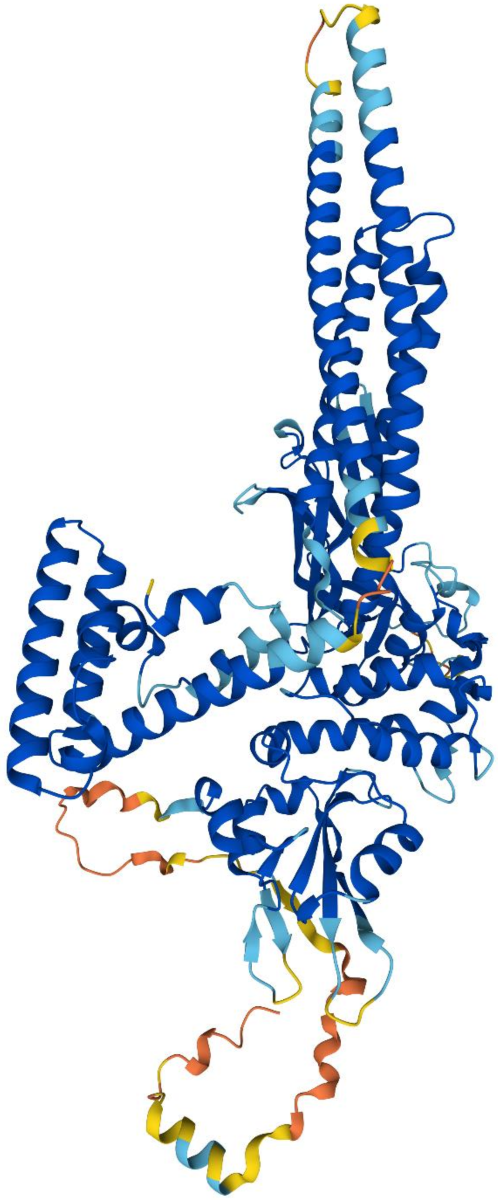

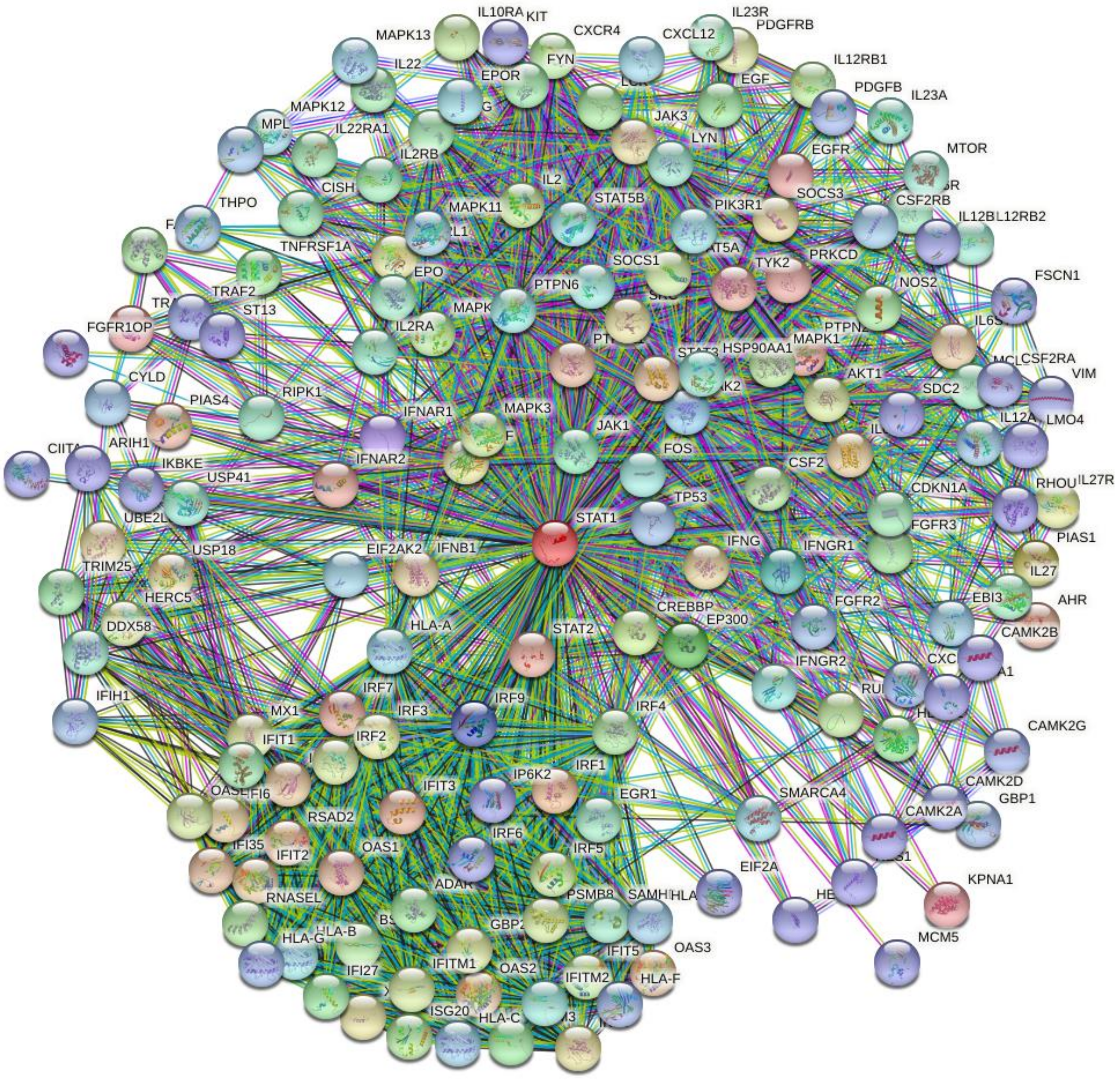

minimum required interaction score: highest confidence (0.900)
number of nodes: 43
number of edges: 257
average node degree: 12
avg. local clustering coefficient: 0.847
expected number of edges: 50
PPI enrichment p-value: < 1.0e-16
number of nodes: 164
number of edges: 2126
average node degree: 25.9
avg. local clustering coefficient: 0.733
expected number of edges: 507
PPI enrichment p-value: < 1.0e-16

STAT2 (UniProt ID: P52630)

>sp|P52630|STAT2_HUMAN Signal transducer and activator of transcription 2 OS=Homo sapiens OX=9606 GN=STAT2 PE=1 SV=1

MAQWEMLQNLDSPFQDQLHQLYSHSLLPVDIRQYLAVWIEDQNWQEAALGSDDSKAT
MLFFHFLDQLNYECGRCSQDPESLLLQHNLRKFCRDIQPFSQDPTQLAEMIFNLLLEEKRI
LIQAQRAQLEQGEPVLETPVESQQHEIESRILDLRAMMEKLVKSISQLKDQQDVFCFRYK
IQAKGKTPSLDPHQTKEQKILQETLNELDKRRKEVLDASKALLGRLTTLIELLLPKLEEW
KAQQQKACIRAPIDHGLEQLETWFTAGAKLLFHLRQLLKELKGLSCLVSYQDDPLTKGV
DLRNAQVTELLQRLLHRAFVVETQPCMPQTPHRPLILKTGSKFTVRTRLLVRLQEGNES
LTVEVSIDRNPPQLQGFRKFNILTSNQKTLTPEKGQSQGLIWDFGYLTLVEQRSGGSGKG
SNKGPLGVTEELHIISFTVKYTYQGLKQELKTDTLPVVIISNMNQLSIAWASVLWFNLLSP
NLQNQQFFSNPPKAPWSLLGPALSWQFSSYVGRGLNSDQLSMLRNKLFGQNCRTEDPL
LSWADFTKRESPPGKLPFWTWLDKILELVHDHLKDLWNDGRIMGFVSRSQERRLLKKT
MSGTFLLRFSESSEGGITCSWVEHQDDDKVLIYSVQPYTKEVLQSLPLTEIIRHYQLLTEE
NIPENPLRFLYPRIPRDEAFGCYYQEKVNLQERRKYLKHRLIVVSNRQVDELQQPLELKP
EPELESLELELGLVPEPELSLDLEPLLKAGLDLGPELESVLESTLEPVIEPTLCMVSQTVPE
PDQGPVSQPVPEPDLPCDLRHLNTEPMEIFRNCVKIEEIMPNGDPLLAGQNTVDEVYVSR
PSHFYTDGPLMPSDF

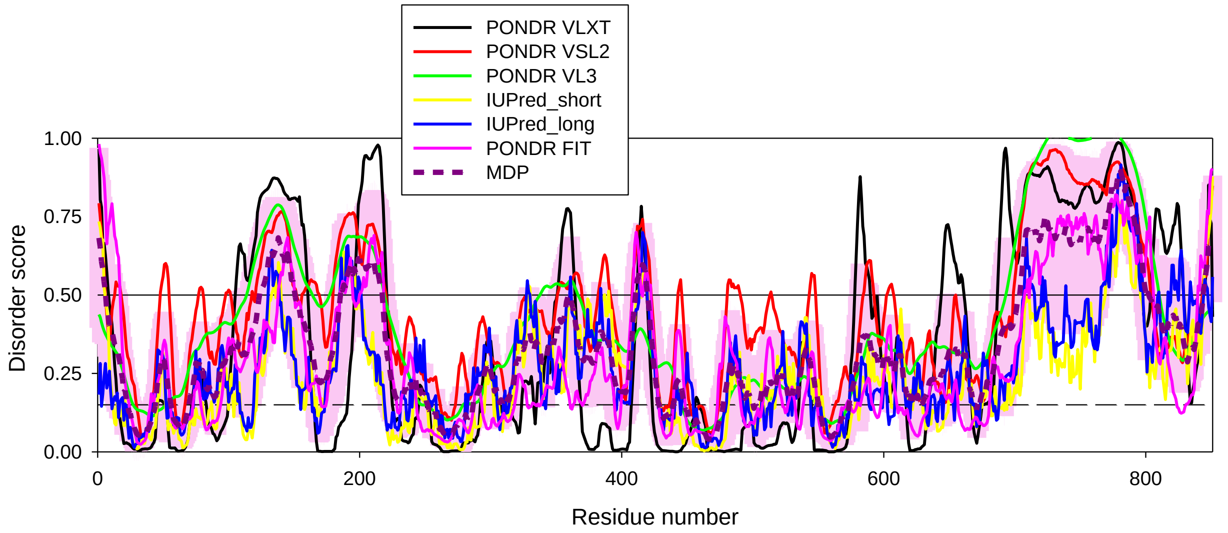

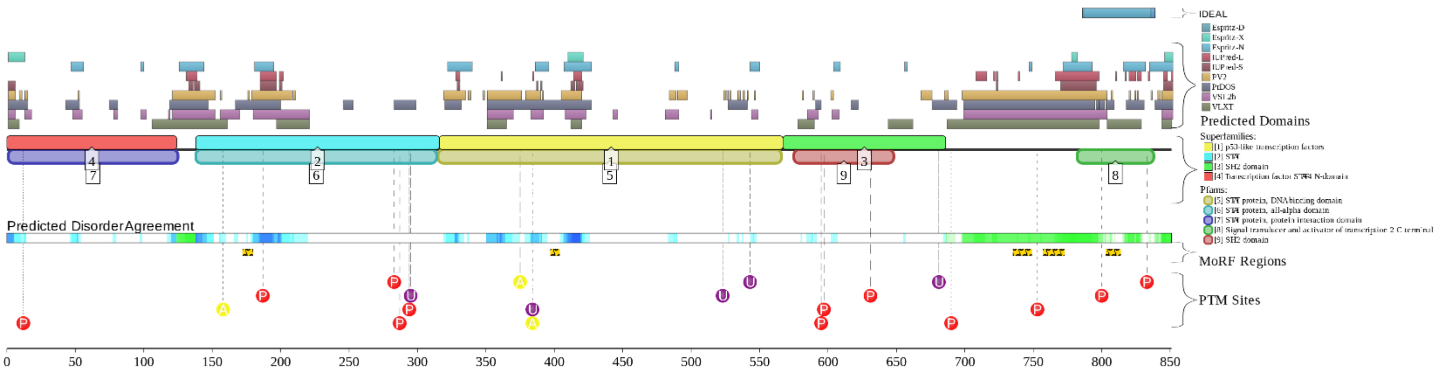

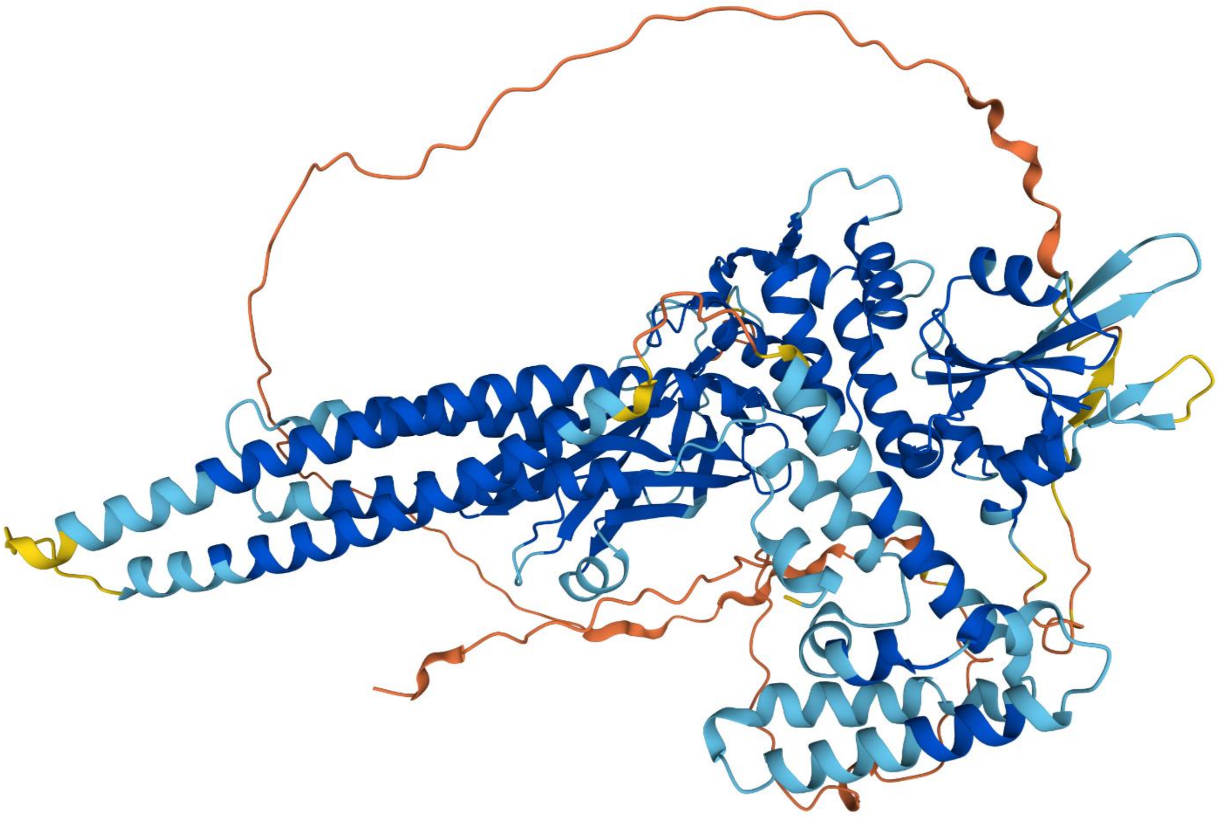

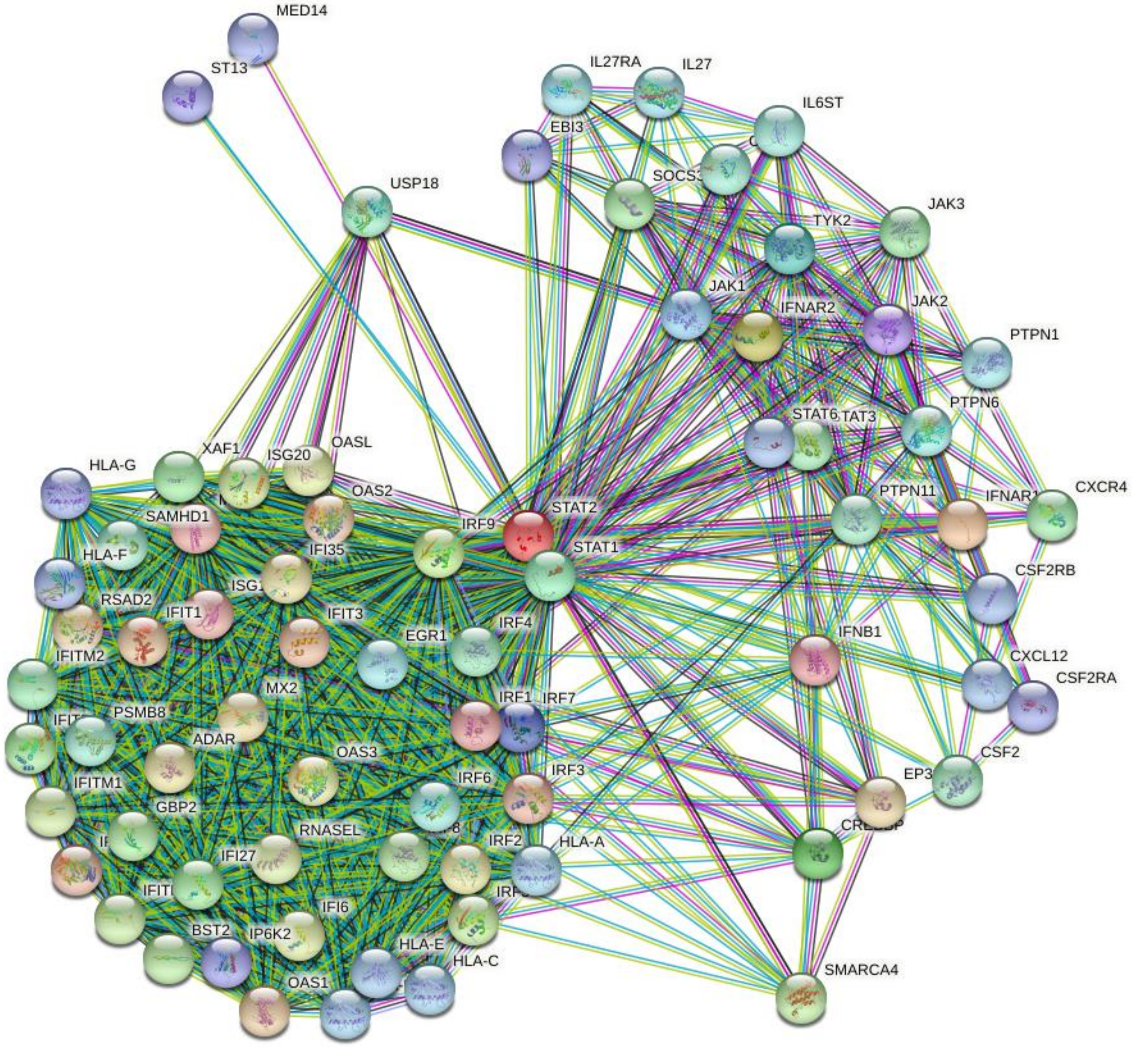

minimum required interaction score: highest confidence (0.900)
number of nodes: 74
number of edges: 1198
average node degree: 32.4
avg. local clustering coefficient: 0.872
expected number of edges: 128
PPI enrichment p-value: < 1.0e-16

STAT3 (UniProt ID: P40763)

>sp|P40763|STAT3_HUMAN Signal transducer and activator of transcription 3 OS=Homo sapiens OX=9606 GN=STAT3 PE=1 SV=2

MAQWNQLQQLDTRYLEQLHQLYSDSFPMELRQFLAPWIESQDWAYAASKESHATLVF
HNLLGEIDQQYSRFLQESNVLYQHNLRRIKQFLQSRYLEKPMEIARIVARCLWEESRLLQ
TAATAAQQGGQANHPTAAVVTEKQQMLEQHLQDVRKRVQDLEQKMKVVENLQDDFD
FNYKTLKSQGDMQDLNGNNQSVTRQKMQQLEQMLTALDQMRRSIVSELAGLLSAMEY
VQKTLTDEELADWKRRQQIACIGGPPNICLDRLENWITSLAESQLQTRQQIKKLEELQQK
VSYKGDPIVQHRPMLEERIVELFRNLMKSAFVVERQPCMPMHPDRPLVIKTGVQFTTKV
RLLVKFPELNYQLKIKVCIDKDSGDVAALRGSRKFNILGTNTKVMNMEESNNGSLSAEF
KHLTLREQRCGNGGRANCDASLIVTEELHLITFETEVYHQGLKIDLETHSLPVVVISNICQ
MPNAWASILWYNMLTNNPKNVNFFTKPPIGTWDQVAEVLSWQFSSTTKRGLSIEQLTTL
AEKLLGPGVNYSGCQITWAKFCKENMAGKGFSFWVWLDNIIDLVKKYILALWNEGYIM
GFISKERERAILSTKPPGTFLLRFSESSKEGGVTFTWVEKDISGKTQIQSVEPYTKQQLNN
MSFAEIIMGYKIMDATNILVSPLVYLYPDIPKEEAFGKYCRPESQEHPEADPGSAAPYLK
TKFICVTPTTCSNTIDLPMSPRTLDSLMQFGNNGEGAEPSAGGQFESLTFDMELTSECATS
PM

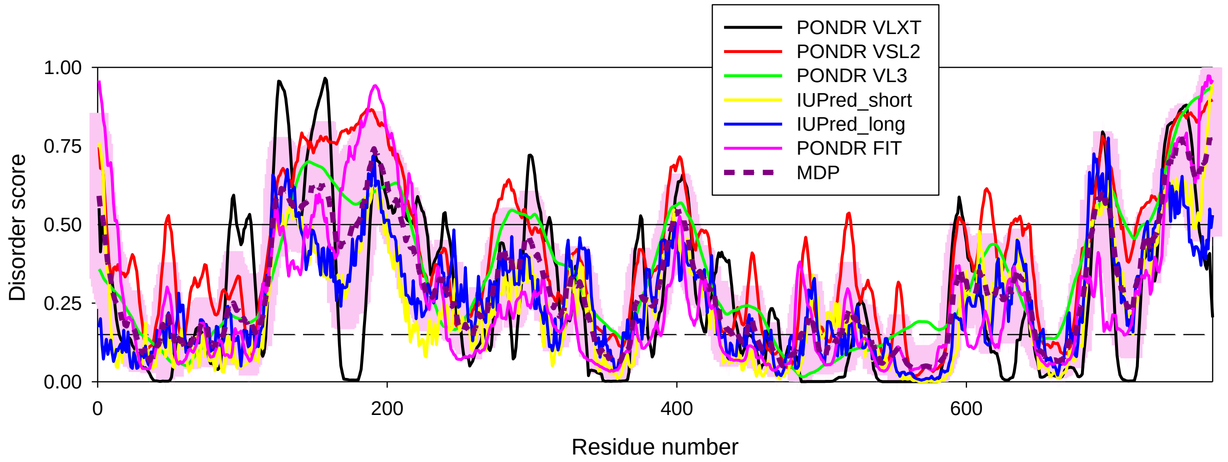

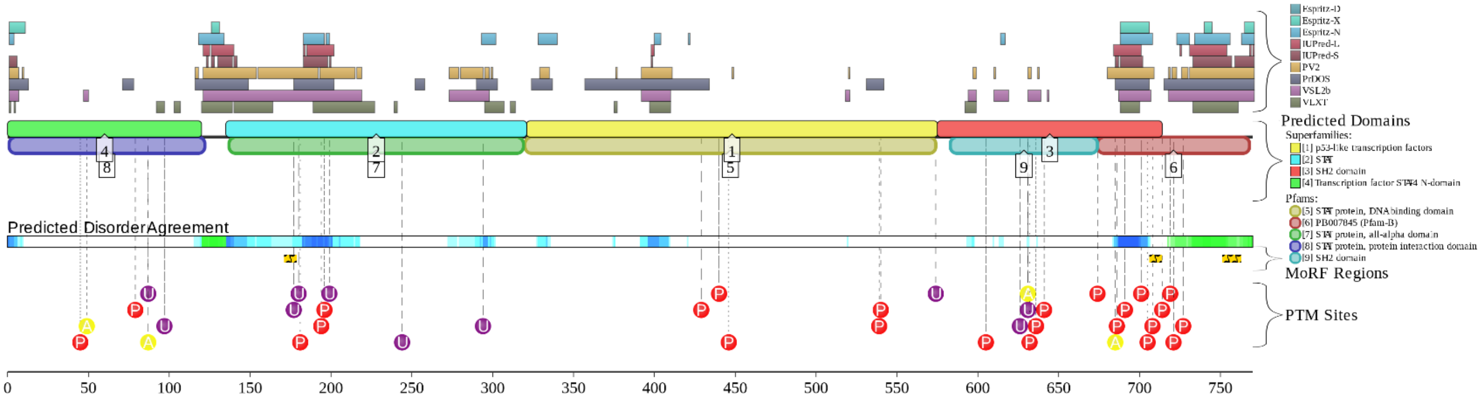

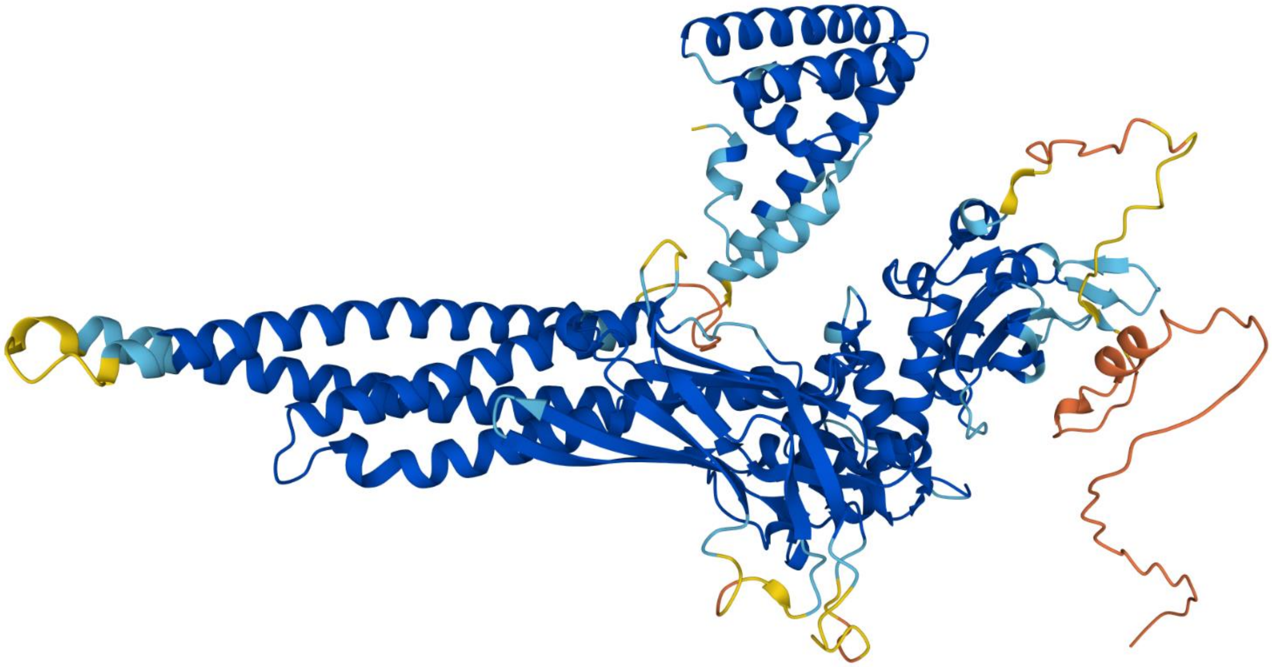

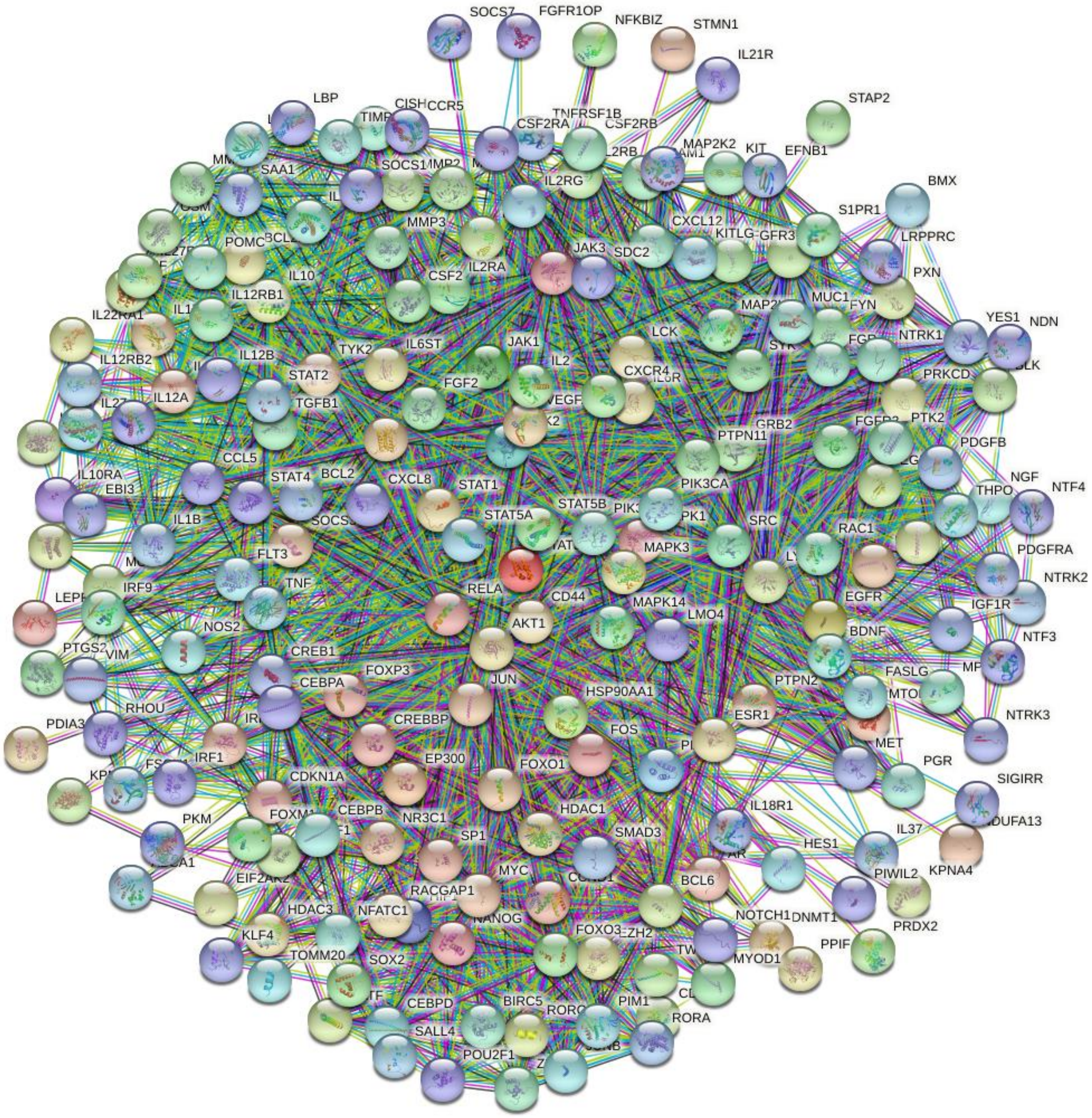

minimum required interaction score: highest confidence (0.900)

number of nodes: 201

number of edges: 2286

average node degree: 22.7

avg. local clustering coefficient: 0.643

expected number of edges: 702

PPI enrichment p-value: < 1.0e-16

PML protein (UniProt ID: P29590)

>sp|P29590|PML_HUMAN Protein PML OS=Homo sapiens OX=9606 GN=PML PE=1 SV=3

MEPAPARSPRPQQDPARPQEPTMPPPETPSEGRQPSPSPSPTERAPASEEEFQFLRCQQCQ
AEAKCPKLLPCLHTLCSGCLEASGMQCPICQAPWPLGADTPALDNVFFESLQRRLSVYR
QIVDAQAVCTRCKESADFWCFECEQLLCAKCFEAHQWFLKHEARPLAELRNQSVREFL
DGTRKTNNIFCSNPNHRTPTLTSIYCRGCSKPLCCSCALLDSSHSELKCDISAEIQQRQEEL
DAMTQALQEQDSAFGAVHAQMHAAVGQLGRARAETEELIRERVRQVVAHVRAQEREL
LEAVDARYQRDYEEMASRLGRLDAVLQRIRTGSALVQRMKCYASDQEVLDMHGFLRQ
ALCRLRQEEPQSLQAAVRTDGFDEFKVRLQDLSSCITQGKDAAVSKKASPEAASTPRDPI
DVDLPEEAERVKAQVQALGLAEAQPMAVVQSVPGAHPVPVYAFSIKGPSYGEDVSNTT
TAQKRKCSQTQCPRKVIKMESEEGKEARLARSSPEQPRPSTSKAVSPPHLDGPPSPRSPVI
GSEVFLPNSNHVASGAGEAEERVVVISSSEDSDAENSSSRELDDSSSESSDLQLEGPSTLR
VLDENLADPQAEDRPLVFFDLKIDNETQKISQLAAVNRESKFRVVIQPEAFFSIYSKAVSL
EVGLQHFLSFLSSMRRPILACYKLWGPGLPNFFRALEDINRLWEFQEAISGFLAALPLIRE
RVPGASSFKLKNLAQTYLARNMSERSAMAAVLAMRDLCRLLEVSPGPQLAQHVYPFSS
LQCFASLQPLVQAAVLPRAEARLLALHNVSFMELLSAHRRDRQGGLKKYSRYLSLQTT
TLPPAQPAFNLQALGTYFEGLLEGPALARAEGVSTPLAGRGLAERASQQS

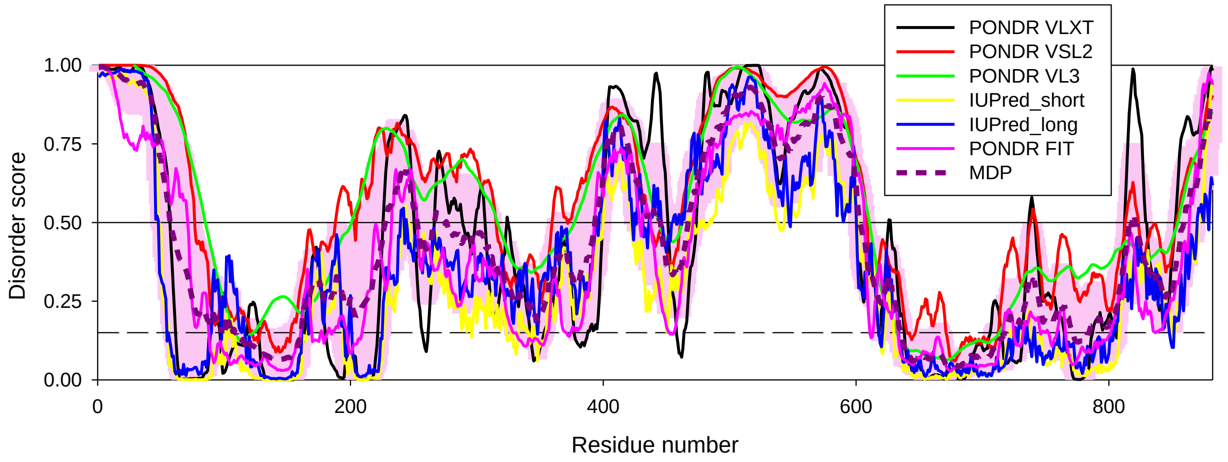

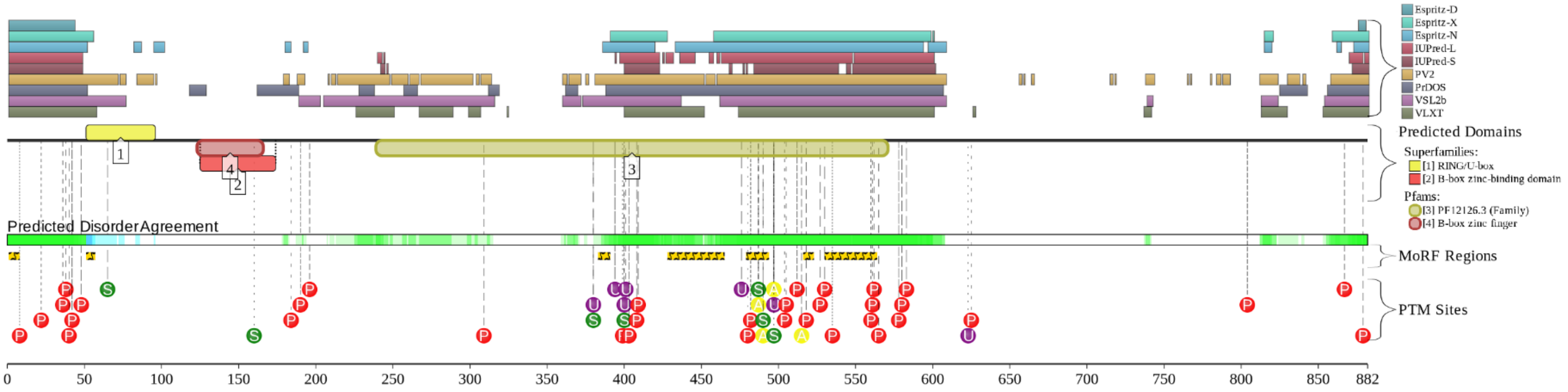

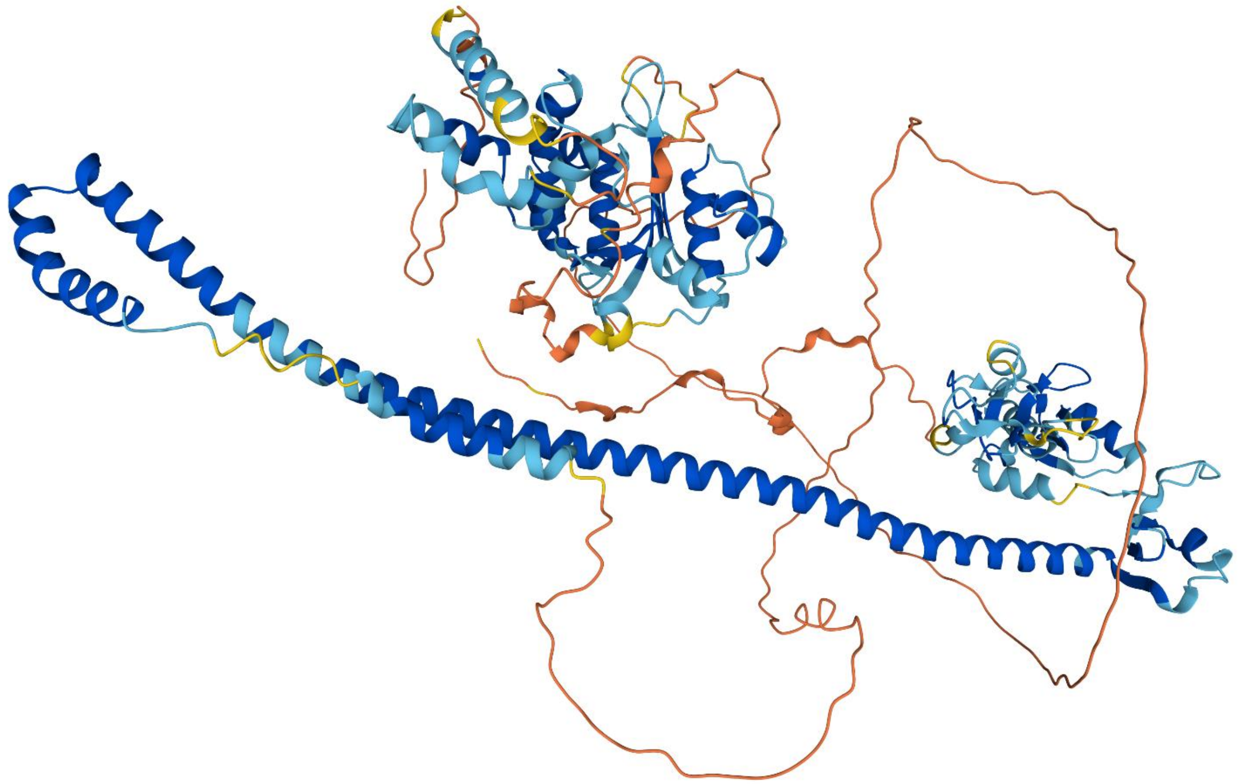

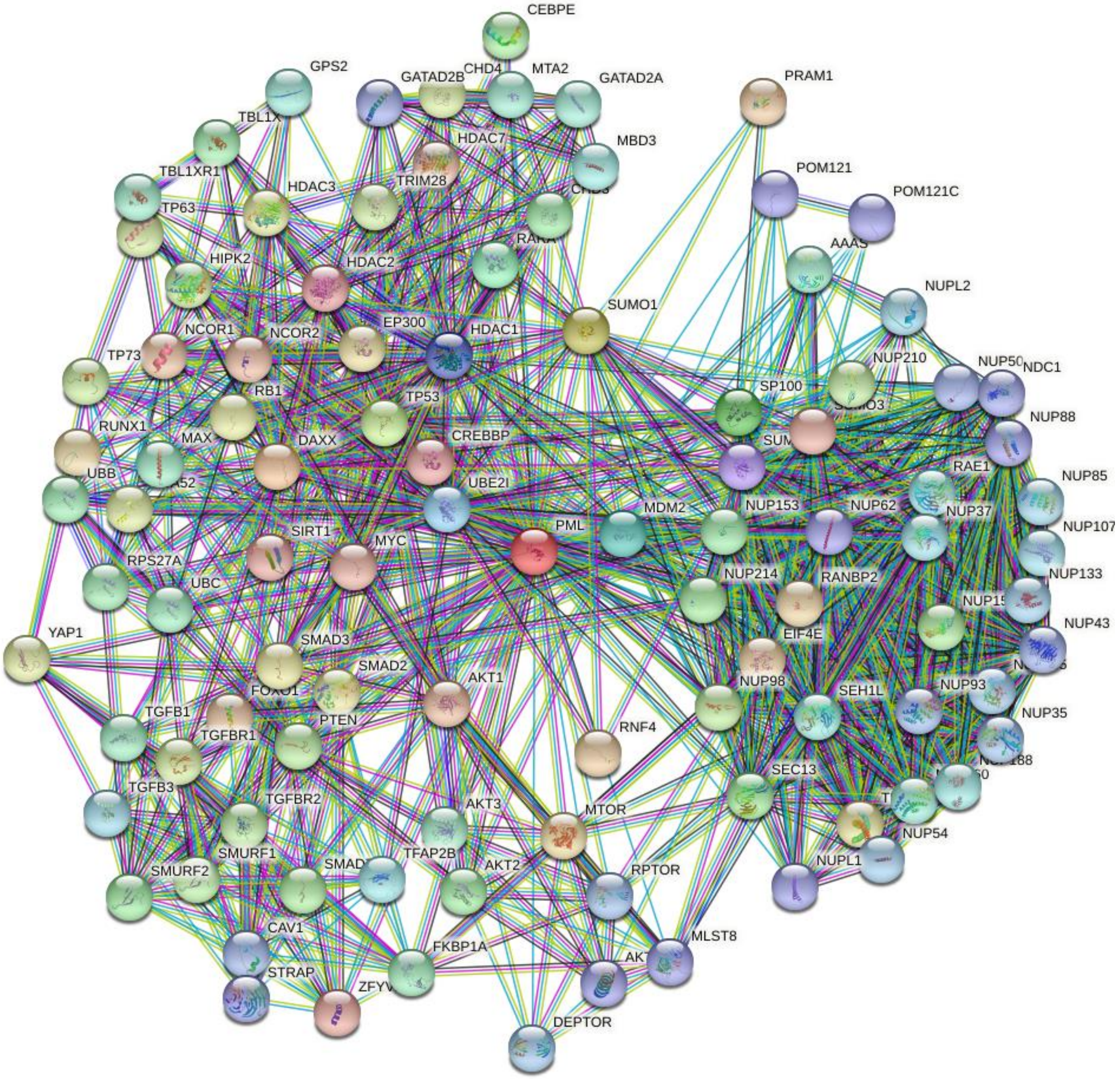

minimum required interaction score: highest confidence (0.900)
number of nodes: 99
number of edges: 989
average node degree: 20
avg. local clustering coefficient: 0.733
expected number of edges: 331
PPI enrichment p-value: < 1.0e-16

Ribosomal protein L9 (UniProt ID: Q02878)

>sp|Q02878|RL6_HUMAN 60S ribosomal protein L6 OS=Homo sapiens OX=9606 GN=RPL6 PE=1 SV=3

MAGEKVEKPDTKEKKPEAKKVDAGGKVKKGNLKAKKPKKGKPHCSRNPVLVRGIGRY
SRSAMYSRKAMYKRKYSAAKSKVEKKKKEKVLATVTKPVGGDKNGGTRVVKLRKMP
RYYPTEDVPRKLLSHGKKPFSQHVRKLRASITPGTILIILTGRHRGKRVVFLKQLASGLLL
VTGPLVLNRVPLRRTHQKFVIATSTKIDISNVKIPKHLTDAYFKKKKLRKPRHQEGEIFDT
EKEKYEITEQRKIDQKAVDSQILPKIKAIPQLQGYLRSVFALTNGIYPHKLVF

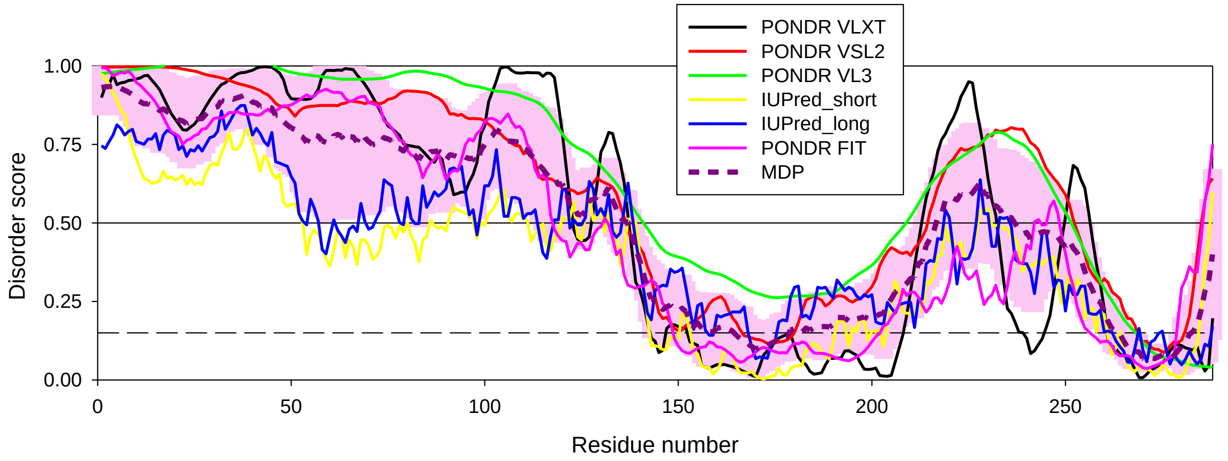

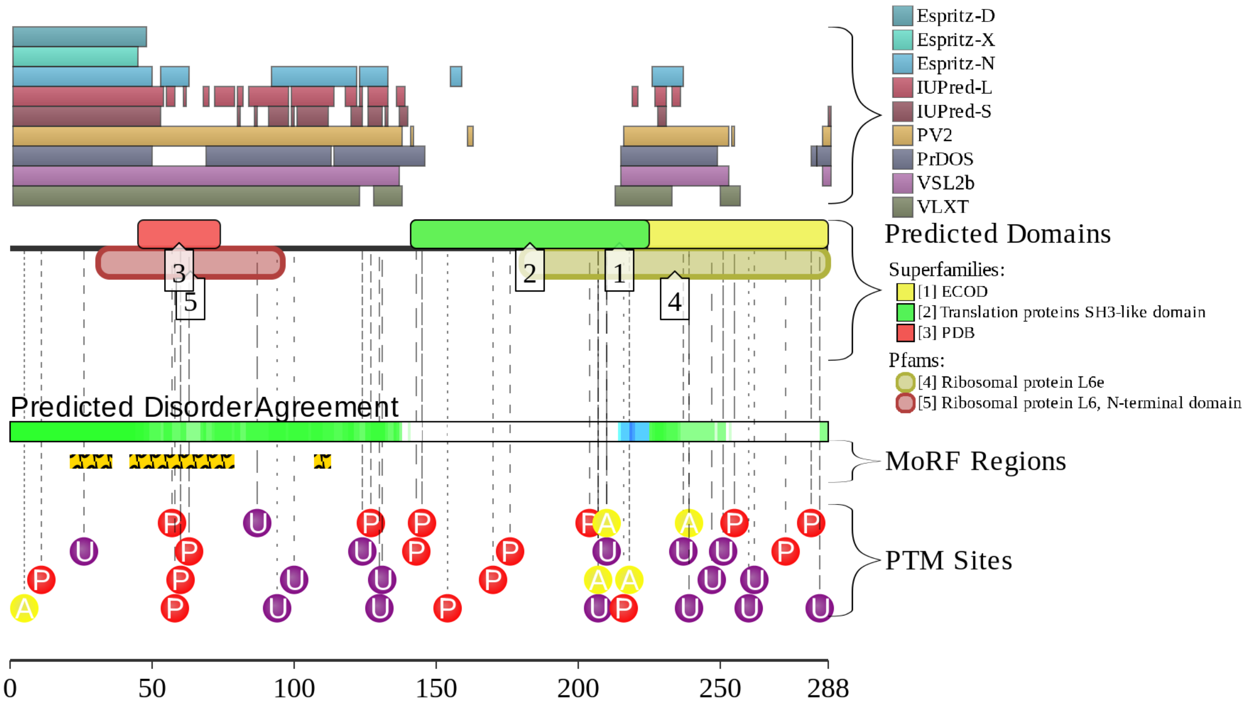

Espritz-D
Espritz-X
Espritz-N
IUPred-L
IUPred-S
PV2
PrDOS
VSL2b
VLXT
Predicted Domains
Superfamilies:
[1] ECOD
[2] Translation proteins SH3-like domain
[3] PDB
Pfams:
[4] Ribosomal protein L6e
[5] Ribosomal protein L6, N-terminal domain
Predicted Disorder Agreement
MoRF Regions
PTM Sites
0
50
100
150
200
250
288

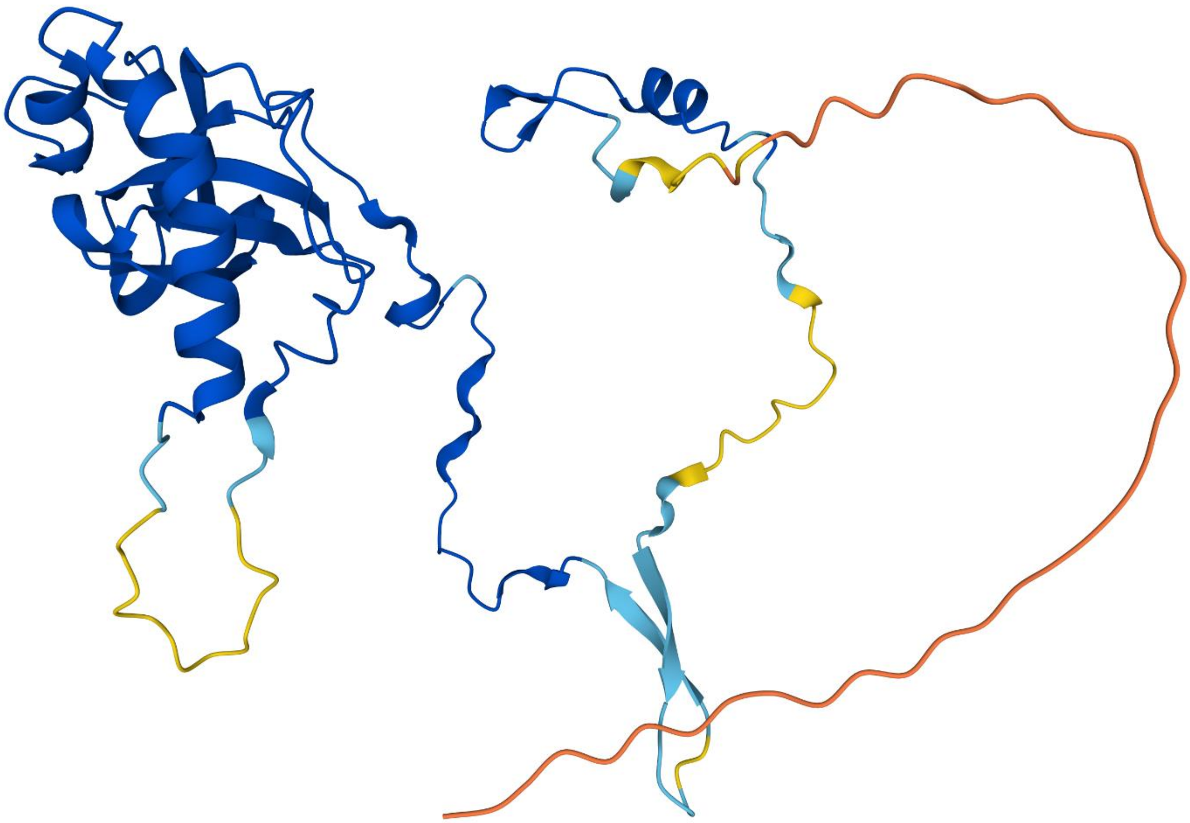

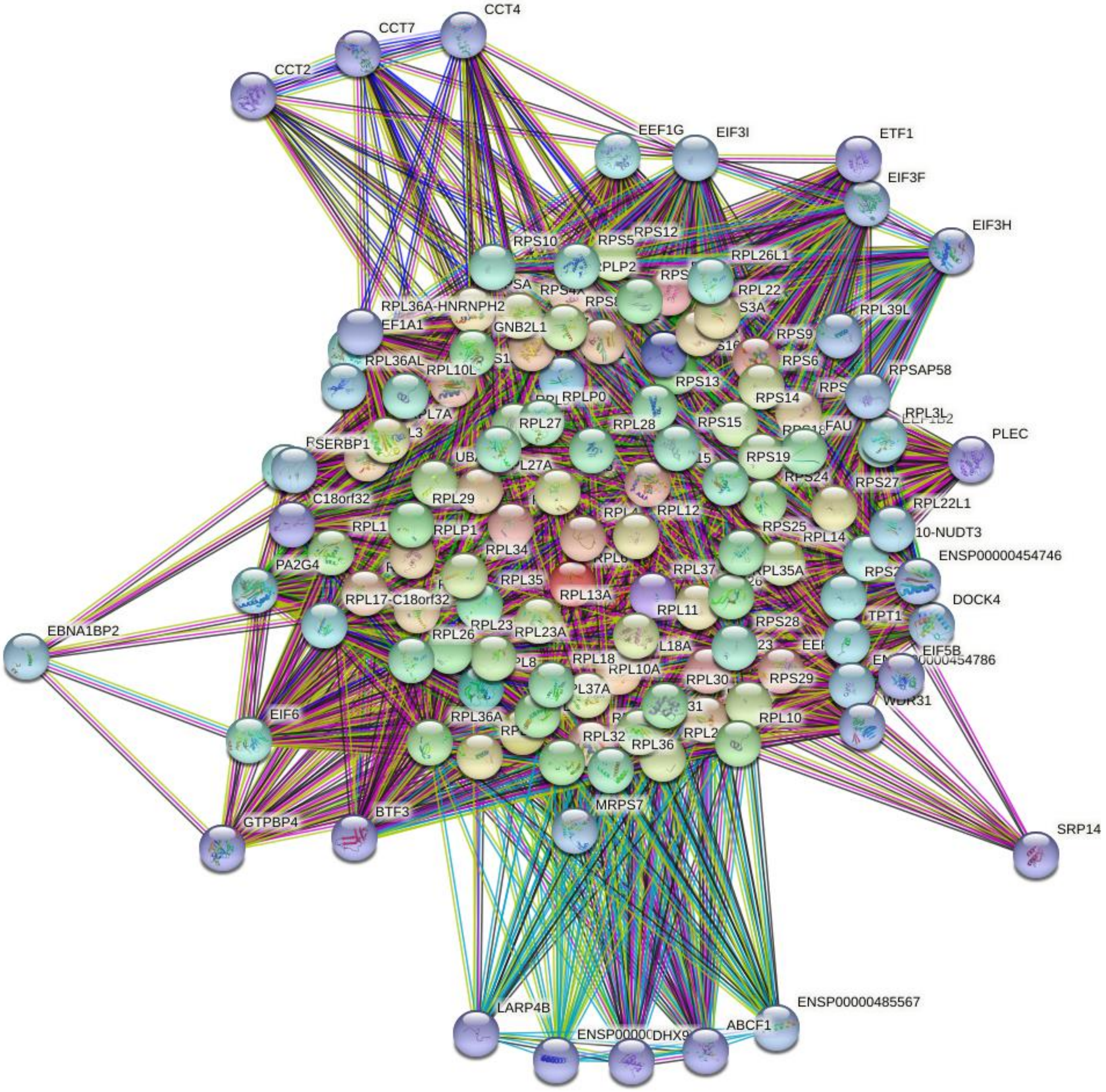

minimum required interaction score: highest confidence (0.900)
number of nodes: 123
number of edges: 5605
average node degree: 91.1
avg. local clustering coefficient: 0.909
expected number of edges: 612
PPI enrichment p-value: < 1.0e-16

NCL (UniProt ID: P19338)

>sp|P19338|NUCL_HUMAN Nucleolin OS=Homo sapiens OX=9606 GN=NCL PE=1 SV=3

MVKLAKAGKNQGDPKKMAPPPKEVEEDSEDEEMSEDEEDDSSGEEVVIPQKKGKKAA
ATSAKKVVVSPTKKVAVATPAKKAAVTPGKKAAATPAKKTVTPAKAVTTPGKKGATP
GKALVATPGKKGAAIPAKGAKNGKNAKKEDSDEEEDDDSEEDEEDDEDEDEDEDEIEP
AAMKAAAAAPASEDEDDEDDEDDEDDDDDEEDDSEEEAMETTPAKGKKAAKVVPVK
AKNVAEDEDEEEDDEDEDDDDDEDDEDDDDEDDEEEEEEEEEEEPVKEAPGKRKKEMA
KQKAAPEAKKQKVEGTEPTTAFNLFVGNLNFNKSAPELKTGISDVFAKNDLAVVDVRIG
MTRKFGYVDFESAEDLEKALELTGLKVFGNEIKLEKPKGKDSKKERDARTLLAKNLPY
KVTQDELKEVFEDAAEIRLVSKDGKSKGIAYIEFKTEADAEKTFEEKQGTEIDGRSISLYY
TGEKGQNQDYRGGKNSTWSGESKTLVLSNLSYSATEETLQEVFEKATFIKVPQNQNGKS
KGYAFIEFASFEDAKEALNSCNKREIEGRAIRLELQGPRGSPNARSQPSKTLFVKGLSEDT
TEETLKESFDGSVRARIVTDRETGSSKGFGFVDFNSEEDAKAAKEAMEDGEIDGNKVTL
DWAKPKGEGGFGGRGGGRGGFGGRGGGRGGRGGFGGRGRGGFGGRGGFRGGRGGG
GDHKPQGKKTKFE

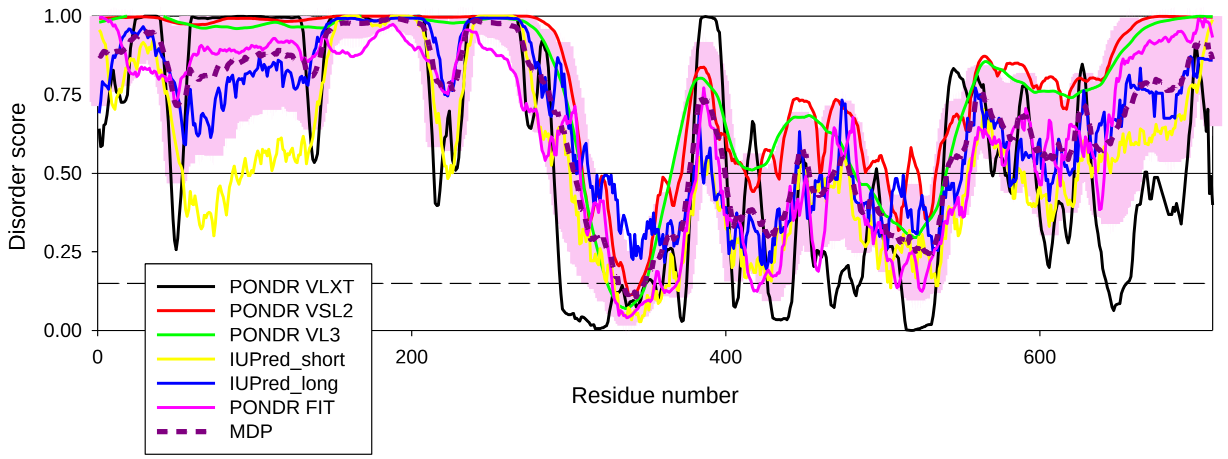

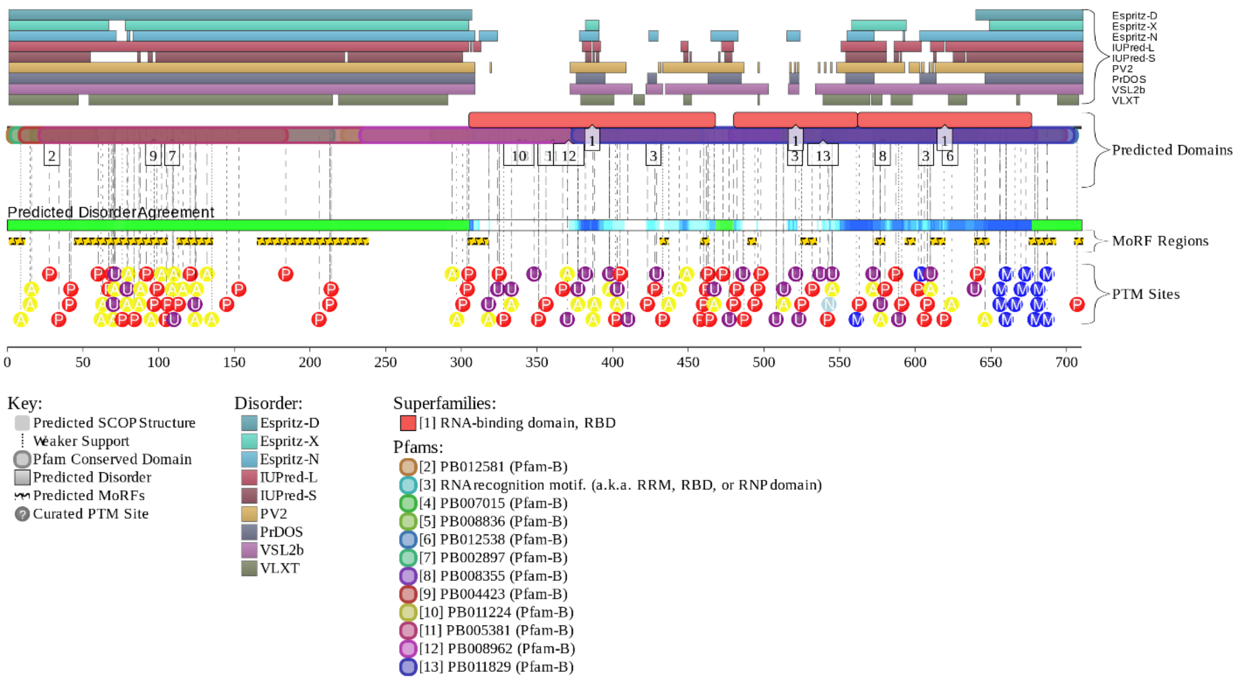
Espritz-D
Espritz-X
Espritz-N
IUPred-L
IUPred-S
PV2
PrDOS
VSL2b
VLXT
Predicted Domains
Predicted DisorderAgreement
MoRF Regions
PTM Sites
0
50
100
150
200
250
300
350
400
450
500
550
600
650
700
Key:
Predicted SCOP Structure
Weaker Support
Pfam Conserved Domain
Predicted Disorder
Predicted MoRFs
Curated PTM Site
Disorder:
Espritz-D
Espritz-X
Espritz-N
IUPred-L
IUPred-S
PV2
PrDOS
VSL2b
VLXT
Superfamilies:
[1] RNA-binding domain, RBD
Pfams:
[2] PB012581 (Pfam-B)
[3] RNA recognition motif. (a.k.a. RRM, RBD, or RNP domain)
[4] PB007015 (Pfam-B)
[5] PB008836 (Pfam-B)
[6] PB012538 (Pfam-B)
[7] PB002897 (Pfam-B)
[8] PB008355 (Pfam-B)
[9] PB004423 (Pfam-B)
[10] PB011224 (Pfam-B)
[11] PB005381 (Pfam-B)
[12] PB008962 (Pfam-B)
[13] PB011829 (Pfam-B)

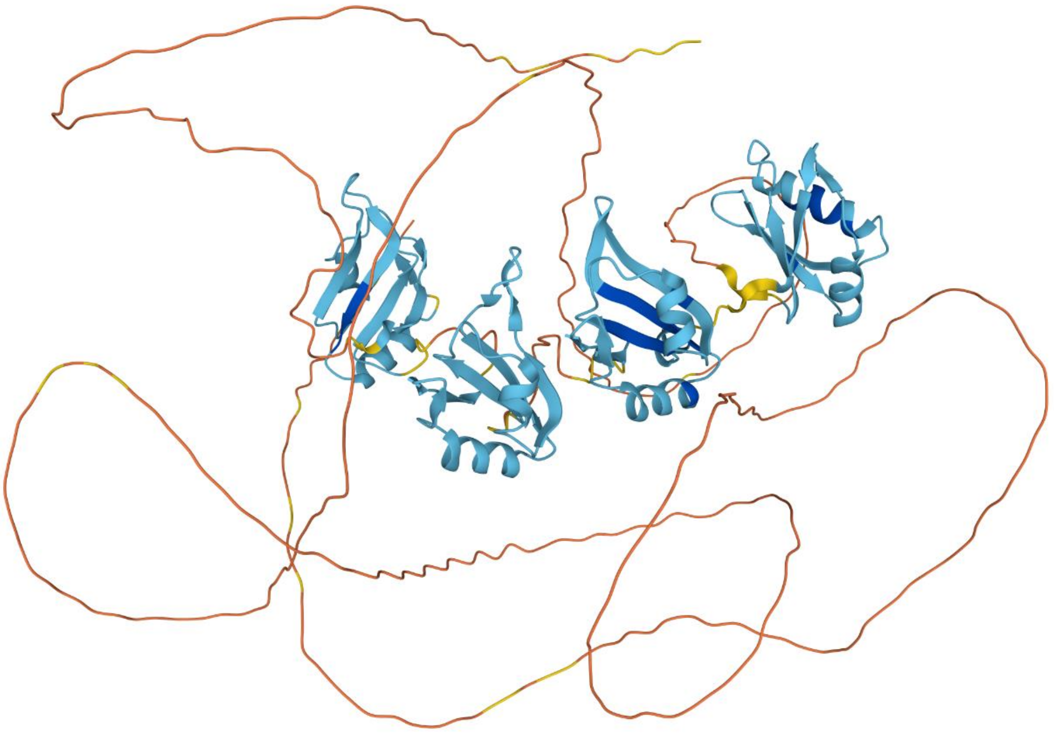

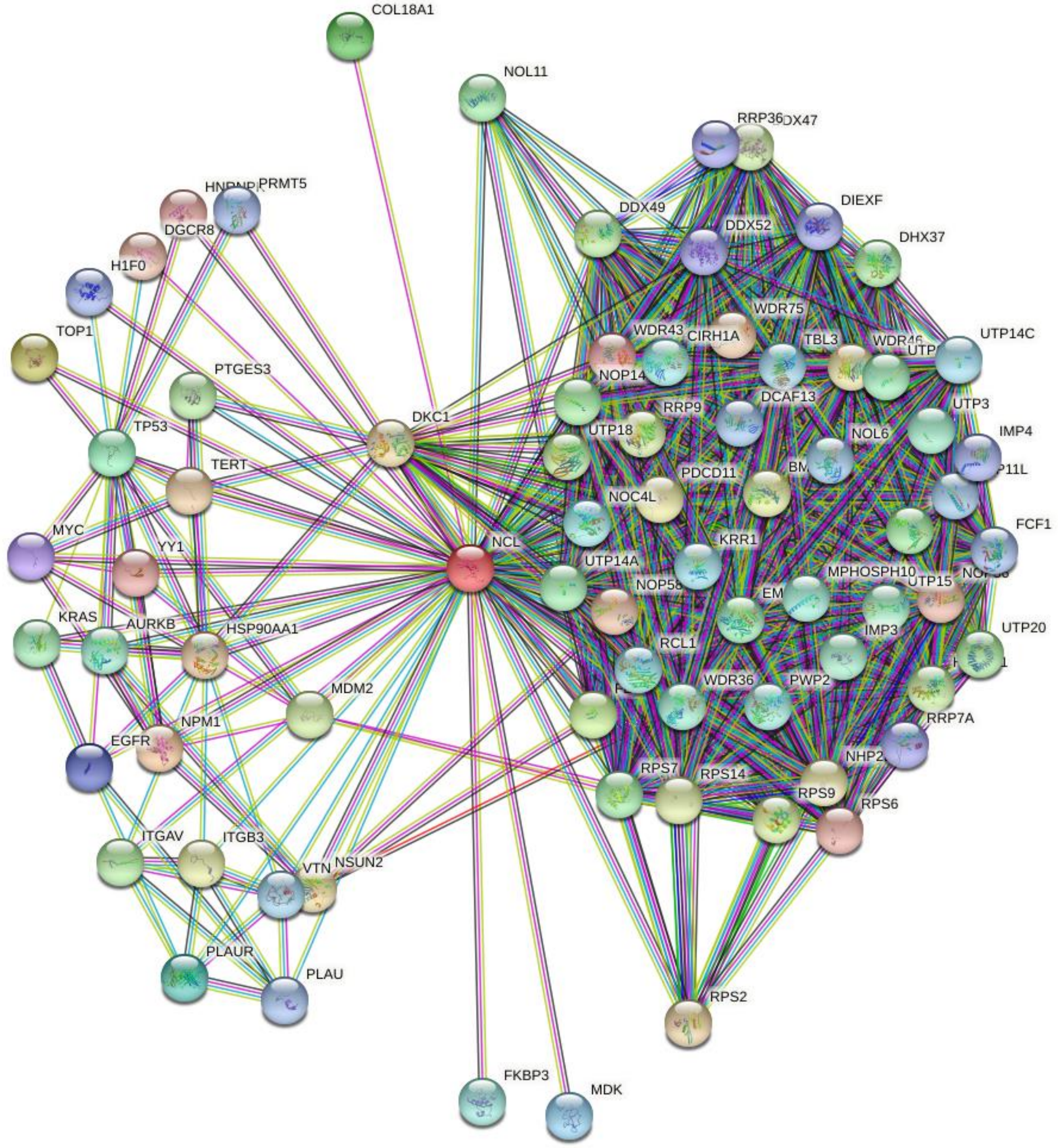

minimum required interaction score: highest confidence (0.900)
number of nodes: 75
number of edges: 1128
average node degree: 30.1
avg. local clustering coefficient: 0.882
expected number of edges: 200
PPI enrichment p-value: < 1.0e-16

FAK (UniProt ID: Q05397)

>sp|Q05397|FAK1_HUMAN Focal adhesion kinase 1 OS=Homo sapiens OX=9606 GN=PTK2 PE=1 SV=2

MAAAYLDPNLNHTPNSSTKTHLGTGMERSPGAMERVLKVFHYFESNSEPTTWASIIRHG
DATDVRGIIQKIVDSHKVKHVACYGFRLSHLRSEEVHWLHVDMGVSSVREKYELAHPP
EEWKYELRIRYLPKGFLNQFTEDKPTLNFFYQQVKSDYMLEIADQVDQEIALKLGCLEIR
RSYWEMRGNALEKKSNYEVLEKDVGLKRFFPKSLLDSVKAKTLRKLIQQTFRQFANLN
REESILKFFEILSPVYRFDKECFKCALGSSWIISVELAIGPEEGISYLTDKGCNPTHLADFT
QVQTIQYSNSEDKDRKGMLQLKIAGAPEPLTVTAPSLTIAENMADLIDGYCRLVNGTSQ
SFIIRPQKEGERALPSIPKLANSEKQGMRTHAVSVSETDDYAEIIDEEDTYTMPSTRDYEI
QRERIELGRCIGEGQFGDVHQGIYMSPENPALAVAIKTCKNCTSDSVREKFLQEALTMR
QFDHPHIVKLIGVITENPVWIIMELCTLGELRSFLQVRKYSLDLASLILYAYQLSTALAYL
ESKRFVHRDIAARNVLVSSNDCVKLGDFGLSRYMEDSTYYKASKGKLPIKWMAPESINF
RRFTSASDVWMFGVCMWEILMHGVKPFQGVKNNDVIGRIENGERLPMPPNCPPTLYSL
MTKCWAYDPSRRPRFTELKAQLSTILEEEKAQQEERMRMESRRQATVSWDSGGSDEAP
PKPSRPGYPSPRSSEGFYPSPQHMVQTNHYQVSGYPGSHGITAMAGSIYPGQASLLDQTD
SWNHRPQEIAMWQPNVEDSTVLDLRGIGQVLPTHLMEERLIRQQQEMEEDQRWLEKEE
RFLKPDVRLSRGSIDREDGSLQGPIGNQHIYQPVGKPDPAAPPKKPPRPGAPGHLGSLAS
LSSPADSYNEGVKLQPQEISPPPTANLDRSNDKVYENVTGLVKAVIEMSSKIQPAPPEEY
VPMVKEVGLALRTLLATVDETIPLLPASTHREIEMAQKLLNSDLGELINKMKLAQQYVM
TSLQQEYKKQMLTAAHALAVDAKNLLDVIDQARLKMLGQTRPH

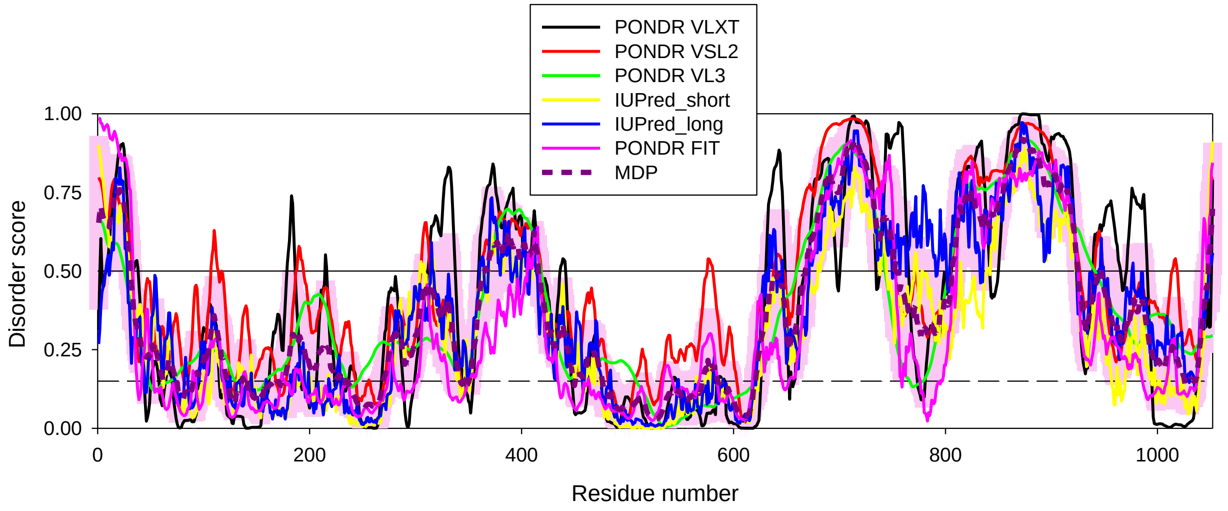

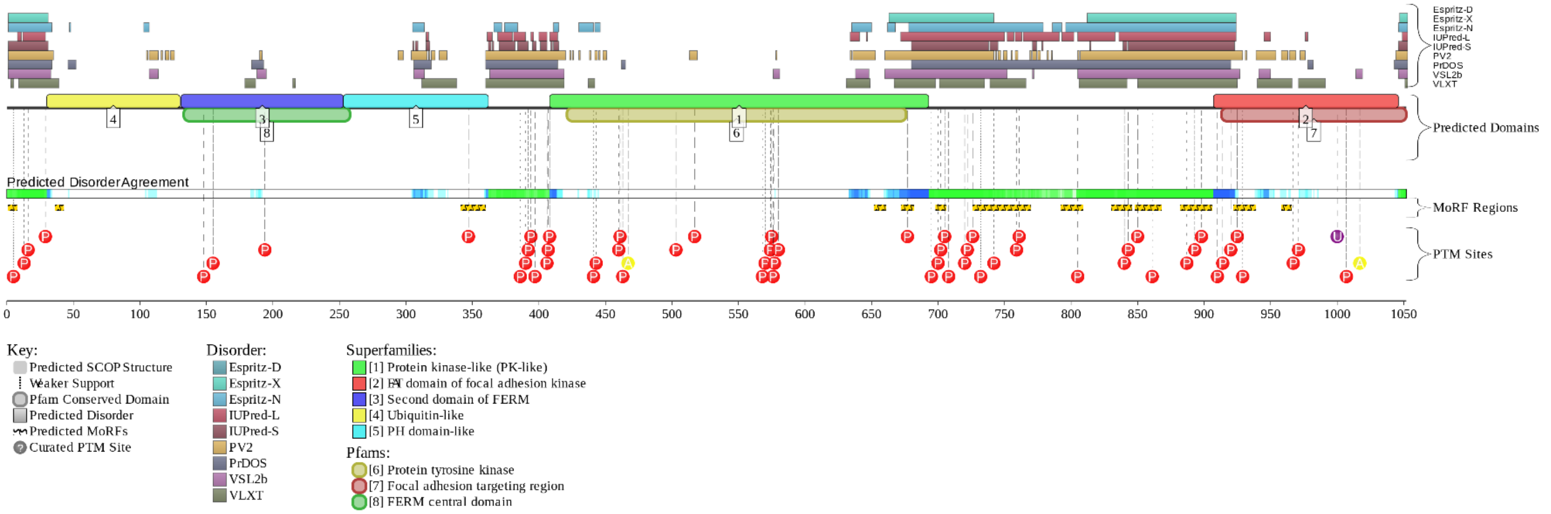

Espritz-D
Espritz-X
Espritz-N
IUPred-L
IUPred-S
PV2
PrDOS
VSL2b
VLXT
Predicted Domains
Predicted Disorder Agreement
MoRF Regions
PTM Sites
0
50
100
150
200
250
300
350
400
450
500
550
600
650
700
750
800
850
900
950
1000
1050
Key:
Predicted SCOP Structure
Weaker Support
Pfam Conserved Domain
Predicted Disorder
Predicted MoRFs
Curated PTM Site
Disorder:
Espritz-D
Espritz-X
Espritz-N
IUPred-L
IUPred-S
PV2
PrDOS
VSL2b
VLXT
Superfamilies:
[1] Protein kinase-like (PK-like)
[2] FAT domain of focal adhesion kinase
[3] Second domain of FERM
[4] Ubiquitin-like
[5] PH domain-like
Pfams:
[6] Protein tyrosine kinase
[7] Focal adhesion targeting region
[8] FERM central domain

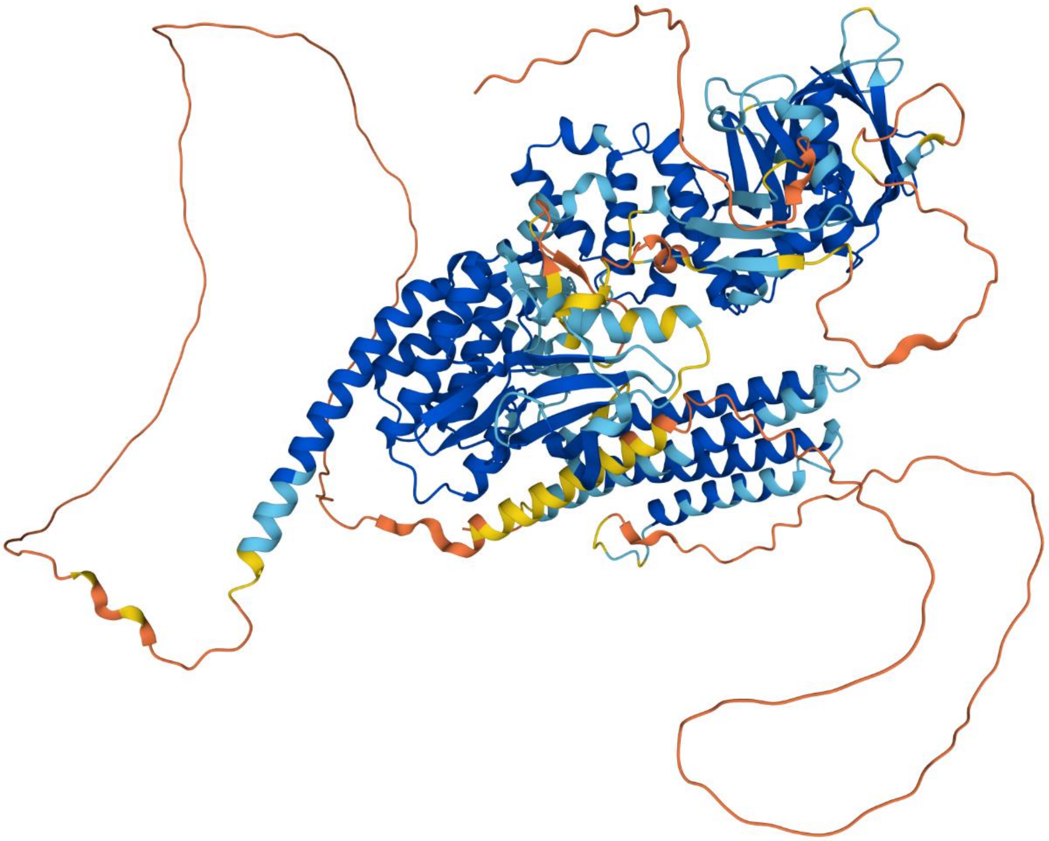

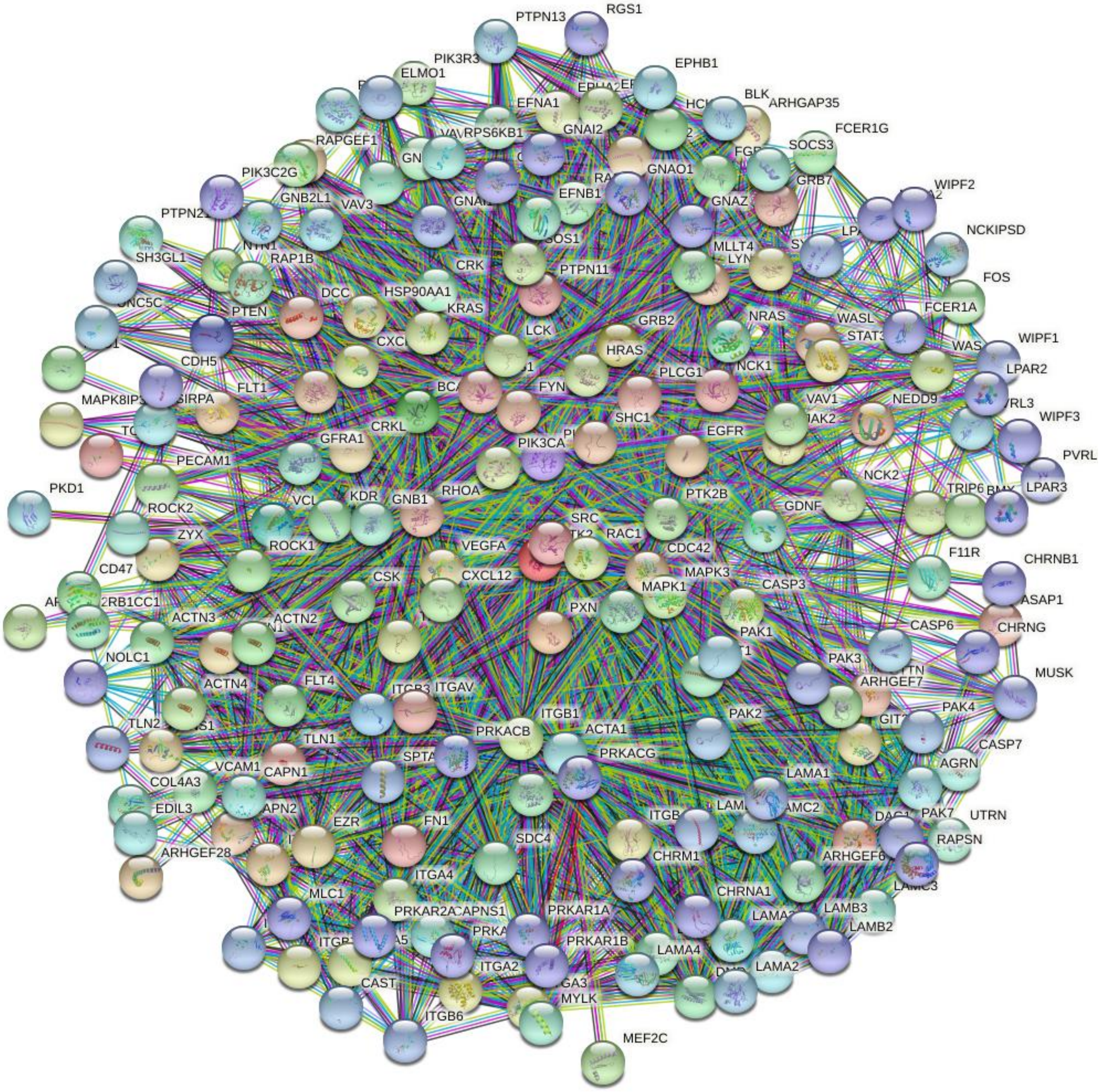

minimum required interaction score: highest confidence (0.900)

number of nodes: 196

number of edges: 2816

average node degree: 28.7

avg. local clustering coefficient: 0.676

expected number of edges: 664

PPI enrichment p-value: < 1.0e-16

JAK1 (UniProt ID: P23458)

>sp|P23458|JAK1_HUMAN Tyrosine-protein kinase JAK1 OS=Homo sapiens OX=9606 GN=JAK1 PE=1 SV=2

MQYLNIKEDCNAMAFCAKMRSSKKTEVNLEAPEPGVEVIFYLSDREPLRLGSGEYTAEE
LCIRAAQACRISPLCHNLFALYDENTKLWYAPNRTITVDDKMSLRLHYRMRFYFTNWH
GTNDNEQSVWRHSPKKQKNGYEKKKIPDATPLLDASSLEYLFAQGQYDLVKCLAPIRD
PKTEQDGHDIENECLGMAVLAISHYAMMKKMQLPELPKDISYKRYIPETLNKSIRQRNL
LTRMRINNVFKDFLKEFNNKTICDSSVSTHDLKVKYLATLETLTKHYGAEIFETSMLLISS
ENEMNWFHSNDGGNVLYYEVMVTGNLGIQWRHKPNVVSVEKEKNKLKRKKLENKHK
KDEEKNKIREEWNNFSYFPEITHIVIKESVVSINKQDNKKMELKLSSHEEALSFVSLVDG
YFRLTADAHHYLCTDVAPPLIVHNIQNGCHGPICTEYAINKLRQEGSEEGMYVLRWSCT
DFDNILMTVTCFEKSEQVQGAQKQFKNFQIEVQKGRYSLHGSDRSFPSLGDLMSHLKKQ
ILRTDNISFMLKRCCQPKPREISNLLVATKKAQEWQPVYPMSQLSFDRILKKDLVQGEHL
GRGTRTHIYSGTLMDYKDDEGTSEEKKIKVILKVLDPSHRDISLAFFEAASMMRQVSHK
HIVYLYGVCVRDVENIMVEEFVEGGPLDLFMHRKSDVLTTPWKFKVAKQLASALSYLE
DKDLVHGNVCTKNLLLAREGIDSECGPFIKLSDPGIPITVLSRQECIERIPWIAPECVEDSK
NLSVAADKWSFGTTLWEICYNGEIPLKDKTLIEKERFYESRCRPVTPSCKELADLMTRC
MNYDPNQRPFFRAIMRDINKLEEQNPDIVSEKKPATEVDPTHFEKRFLKRIRDLGEGHFG
KVELCRYDPEGDNTGEQVAVKSLKPESGGNHIADLKKEIEILRNLYHENIVKYKGICTED
GGNGIKLIMEFLPSGSLKEYLPKNKNKINLKQQLKYAVQICKGMDYLGSRQYVHRDLA
ARNVLVESEHQVKIGDFGLTKAIETDKEYYTVKDDRDSPVFWYAPECLMQSKFYIASDV
WSFGVTLHELLTYCDSDSSPMALFLKMIGPTHGQMTVTRLVNTLKEGKRLPCPPNCPDE
VYQLMRKCWEFQPSNRTSFQNLIEGFEALLK

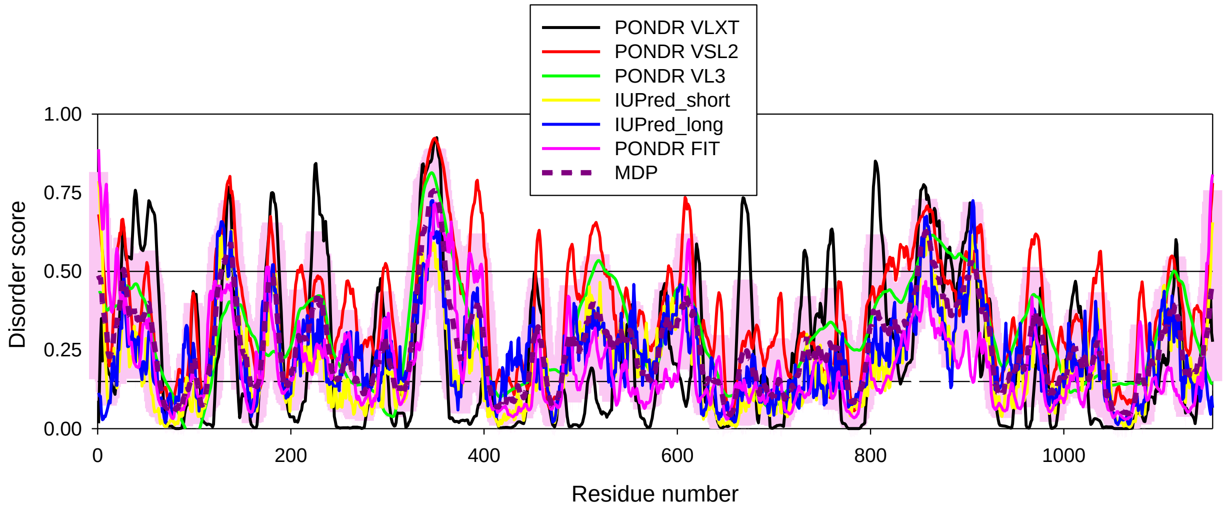

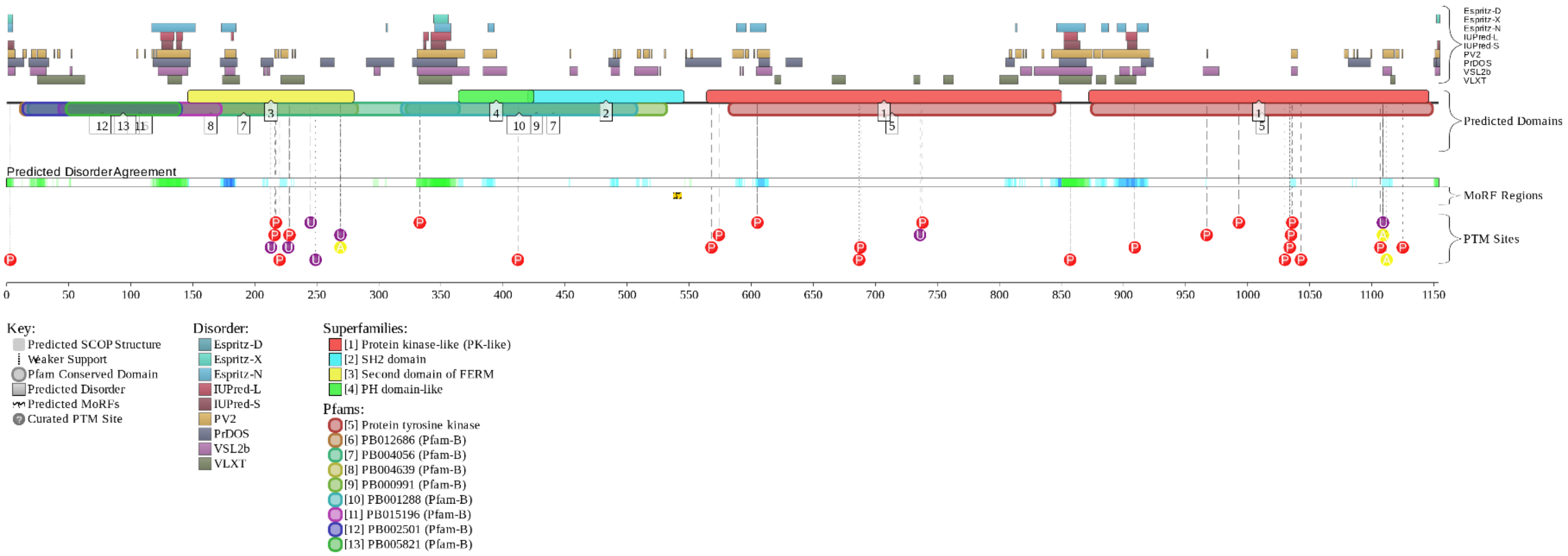
Espritz-D
Espritz-X
Espritz-N
IUPred-L
IUPred-S
PV2
PrDOS
VSL2b
VLXT
Predicted Domains
Predicted DisorderAgreement
MoRF Regions
PTM Sites
0 50 100 150 200 250 300 350 400 450 500 550 600 650 700 750 800 850 900 950 1000 1050 1100 1150
Key:
Predicted SCOP Structure
Weaker Support
Pfam Conserved Domain
Predicted Disorder
Predicted MoRFs
Curated PTM Site
Disorder:
Espritz-D
Espritz-X
Espritz-N
IUPred-L
IUPred-S
PV2
PrDOS
VSL2b
VLXT
Superfamilies:
[1] Protein kinase-like (PK-like)
[2] SH2 domain
[3] Second domain of FERM
[4] PH domain-like
Pfams:
[5] Protein tyrosine kinase
[6] PB012686 (Pfam-B)
[7] PB004056 (Pfam-B)
[8] PB004639 (Pfam-B)
[9] PB000991 (Pfam-B)
[10] PB001288 (Pfam-B)
[11] PB015196 (Pfam-B)
[12] PB002501 (Pfam-B)
[13] PB005821 (Pfam-B)

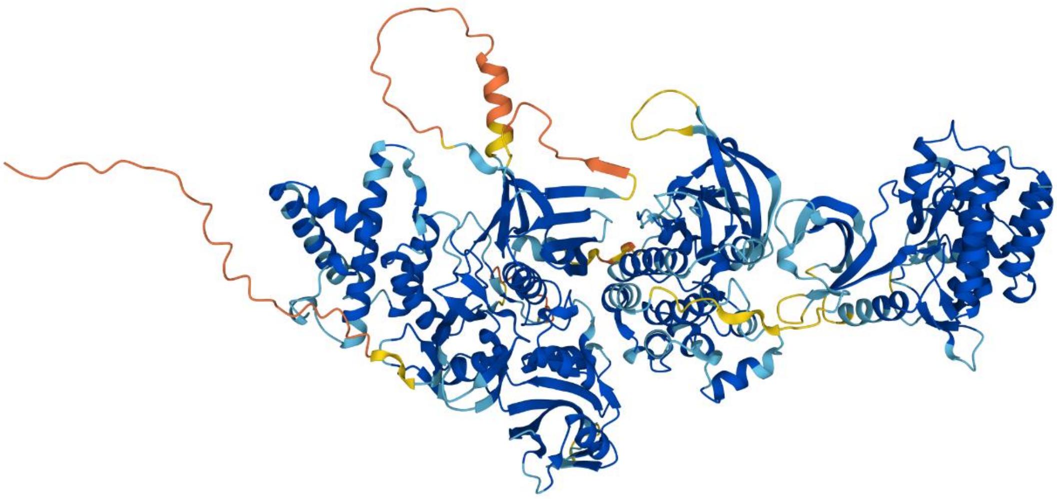

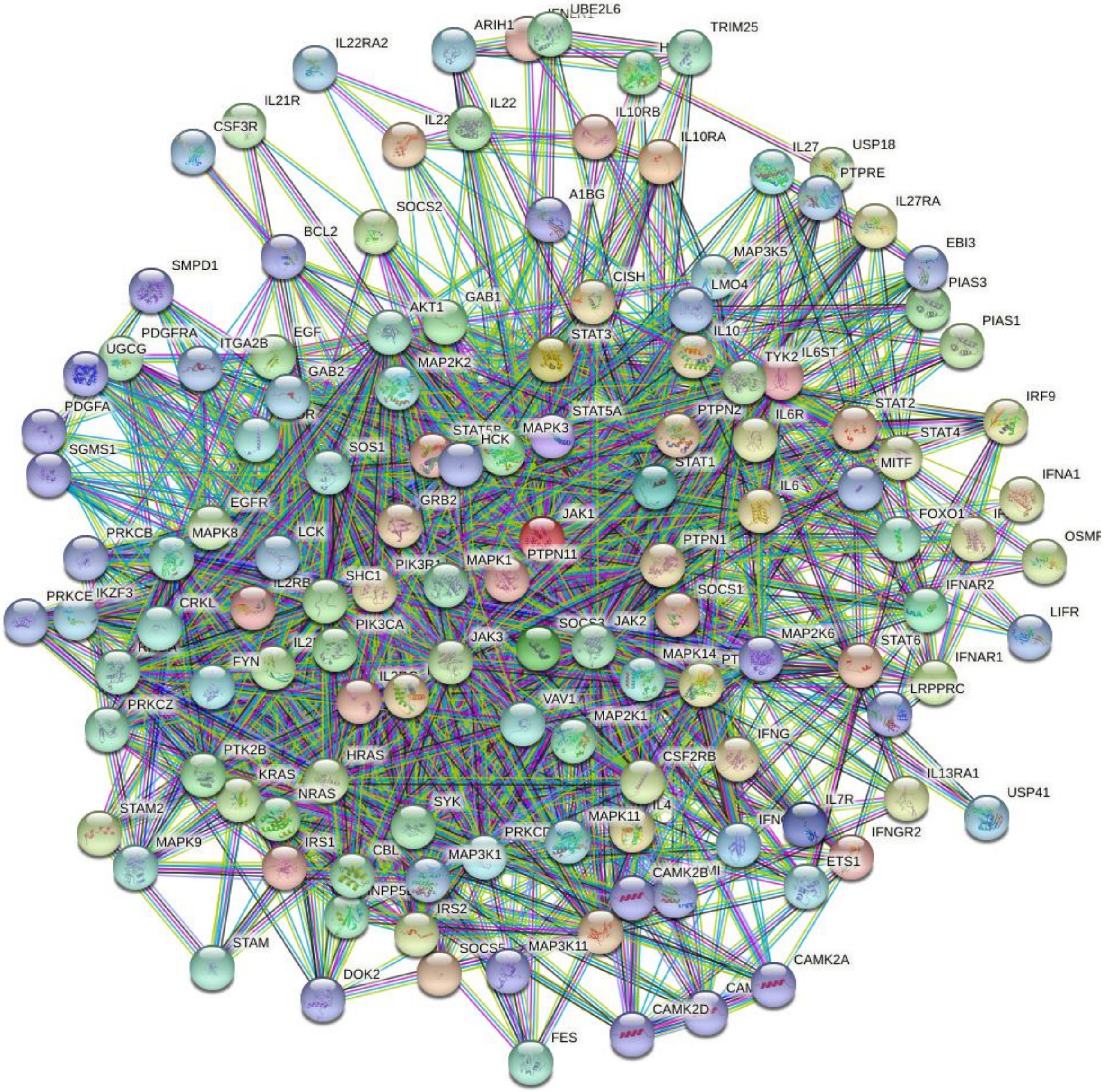

minimum required interaction score: highest confidence (0.900)
number of nodes: 128
number of edges: 1555
average node degree: 24.3
avg. local clustering coefficient: 0.668
expected number of edges: 333
PPI enrichment p-value: < 1.0e-16

IKKε (UniProt ID: Q14164)

>sp|Q14164|IKKE_HUMAN Inhibitor of nuclear factor kappa-B kinase subunit epsilon OS=Homo sapiens OX=9606 GN=IKBKE PE=1 SV=1

MQSTANYLWHTDDLLGQGATASVYKARNKKSGELVAVKVFNTTSYLRPREVQVREFE
VLRKLNHQNIVKLFAVEETGGSRQKVLVMEYCSSGSLLSVLESPENAFGLPEDEFLVVL
RCVVAGMNHLRENGIVHRDIKPGNIMRLVGEEGQSIYKLTDFGAARELDDDEKFVSVY
GTEEYLHPDMYERAVLRKPQQKAFGVTVDLWSIGVTLYHAATGSLPFIPFGGPRRNKEI
MYRITTEKPAGAIAGAQRRENGPLEWSYTLPITCQLSLGLQSQLVPILANILEVEQAKCW
GFDQFFAETSDILQRVVVHVFSLSQAVLHHIYIHAHNTIAIFQEAVHKQTSVAPRHQEYL
FEGHLCVLEPSVSAQHIAHTTASSPLTLFSTAIPKGLAFRDPALDVPKFVPKVDLQADYN
TAKGVLGAGYQALRLARALLDGQELMFRGLHWVMEVLQATCRRTLEVARTSLLYLSS
SLGTERFSSVAGTPEIQELKAAAELRSRLRTLAEVLSRCSQNITETQESLSSLNRELVKSR
DQVHEDRSIQQIQCCLDKMNFIYKQFKKSRMRPGLGYNEEQIHKLDKVNFSHLAKRLLQ
VFQEECVQKYQASLVTHGKRMRVVHETRNHLRLVGCSVAACNTEAQGVQESLSKLLE
ELSHQLLQDRAKGAQASPPPIAPYPSPTRKDLLLHMQELCEGMKLLASDLLDNNRIIERL
NRVPAPPDV

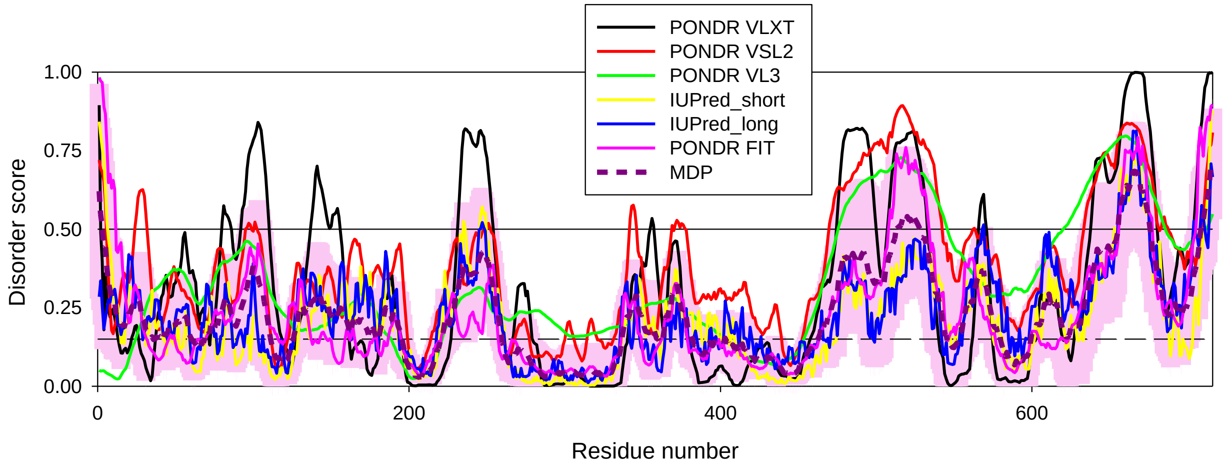

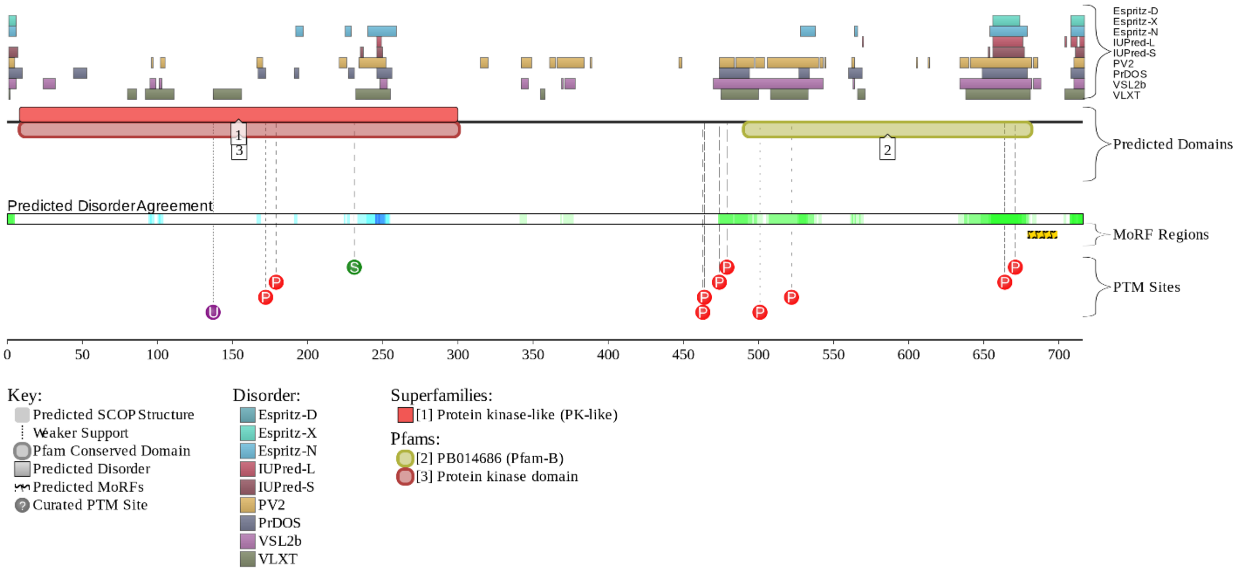

Espritz-D
Espritz-X
Espritz-N
IUPred-L
IUPred-S
PV2
PrDOS
VSL2b
VLXT
Predicted Domains
Predicted Disorder Agreement
MoRF Regions
PTM Sites
0
50
100
150
200
250
300
350
400
450
500
550
600
650
700
Key:
Predicted SCOP Structure
Weaker Support
Pfam Conserved Domain
Predicted Disorder
Predicted MoRFs
Curated PTM Site
Disorder:
Espritz-D
Espritz-X
Espritz-N
IUPred-L
IUPred-S
PV2
PrDOS
VSL2b
VLXT
Superfamilies:
[1] Protein kinase-like (PK-like)
Pfams:
[2] PB014686 (Pfam-B)
[3] Protein kinase domain

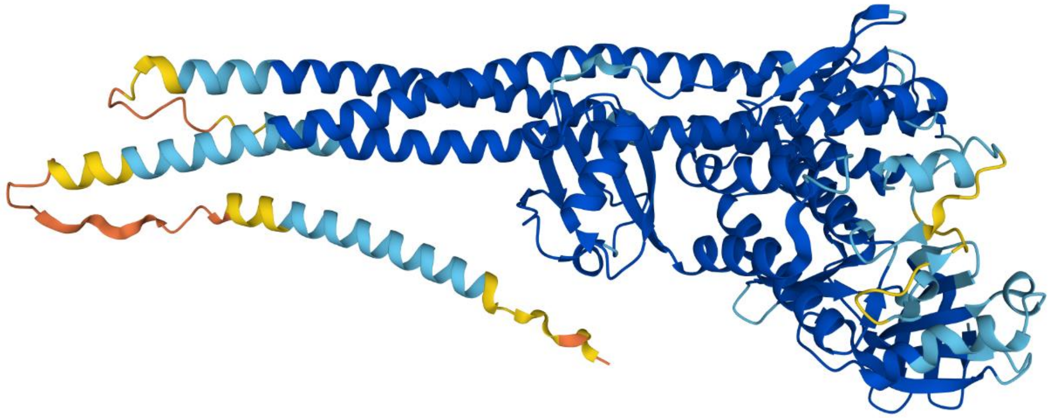

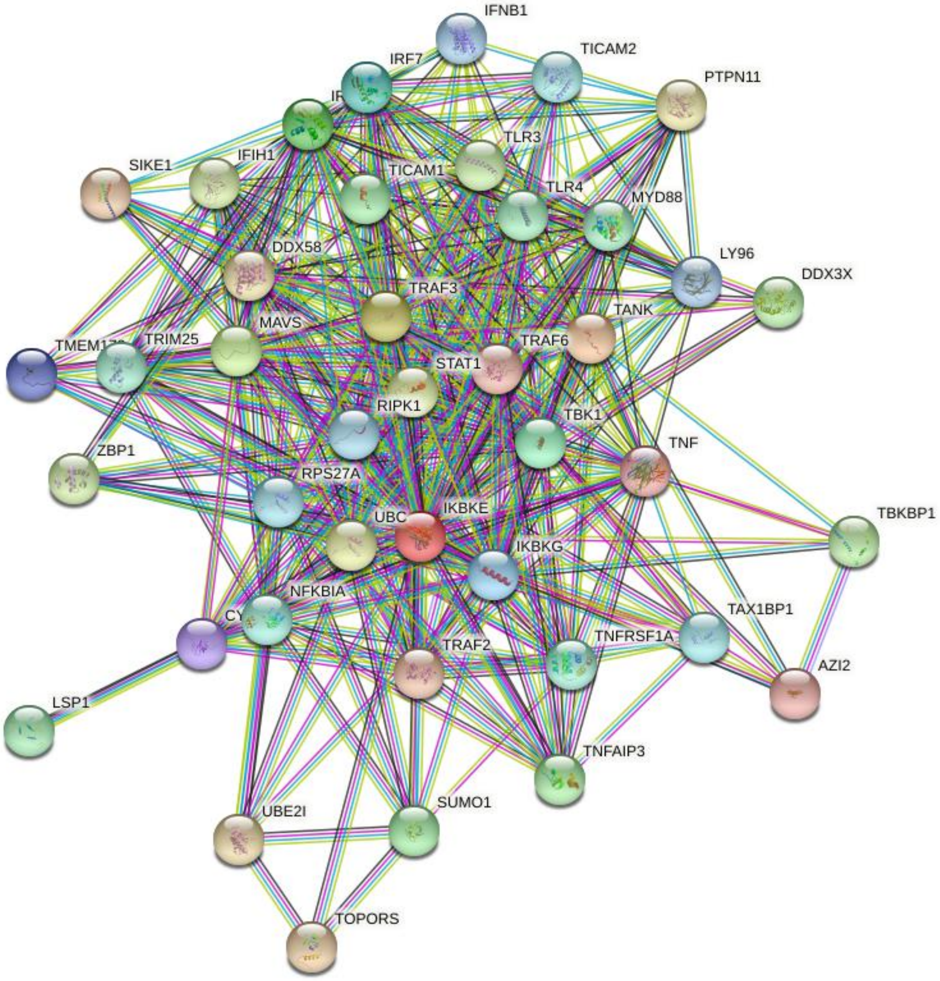

minimum required interaction score: high confidence (0.700)
number of nodes: 41
number of edges: 380
average node degree: 18.5
avg. local clustering coefficient: 0.769
expected number of edges: 70
PPI enrichment p-value: < 1.0e-16

BECN1 (UniProt ID: Q14457)

>sp|Q14457|BECN1_HUMAN Beclin-1 OS=Homo sapiens OX=9606 GN=BECN1 PE=1 SV=2

MEGSKTSNNSTMQVSFVCQRCSQPLKLDTSFKILDRVTIQELTAPLLTTAQAKPGETQEE
ETNSGEEPFIETPRQDGVSRRFIPPARMMSTESANSFTLIGEASDGGTMENLSRRLKVTGD
LFDIMSGQTDVDHPLCEECTDTLLDQLDTQLNVTENECQNYKRCLEILEQMNEDDSEQL
QMELKELALEEERLIQELEDVEKNRKIVAENLEKVQAEAERLDQEEAQYQREYSEFKRQ
QLELDDELKSVENQMRYAQTQLDKLKKTNVFNATFHIWHSGQFGTINNFRLGRLPSVPV
EWNEINAAWGQTVLLLHALANKMGLKFQRYRLVPYGNHSYLESLTDKSKELPLYCSGG
LRFFWDNKFDHAMVAFLDCVQQFKEEVEKGETRFCLPYRMDVEKGKIEDTGGSGGSYS
IKTQFNSEEQWTKALKFMLTNLKWGLAWVSSQFYNK

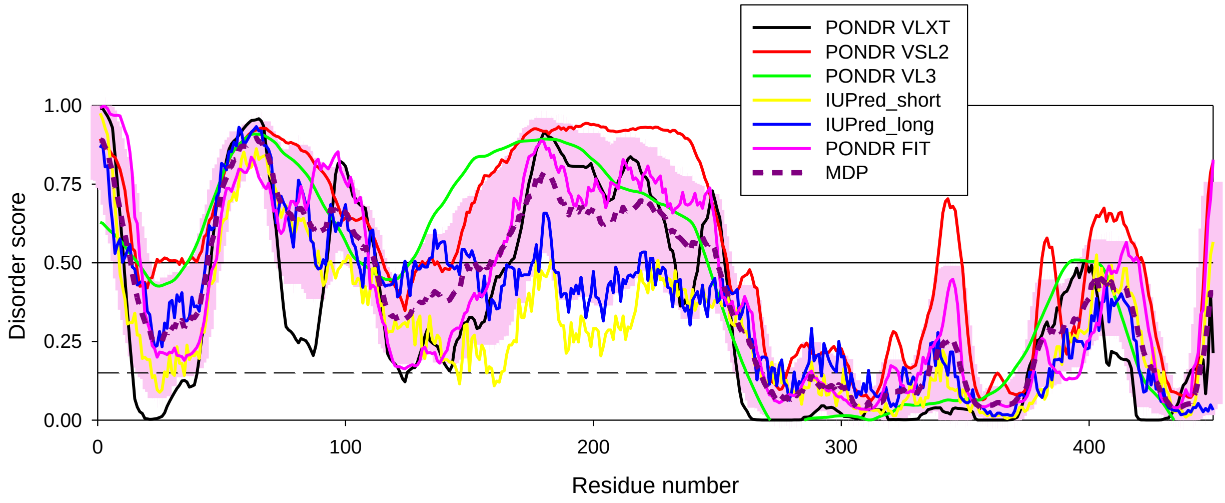

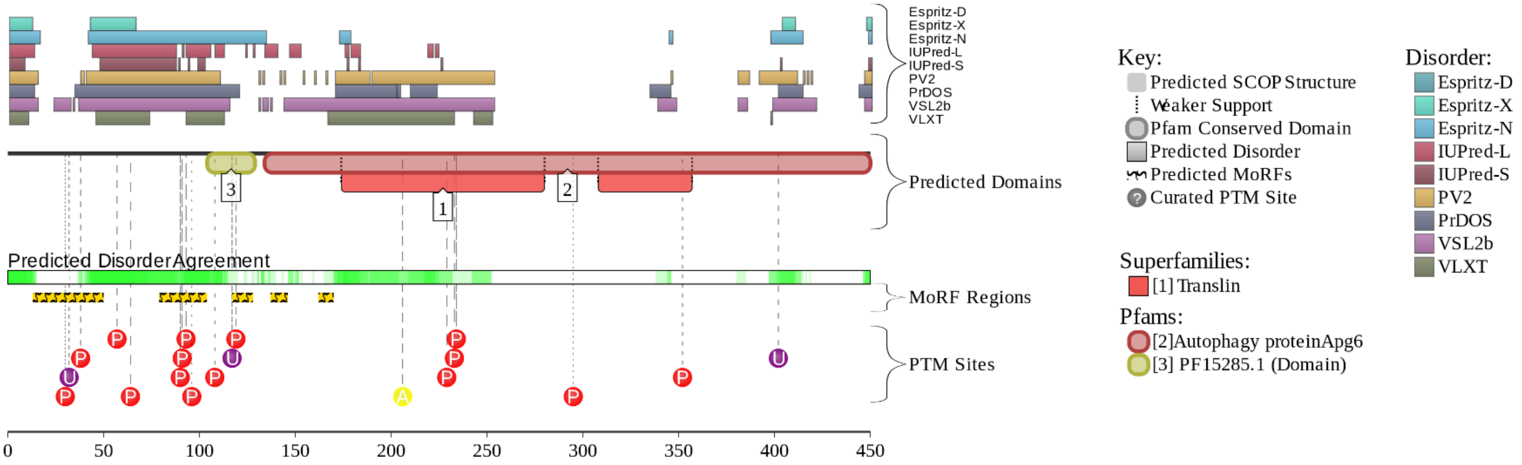

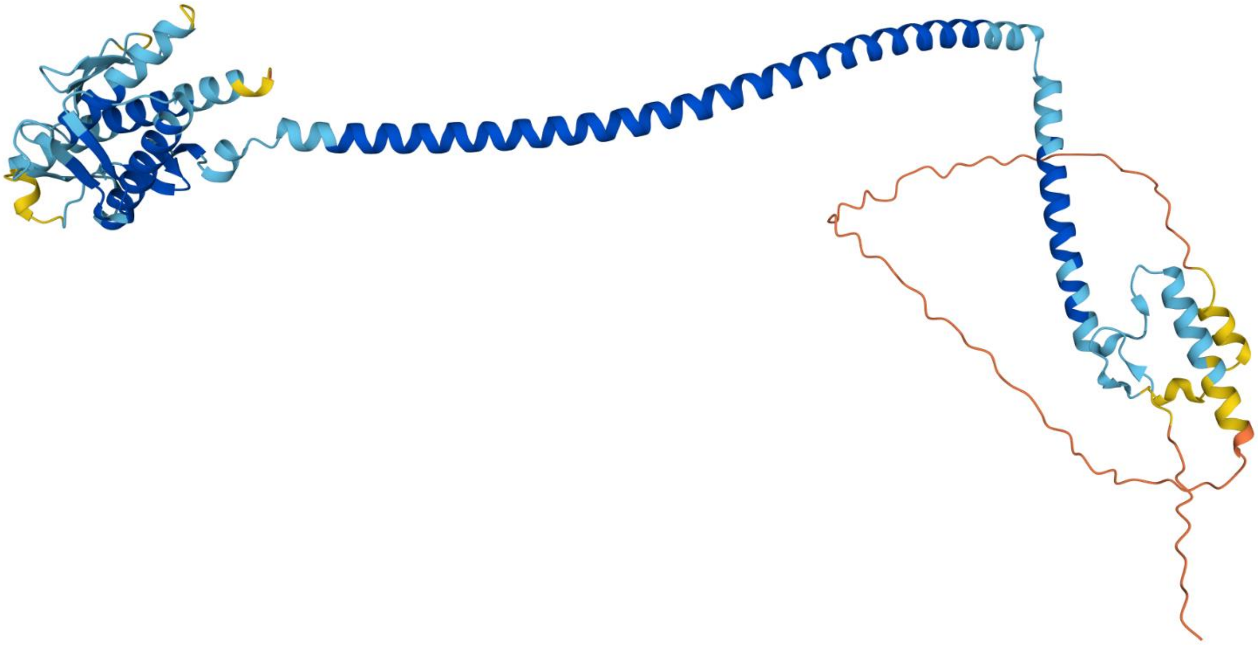

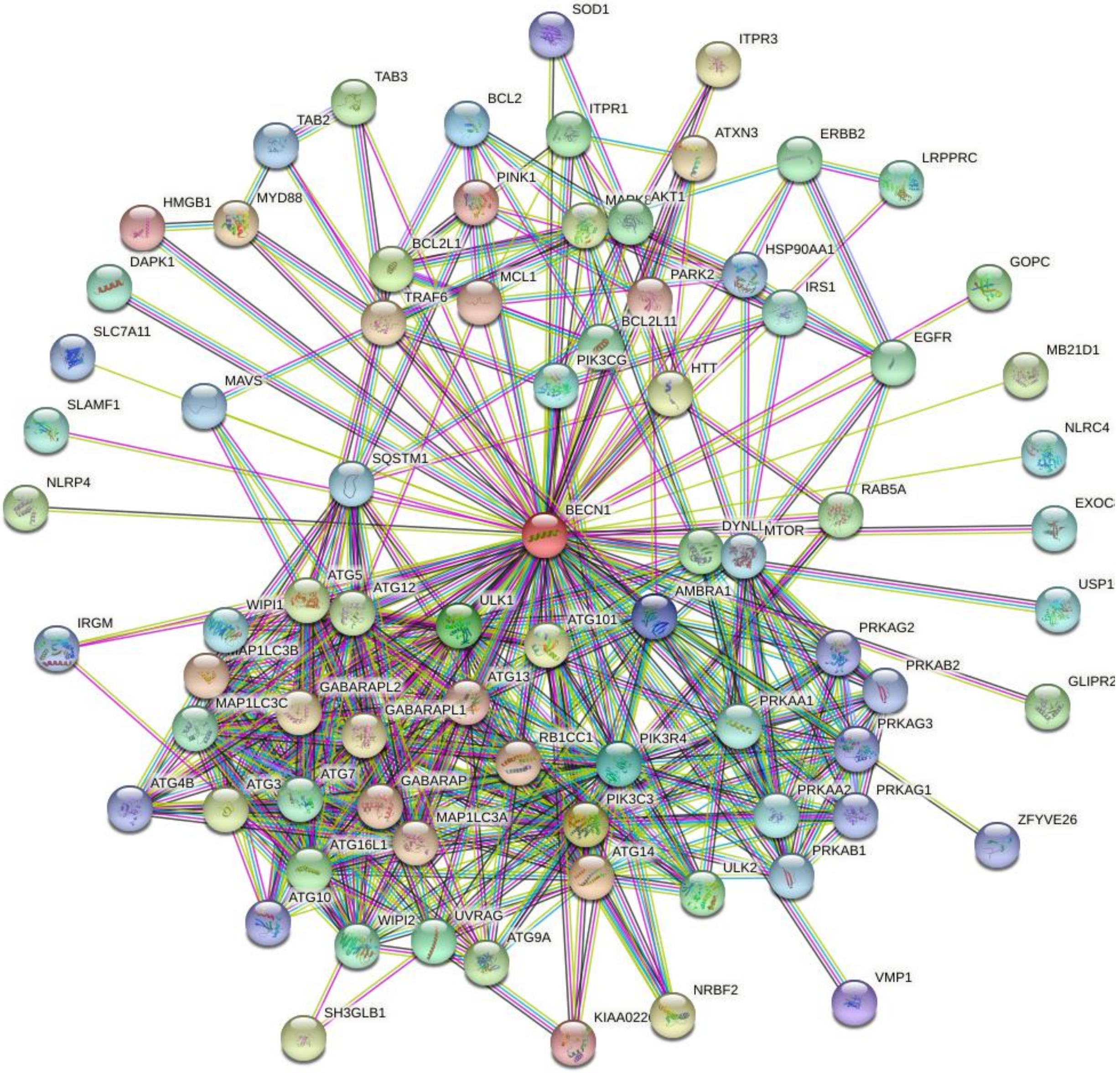

minimum required interaction score: highest confidence (0.900)
number of nodes: 79
number of edges: 461
average node degree: 11.7
avg. local clustering coefficient: 0.754
expected number of edges: 104
PPI enrichment p-value: < 1.0e-16

TUB-α (UniProt ID: Q71U36)

>sp|Q71U36|TBA1A_HUMAN Tubulin alpha-1A chain OS=Homo sapiens OX=9606 GN=TUBA1A PE=1 SV=1

MRECISIHVGQAGVQIGNACWELYCLEHGIQPDGQMPSDKTIGGGDDSFNTFFSETGAG
KHVPRAVFVDLEPTVIDEVRTGTYRQLFHPEQLITGKEDAANNYARGHYTIGKEIIDLVL
DRIRKLADQCTGLQGFLVFHSFGGGTGSGFTSLLMERLSVDYGKKSKLEFSIYPAPQVST
AVVEPYNSILTTHTTLEHSDCAFMVDNEAIYDICRRNLDIERPTYTNLNRLIGQIVSSITAS
LRFDGALNVDLTEFQTNLVPYPRIHFPLATYAPVISAEKAYHEQLSVAEITNACFEPANQ
MVKCDPRHGKYMACCLLYRGDVVPKDVNAAIATIKTKRTIQFVDWCPTGFKVGINYQP
PTVVPGGDLAKVQRAVCMLSNTTAIAEAWARLDHKFDLMYAKRAFVHWYVGEGMEE
GEFSEAREDMAALEKDYEEVGVDSVEGEGEEEGEEY

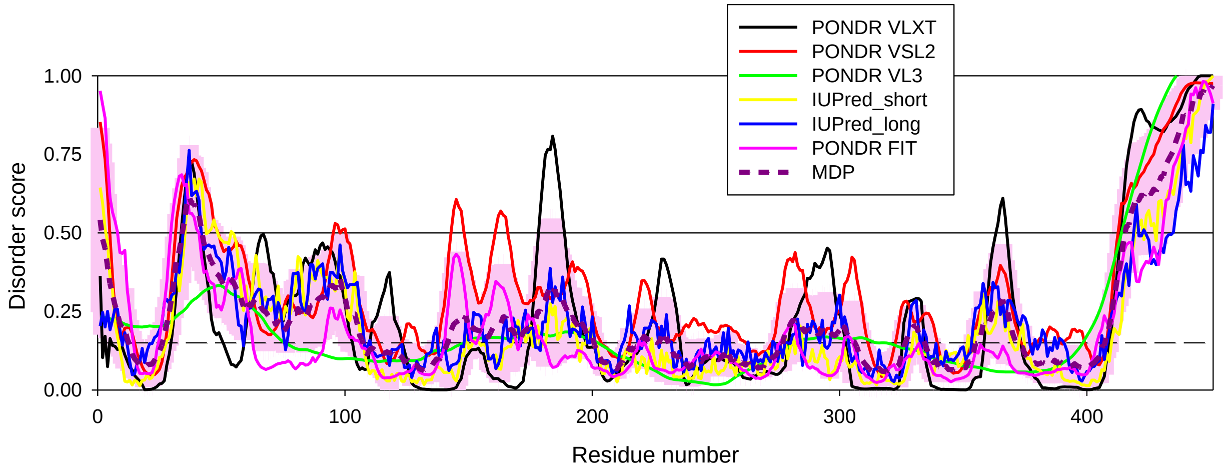

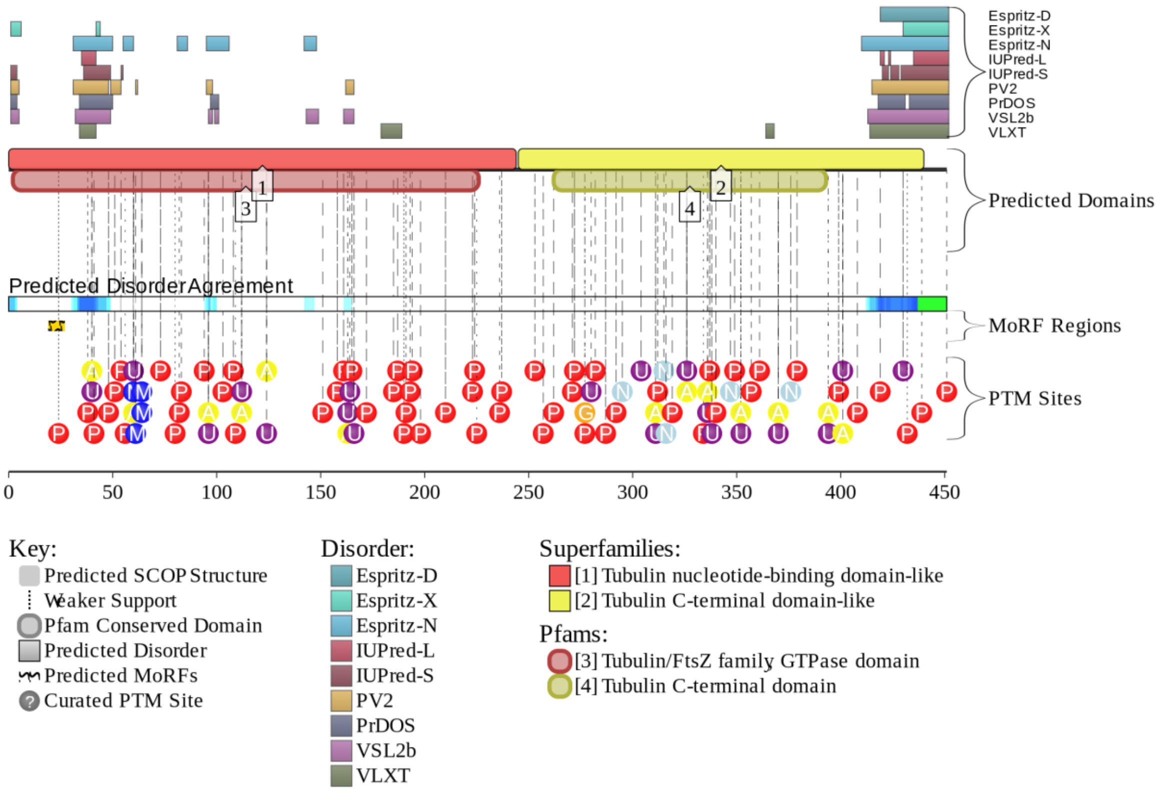
Espritz-D
Espritz-X
Espritz-N
IUPred-L
IUPred-S
PV2
PrDOS
VSL2b
VLXT
Predicted Domains
Predicted Disorder Agreement
MoRF Regions
PTM Sites
0
50
100
150
200
250
300
350
400
450
Key:
Predicted SCOP Structure
Weaker Support
Pfam Conserved Domain
Predicted Disorder
Predicted MoRFs
Curated PTM Site
Disorder:
Espritz-D
Espritz-X
Espritz-N
IUPred-L
IUPred-S
PV2
PrDOS
VSL2b
VLXT
Superfamilies:
[1] Tubulin nucleotide-binding domain-like
[2] Tubulin C-terminal domain-like
Pfams:
[3] Tubulin/FtsZ family GTPase domain
[4] Tubulin C-terminal domain

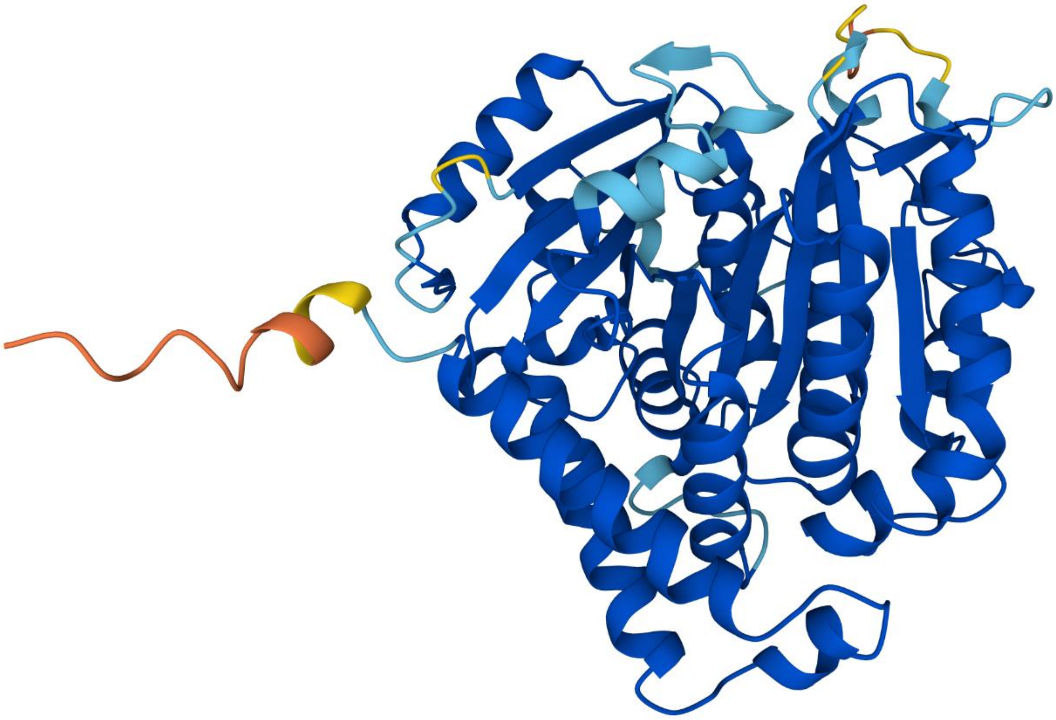

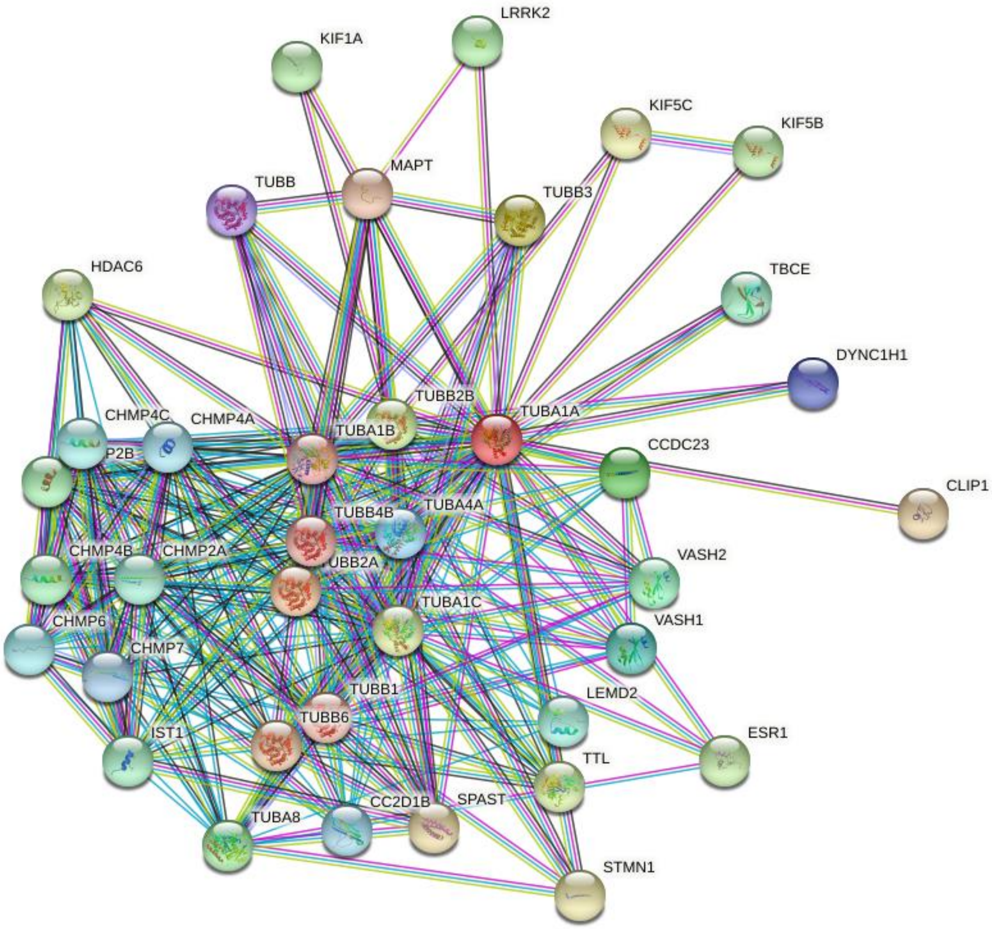

minimum required interaction score: highest confidence (0.900)
number of nodes: 38
number of edges: 254
average node degree: 13.4
avg. local clustering coefficient: 0.755
expected number of edges: 40
PPI enrichment p-value: < 1.0e-16

TUB-β (UniProt ID: Q9H4B7)

>sp|Q9H4B7|TBB1_HUMAN Tubulin beta-1 chain OS=Homo sapiens OX=9606 GN=TUBB1 PE=1 SV=1

MREIVHIQIGQCGNQIGAKFWEMIGEEHGIDLAGSDRGASALQLERISVYYNEAYGRKY
VPRAVLVDLEPGTMDSIRSSKLGALFQPDSFVHGNSGAGNNWAKGHYTEGAELIENVL
EVVRHESESCDCLQGFQIVHSLGGGTGSGMGTLLMNKIREEYPDRIMNSFSVMPSPKVS
DTVVEPYNAVLSIHQLIENADACFCIDNEALYDICFRTLKLTTPTYGDLNHLVSLTMSGIT
TSLRFPGQLNADLRKLAVNMVPFPRLHFFMPGFAPLTAQGSQQYRALSVAELTQQMFD
ARNTMAACDLRRGRYLTVACIFRGKMSTKEVDQQLLSVQTRNSSCFVEWIPNNVKVAV
CDIPPRGLSMAATFIGNNTAIQEIFNRVSEHFSAMFKRKAFVHWYTSEGMDINEFGEAEN
NIHDLVSEYQQFQDAKAVLEEDEEVTEEAEMEPEDKGH

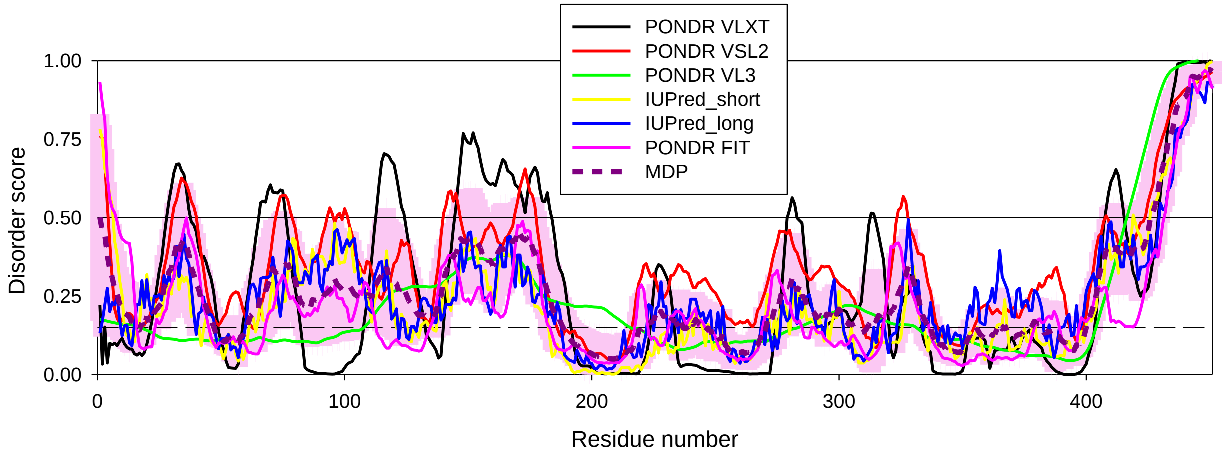

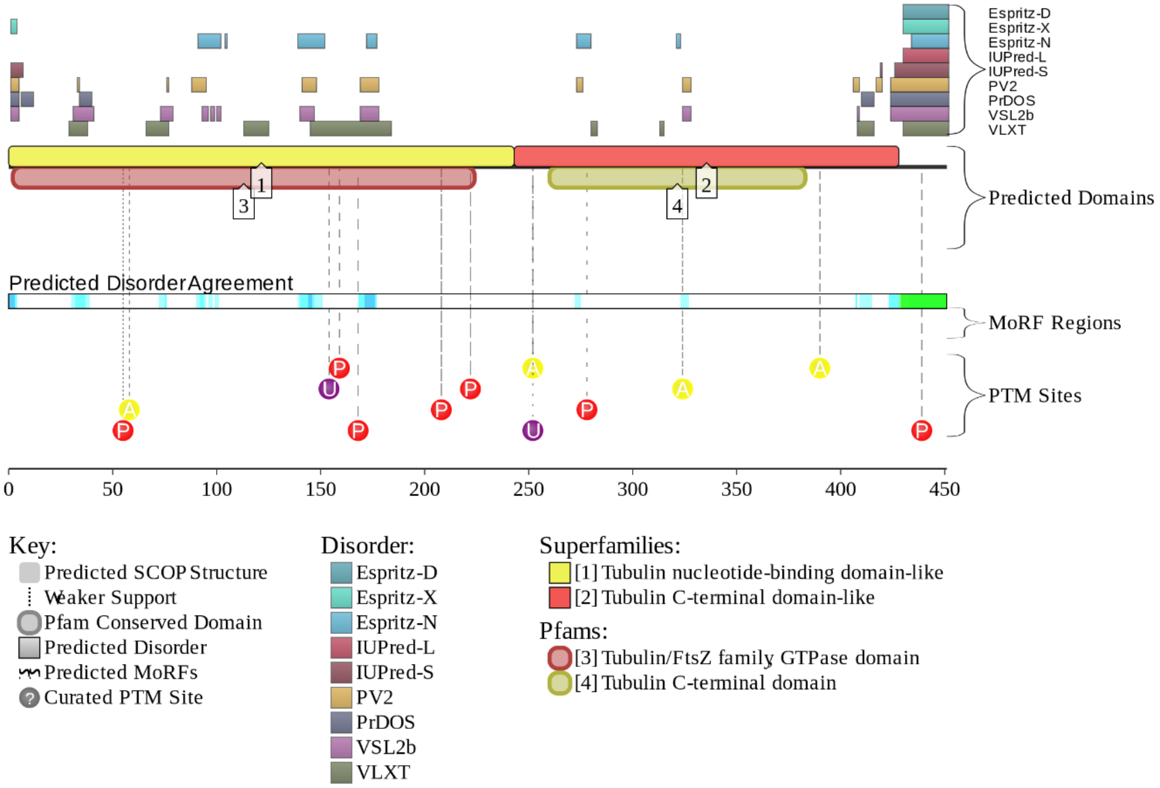
Espritz-D
Espritz-X
Espritz-N
IUPred-L
IUPred-S
PV2
PrDOS
VSL2b
VLXT
Predicted Domains
Predicted Disorder Agreement
MoRF Regions
PTM Sites
0
50
100
150
200
250
300
350
400
450
Key:
Predicted SCOP Structure
Weaker Support
Pfam Conserved Domain
Predicted Disorder
Predicted MoRFs
Curated PTM Site
Disorder:
Espritz-D
Espritz-X
Espritz-N
IUPred-L
IUPred-S
PV2
PrDOS
VSL2b
VLXT
Superfamilies:
[1] Tubulin nucleotide-binding domain-like
[2] Tubulin C-terminal domain-like
Pfams:
[3] Tubulin/FtsZ family GTPase domain
[4] Tubulin C-terminal domain

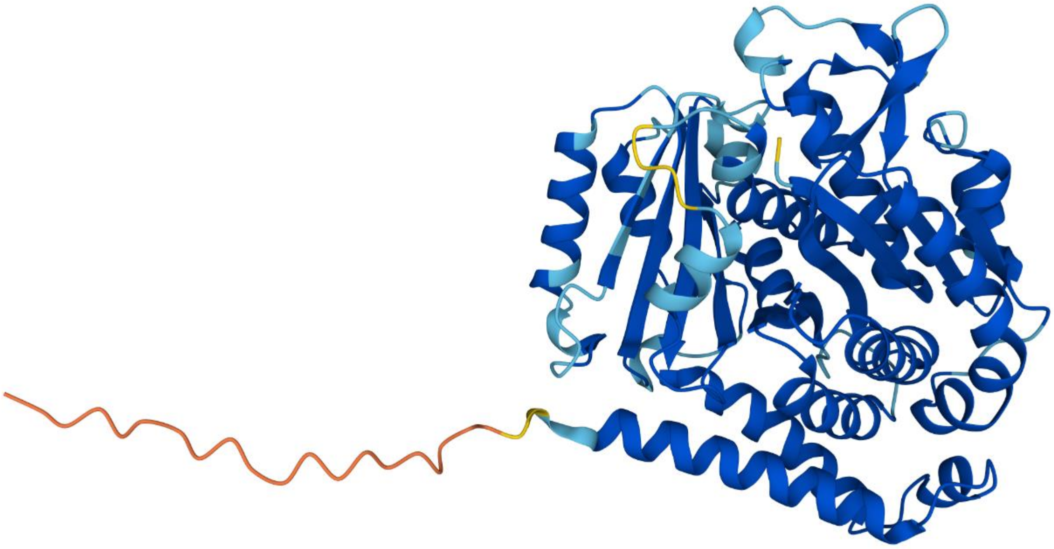

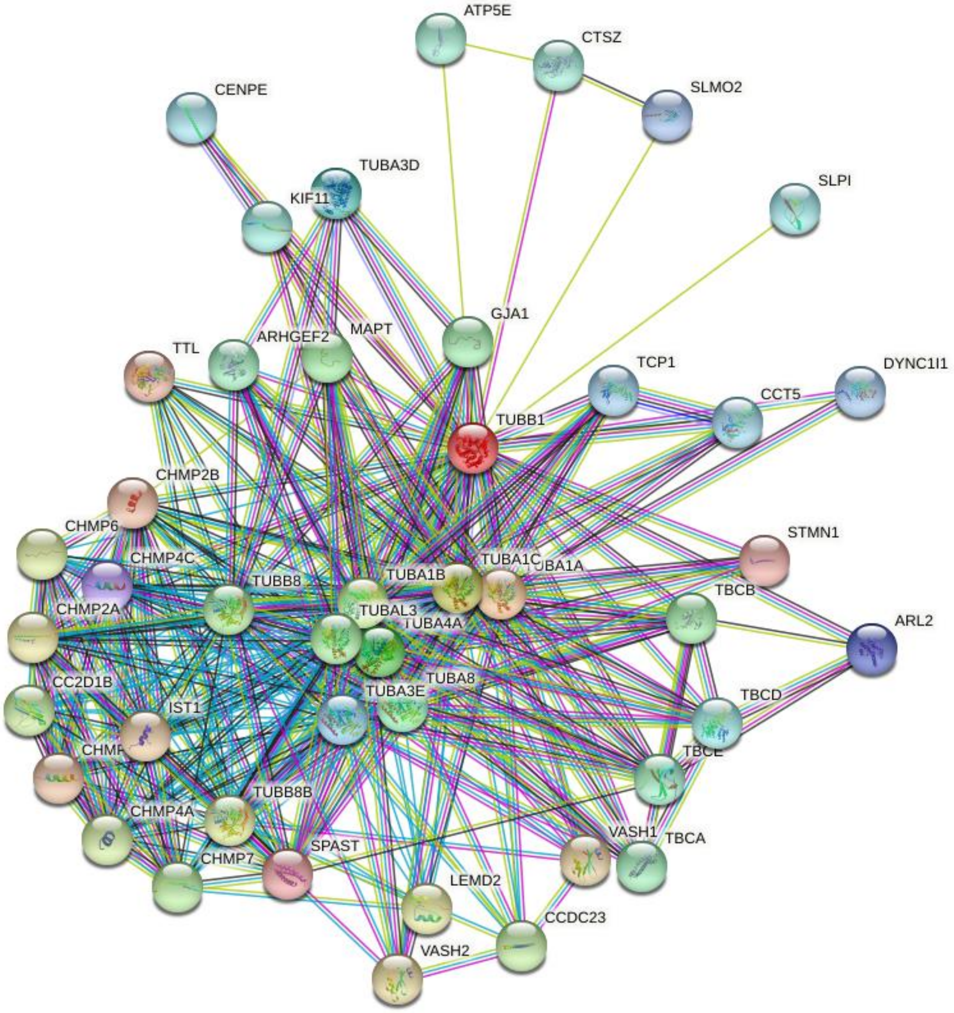

minimum required interaction score: high confidence (0.700)
number of nodes: 44
number of edges: 325
average node degree: 14.8
avg. local clustering coefficient: 0.757
expected number of edges: 49
PPI enrichment p-value: < 1.0e-16

ABCE1 (UniProt ID: P61221)

>sp|P61221|ABCE1_HUMAN ATP-binding cassette sub-family E member 1 OS=Homo sapiens OX=9606 GN=ABCE1 PE=1 SV=1

MADKLTRIAIVNHDKCKPKKCRQECKKSCPVVRMGKLCIEVTPQSKIAWISETLCIGCGI
CIKKCPFGALSIVNLPSNLEKETTHRYCANAFKLHRLPIPRPGEVLGLVGTNGIGKSTALK
ILAGKQKPNLGKYDDPPDWQEILTYFRGSELQNYFTKILEDDLKAIIKPQYVDQIPKAAK
GTVGSILDRKDETKTQAIVCQQLDLTHLKERNVEDLSGGELQRFACAVVCIQKADIFMF
DEPSSYLDVKQRLKAAITIRSLINPDRYIIVVEHDLSVLDYLSDFICCLYGVPSAYGVVTM
PFSVREGINIFLDGYVPTENLRFRDASLVFKVAETANEEEVKKMCMYKYPGMKKKMGE
FELAIVAGEFTDSEIMVMLGENGTGKTTFIRMLAGRLKPDEGGEVPVLNVSYKPQKISPK
STGSVRQLLHEKIRDAYTHPQFVTDVMKPLQIENIIDQEVQTLSGGELQRVALALCLGKP
ADVYLIDEPSAYLDSEQRLMAARVVKRFILHAKKTAFVVEHDFIMATYLADRVIVFDGV
PSKNTVANSPQTLLAGMNKFLSQLEITFRRDPNNYRPRINKLNSIKDVEQKKSGNYFFLD
D

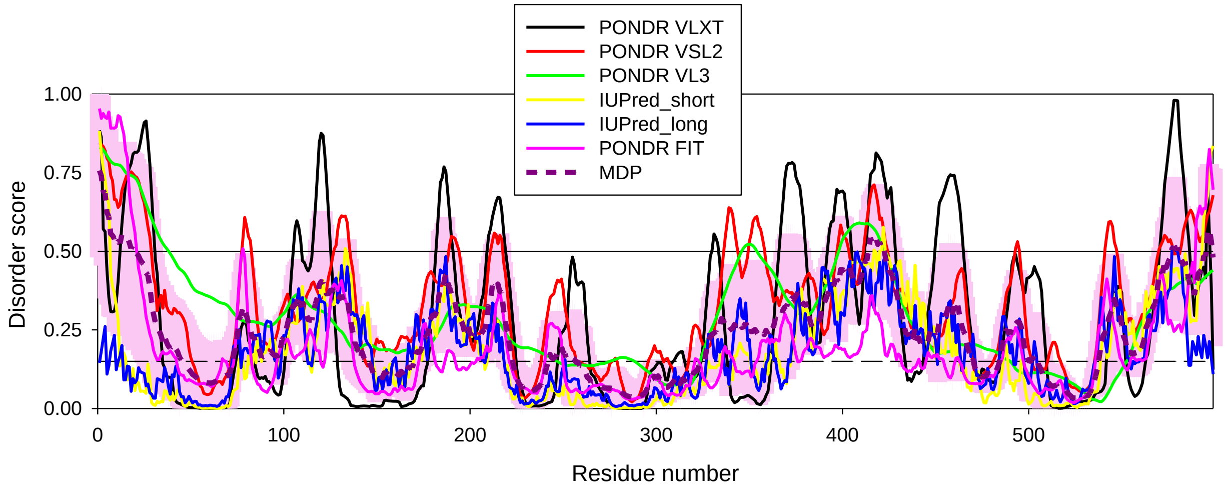

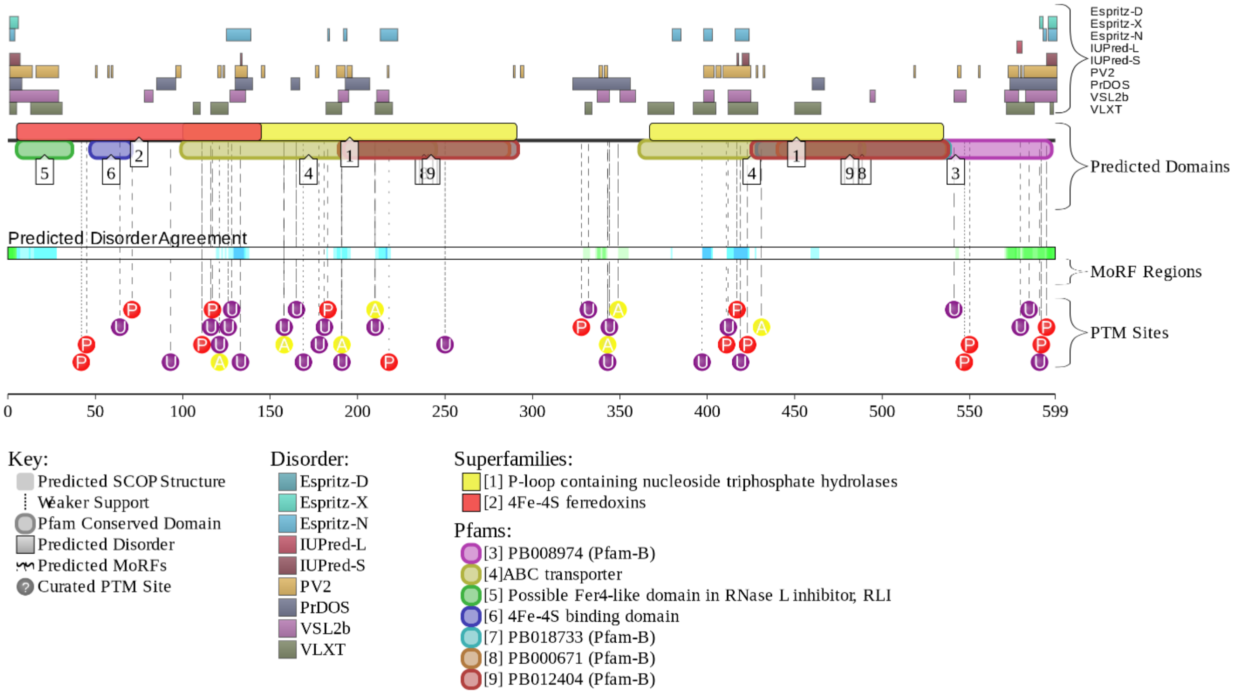
Espritz-D
Espritz-X
Espritz-N
IUPred-L
IUPred-S
PV2
PrDOS
VSL2b
VLXT
Predicted Domains
Predicted Disorder Agreement
MoRF Regions
PTM Sites
0 50 100 150 200 250 300 350 400 450 500 550 599
Key:
Predicted SCOP Structure
Weaker Support
Pfam Conserved Domain
Predicted Disorder
Predicted MoRFs
Curated PTM Site
Disorder:
Espritz-D
Espritz-X
Espritz-N
IUPred-L
IUPred-S
PV2
PrDOS
VSL2b
VLXT
Superfamilies:
[1] P-loop containing nucleoside triphosphate hydrolases
[2] 4Fe-4S ferredoxins
Pfams:
[3] PB008974 (Pfam-B)
[4]ABC transporter
[5] Possible Fer4-like domain in RNase L inhibitor, RLI
[6] 4Fe-4S binding domain
[7] PB018733 (Pfam-B)
[8] PB000671 (Pfam-B)
[9] PB012404 (Pfam-B)

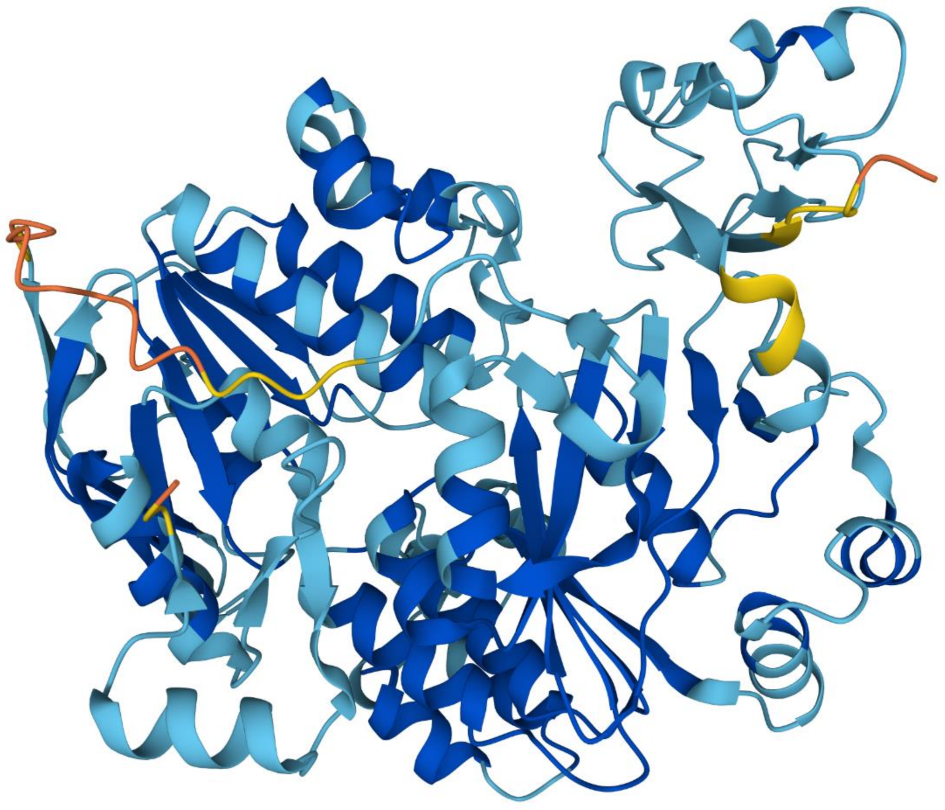

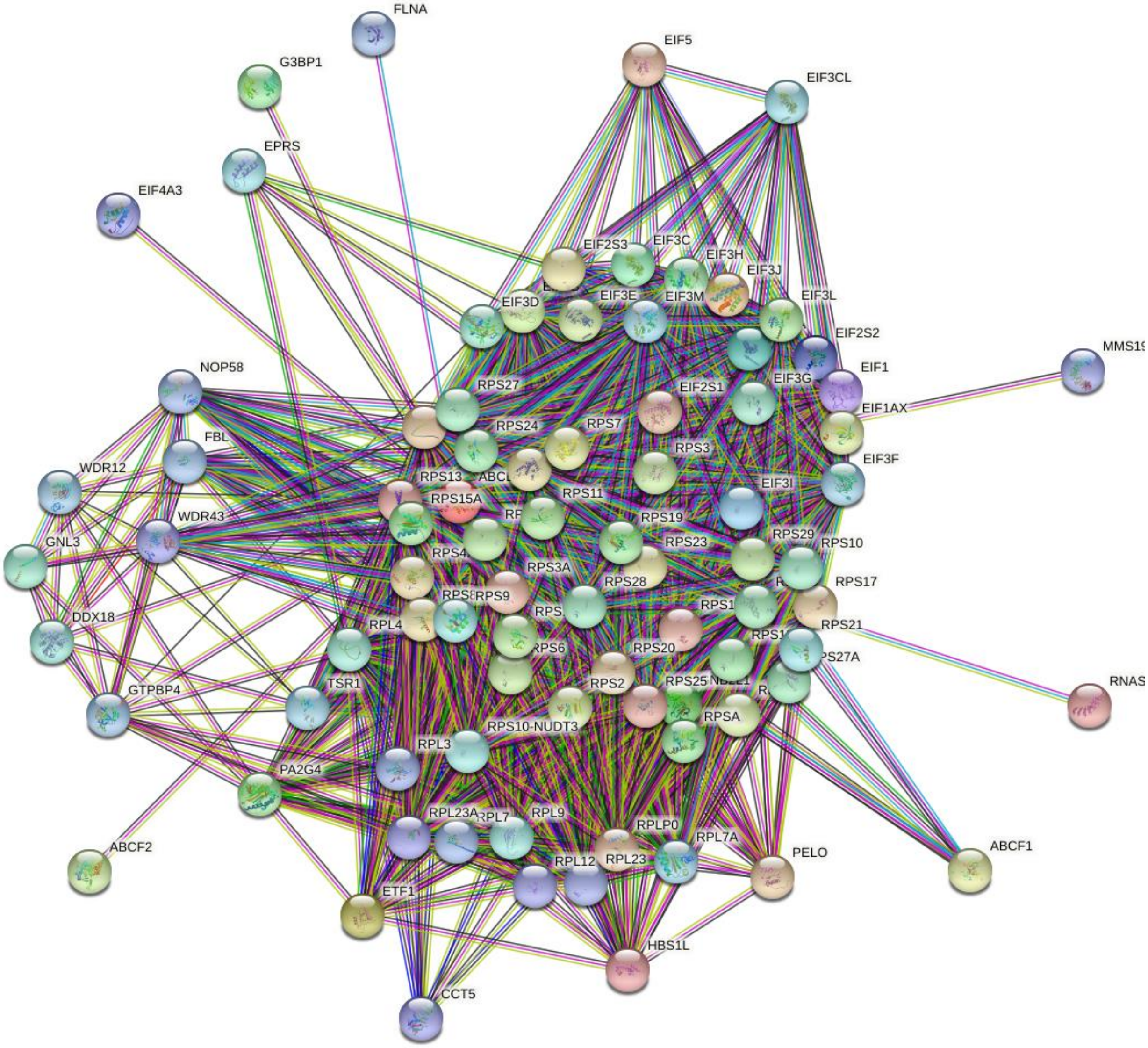

minimum required interaction score: highest confidence (0.900)
number of nodes: 84
number of edges: 1985
average node degree: 47.3
avg. local clustering coefficient: 0.881
expected number of edges: 289
PPI enrichment p-value: < 1.0e-16

CCTγ (UniProt ID: P49368)

>sp|P49368|TCPG_HUMAN T-complex protein 1 subunit gamma OS=Homo sapiens OX=9606 GN=CCT3 PE=1 SV=4

MMGHRPVLVLSQNTKRESGRKVQSGNINAAKTIADIIRTCLGPKSMMKMLLDPMGGIV
MTNDGNAILREIQVQHPAAKSMIEISRTQDEEVGDGTTSVIILAGEMLSVAEHFLEQQMH
PTVVISAYRKALDDMISTLKKISIPVDISDSDMMLNIINSSITTKAISRWSSLACNIALDAV
KMVQFEENGRKEIDIKKYARVEKIPGGIIEDSCVLRGVMINKDVTHPRMRRYIKNPRIVL
LDSSLEYKKGESQTDIEITREEDFTRILQMEEEYIQQLCEDIIQLKPDVVITEKGISDLAQH
YLMRANITAIRRVRKTDNNRIARACGARIVSRPEELREDDVGTGAGLLEIKKIGDEYFTFI
TDCKDPKACTILLRGASKEILSEVERNLQDAMQVCRNVLLDPQLVPGGGASEMAVAHA
LTEKSKAMTGVEQWPYRAVAQALEVIPRTLIQNCGASTIRLLTSLRAKHTQENCETWGV
NGETGTLVDMKELGIWEPLAVKLQTYKTAVETAVLLLRIDDIVSGHKKKGDDQSRQGG
APDAGQE

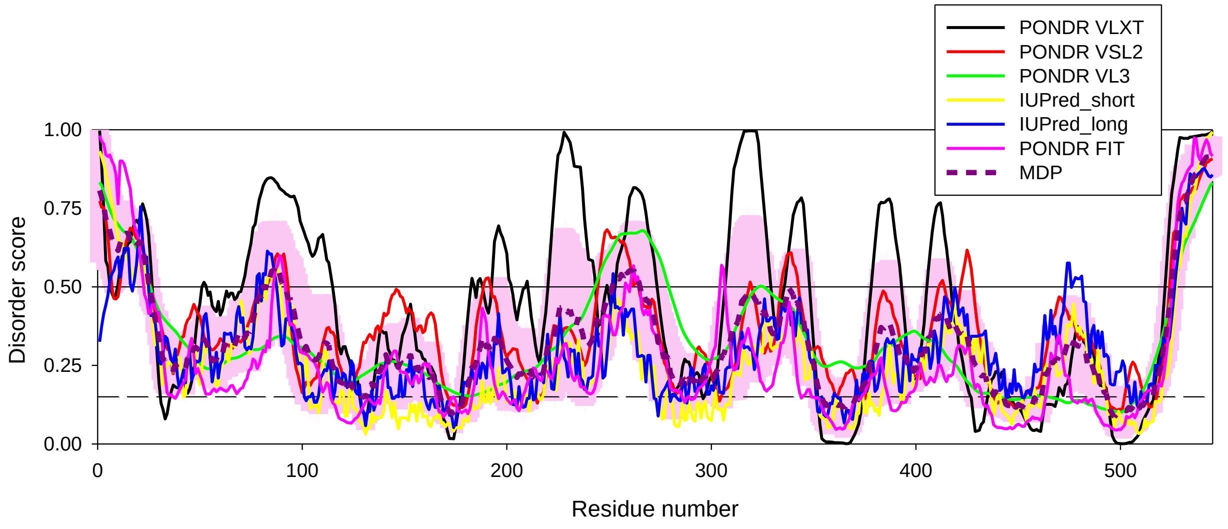

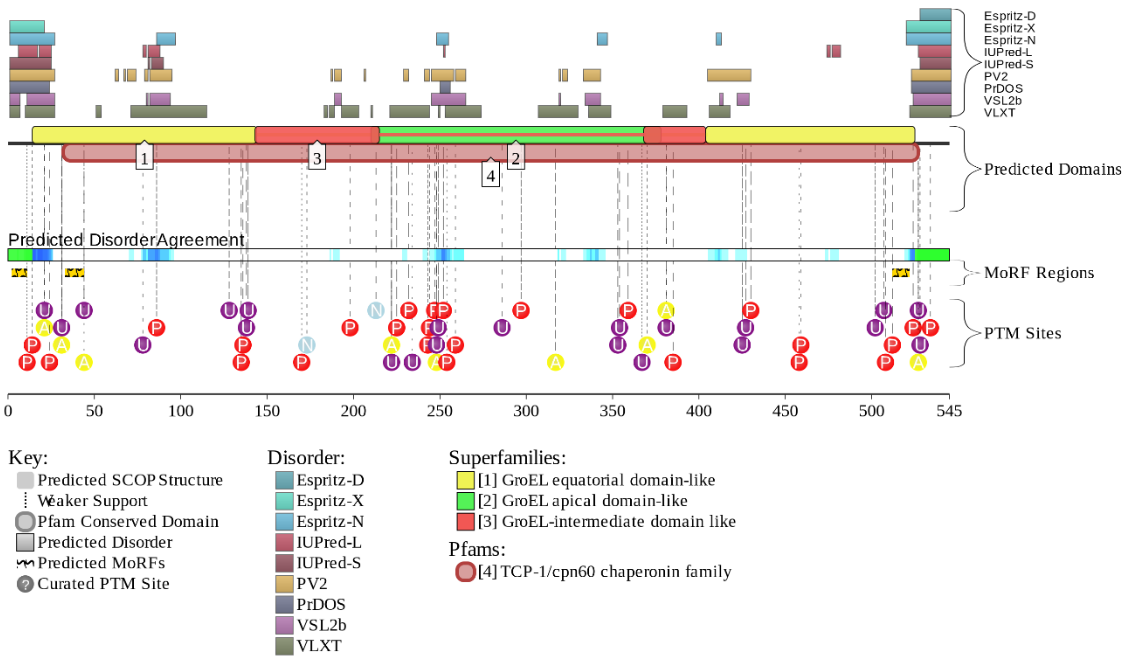
Espritz-D
Espritz-X
Espritz-N
IUPred-L
IUPred-S
PV2
PrDOS
VSL2b
VLXT
Predicted Domains
Predicted Disorder Agreement
MoRF Regions
PTM Sites
0
50
100
150
200
250
300
350
400
450
500
545
Key:
Predicted SCOP Structure
Weaker Support
Pfam Conserved Domain
Predicted Disorder
Predicted MoRFs
Curated PTM Site
Disorder:
Espritz-D
Espritz-X
Espritz-N
IUPred-L
IUPred-S
PV2
PrDOS
VSL2b
VLXT
Superfamilies:
[1] GroEL equatorial domain-like
[2] GroEL apical domain-like
[3] GroEL-intermediate domain like
Pfams:
[4] TCP-1/cpn60 chaperonin family

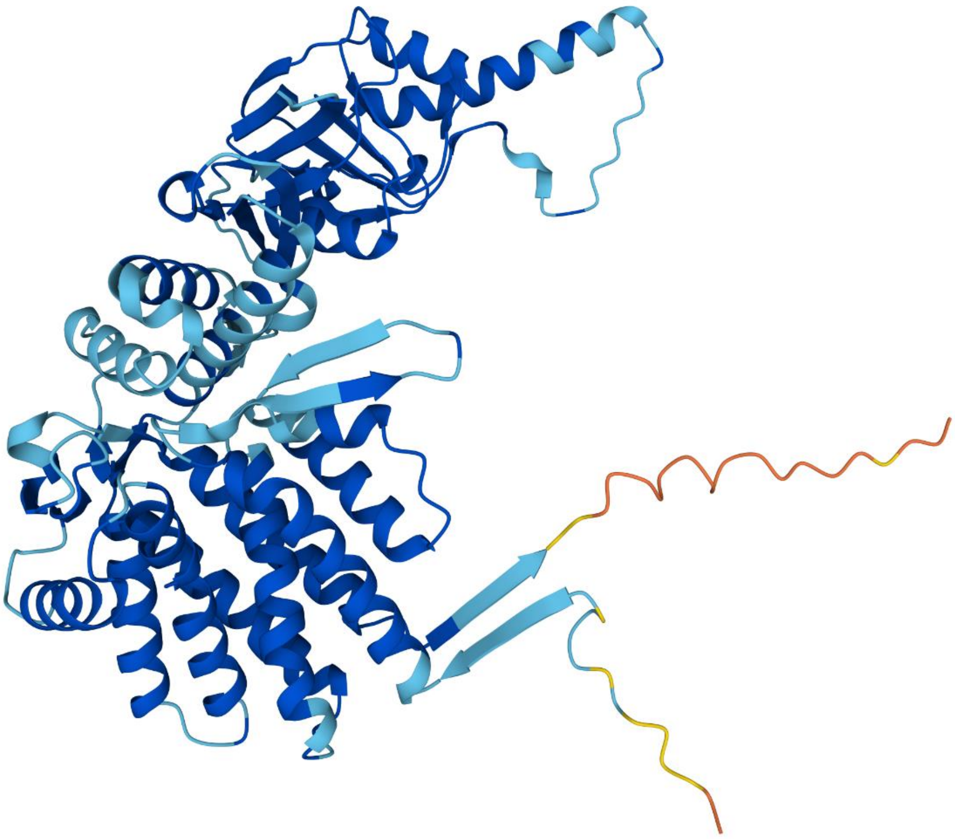

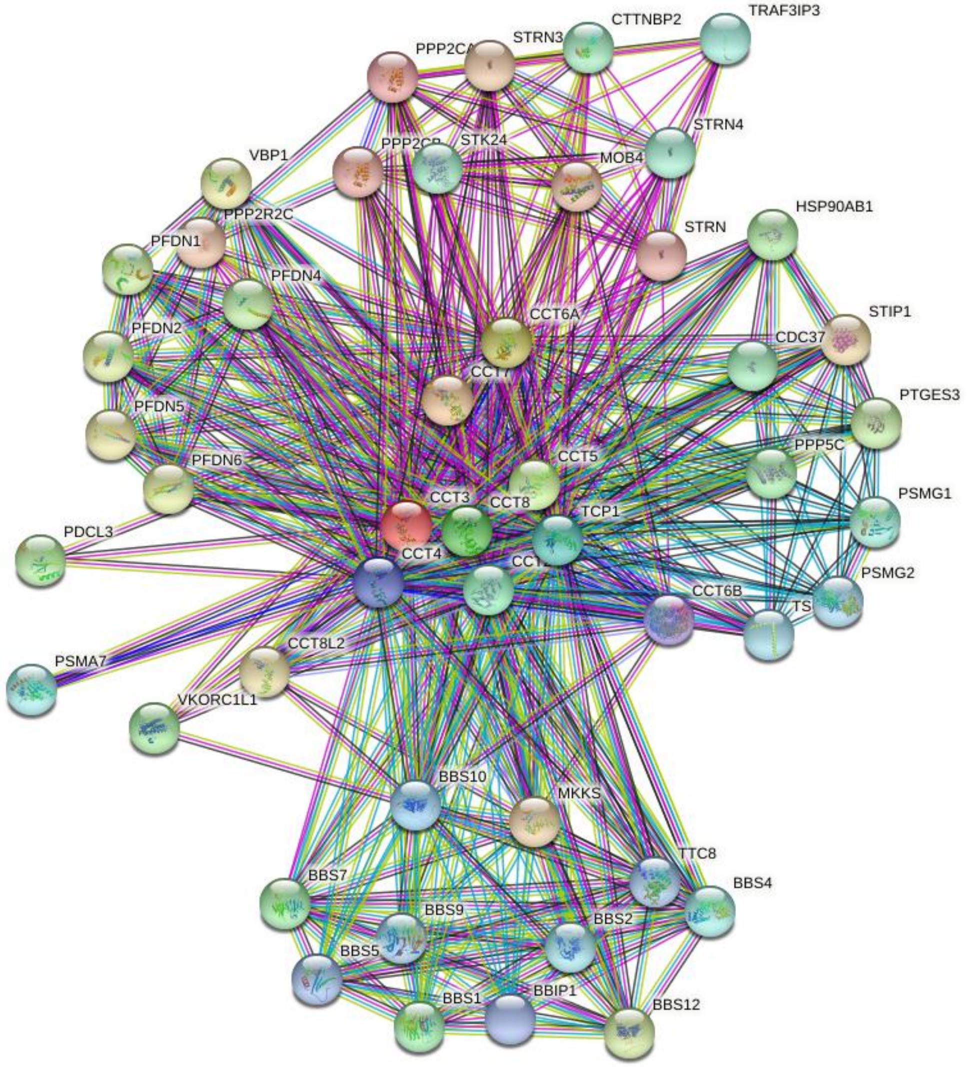

minimum required interaction score: highest confidence (0.900)
number of nodes: 48
number of edges: 443
average node degree: 18.5
avg. local clustering coefficient: 0.872
expected number of edges: 53
PPI enrichment p-value: < 1.0e-16

CDC37 (UniProt ID: Q16543)

>sp|Q16543|CDC37_HUMAN Hsp90 co-chaperone Cdc37 OS=Homo sapiens OX=9606
GN=CDC37 PE=1 SV=1

MVDYSVWDHIEVSDDEDETHPNIDTASLFRWRHQARVERMEQFQKEKEELDRGCRECK
RKVAECQRKLKELEVAEGGKAELERLQAEAQQLRKEERSWEQKLEEMRKKEKSMPWN
VDTLSKDGFSKSMVNTKPEKTEEDSEEVREQKHKTFVEKYEKQIKHFGMLRRWDDSQK
YLSDNVHLVCEETANYLVIWCIDLEVEEKCALMEQVAHQTIVMQFILELAKSLKVDPRA
CFRQFFTKIKTADRQYMEGFNDELEAFKERVRGRAKLRIEKAMKEYEEEERKKRLGPG
GLDPVEVYESLPEELQKCFDVKDVQMLQDAISKMDPTDAKYHMQRCIDSGLWVPNSK
ASEAKEGEEAGPGDPLLEAVPKTGDEKDVSV

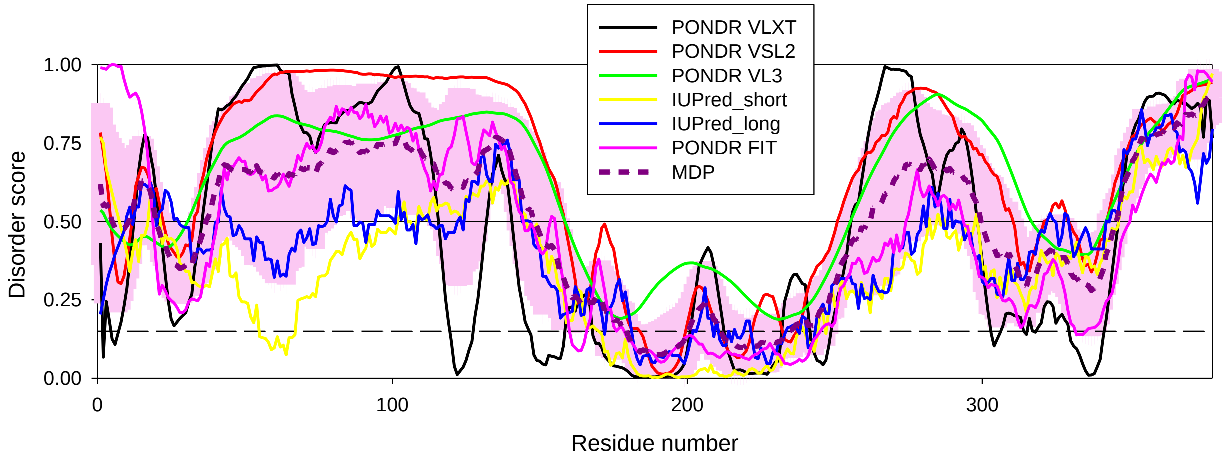

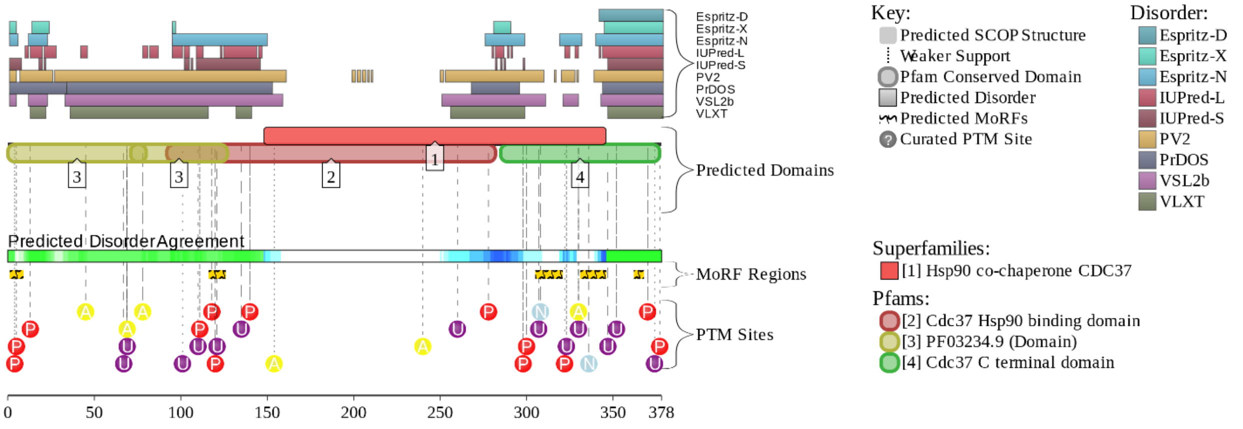

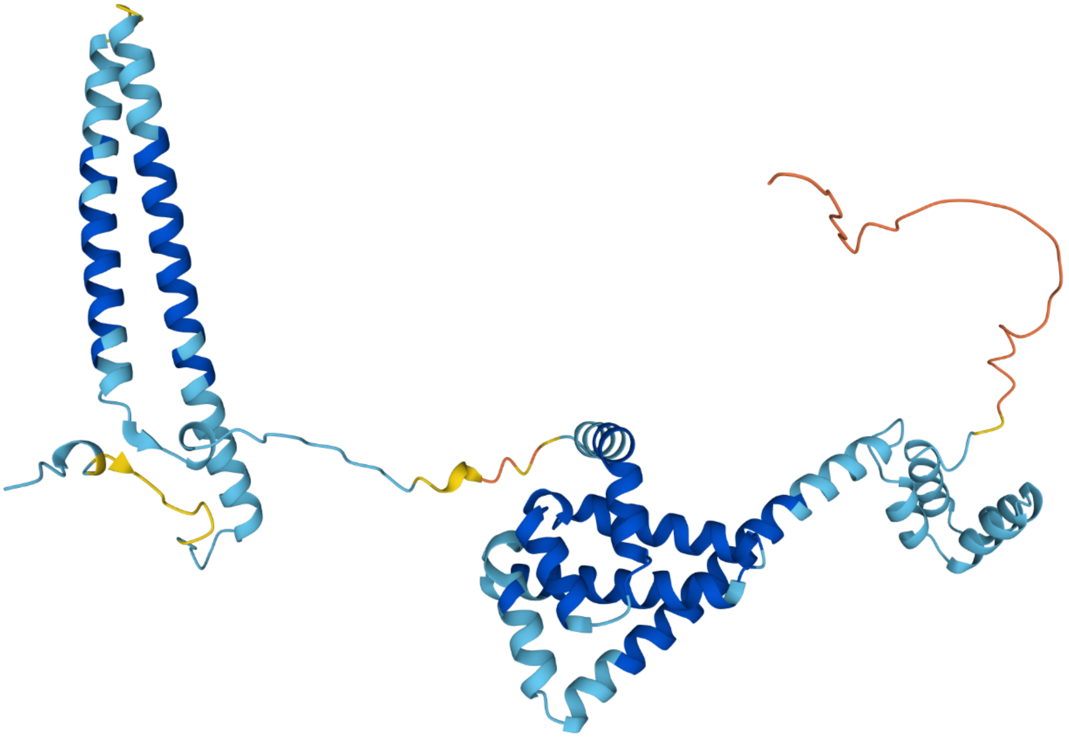

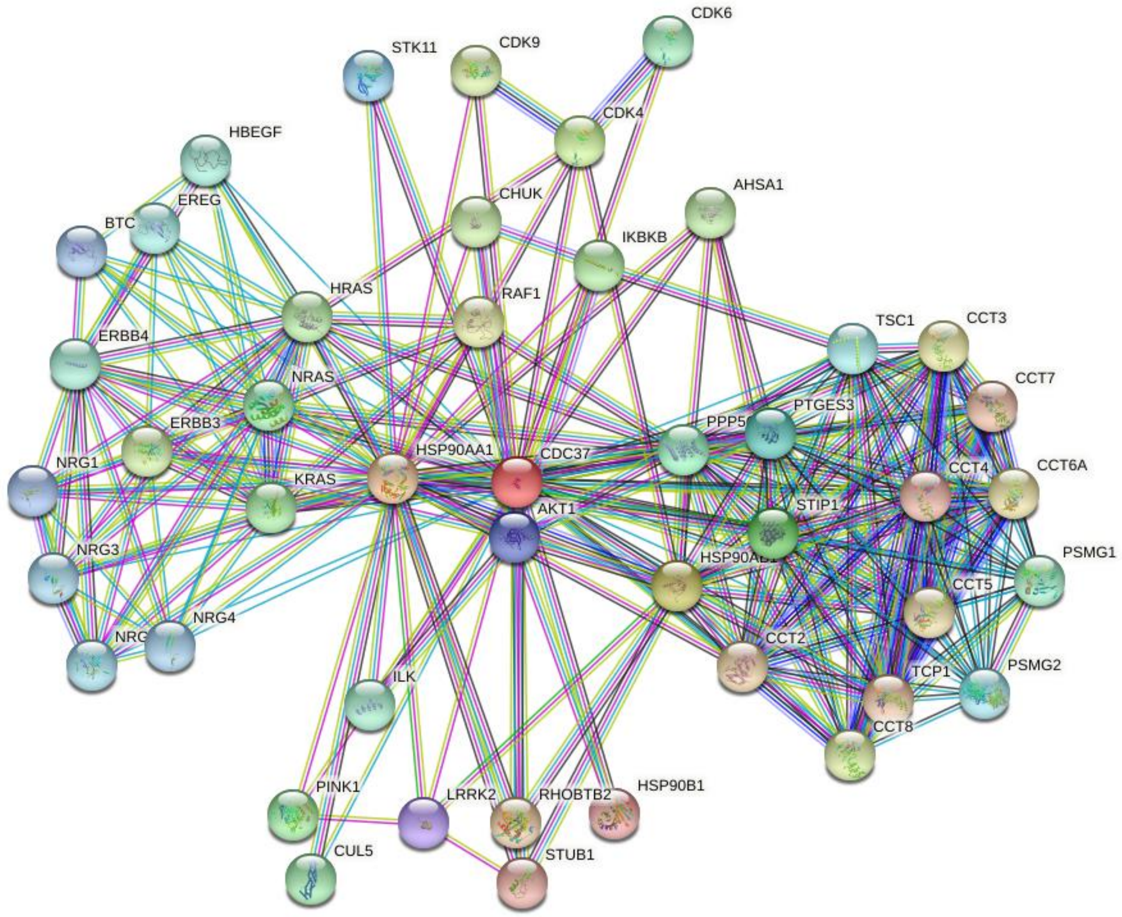

minimum required interaction score: highest confidence (0.900)
number of nodes: 45
number of edges: 257
average node degree: 11.4
avg. local clustering coefficient: 0.856
expected number of edges: 65
PPI enrichment p-value: < 1.0e-16

Hsp90AA1 (UniProt ID: P07900)

>sp|P07900|HS90A_HUMAN Heat shock protein HSP 90-alpha OS=Homo sapiens OX=9606 GN=HSP90AA1 PE=1 SV=5

MPEETQTQDQPMEEEEVETFAFQAEIAQLMSLIINTFYSNKEIFLRELISNSSDALDKIRYE
SLTDPSKLDSGKELHINLIPNKQDRTLTIVDTGIGMTKADLINNLGTIAKSGTKAFMEALQ
AGADISMIGQFGVGFYSAYLVAEKVTVITKHNDDEQYAWESSAGGSFTVRTDTGEPMG
RGTKVILHLKEDQTEYLEERRIKEIVKKHSQFIGYPITLFVEKERDKEVSDDEAEEKEDKE
EEKEKEEKESEDKPEIEDVGSDEEEEKKDGDKKKKKKIKEKYIDQEELNKTKPIWTRNPD
DITNEEYGEFYKSLTNDWEDHLAVKHFSVEGQLEFRALLFVPRRAPFDLFENRKKKNNI
KLYVRRVFIMDNCEELIPEYLNFIRGVVDSEDLPLNISREMLQQSKILKVIRKNLVKKCLE
LFTELAEDKENYKKFYEQFSKNIKLGIHEDSQNRKKLSELLRYYTSASGDEMVSLKDYC
TRMKENQKHIYYITGETKDQVANSAFVERLRKHGLEVIYMIEPIDEYCVQQLKEFEGKT
LVSVTKEGLELPEDEEEKKKQEEKKTKFENLCKIMKDILEKKVEKVVVSNRLVTSPCCIV
TSTYGWTANMERIMKAQALRDNSTMGYMAAKKHLEINPDHSIIETLRQKAEADKNDKS
VKDLVILLYETALLSSGFSLEDPQTHANRIYRMIKLGLGIDEDDPTADDTSAAVTEEMPP
LEGDDDTSRMEEVD

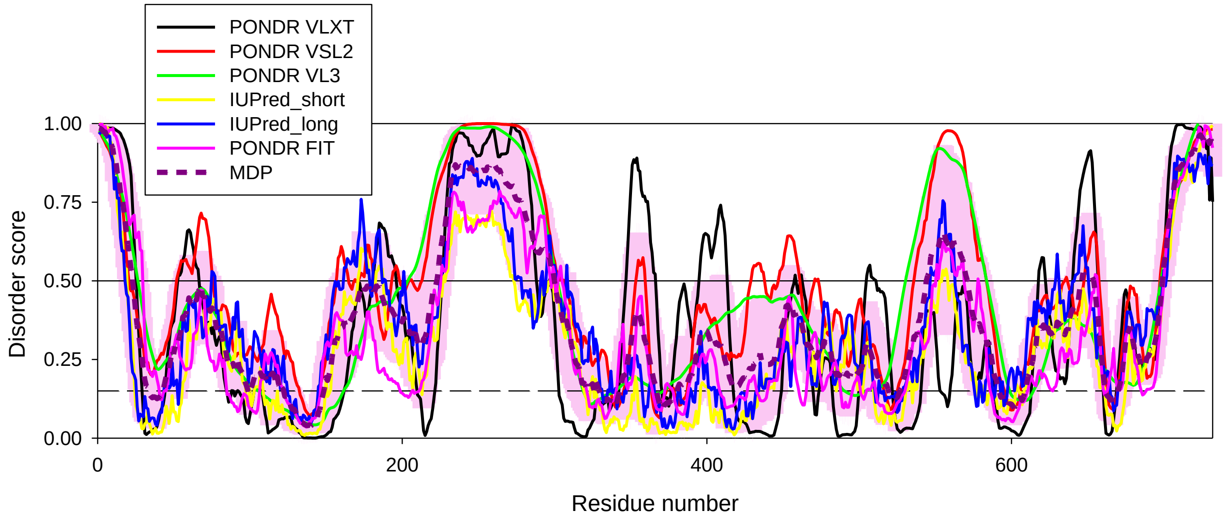

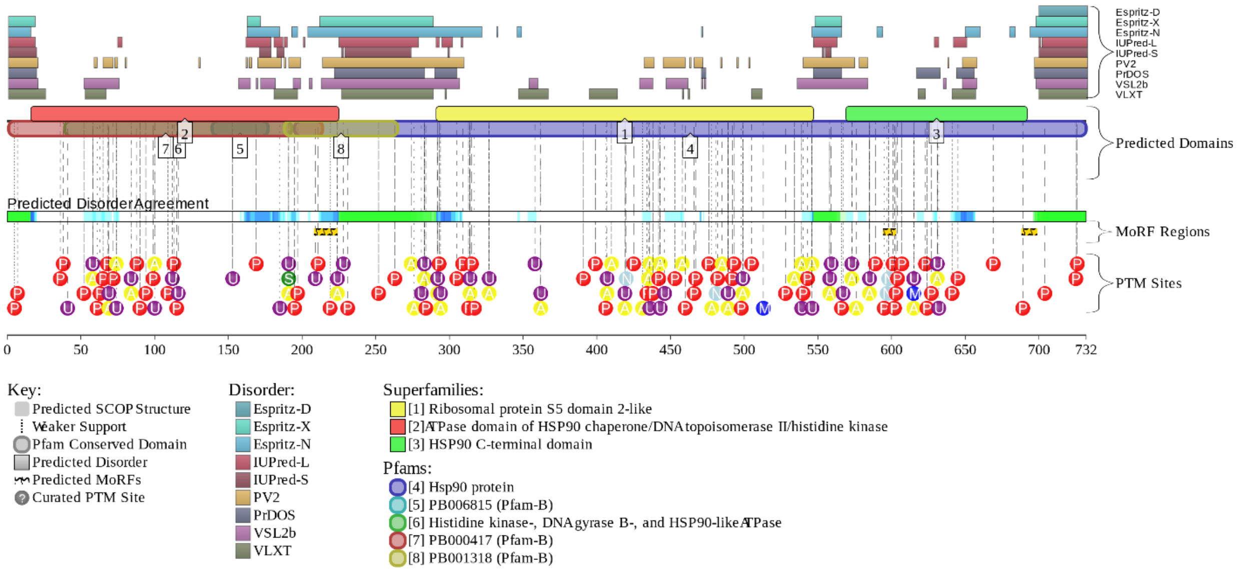
Esspritz-D
Espritz-X
Espritz-N
IUPred-L
IUPred-S
PV2
PrDOS
VSL2b
VLXT
Predicted Domains
Predicted Disorder Agreement
MoRF Regions
PTM Sites
0
50
100
150
200
250
300
350
400
450
500
550
600
650
700
732
Key:
Predicted SCOP Structure
Weaker Support
Pfam Conserved Domain
Predicted Disorder
Predicted MoRFs
Curated PTM Site
Disorder:
Espritz-D
Espritz-X
Espritz-N
IUPred-L
IUPred-S
PV2
PrDOS
VSL2b
VLXT
Superfamilies:
[1] Ribosomal protein S5 domain 2-like
[2] ATPase domain of HSP90 chaperone/DNA topoisomerase II/histidine kinase
[3] HSP90 C-terminal domain
Pfams:
[4] Hsp90 protein
[5] PB006815 (Pfam-B)
[6] Histidine kinase-, DNA gyrase B-, and HSP90-like ATPase
[7] PB000417 (Pfam-B)
[8] PB001318 (Pfam-B)

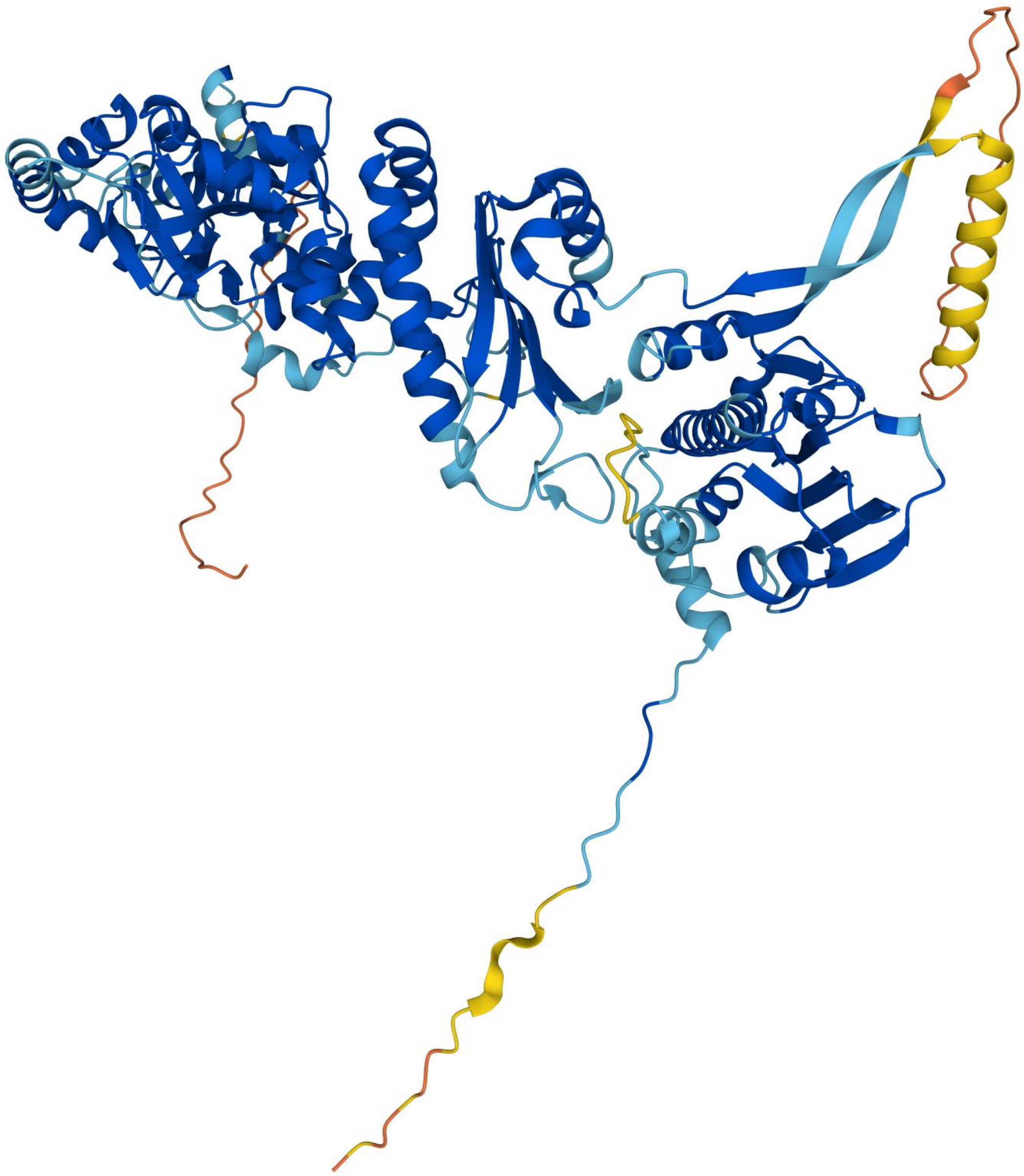

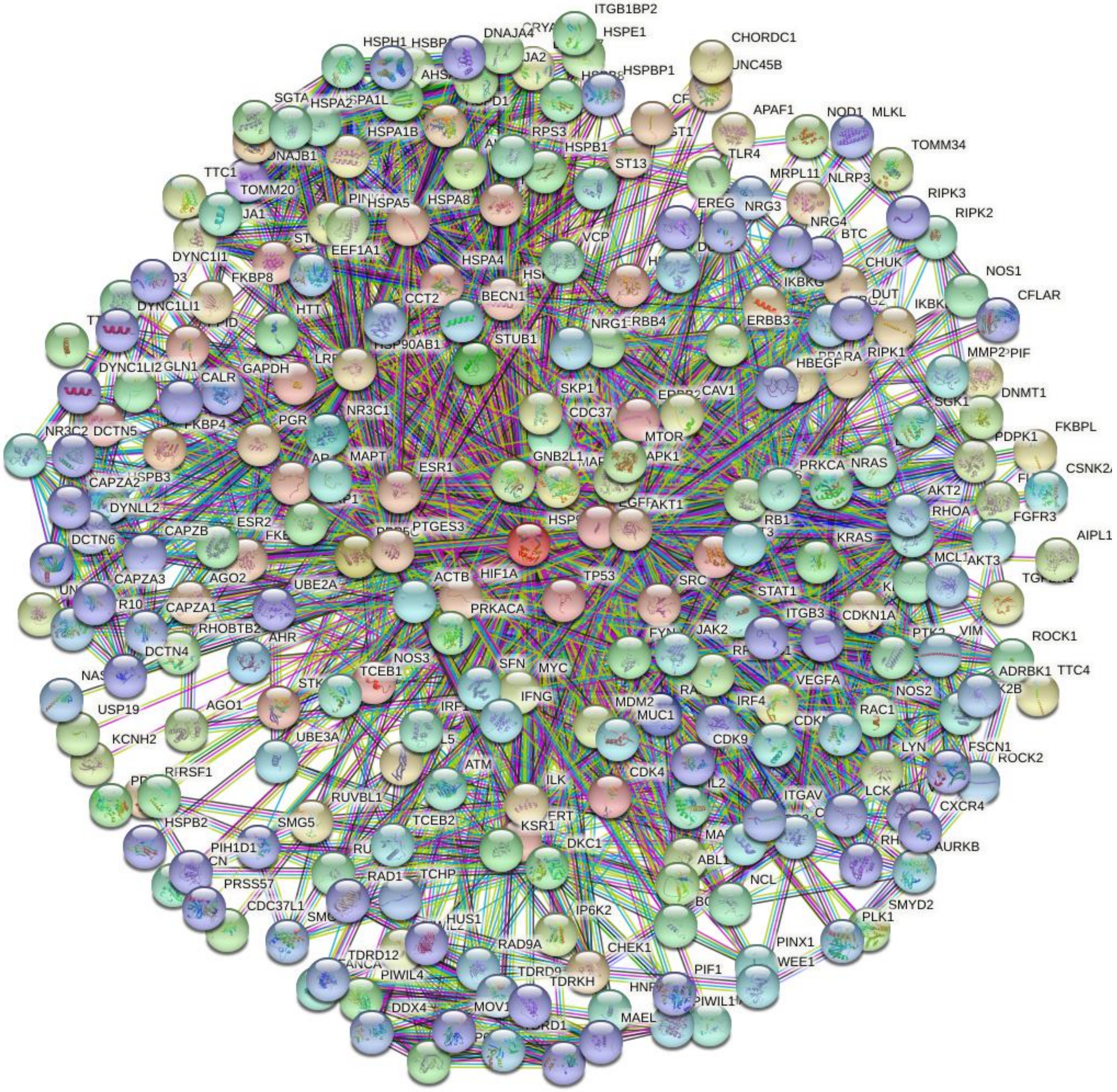

minimum required interaction score: highest confidence (0.900)
number of nodes: 245
number of edges: 1782
average node degree: 14.5
avg. local clustering coefficient: 0.63
expected number of edges: 798
PPI enrichment p-value: < 1.0e-16

Supplementary Figure S2. Functional disorder in human proteins interacting with the RABV M-protein. For each protein, an amino acid sequence in FASTA format is shown followed by the disorder profile generated by RIDAO, D^{2}P^2-generated functional disorder profile, modeled 3D structure generated by AlphaFold, and STRING-based protein-protein interaction network.

RelAp43 (UniProt ID: Q04206)

>sp|Q04206|TF65_HUMAN Transcription factor p65 OS=Homo sapiens OX=9606 GN=RELA PE=1 SV=2

MDELFPLIFPAEPAQASGPYVEIIEQPKQRGMRFRYKCEGRSAGSIPGERSTDTTKTHPTI
KINGYTGPGTVRISLVTKDPPHRPHPHELVGKDCRDGFYEAELCPDRCIHSFQNLGIQCV
KKRDLEQAISQRIQTNNNPFQVPIEEQRGDYDLNAVRLCFQVTVRDPSGRPLRLPPVLSH
PIFDNRAPNTAELKICRVNRNSGSCLGGDEIFLLCDKVQKEDIEVYFTGPGWEARGSFSQ
ADVHRQVAIVFRTPPYADPSLQAPVRVSMQLRRPSDRELSEPMEFQYLPDTDDRHRIEE
KRKRTYETFKSIMKKSPFSGPTDPRPPPRRIAVPSRSSASVPKPAPQPYPFTSSLSTINYDEF
PTMVFPSGQISQASALAPAPPQVLPQAPAPAPAPAMVSALAQAPAPVPVLAPGPPQAVA
PPAPKPTQAGEGTLSEALLQLQFDDEDLGALLGNSTDPAVFTDLASVDNSEFQQLLNQGI
PVAPHTTEPMLMEYPEAITRLVTGAQRPPDPAPAPLGAPGLPNGLLSGDEDFSSIADMDF
SALLSQISS

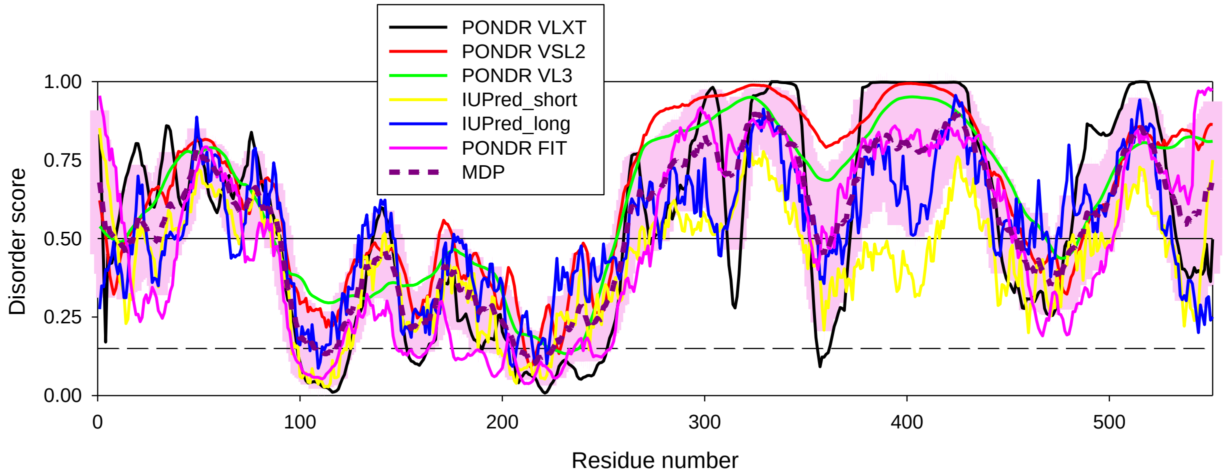

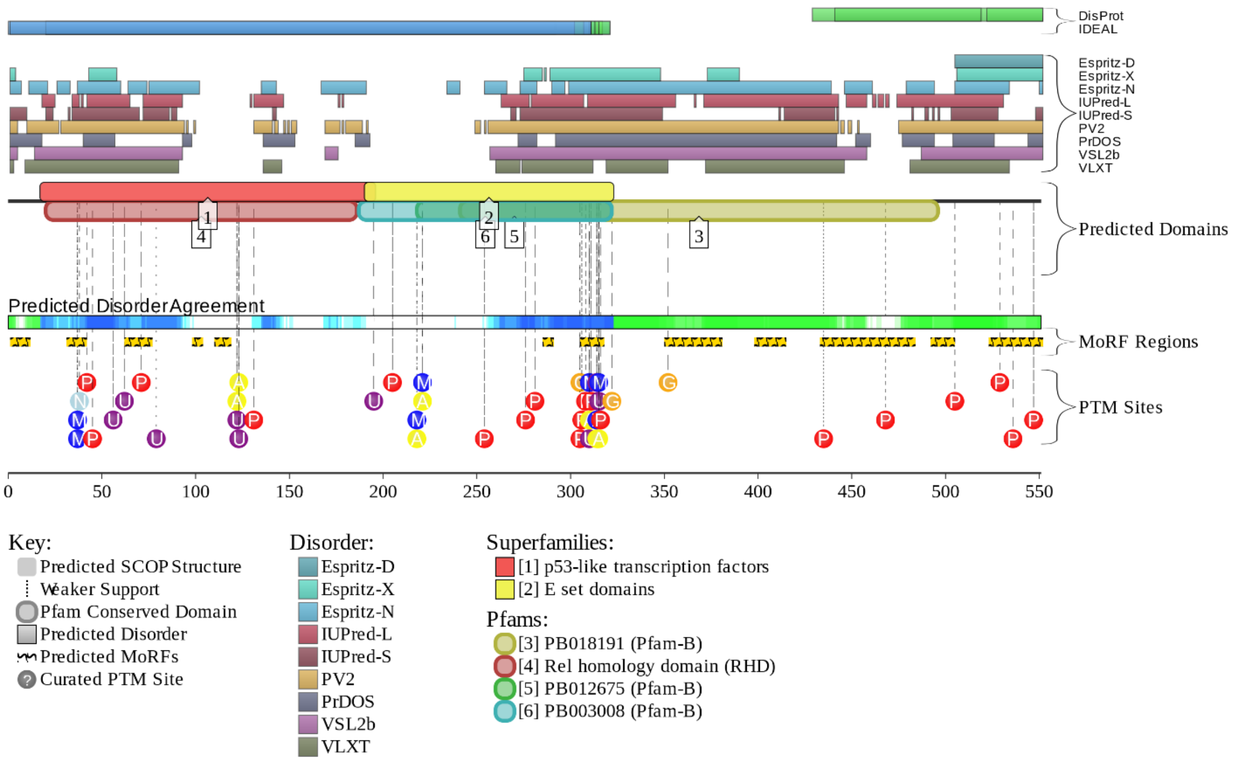
DisProt
IDEAL
Espritz-D
Espritz-X
Espritz-N
IUPred-L
IUPred-S
PV2
PrDOS
VSL2b
VLXT
Predicted Domains
Predicted Disorder Agreement
MoRF Regions
PTM Sites
0
50
100
150
200
250
300
350
400
450
500
550
Key:
Predicted SCOP Structure
Weaker Support
Pfam Conserved Domain
Predicted Disorder
Predicted MoRFs
Curated PTM Site
Disorder:
Espritz-D
Espritz-X
Espritz-N
IUPred-L
IUPred-S
PV2
PrDOS
VSL2b
VLXT
Superfamilies:
[1] p53-like transcription factors
[2] E set domains
Pfams:
[3] PB018191 (Pfam-B)
[4] Rel homology domain (RHD)
[5] PB012675 (Pfam-B)
[6] PB003008 (Pfam-B)

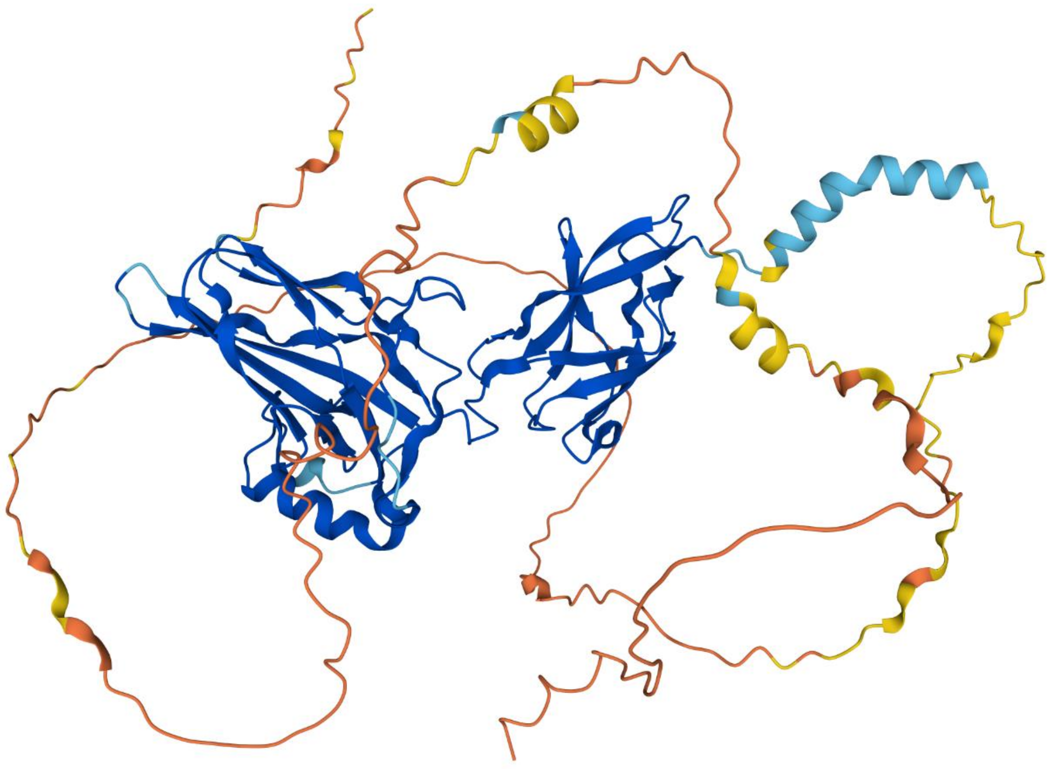

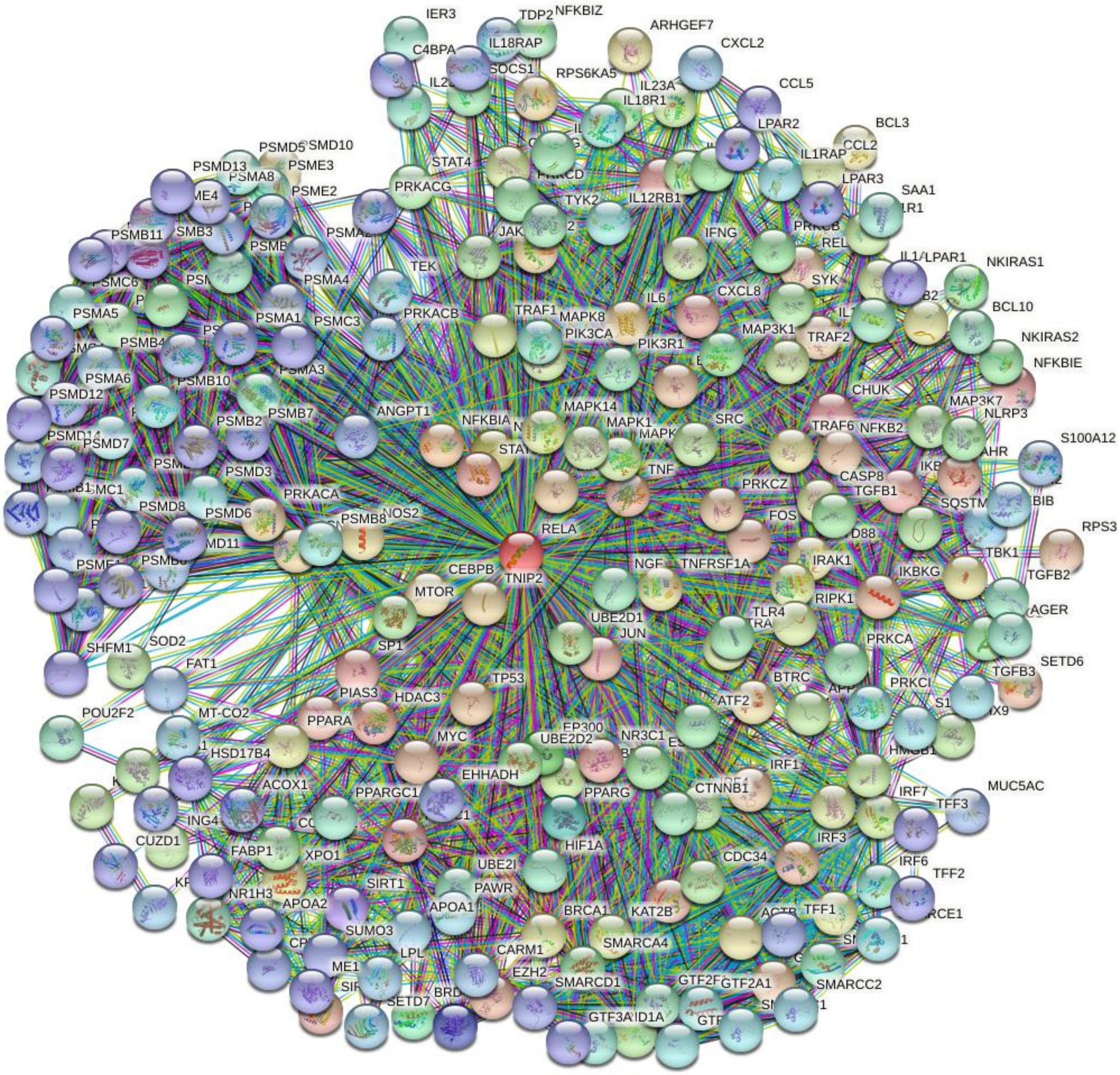

minimum required interaction score: highest confidence (0.900)
number of nodes: 236
number of edges: 3109
average node degree: 26.3
avg. local clustering coefficient: 0.727
expected number of edges: 1044
PPI enrichment p-value: < 1.0e-16

ATP6V1A (UniProt ID: P38606)

>sp|P38606|VATA_HUMAN V-type proton ATPase catalytic subunit A OS=Homo sapiens OX=9606 GN=ATP6V1A PE=1 SV=2

MDFSKLPKILDEDKESTFGYVHGVSGPVVTACDMAGAAMYELVRVGHSELVGEIIRLE
GDMATIQVYEETSGVSVGDPVLRTGKPLSVELGPGIMGAIFDGIQRPLSDISSQTQSIYIPR
GVNVSALSRDIKWDFTPCKNLRVGSHITGGDIYGIVSENSLIKHKIMLPPRNRGTVTYIAP
PGNYDTSDVVLELEFEGVKEKFTMVQVWPVRQVRPVTEKLPANHPLLTGQRVLDALFP
CVQGGTTAIPGAFGCGKTVISQSLSKYSNSDVIIYVGCGERGNEMSEVLRDFPELTMEVD
GKVESIMKRTALVANTSNMPVAAREASIYTGITLSEYFRDMGYHVSMMADSTSRWAEA
LREISGRLAEMPADSGYPAYLGARLASFYERAGRVKCLGNPEREGSVSIVGAVSPPGGD
FSDPVTSATLGIVQVFWGLDKKLAQRKHFPSVNWLISYSKYMRALDEYYDKHFTEFVPL
RTKAKEILQEEEDLAEIVQLVGKASLAETDKITLEVAKLIKDDFLQQNGYTPYDRFCPFY
KTVGMLSNMIAFYDMARRAVETTAQSDNKITWSIIREHMGDILYKLSSMKFKDPLKDG
EAKIKSDYAQLLEDMQNAFRSLED

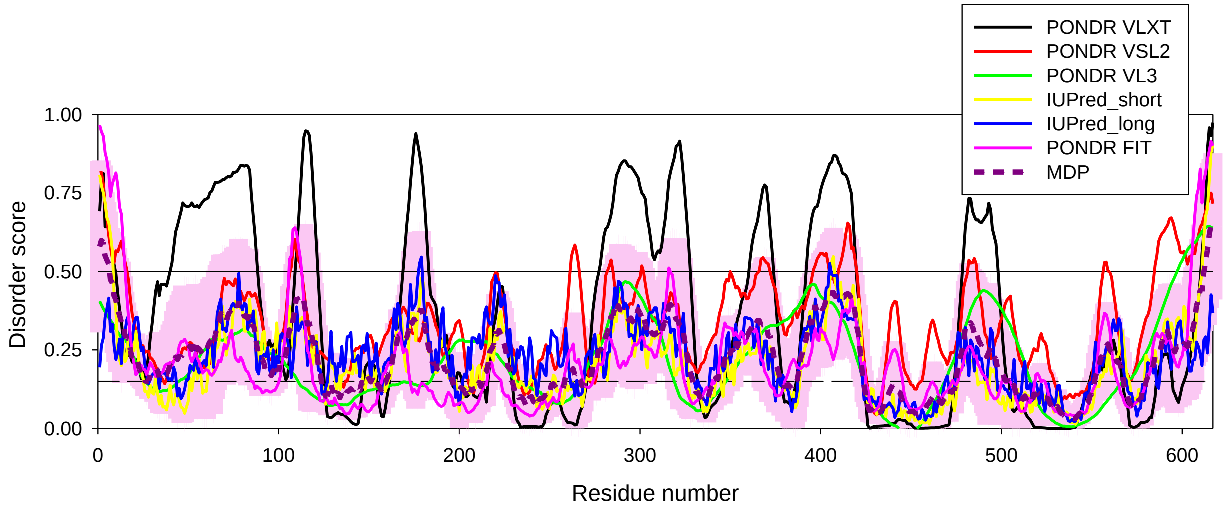

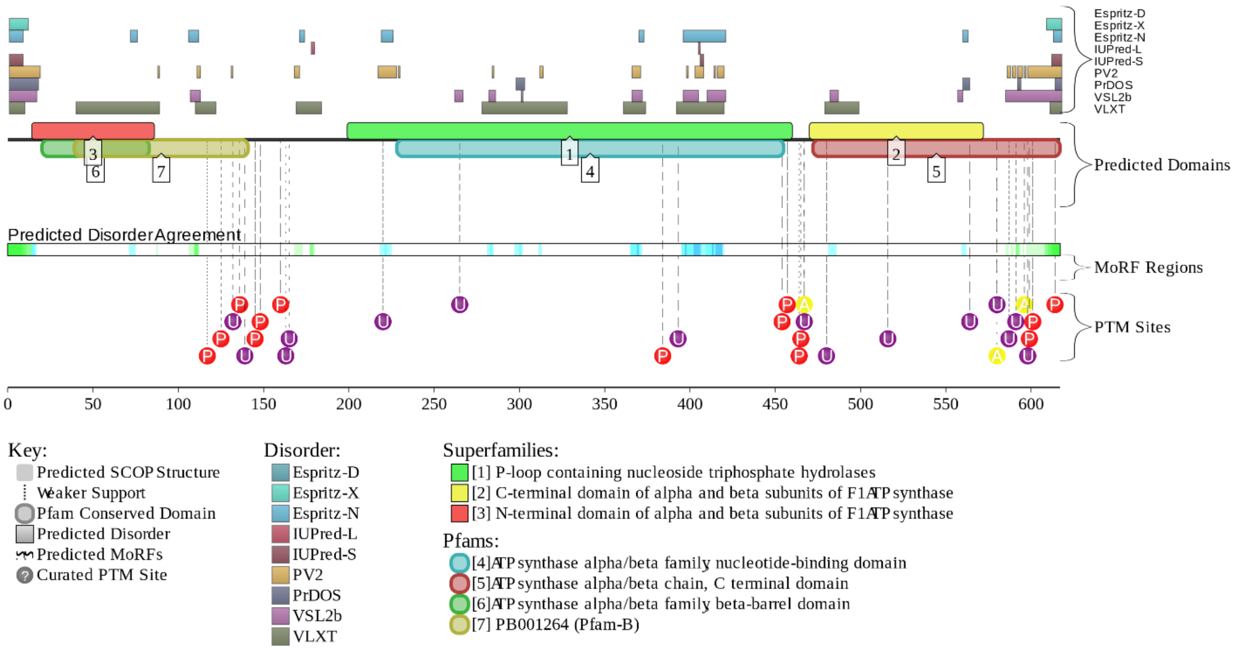
Espritz-D
Espritz-X
Espritz-N
IUPred-L
IUPred-S
PV2
PrDOS
VSL2b
VLXT
Predicted Domains
Predicted Disorder Agreement
MoRF Regions
PTM Sites
0
50
100
150
200
250
300
350
400
450
500
550
600
Key:
Predicted SCOP Structure
Weaker Support
Pfam Conserved Domain
Predicted Disorder
Predicted MoRFs
Curated PTM Site
Disorder:
Espritz-D
Espritz-X
Espritz-N
IUPred-L
IUPred-S
PV2
PrDOS
VSL2b
VLXT
Superfamilies:
[1] P-loop containing nucleoside triphosphate hydrolases
[2] C-terminal domain of alpha and beta subunits of F1 ATP synthase
[3] N-terminal domain of alpha and beta subunits of F1 ATP synthase
Pfams:
[4] ATP synthase alpha/beta family nucleotide-binding domain
[5] ATP synthase alpha/beta chain, C terminal domain
[6] ATP synthase alpha/beta family beta-barrel domain
[7] PB001264 (Pfam-B)

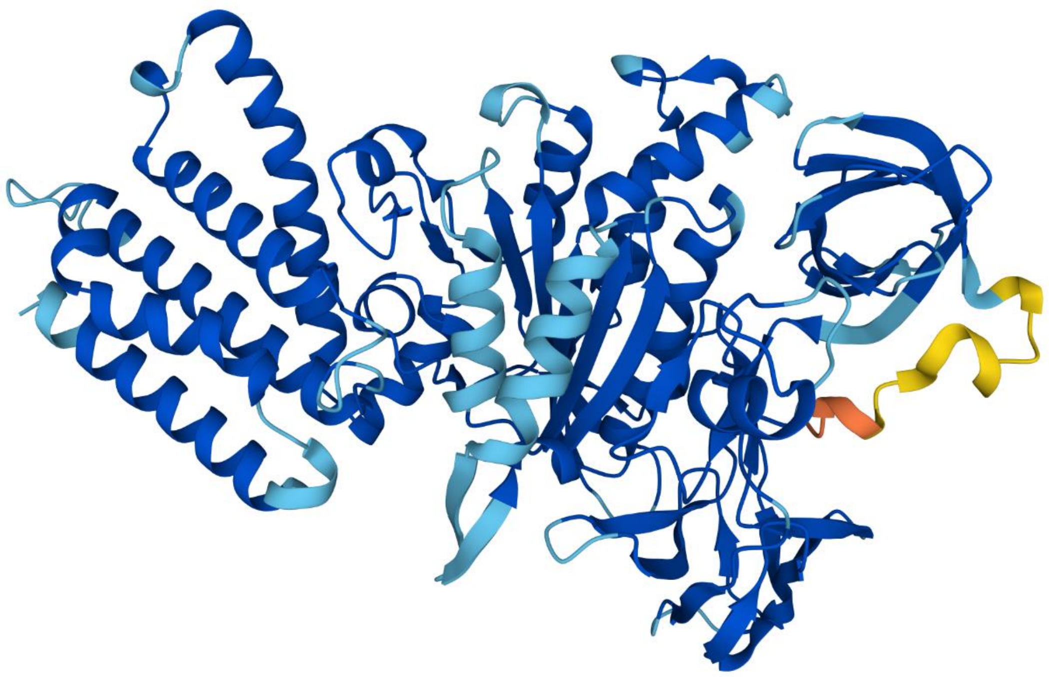

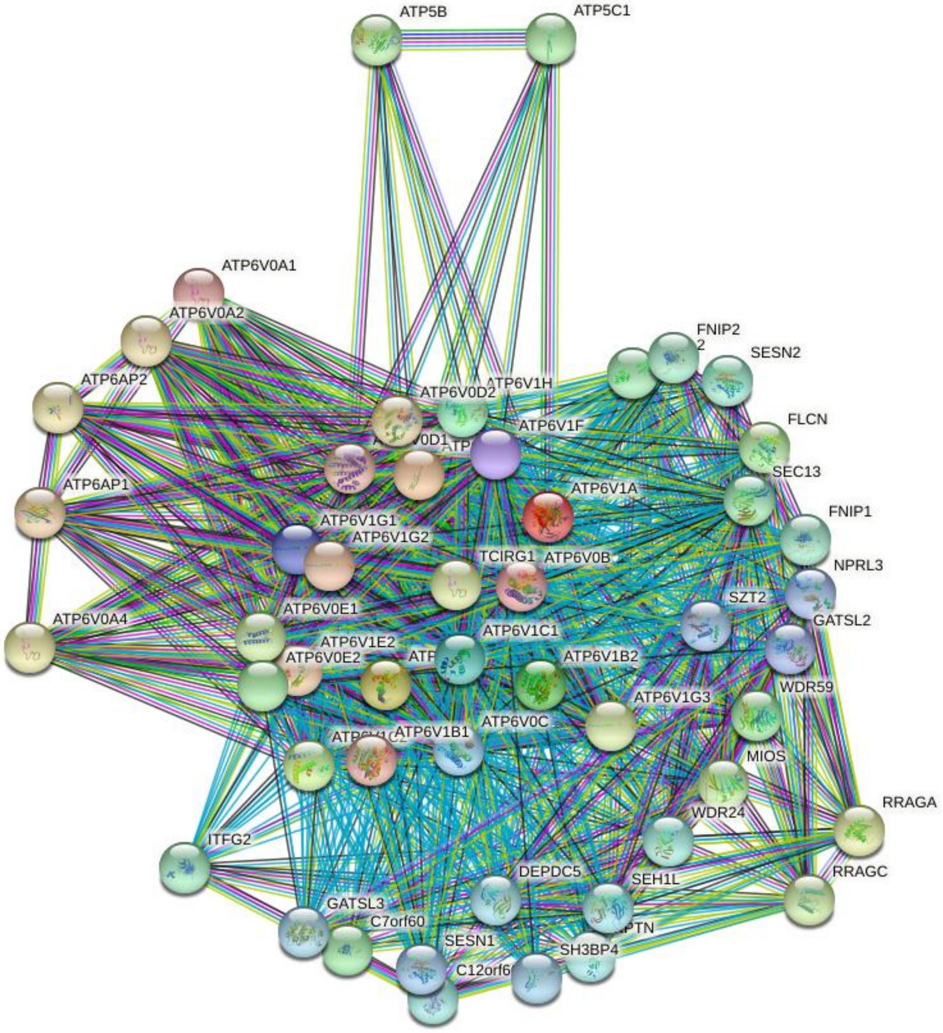

minimum required interaction score: highest confidence (0.900)
number of nodes: 50
number of edges: 828
average node degree: 33.1
avg. local clustering coefficient: 0.801
expected number of edges: 60
PPI enrichment p-value: < 1.0e-16

NEDD4 (UniProT ID: P46934)

>sp|P46934|NEDD4_HUMAN E3 ubiquitin-protein ligase NEDD4 OS=Homo sapiens OX=9606 GN=NEDD4 PE=1 SV=4

MAQSLRLHFAARRSNTYPLSETSGDDLDSHVHMCFKRPTRISTSNVVQMKLTPRQTALA
PLIKENVQSQERSSVPSSENVNKKSSCLQISLQPTRYSGYLQSSNVLADSDDASFTCILKD
GIYSSAVVDNELNAVNDGHLVSSPAICSGSLSNFSTSDNGSYSSNGSDFGSCASITSGGSY
TNSVISDSSSYTFPPSDDTFLGGNLPSDSTSNRSVPNRNTTPCEIFSRSTSTDPFVQDDLEH
GLEIMKLPVSRNTKIPLKRYSSLVIFPRSPSTTRPTSPTSLCTLLSKGSYQTSHQFIISPSEIA
HNEDGTSAKGFLSTAVNGLRLSKTICTPGEVRDIRPLHRKGSLQKKIVLSNNTPRQTVCE
KSSEGYSCVSVHFTQRKAATLDCETTNGDCKPEMSEIKLNSDSEYIKLMHRTSACLPSSQ
NVDCQININGELERPHSQMNKNHGILRRSISLGGAYPNISCLSSLKHNCSKGGPSQLLIKF
ASGNEGKVDNLSRDSNRDCTNELSNSCKTRDDFLGQVDVPLYPLPTENPRLERPYTFKD
FVLHPRSHKSRVKGYLRLKMTYLPKTSGSEDDNAEQAEELEPGWVVLDQPDAACHLQ
QQQEPSPLPPGWEERQDILGRTYYVNHESRRTQWKRPTPQDNLTDAENGNIQLQAQRA
FTTRRQISEETESVDNRESSENWEIIREDEATMYSNQAFPSPPPSSNLDVPTHLAEELNAR
LTIFGNSAVSQPASSSNHSSRRGSLQAYTFEEQPTLPVLLPTSSGLPPGWEEKQDERGRSY
YVDHNSRTTTWTKPTVQATVETSQLTSSQSSAGPQSQASTSDSGQQVTQPSEIEQGFLPK
GWEVRHAPNGRPFFIDHNTKTTTWEDPRLKIPAHLRGKTSLDTSNDLGPLPPGWEERTH
TDGRIFYINHNIKRTQWEDPRLENVAITGPAVPYSRDYKRKYEFFRRKLKKQNDIPNKFE
MKLRRATVLEDSYRRIMGVKRADFLKARLWIEFDGEKGLDYGGVAREWFFLISKEMFN
PYYGLFEYSATDNYTLQINPNSGLCNEDHLSYFKFIGRVAGMAVYHGKLLDGFFIRPFY
KMMLHKPITLHDMESVDSEYYNSLRWILENDPTELDLRFIIDEELFGQTHQHELKNGGSE
IVVTNKNKKEYIYLVIQWRFVNRIQKQMAAFKEGFFELIPQDLIKIFDENELELLMCGLG
DVDVNDWREHTKYKNGYSANHQVIQWFWKAVLMMDSEKRIRLLQFVTGTSRVPMNG
FAELYGSNGPQSFTVEQWGTPEKLPRAHTCFNRLDLPPYESFEELWDKLQMAIENTQGF
DGVD

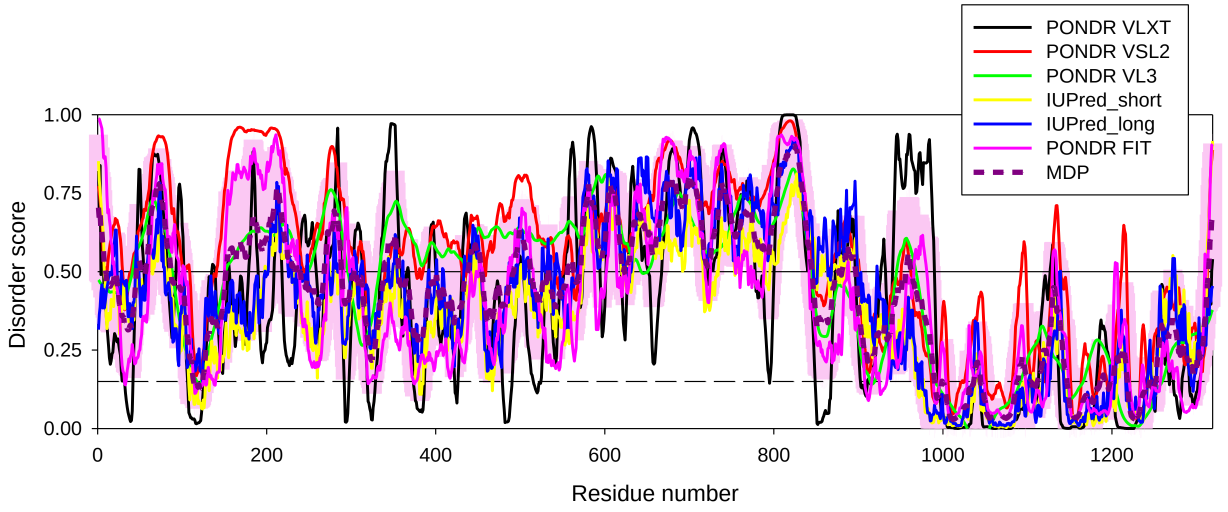

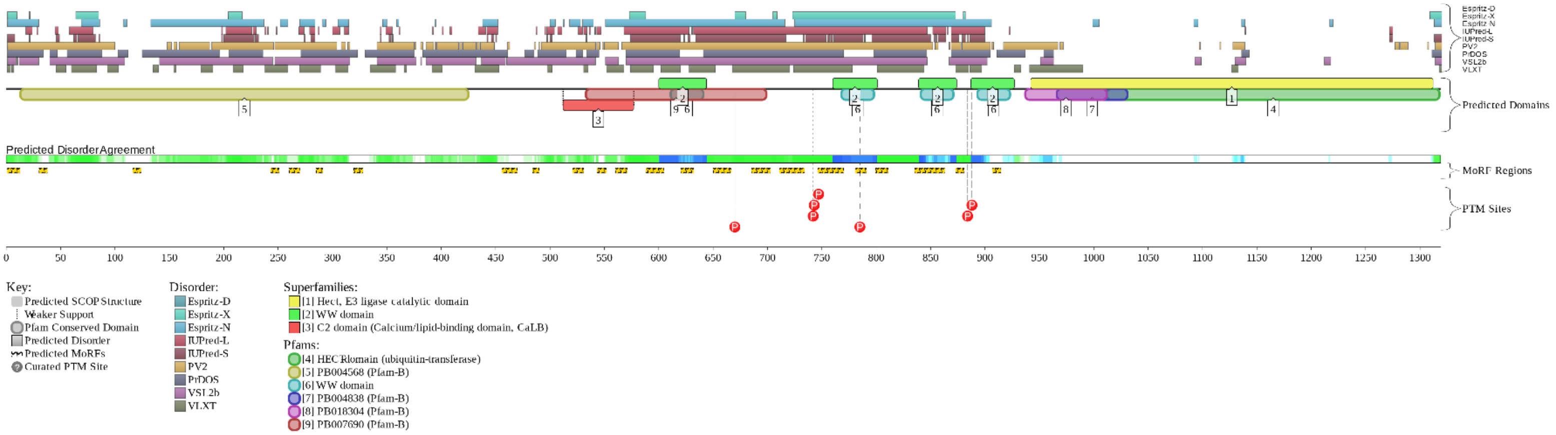
Espritz-D
Espritz-X
Espritz-N
IUPred-L
IUPred-S
PV2
PrDOS
VSL2b
VLXT
Predicted Domains
Predicted Disorder Agreement
MoRF Regions
PTM Sites
0 50 100 150 200 250 300 350 400 450 500 550 600 650 700 750 800 850 900 950 1000 1050 1100 1150 1200 1250 1300
Key:
Predicted SCOP Structure
Weaker Support
Pfam Conserved Domain
Predicted Disorder
Predicted MoRFs
Curated PTM Site
Disorder:
Espritz-D
Espritz-X
Espritz-N
IUPred-L
IUPred-S
PV2
PrDOS
VSL2b
VLXT
Superfamilies:
[1] Hect, E3 ligase catalytic domain
[2] WW domain
[3] C2 domain (Calcium/lipid-binding domain, CaLB)
Pfams:
[4] HECT-domain (ubiquitin-transferase)
[5] PB004568 (Pfam-B)
[6] WW domain
[7] PB004838 (Pfam-B)
[8] PB018304 (Pfam-B)
[9] PB007690 (Pfam-B)

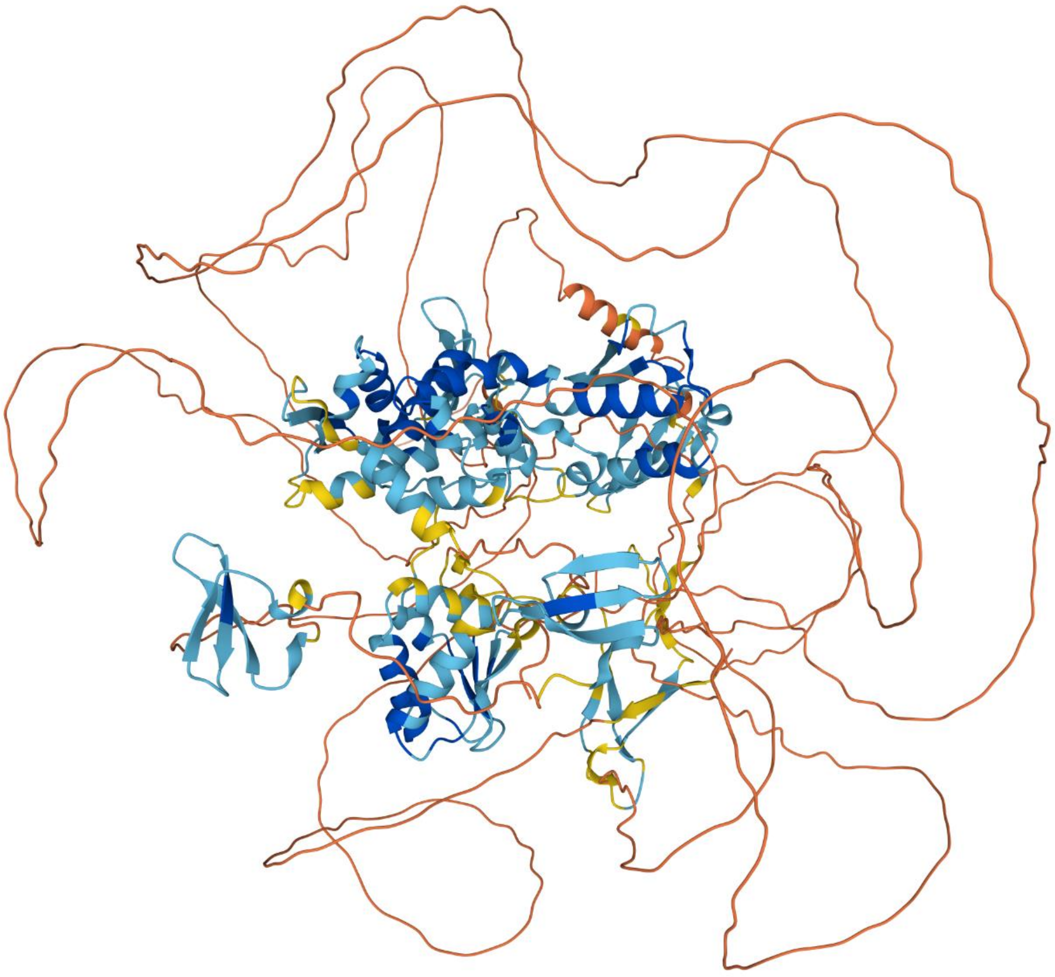

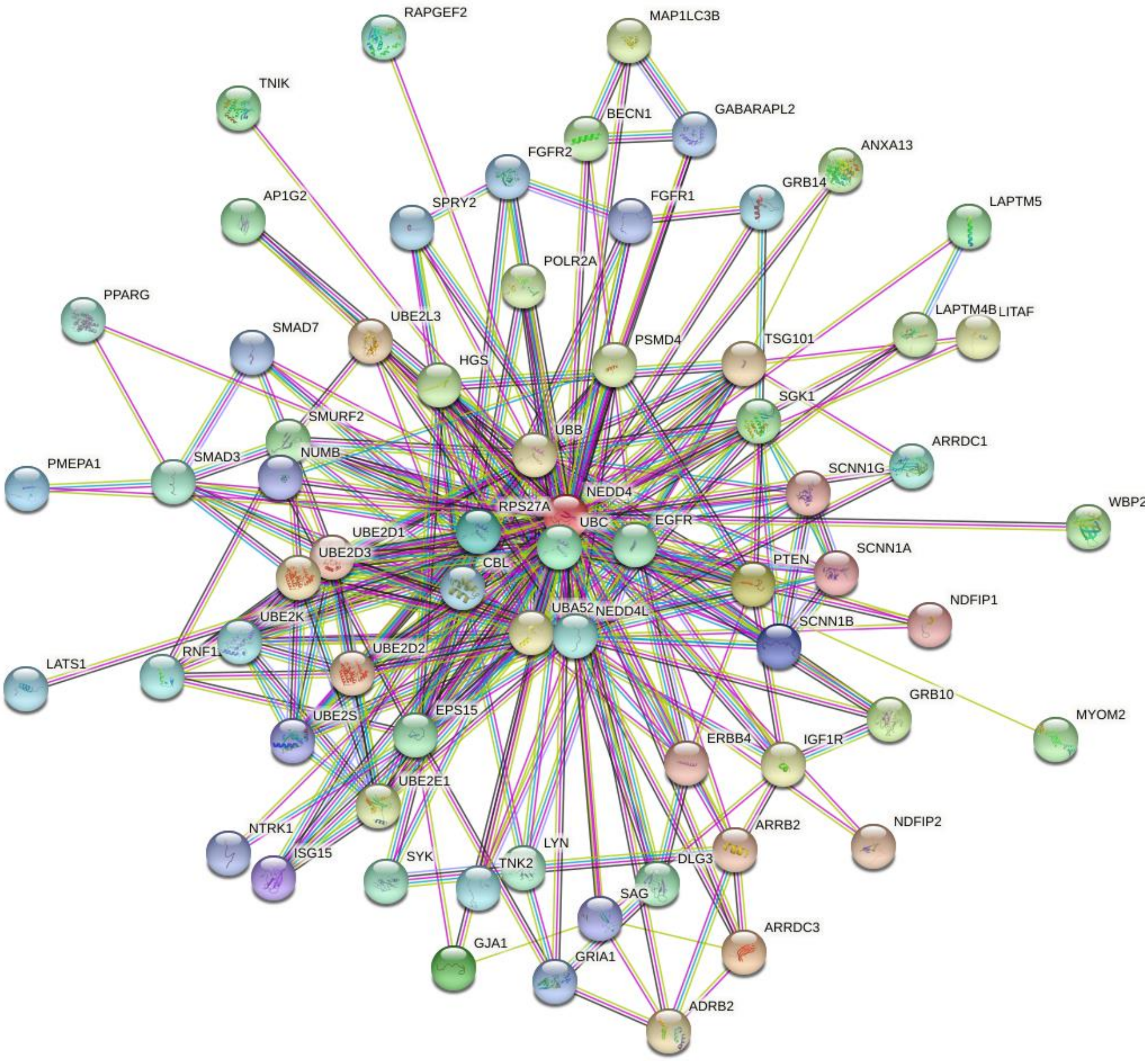

minimum required interaction score: high confidence (0.700)
number of nodes: 67
number of edges: 287
average node degree: 8.57
avg. local clustering coefficient: 0.72
expected number of edges: 132
PPI enrichment p-value: < 1.0e-16

YAP1 (UniProt ID: P46937)

>sp|P46937|YAP1_HUMAN Transcriptional coactivator YAP1 OS=Homo sapiens OX=9606 GN=YAP1 PE=1 SV=2

MDPGQQPPPQPAPQGQGQPPSQPPQGQGPPSGPGQPAPAATQAAPQAPPAGHQIVHVRG
DSETDLEALFNAVMNPKTANVPQTVPMRLRKLPDSFFKPPEPKSHSRQASTDAGTAGAL
TPQHVRAHSSPASLQLGAVSPGTLTPTGVVSGPAATPTAQHLRQSSFEIPDDVPLPAGWE
MAKTSSGQRYFLNHIDQTTTWQDPRKAMLSQMNVTAPTSPPVQQNMMNSASGPLPDG
WEQAMTQDGEIYYINHKNKTTSWLDPRLDPRFAMNQRISQSAPVKQPPPLAPQSPQGG
VMGGSNSNQQQQMRLQQLQMEKERLRLKQQELLRQAMRNINPSTANSPKCQELALRS
QLPTLEQDGGTQNPVSSPGMSQELRTMTTNSSDPFLNSGTYHSRDESTDSGLSMSSYSVP
RTPDDFLNSVDEMDTGDTINQSTLPSQQNRFPDYLEAIPGTNVDLGTLEGDGMNIEGEEL
MPSLQEALSSDILNDMESVLAATKLDKESFLTWL

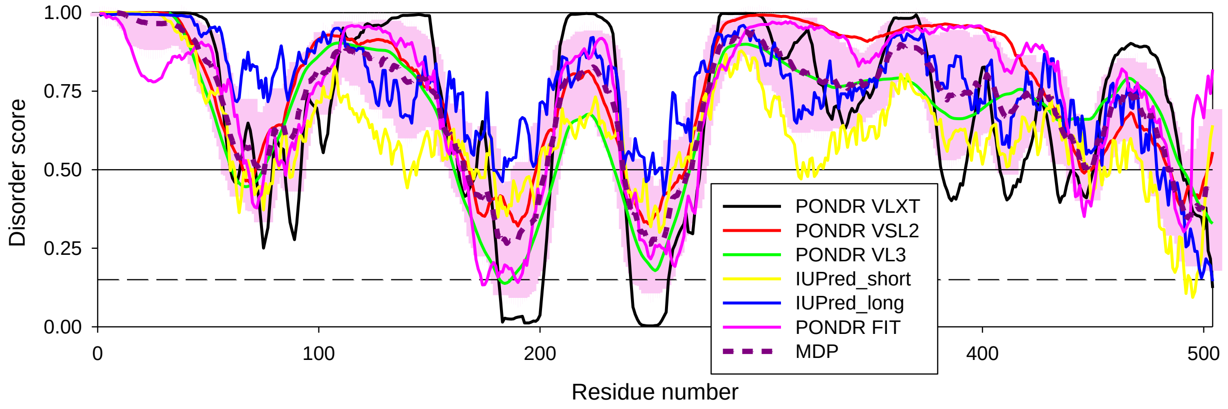

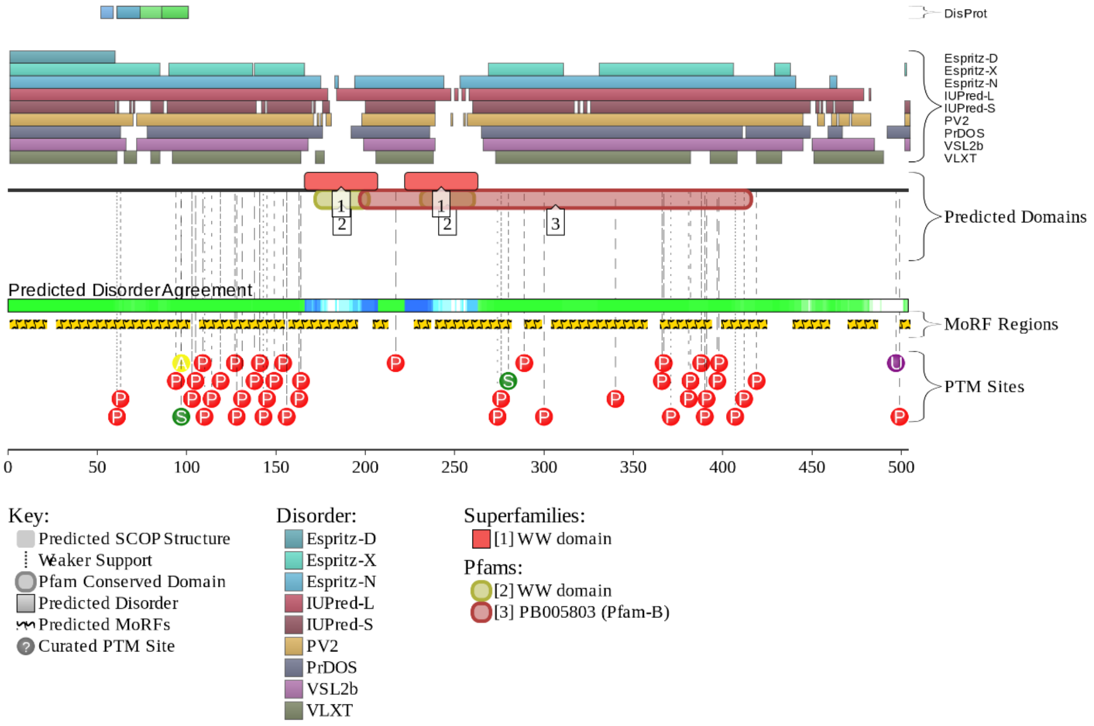

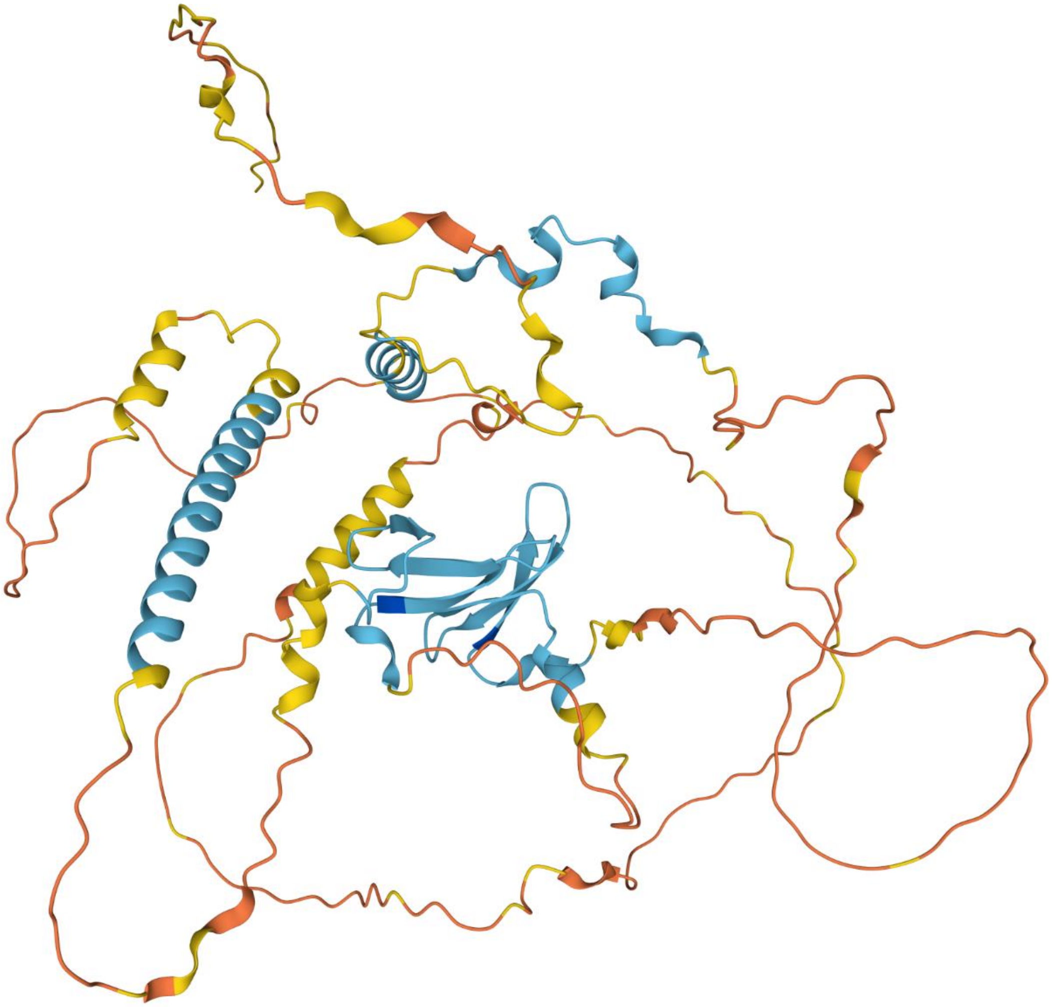

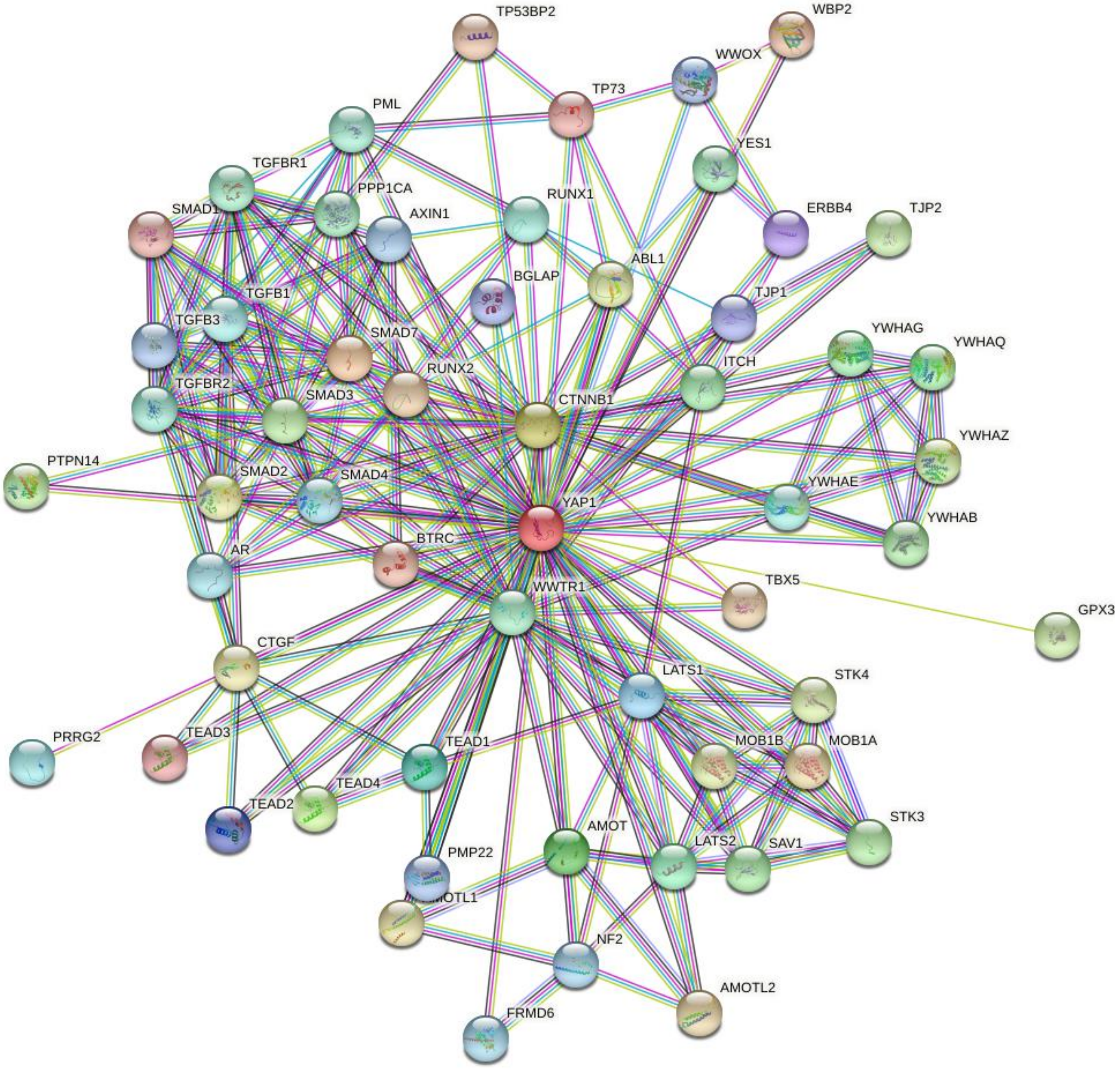

minimum required interaction score: highest confidence (0.900)
number of nodes: 57
number of edges: 241
average node degree: 8.46
avg. local clustering coefficient: 0.754
expected number of edges: 76
PPI enrichment p-value: < 1.0e-16

EIF3H (UniProt ID: O15372)

>sp|O15372|EIF3H_HUMAN Eukaryotic translation initiation factor 3 subunit H OS=Homo sapiens OX=9606 GN=EIF3H PE=1 SV=1

MASRKEGTGSTATSSSSTAGAAGKGKGKGGSGDSAVKQVQIDGLVVLKIIKHYQEEGQ
GTEVVQGVLLGLVVEDRLEITNCFPFPQHTEDDADFDEVQYQMEMMRSLRHVNIDHLH
VGWYQSTYYGSFVTRALLDSQFSYQHAIEESVVLIYDPIKTAQGSLSLKAYRLTPKLME
VCKEKDFSPEALKKANITFEYMFEEVPIVIKNSHLINVLMWELEKKSAVADKHELLSLAS
SNHLGKNLQLLMDRVDEMSQDIVKYNTYMRNTSKQQQQKHQYQQRRQQENMQRQSR
GEPPLPEEDLSKLFKPPQPPARMDSLLIAGQINTYCQNIKEFTAQNLGKLFMAQALQEYN
N

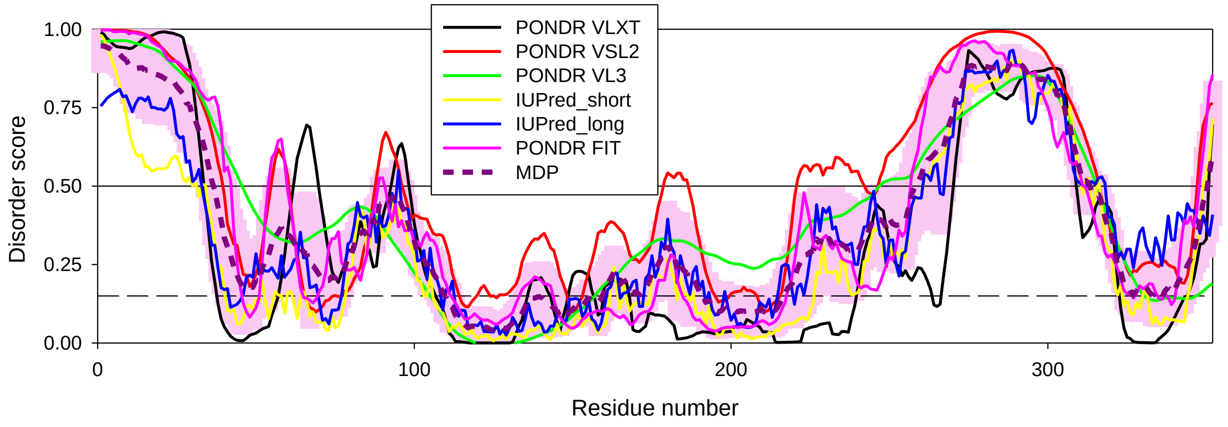

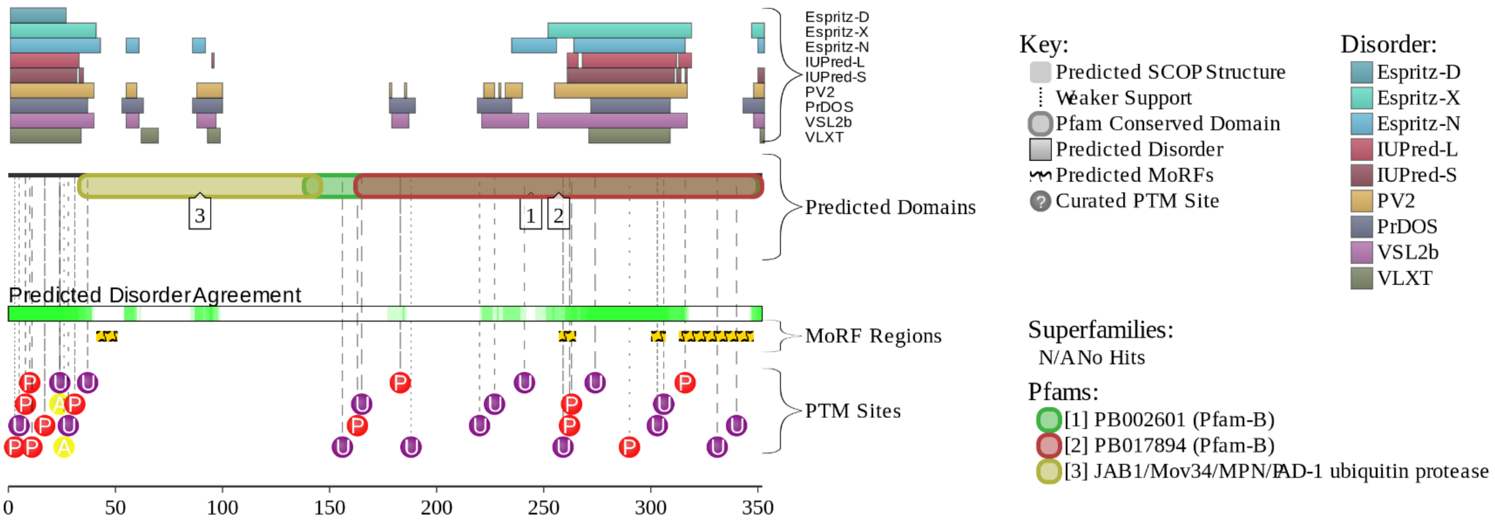

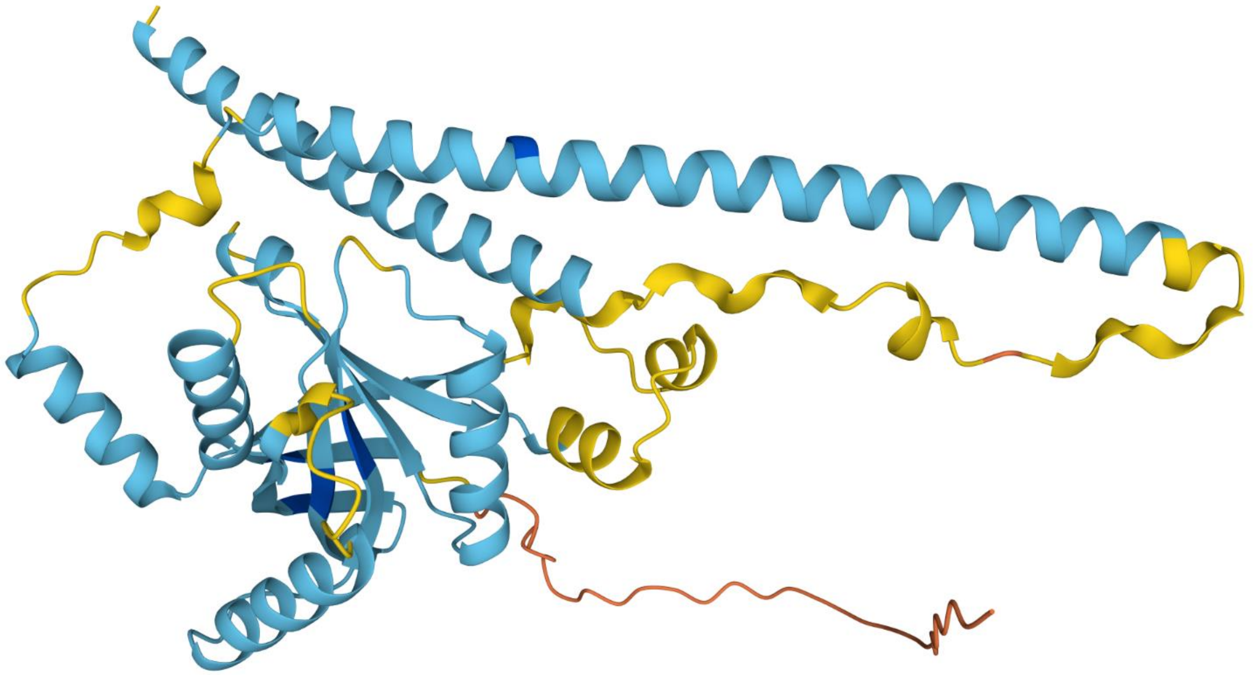

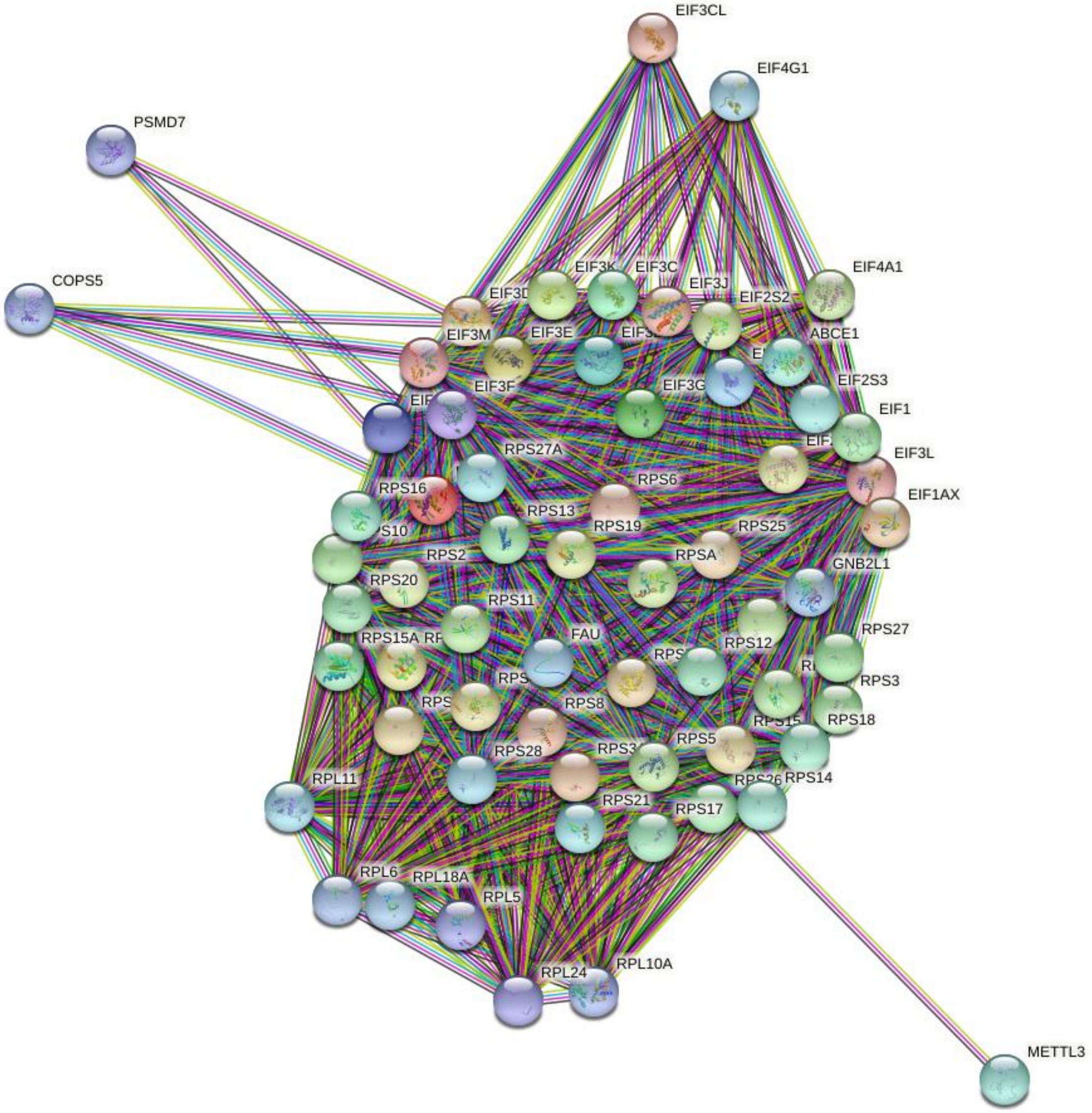

minimum required interaction score: highest confidence (0.900)
number of nodes: 64
number of edges: 1654
average node degree: 51.7
avg. local clustering coefficient: 0.941
expected number of edges: 214
PPI enrichment p-value: < 1.0e-16

JAK1 (UniProt ID: P23458)

>sp|P23458|JAK1_HUMAN Tyrosine-protein kinase JAK1 OS=Homo sapiens OX=9606 GN=JAK1 PE=1 SV=2

MQYLNIKEDCNAMAFCAKMRSSKKTEVNLEAPEPGVEVIFYLSDREPLRLGSGEYTAEE
LCIRAAQACRISPLCHNLFALYDENTKLWYAPNRTITVDDKMSLRLHYRMRFYFTNWH
GTNDNEQSVWRHSPKKQKNGYEKKKIPDATPLLDASSLEYLFAQGQYDLVKCLAPIRD
PKTEQDGHDIENECLGMAVLAISHYAMMKKMQLPELPKDISYKRYIPETLNKSIRQRNL
LTRMRINNVFKDFLKEFNNKTICDSSVSTHDLKVKYLATLETLTKHYGAEIFETSMLLISS
ENEMNWFHSNDGGNVLYYEVMVTGNLGIQWRHKPNVVSVEKEKNKLKRKKLENKHK
KDEEKNKIREEWNNFSYFPEITHIVIKESVVSINKQDNKKMELKLSSHEEALSFVSLVDG
YFRLTADAHHYLCTDVAPPLIVHNIQNGCHGPICTEYAINKLRQEGSEEGMYVLRWSCT
DFDNILMTVTCFEKSEQVQGAQKQFKNFQIEVQKGRYSLHGSDRSFPSLGDLMSHLKKQ
ILRTDNISFMLKRCCQPKPREISNLLVATKKAQEWQPVYPMSQLSFDRILKKDLVQGEHL
GRGTRTHIYSGTLMDYKDDEGTSEEKKIKVILKVLDPSHRDISLAFFEAASMMRQVSHK
HIVYLYGVCVRDVENIMVEEFVEGGPLDLFMHRKSDVLTTPWKFKVAKQLASALSYLE
DKDLVHGNVCTKNLLLAREGIDSECGPFIKLSDPGIPITVLSRQECIERIPWIAPECVEDSK
NLSVAADKWSFGTTLWEICYNGEIPLKDKTLIEKERFYESRCRPVTPSCKELADLMTRC
MNYDPNQRPFFRAIMRDINKLEEQNPDIVSEKKPATEVDPTHFEKRFLKRIRDLGEGHFG
KVELCRYDPEGDNTGEQVAVKSLKPESGGNHIADLKKEIEILRNLYHENIVKYKGICTED
GGNGIKLIMEFLPSGSLKEYLPKNKNKINLKQQLKYAVQICKGMDYLGSRQYVHRDLA
ARNVLVESEHQVKIGDFGLTKAIETDKEYYTVKDDRDSPVFWYAPECLMQSKFYIASDV
WSFGVTLHELLTYCDSDSSPMALFLKMIGPTHGQMTVTRLVNTLKEGKRLPCPPNCPDE
VYQLMRKCWEFQPSNRTSFQNLIEGFEALLK

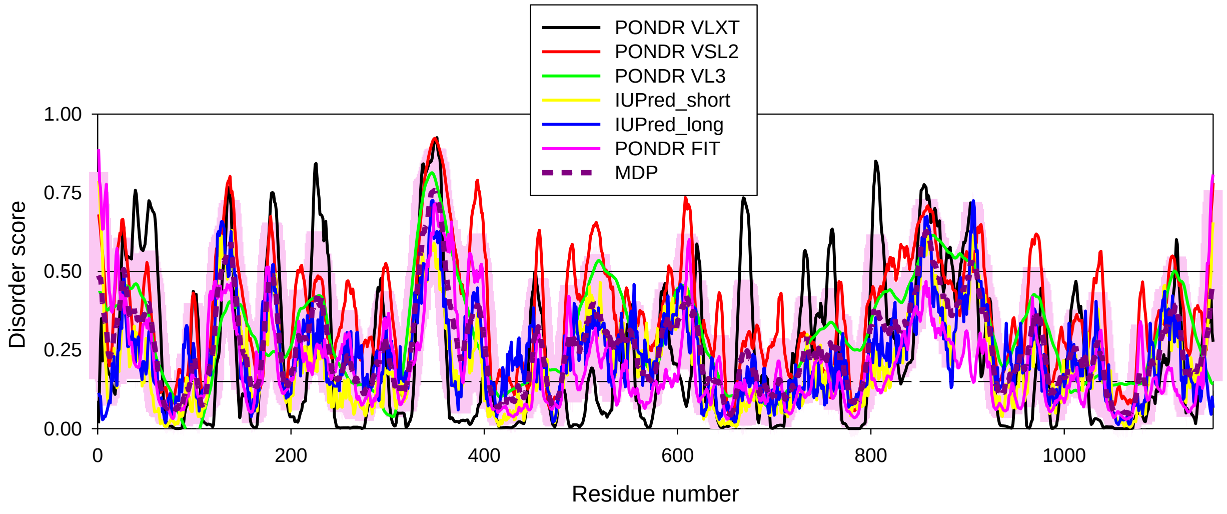

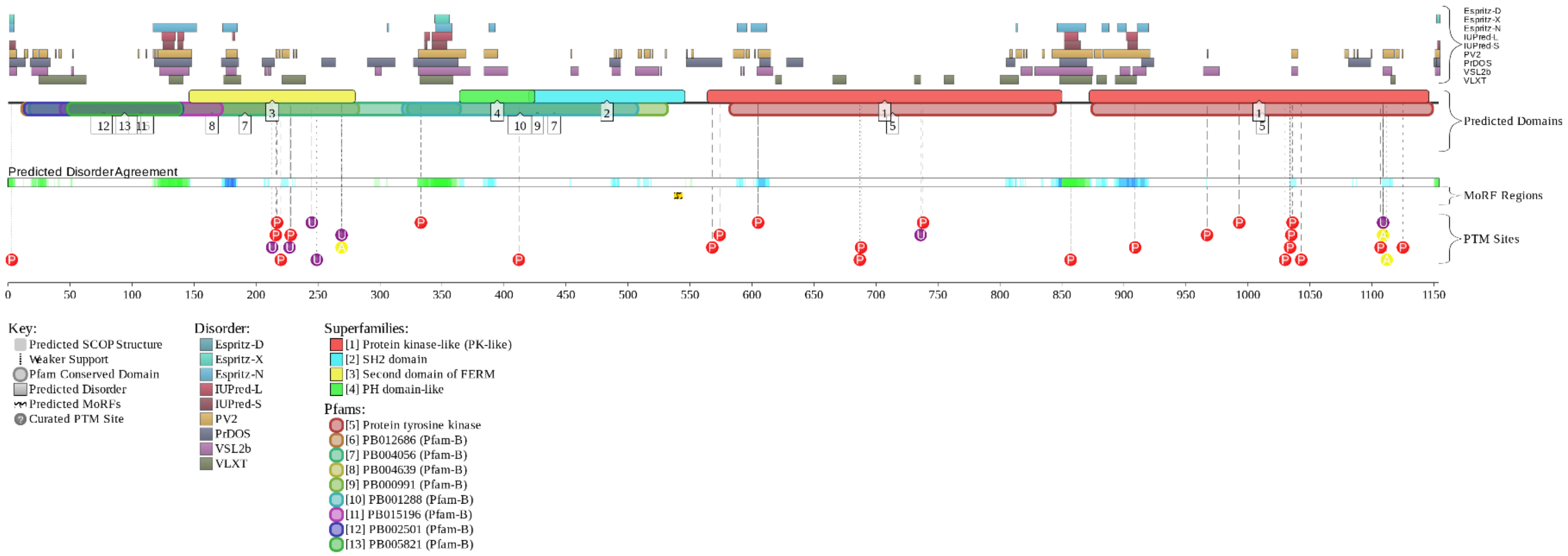
Espritz-D
Espritz-X
Espritz-N
IUPred-L
IUPred-S
PV2
PrDOS
VSL2b
VLXT
Predicted Domains
Predicted DisorderAgreement
MoRF Regions
PTM Sites
0 50 100 150 200 250 300 350 400 450 500 550 600 650 700 750 800 850 900 950 1000 1050 1100 1150
Key:
Predicted SCOP Structure
Weaker Support
Pfam Conserved Domain
Predicted Disorder
Predicted MoRFs
Curated PTM Site
Disorder:
Espritz-D
Espritz-X
Espritz-N
IUPred-L
IUPred-S
PV2
PrDOS
VSL2b
VLXT
Superfamilies:
[1] Protein kinase-like (PK-like)
[2] SH2 domain
[3] Second domain of FERM
[4] PH domain-like
Pfams:
[5] Protein tyrosine kinase
[6] PB012686 (Pfam-B)
[7] PB004056 (Pfam-B)
[8] PB004639 (Pfam-B)
[9] PB000991 (Pfam-B)
[10] PB001288 (Pfam-B)
[11] PB015196 (Pfam-B)
[12] PB002501 (Pfam-B)
[13] PB005821 (Pfam-B)

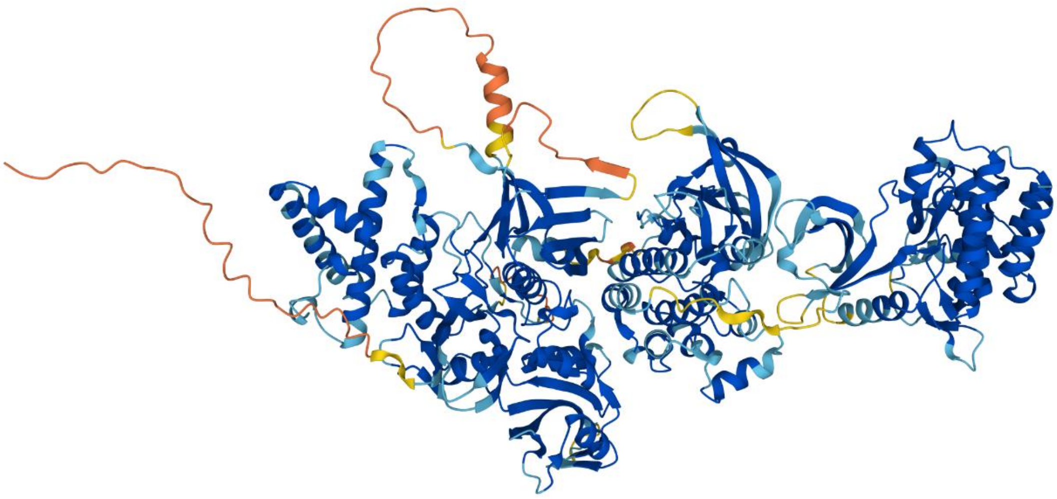

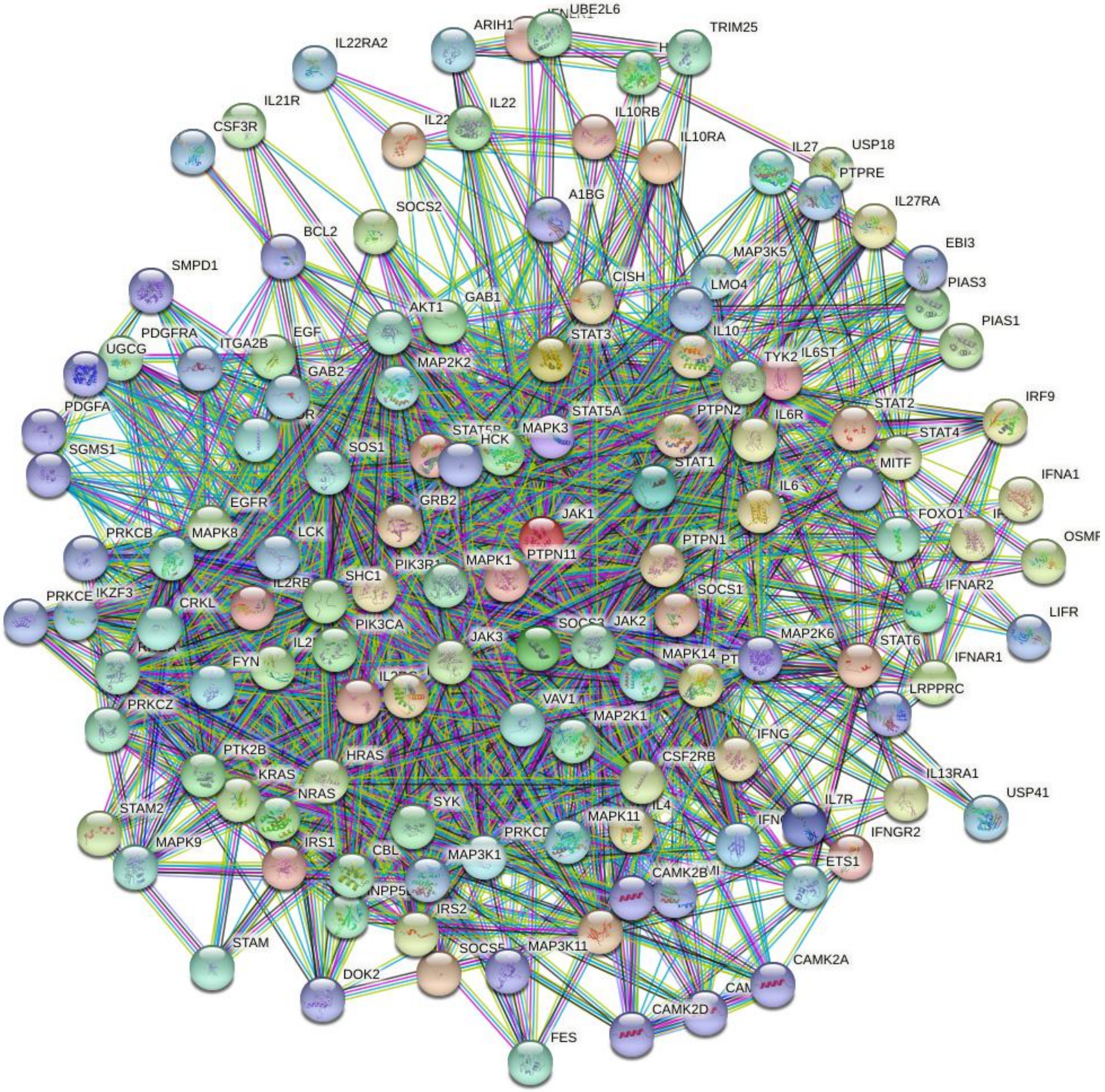

minimum required interaction score: highest confidence (0.900)

number of nodes: 128

number of edges: 1555

average node degree: 24.3

avg. local clustering coefficient: 0.668

expected number of edges: 333

PPI enrichment p-value: < 1.0e-16

STAT1 (UniProt ID: P42224)

>sp|P42224|STAT1_HUMAN Signal transducer and activator of transcription 1-alpha/beta OS=Homo sapiens OX=9606 GN=STAT1 PE=1 SV=2

MSQWYELQQLDSKFLEQVHQLYDDSFPMEIRQYLAQWLEKQDWEHAANDVSFATIRF
HDLLSQLDDQYSRFSLENNFLLQHNIRKSKRNLQDNFQEDPIQMSMIIYSCLKEERKILEN
AQRFNQAQSGNIQSTVMLDKQKELDSKVRNVKDKVMCIEHEIKSLEDLQDEYDFKCKT
LQNREHETNGVAKSDQKQEQLLLKKMYLMLDNKRKEVVHKIIELLNVTELTQNALIND
ELVEWKRRQQSACIGGPPNACLDQLQNWFTIVAESLQQVRQQLKKLEELEQKYTYEHD
PITKNKQVLWDRTFSLFQQLIQSSFVVERQPCMPTHPQRPLVLKTGVQFTVKLRLLVKL
QELNYNLKVKVLFDKDVNERNTVKGFRKFNILGTHTKVMNMEESTNGSLAAEFRHLQL
KEQKNAGTRTNEGPLIVTEELHSLSFETQLCQPGLVIDLETTSLPVVVISNVSQLPSGWAS
ILWYNMLVAEPRNLSFFLTPPCARWAQLSEVLSWQFSSVTKRGLNVDQLNMLGEKLLG
PNASPDGLIPWTRFCKENINDKNFPFWLWIESILELIKKHLLPLWNDGCIMGFISKERERA
LLKDQQPGTFLLRFSESSREGAITFTWVERSQNGGEPDFHAVEPYTKKELSAVTFPDIIRN
YKVMAAENIPENPLKYLYPNIDKDHAFGKYYSRPKEAPEPMELDGPKGTGYIKTELISVS
EVHPSRLQTTDNLLPMSPEEFDEVSRIVGSVEFDSMMNTV

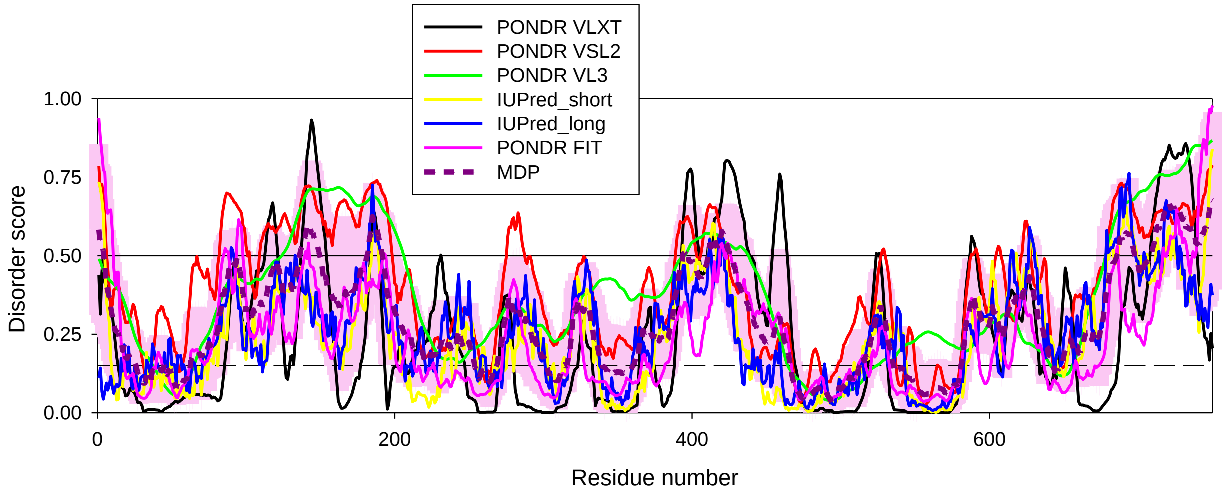

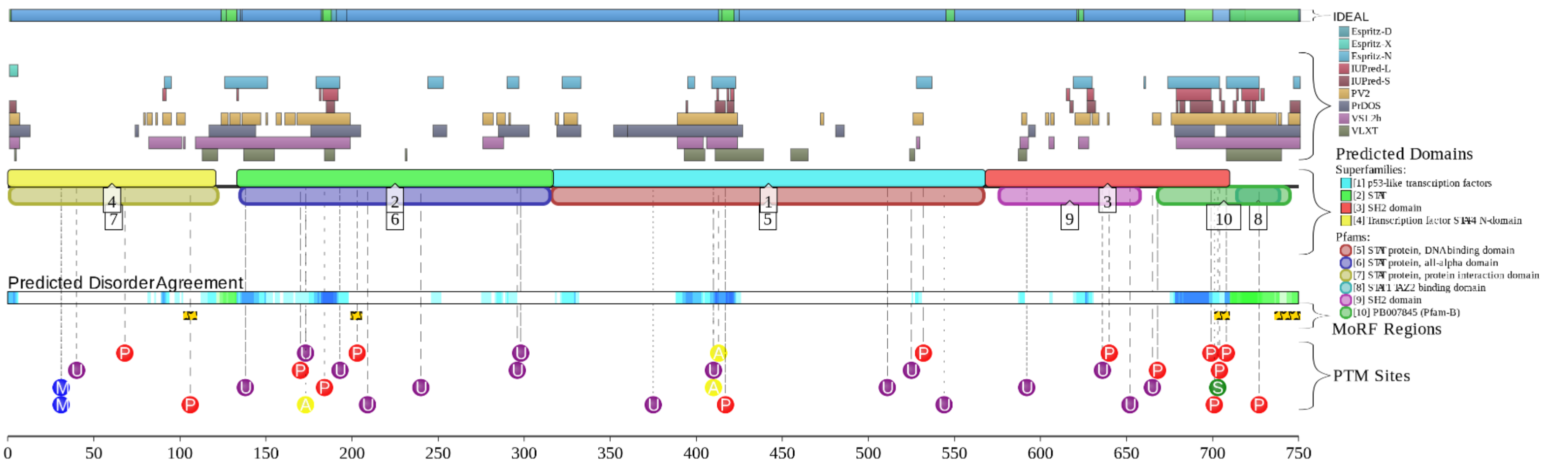

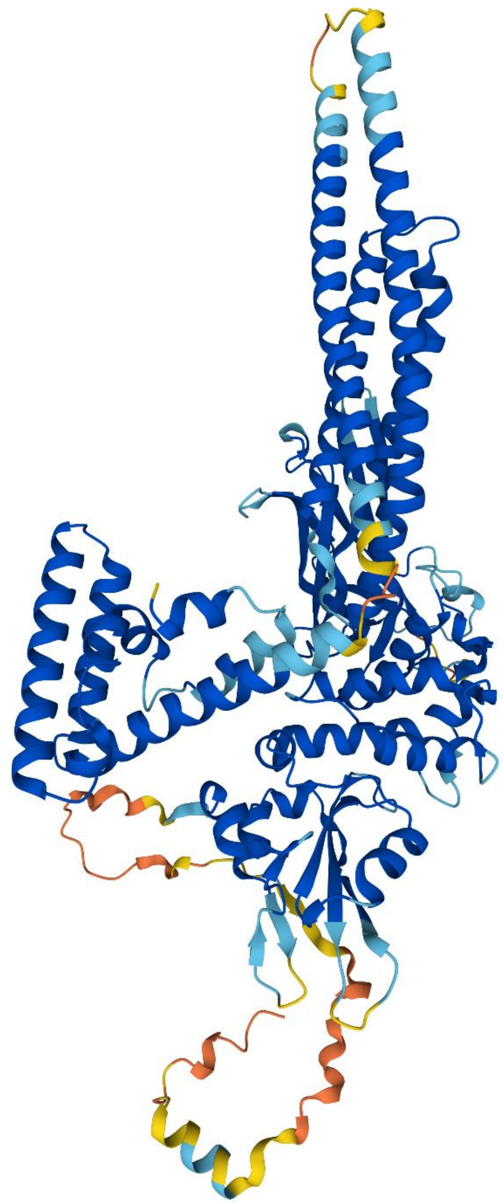

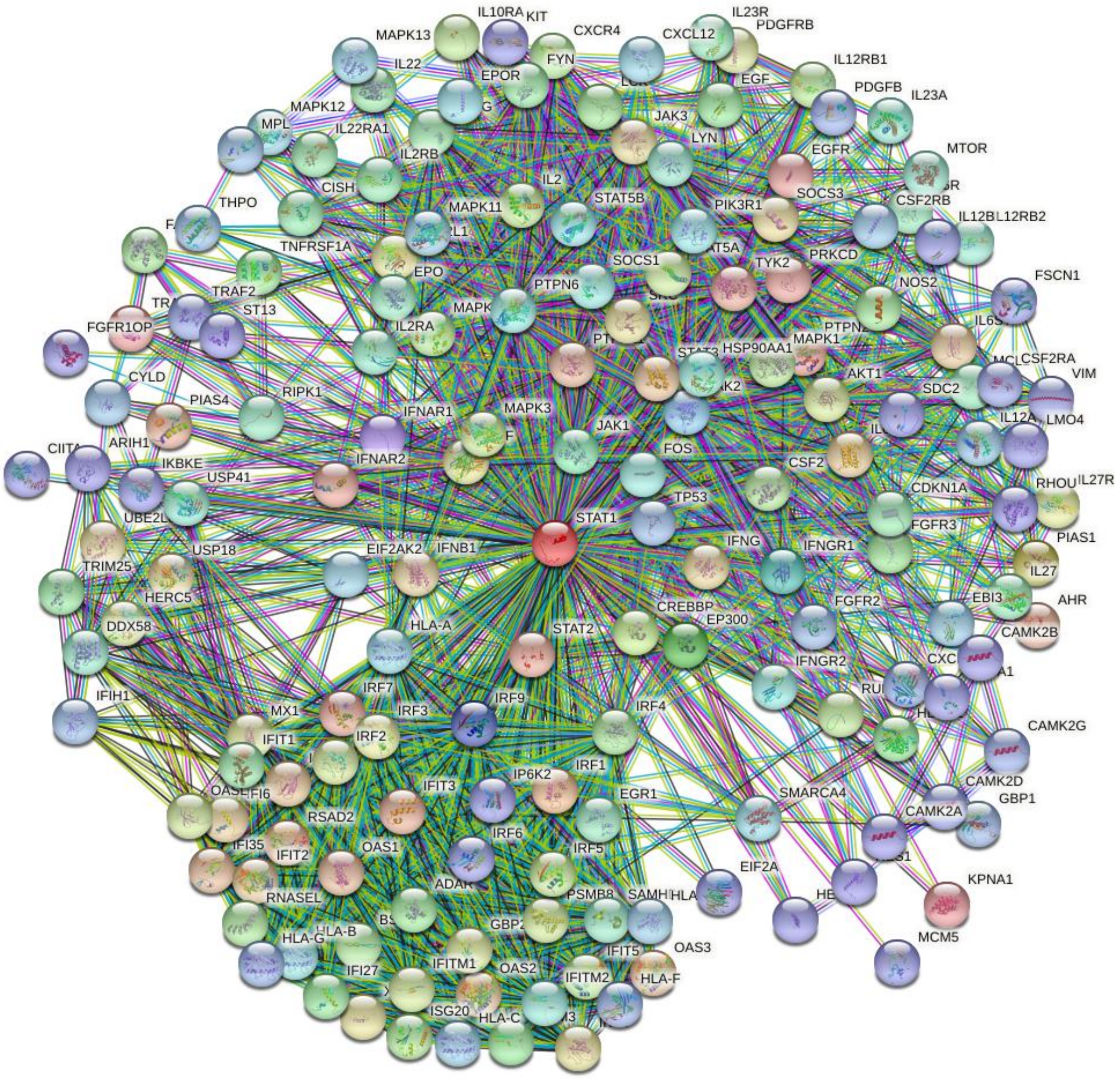

minimum required interaction score: highest confidence (0.900)
number of nodes: 43
number of edges: 257
average node degree: 12
avg. local clustering coefficient: 0.847
expected number of edges: 50
PPI enrichment p-value: < 1.0e-16
number of nodes: 164
number of edges: 2126
average node degree: 25.9
avg. local clustering coefficient: 0.733
expected number of edges: 507
PPI enrichment p-value: < 1.0e-16

Supplementary Figure S3. Functional disorder in human proteins interacting with the RABV N-protein. For each protein, an amino acid sequence in FASTA format is shown followed by the disorder profile generated by RIDAO, D^{2}P^2-generated functional disorder profile, modeled 3D structure generated by AlphaFold, and STRING-based protein-protein interaction network.

HSPA1A (UniProt ID: P0DMV8)

>sp|P0DMV8|HS71A_HUMAN Heat shock 70 kDa protein 1A OS=Homo sapiens OX=9606 GN=HSPA1A PE=1 SV=1

MAKAAAIGIDLGTTYSCVGVFQHGKVEIIANDQGNRTTPSYVAFTDTERLIGDAAKNQV
ALNPQNTVFDAKRLIGRKFGDPVVQSDMKHWPFQVINDGDKPKVQVSYKGETKAFYPE
EISSMVLTKMKEIAEAYLGYPVTNAVITVPAYFNDSQRQATKDAGVIAGLNVLRIINEPT
AAAIAYGLDRTGKGERNVLIFDLGGGTFDVSILTIDDGIFEVKATAGDTHLGGEDFDNRL
VNHFVEEFKRKHKKDISQNKRAVRRLRTACERAKRTLSSSTQASLEIDSLFEGIDFYTSIT
RARFEELCSDLFRSTLEPVEKALRDAKLDKAQIHDLVLVGGSTRIPKVQKLLQDFFNGR
DLNKSINPDEAVAYGAAVQAAILMGDKSENVQDLLLLDVAPLSLGLETAGGVMTALIK
RNSTIPTKQTQIFTTYSDNQPGVLIQVYEGERAMTKDNNLLGRFELSGIPPAPRGVPQIEV
TFDIDANGILNVTATDKSTGKANKITITNDKGRLSKEEIERMVQEAEKYKAEDEVQRER
VSAKNALESYAFNMKSAVEDEGLKGKISEADKKKVLDKCQEVISWLDANTLAEKDEFE
HKRKELEQVCNPIISGLYQGAGGPGPGGFGAQGPKGGSGSGPTIEEVD

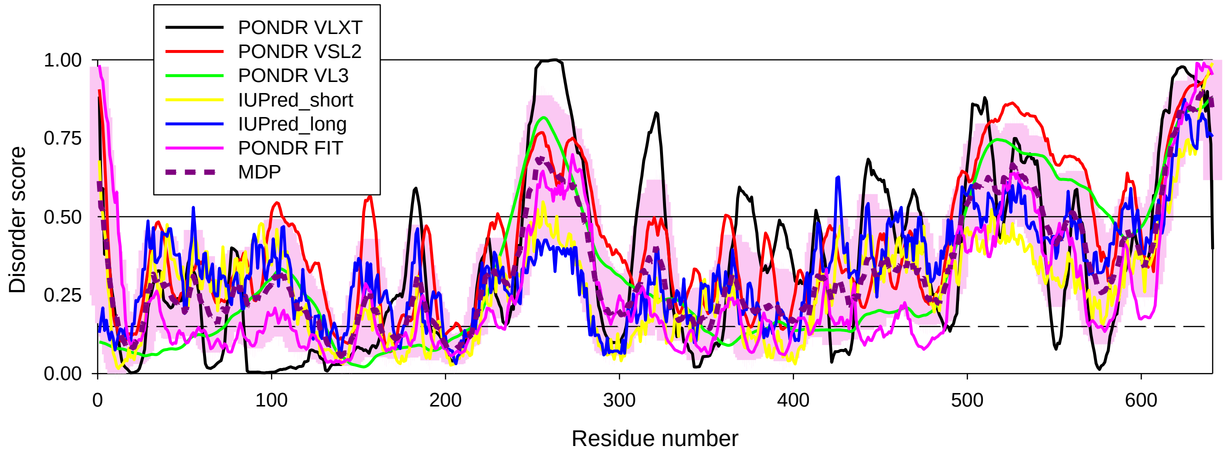

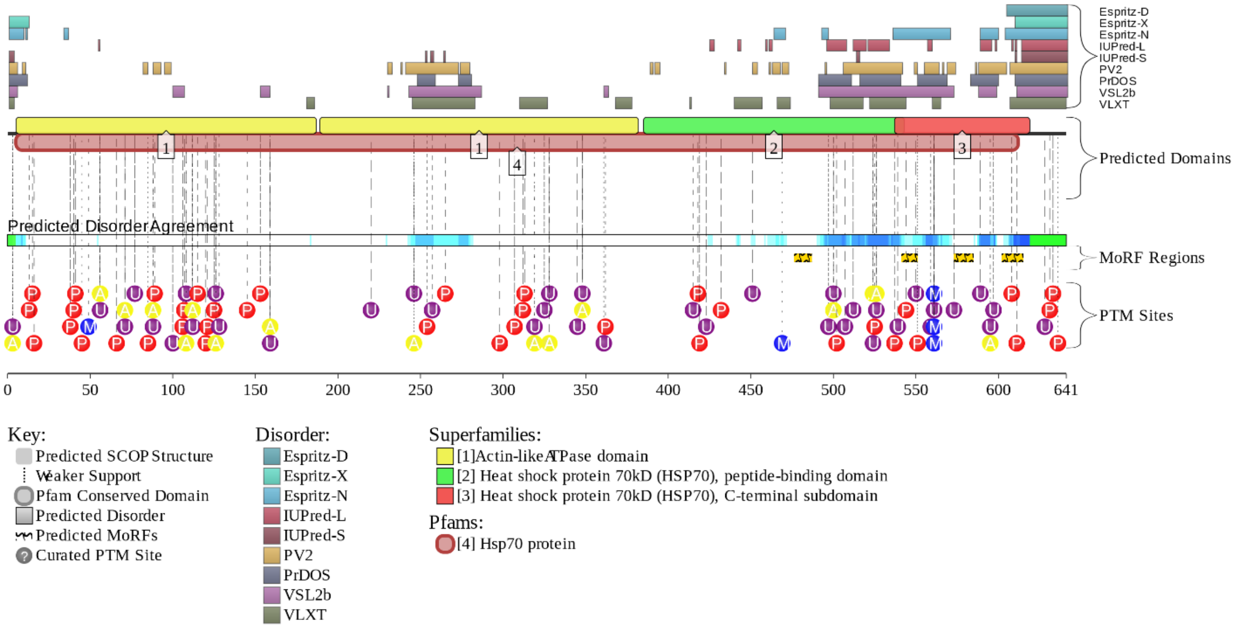
Espritz-D
Espritz-X
Espritz-N
IUPred-L
IUPred-S
PV2
PrDOS
VSL2b
VLXT
Predicted Domains
Predicted Disorder Agreement
MoRF Regions
PTM Sites
0
50
100
150
200
250
300
350
400
450
500
550
600
641
Key:
Predicted SCOP Structure
Weaker Support
Pfam Conserved Domain
Predicted Disorder
Predicted MoRFs
Curated PTM Site
Disorder:
Espritz-D
Espritz-X
Espritz-N
IUPred-L
IUPred-S
PV2
PrDOS
VSL2b
VLXT
Superfamilies:
[1] Actin-like ATPase domain
[2] Heat shock protein 70kD (HSP70), peptide-binding domain
[3] Heat shock protein 70kD (HSP70), C-terminal subdomain
Pfams:
[4] Hsp70 protein

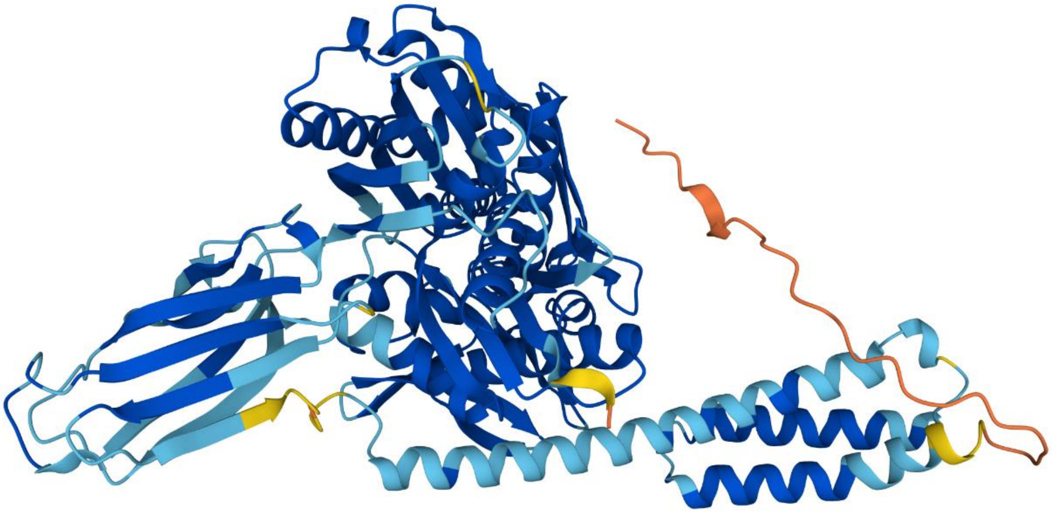

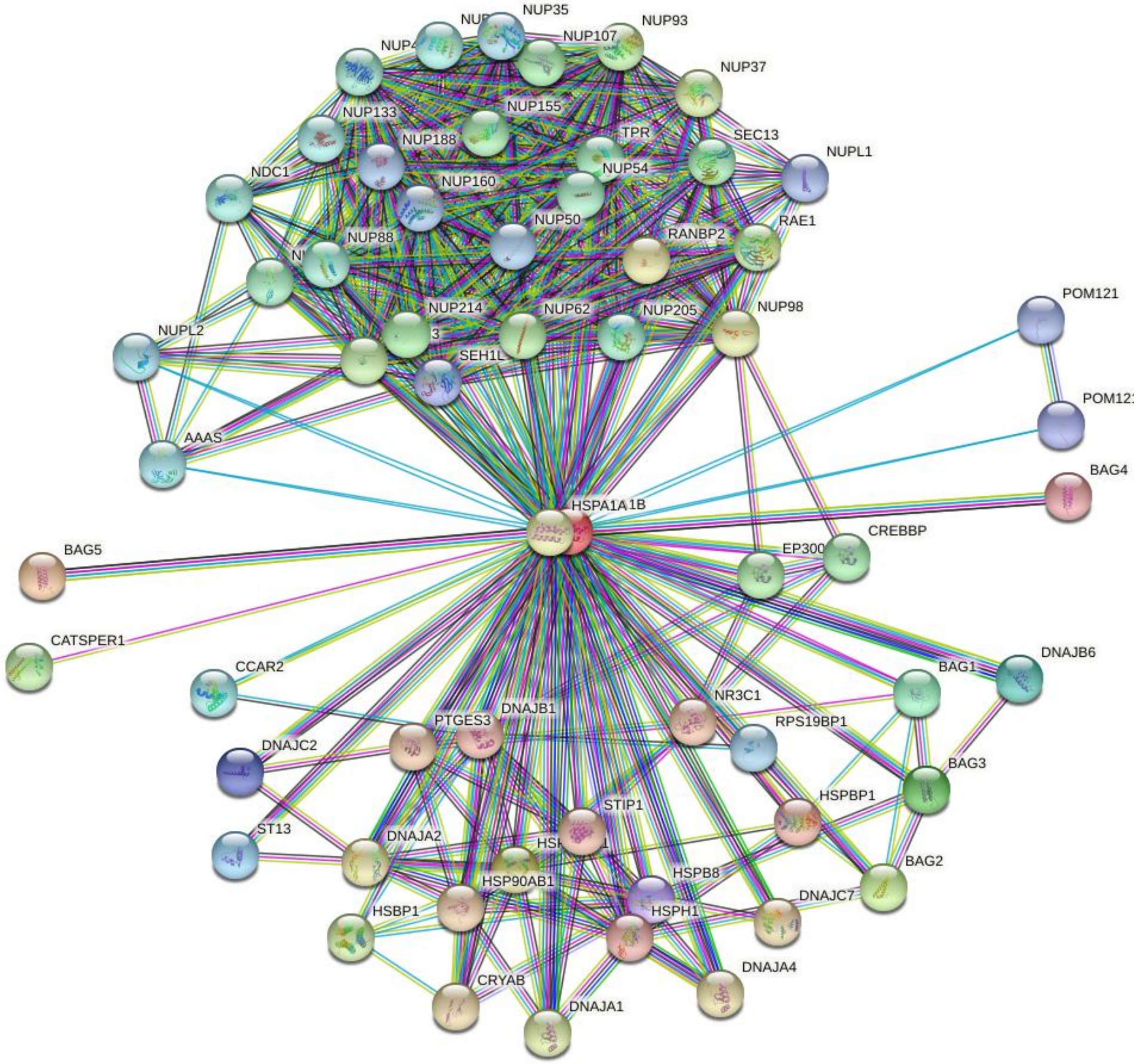

minimum required interaction score: highest confidence (0.900)
number of nodes: 60
number of edges: 459
average node degree: 15.3
avg. local clustering coefficient: 0.849
expected number of edges: 97
PPI enrichment p-value: < 1.0e-16

HSPA1B (UniProt ID: P0DMV9)

>sp|P0DMV9|HS71B_HUMAN Heat shock 70 kDa protein 1B OS=Homo sapiens OX=9606 GN=HSPA1B PE=1 SV=1

MAKAAAIGIDLGTTYSCVGVFQHGKVEIIANDQGNRTTPSYVAFTDTERLIGDAAKNQV
ALNPQNTVFDAKRLIGRKFGDPVVQSDMKHWPFQVINDGDKPKVQVSYKGETKAFYPE
EISSMVLTKMKEIAEAYLGYPVTNAVITVPAYFNDSQRQATKDAGVIAGLNVLRIINEPT
AAAIAYGLDRTGKGERNVLIFDLGGGTFDVSILTIDDGIFEVKATAGDTHLGGEDFDNRL
VNHFVEEFKRKHKKDISQNKRAVRRLRTACERAKRTLSSSTQASLEIDSLFEGIDFYTSIT
RARFEELCSDLFRSTLEPVEKALRDAKLDKAQIHDLVLVGGSTRIPKVQKLLQDFFNGR
DLNKSINPDEAVAYGAAVQAAILMGDKSENVQDLLLLDVAPLSLGLETAGGVMTALIK
RNSTIPTKQTQIFTTYSDNQPGVLIQVYEGERAMTKDNNLLGRFELSGIPPAPRGVPQIEV
TFDIDANGILNVTATDKSTGKANKITITNDKGRLSKEEIERMVQEAEKYKAEDEVQRER
VSAKNALESYAFNMKSAVEDEGLKGKISEADKKKVLDKCQEVISWLDANTLAEKDEFE
HKRKELEQVCNPIISGLYQGAGGPGPGGFGAQGPKGGSGSGPTIEEVD

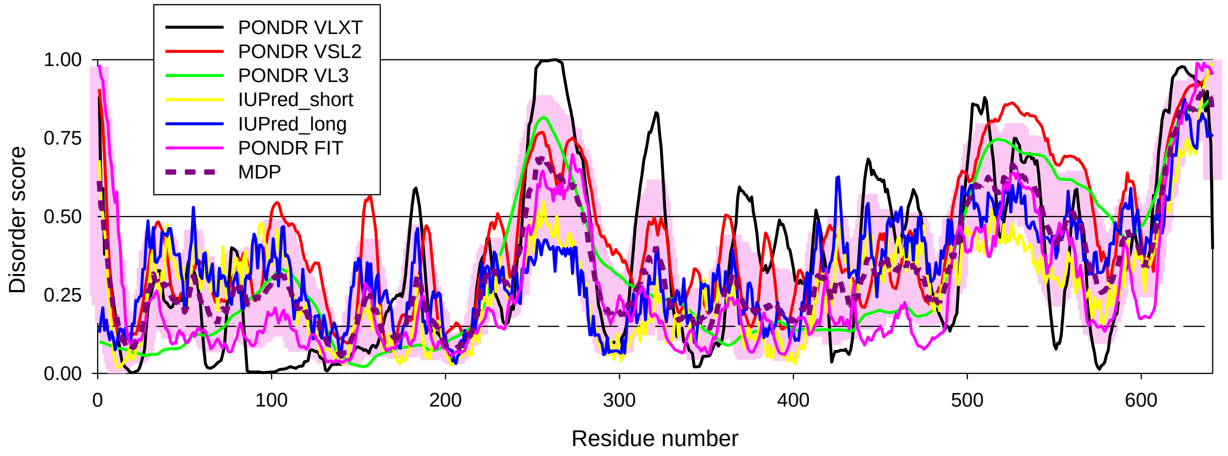

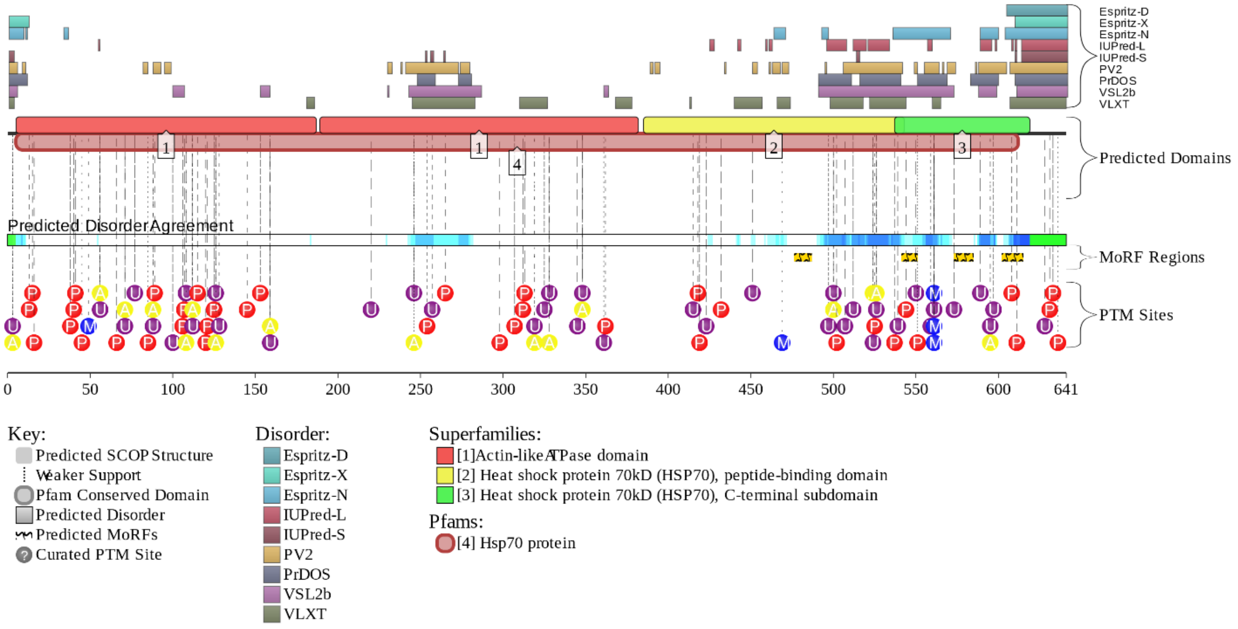

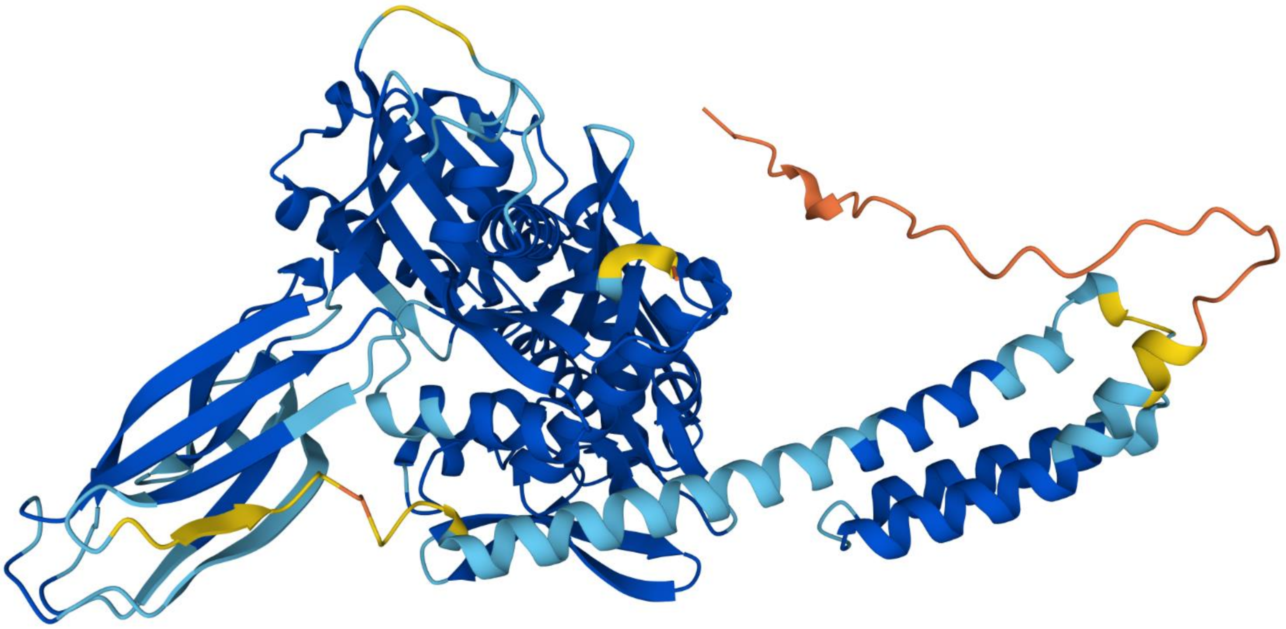

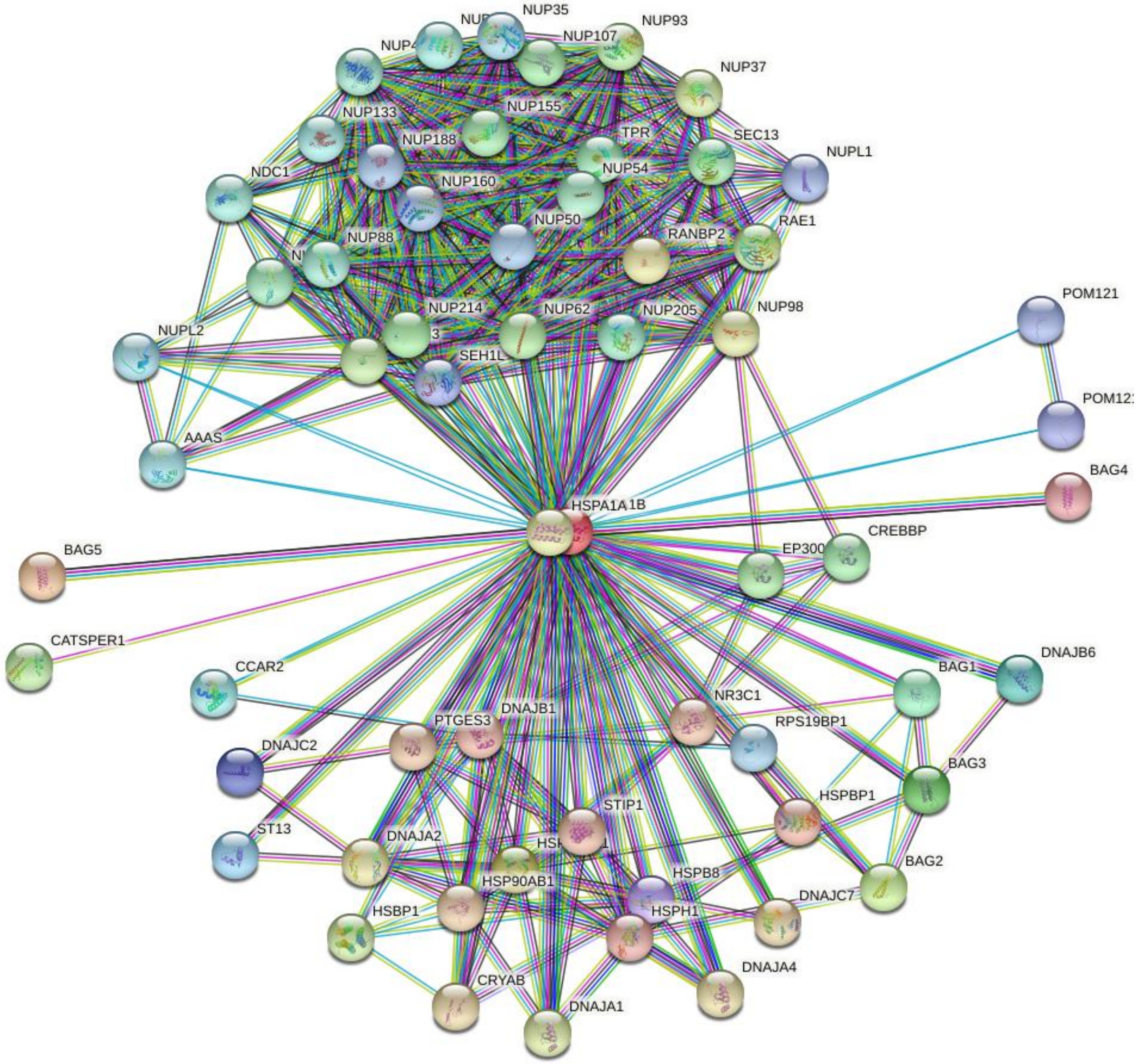

minimum required interaction score: highest confidence (0.900)
number of nodes: 60
number of edges: 459
average node degree: 15.3
avg. local clustering coefficient: 0.849
expected number of edges: 97
PPI enrichment p-value: < 1.0e-16

Prefoldin 1 (PFDN1, UniProt ID: O60925

>sp|O60925|PFD1_HUMAN Prefoldin subunit 1 OS=Homo sapiens OX=9606 GN=PFDN1 PE=1 SV=2

MAAPVDLELKKAFTELQAKVIDTQQKVKLADIQIEQLNRTKKHAHLTDTEIMTLVDETN
MYEGVGRMFILQSKEAIHSQLLEKQKIAEEKIKELEQKKSYLERSVKEAEDNIREMLMA
RRAQ

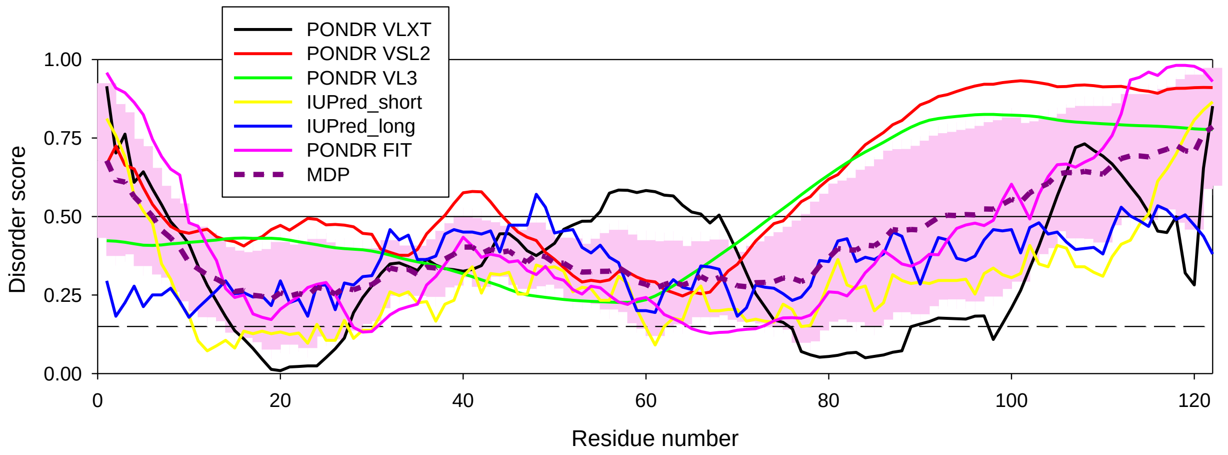

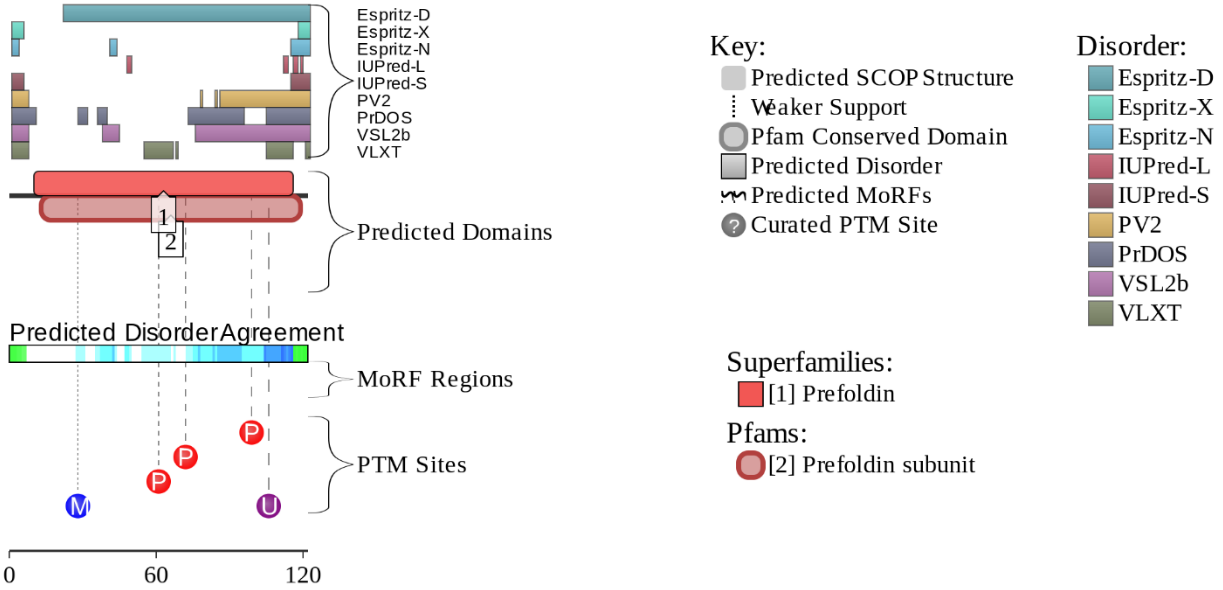

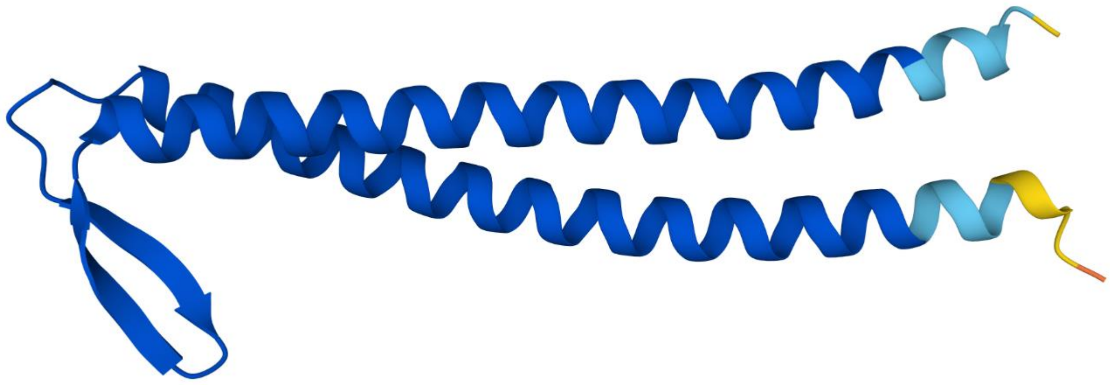

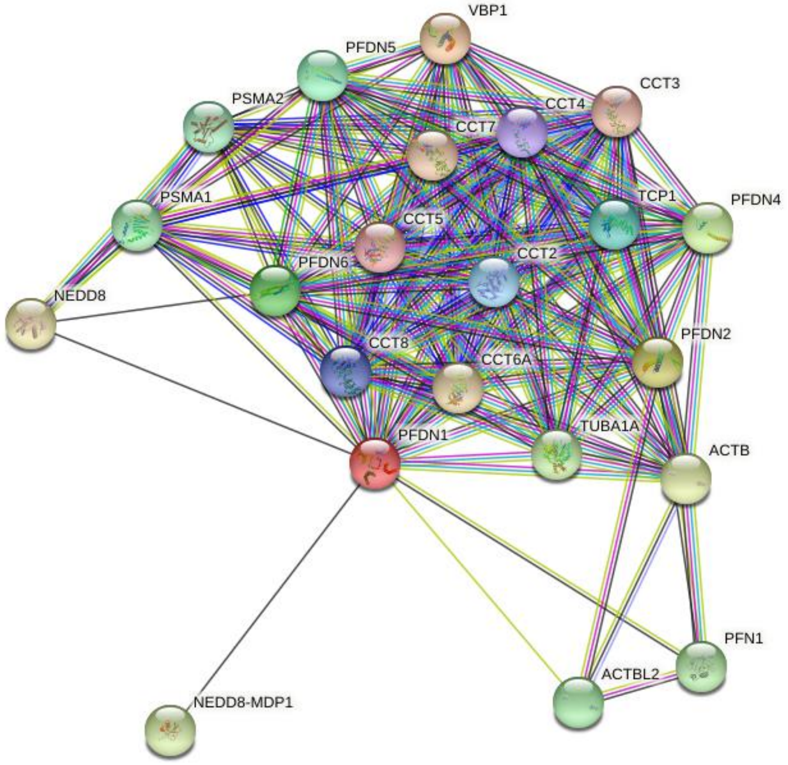

minimum required interaction score: high confidence (0.700)
number of nodes: 22
number of edges: 151
average node degree: 13.7
avg. local clustering coefficient: 0.895
expected number of edges: 26
PPI enrichment p-value: < 1.0e-16

CCTγ (UniProt ID: P49368)

>sp|P49368|TCPG_HUMAN T-complex protein 1 subunit gamma OS=Homo sapiens OX=9606 GN=CCT3 PE=1 SV=4

MMGHRPVLVLSQNTKRESGRKVQSGNINAAKTIADIIRTCLGPKSMMKMLLDPMGGIV
MTNDGNAILREIQVQHPAAKSMIEISRTQDEEVGDGTTSVIILAGEMLSVAEHFLEQQMH
PTVVISAYRKALDDMISTLKKISIPVDISDSDMMLNIINSSITTKAISRWSSLACNIALDAV
KMVQFEENGRKEIDIKKYARVEKIPGGIIEDSCVLRGVMINKDVTHPRMRRYIKNPRIVL
LDSSLEYKKGESQTDIEITREEDFTRILQMEEEYIQQLCEDIIQLKPDVVITEKGISDLAQH
YLMRANITAIRRVRKTDNNRIARACGARIVSRPEELREDDVGTGAGLLEIKKIGDEYFTFI
TDCKDPKACTILLRGASKEILSEVERNLQDAMQVCRNVLLDPQLVPGGGASEMAVAHA
LTEKSKAMTGVEQWPYRAVAQALEVIPRTLIQNCGASTIRLLTSLRAKHTQENCETWGV
NGETGTLVDMKELGIWEPLAVKLQTYKTAVETAVLLLRIDDIVSGHKKKGDDQSRQGG
APDAGQE

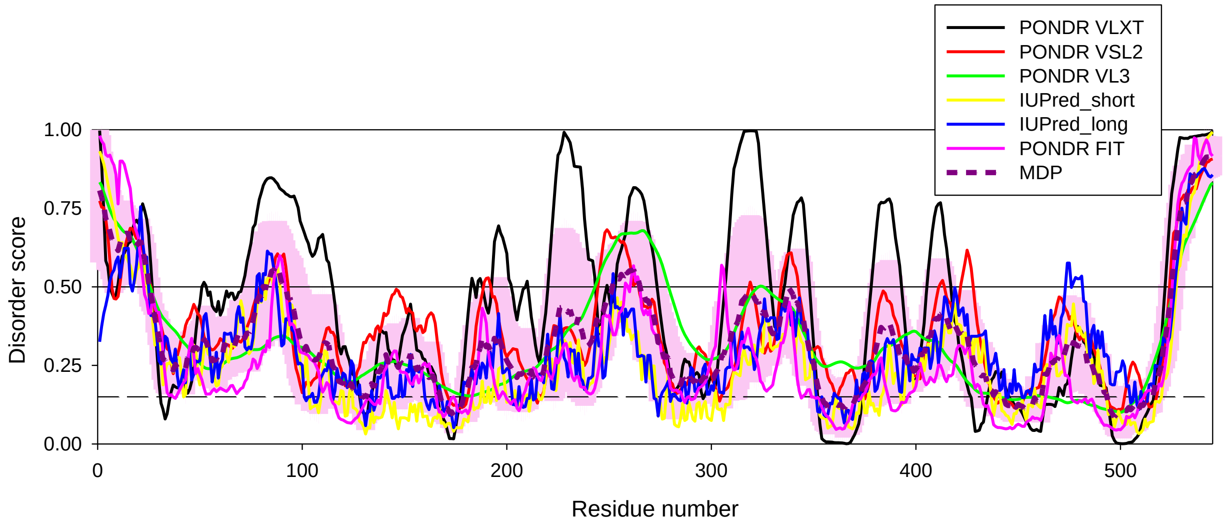

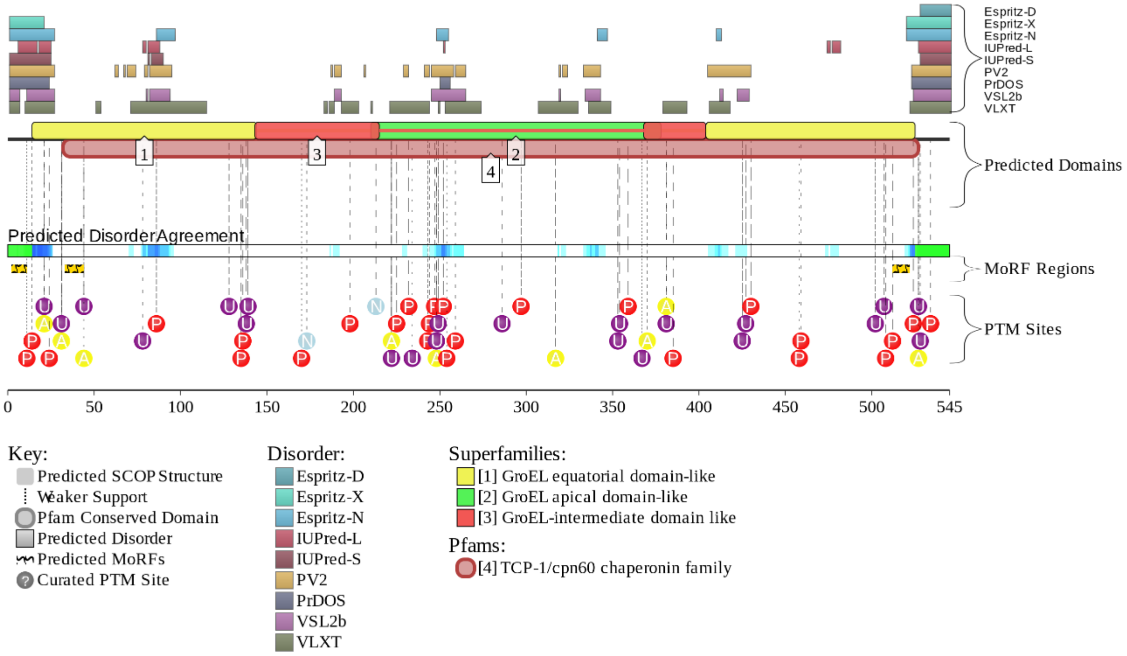
Espritz-D
Espritz-X
Espritz-N
IUPred-L
IUPred-S
PV2
PrDOS
VSL2b
VLXT
Predicted Domains
Predicted Disorder Agreement
MoRF Regions
PTM Sites
0
50
100
150
200
250
300
350
400
450
500
545
Key:
Predicted SCOP Structure
Weaker Support
Pfam Conserved Domain
Predicted Disorder
Predicted MoRFs
Curated PTM Site
Disorder:
Esprtiz-D
Esprtiz-X
Esprtiz-N
IUPred-L
IUPred-S
PV2
PrDOS
VSL2b
VLXT
Superfamilies:
[1] GroEL equatorial domain-like
[2] GroEL apical domain-like
[3] GroEL-intermediate domain like
Pfams:
[4] TCP-1/cpn60 chaperonin family

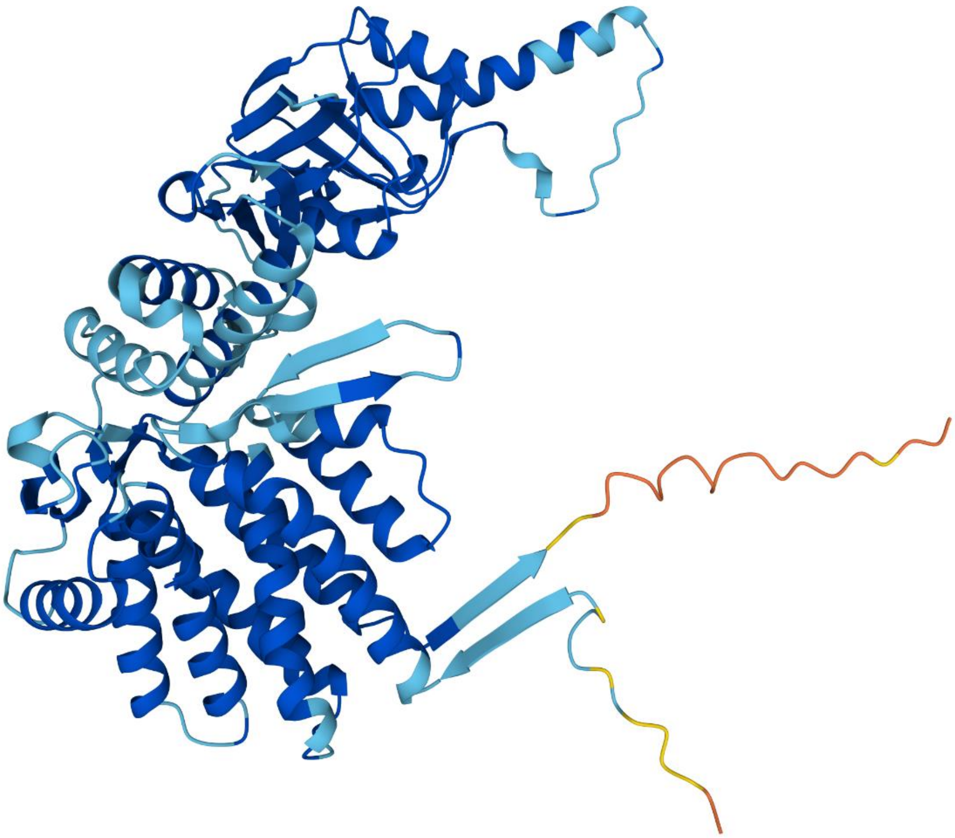

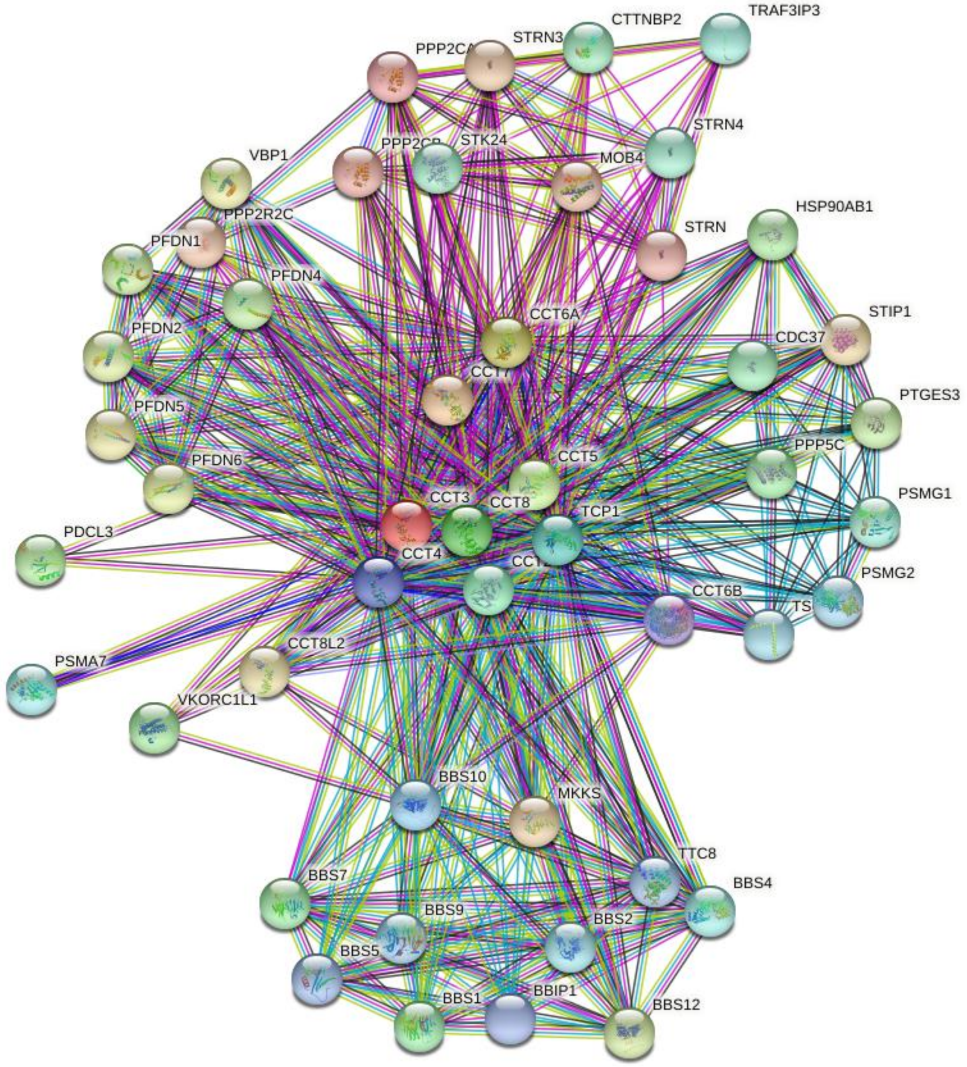

minimum required interaction score: highest confidence (0.900)
number of nodes: 48
number of edges: 443
average node degree: 18.5
avg. local clustering coefficient: 0.872
expected number of edges: 53
PPI enrichment p-value: < 1.0e-16

Supplementary Figure S4. Functional disorder in human proteins interacting with the RABV G-protein. For each protein, an amino acid sequence in FASTA format is shown followed by the disorder profile generated by RIDAO, D[2]P[2]-generated functional disorder profile, modeled 3D structure generated by AlphaFold, and STRING-based protein-protein interaction network.

nAChR α1 (CHRNA1; UniProt ID: P02708)

>sp|P02708|ACHA_HUMAN Acetylcholine receptor subunit alpha OS=Homo sapiens OX=9606 GN=CHRNA1 PE=1 SV=3

MEPWPLLLLFSLCSAGLVLGSEHETRLVAKLFKDYSSVVRPVEDHRQVVEVTVGLQLIQ
LINVDEVNQIVTTNVRLKQQWVDYNLKWNPDDYGGVKKIHIPSEKIWRPDLVLYNNAD
GDFAIVKFTKVLLQYTGHITWTPPAIFKSYCEIIVTHFPFDEQNCSMKLGTWTYDGSVVA
INPESDQPDLSNFMESGEWVIKESRGWKHSVTYSCCPDTPYLDITYHFVMQRLPLYFIVN
VIIPCLLFSFLTGLVFYLPTDSGEKMTLSISVLLSLTVFLLVIVELIPSTSSAVPLIGKYMLFT
MVFVIASIIITVIVINTHHRSPSTHVMPNWVRKVFIDTIPNIMFFSTMKRPSREKQDKKIFT
EDIDISDISGKPGPPPMGFHSPLIKHPEVKSAIEGIKYIAETMKSDQESNNAAAEWKYVA
MVMDHILLGVFMLVCIIGTLAVFAGRLIELNQQG

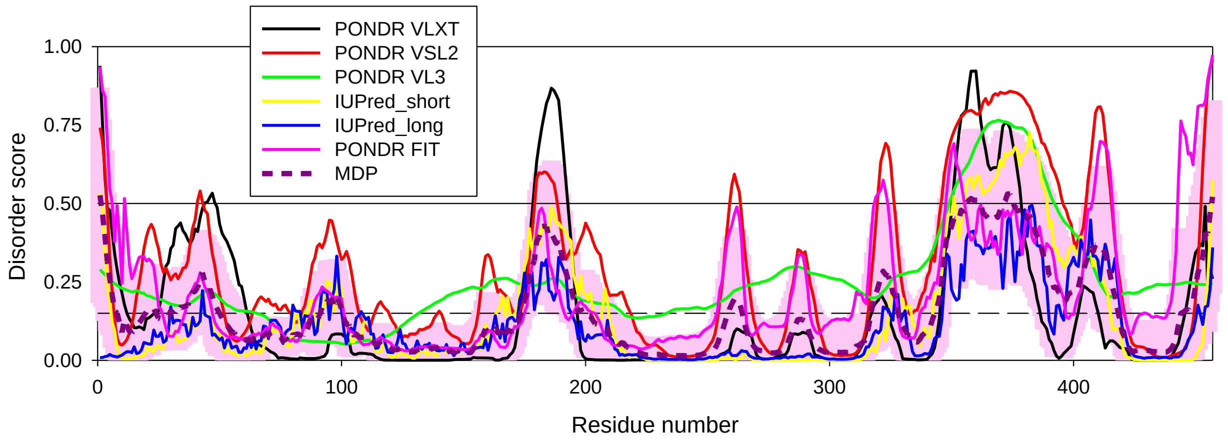

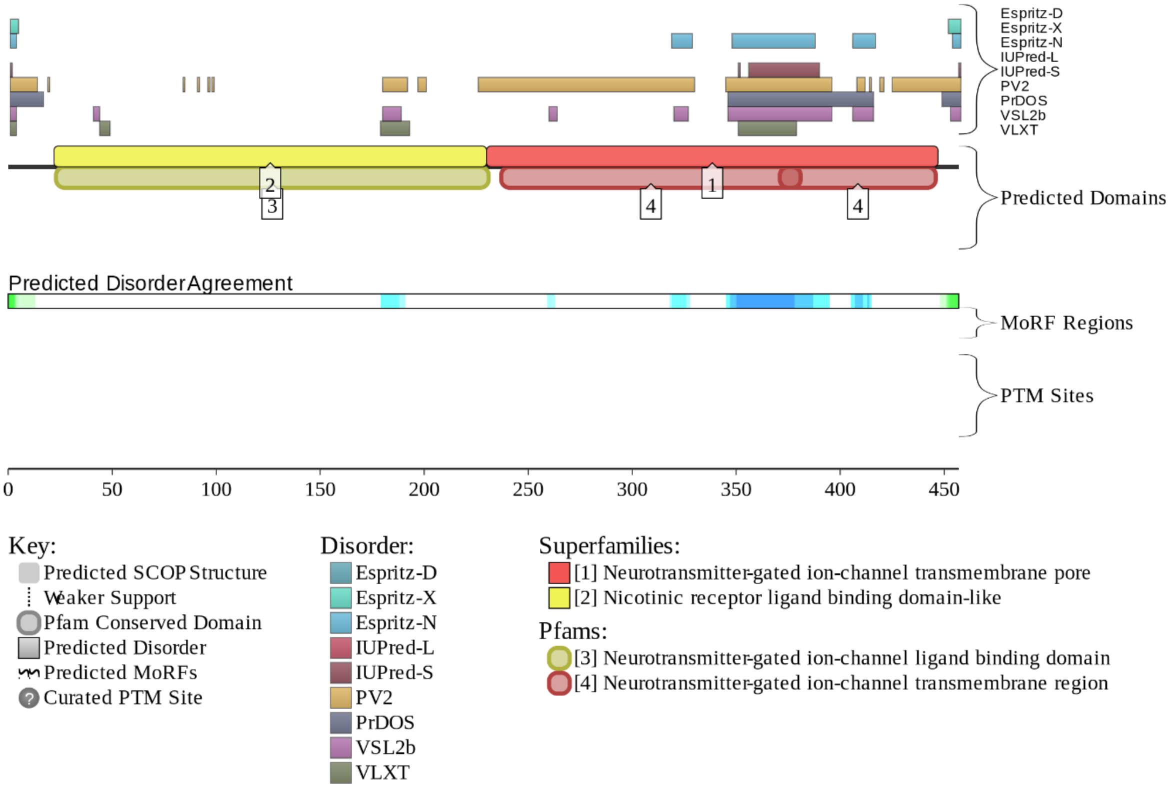
Espritz-D
Espritz-X
Espritz-N
IUPred-L
IUPred-S
PV2
PrDOS
VSL2b
VLXT
Predicted Domains
Predicted Disorder Agreement
MoRF Regions
PTM Sites
0
50
100
150
200
250
300
350
400
450
Key:
Predicted SCOP Structure
Weaker Support
Pfam Conserved Domain
Predicted Disorder
Predicted MoRFs
Curated PTM Site
Disorder:
Espritz-D
Espritz-X
Espritz-N
IUPred-L
IUPred-S
PV2
PrDOS
VSL2b
VLXT
Superfamilies:
[1] Neurotransmitter-gated ion-channel transmembrane pore
[2] Nicotinic receptor ligand binding domain-like
Pfams:
[3] Neurotransmitter-gated ion-channel ligand binding domain
[4] Neurotransmitter-gated ion-channel transmembrane region

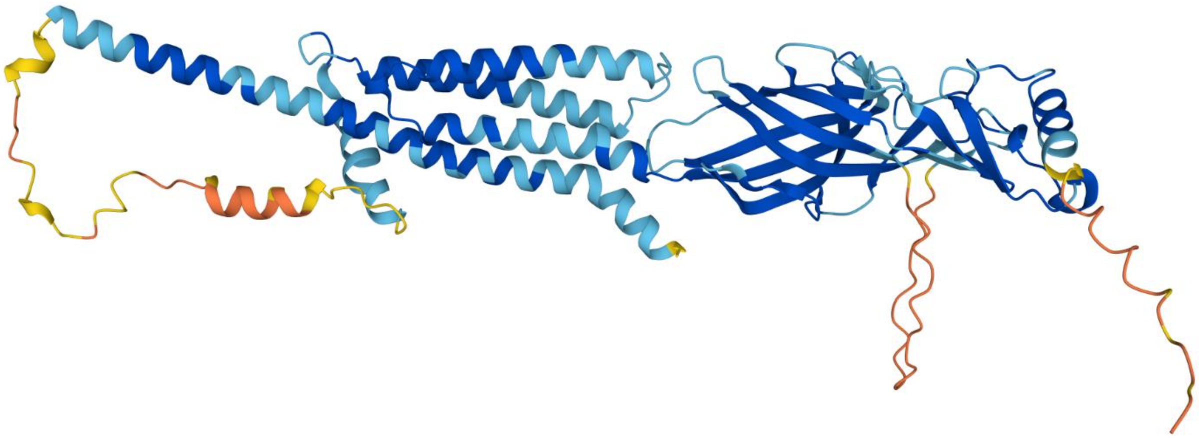

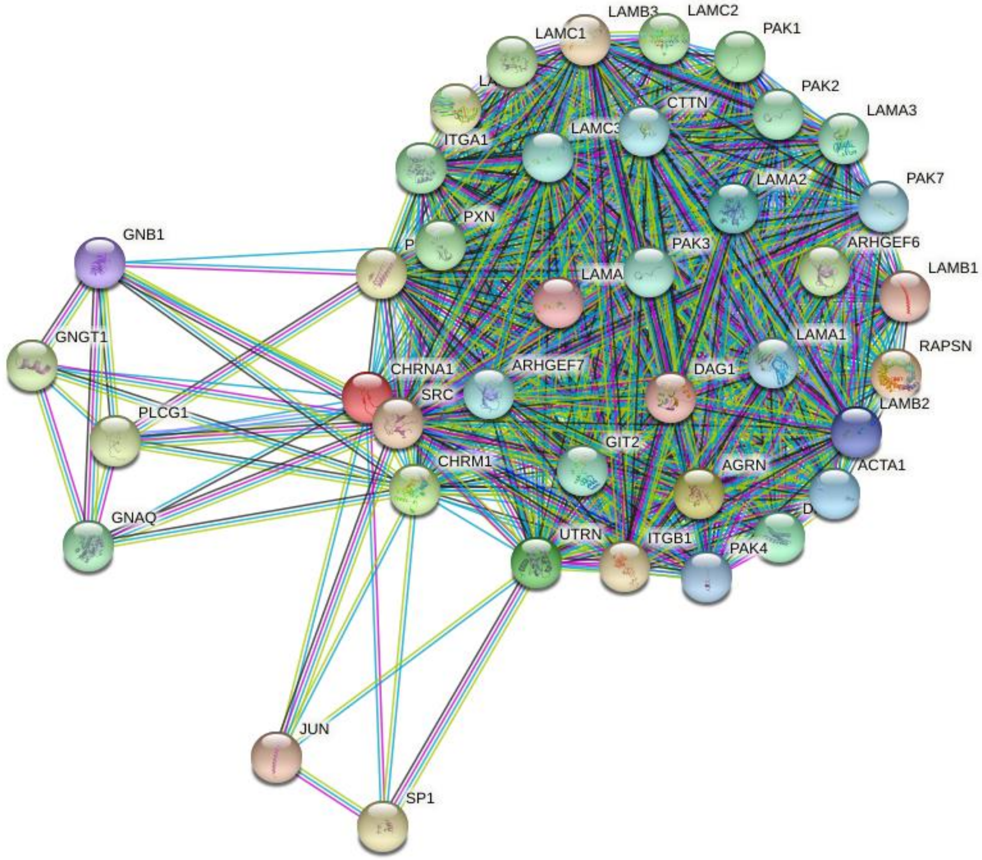

minimum required interaction score: highest confidence (0.900)
number of nodes: 39
number of edges: 558
average node degree: 28.6
avg. local clustering coefficient: 0.961
expected number of edges: 62
PPI enrichment p-value: < 1.0e-16

nAChr α7 (CHRNA7, UniProt ID: P36544)

>sp|P36544|ACHA7_HUMAN Neuronal acetylcholine receptor subunit alpha-7 OS=Homo sapiens OX=9606 GN=CHRNA7 PE=1 SV=5

MRCSPGGVWLALAASLLHVSLQGEFQRKLYKELVKNYNPLERPVANDSQPLTVYFSLS
LLQIMDVDEKNQVLTTNIWLQMSWTDHYLQWNVSEYPGVKTVRFPDGQIWKPDILLY
NSADERFDATFHTNVLVNSSGHCQYLPPGIFKSSCYIDVRWFPFDVQHCKLKFGSWSYG
GWSLDLQMQEADISGYIPNGEWDLVGIPGKRSERFYECCKEPYPDVTFTVTMRRRTLYY
GLNLLIPCVLISALALLVFLLPADSGEKISLGITVLLSLTVFMLLVAEIMPATSDSVPLIAQ
YFASTMIIVGLSVVVTVIVLQYHHHDPDGGKMPKWTRVILLNWCAWFLRMKRPGEDK
VRPACQHKQRRCSLASVEMSAVAPPPASNGNLLYIGFRGLDGVHCVPTPDSGVVCGRM
ACSPTHDEHLLHGGQPPEGDPDLAKILEEVRYIANRFRCQDESEAVCSEWKFAACVVDR
LCLMAFSVFTIICTIGILMSAPNFVEAVSKDFA

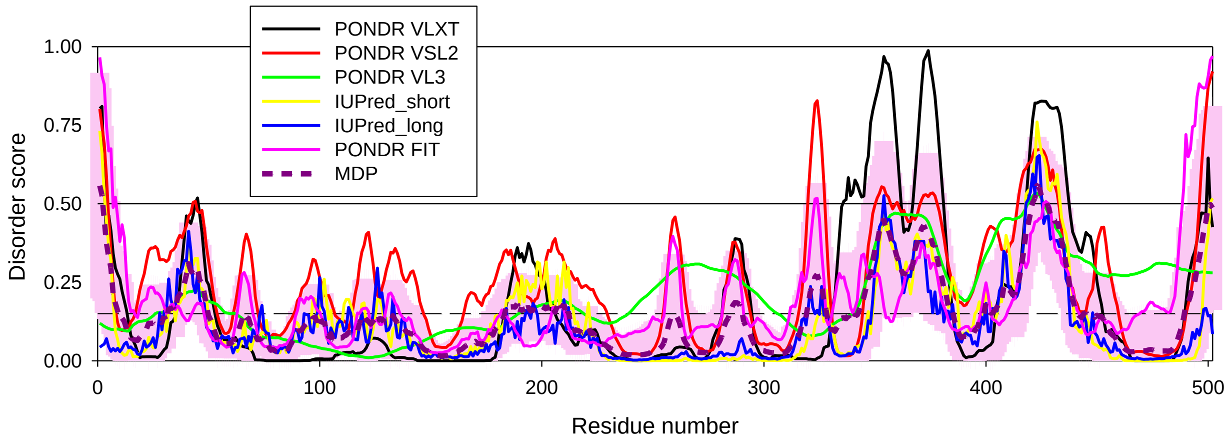

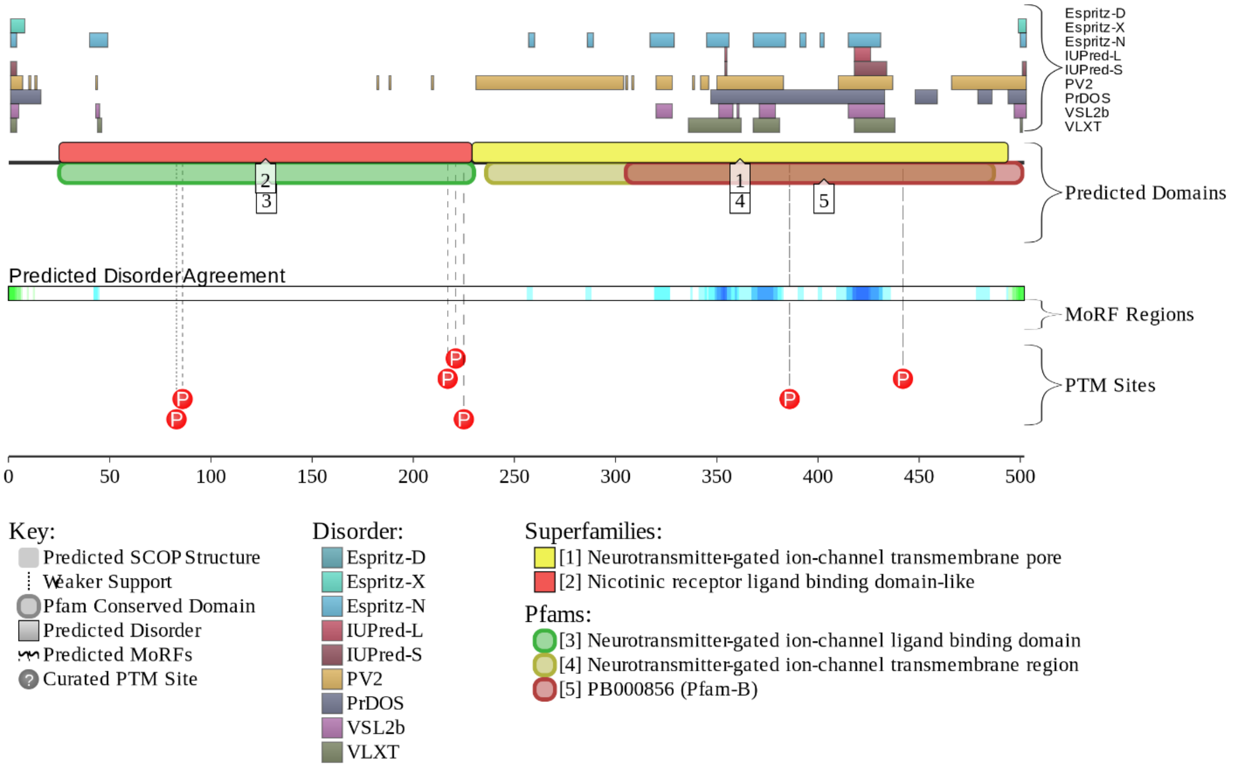
Espritz-D
Espritz-X
Espritz-N
IUPred-L
IUPred-S
PV2
PrDOS
VSL2b
VLXT
Predicted Domains
Predicted Disorder Agreement
MoRF Regions
PTM Sites
0
50
100
150
200
250
300
350
400
450
500
Key:
Predicted SCOP Structure
Weaker Support
Pfam Conserved Domain
Predicted Disorder
Predicted MoRFs
Curated PTM Site
Disorder:
Espritz-D
Espritz-X
Espritz-N
IUPred-L
IUPred-S
PV2
PrDOS
VSL2b
VLXT
Superfamilies:
[1] Neurotransmitter-gated ion-channel transmembrane pore
[2] Nicotinic receptor ligand binding domain-like
Pfams:
[3] Neurotransmitter-gated ion-channel ligand binding domain
[4] Neurotransmitter-gated ion-channel transmembrane region
[5] PB000856 (Pfam-B)

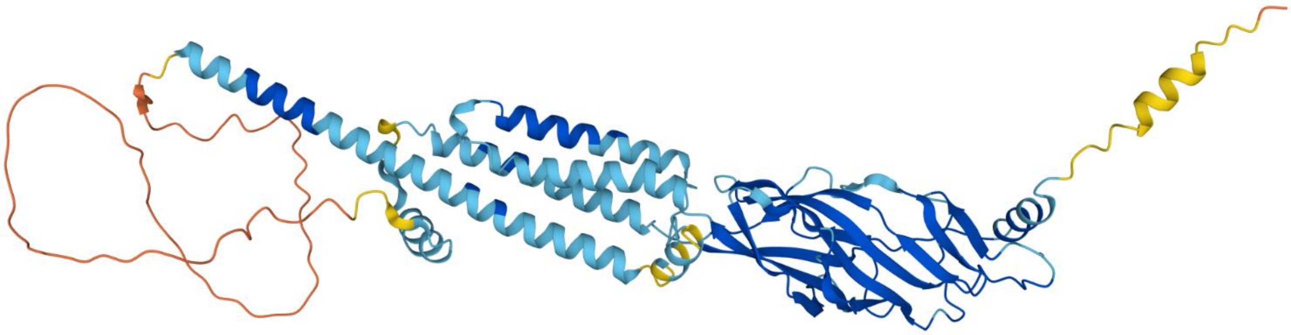

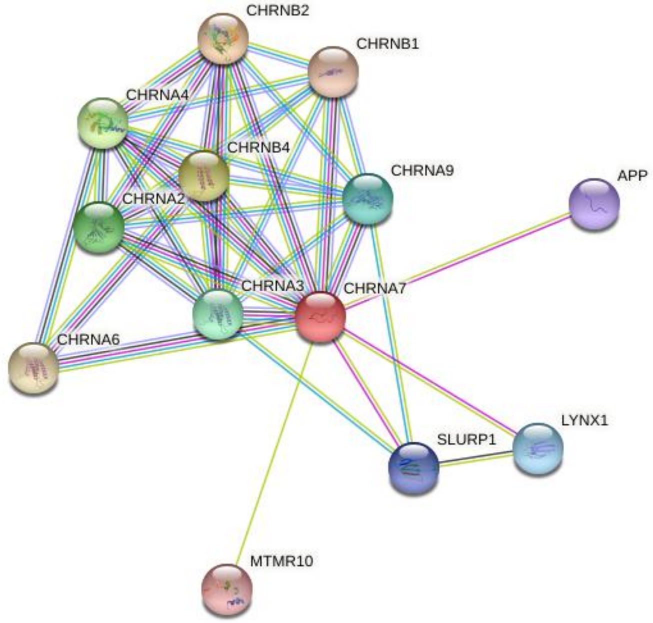

minimum required interaction score: high confidence (0.700)
number of nodes: 13
number of edges: 38
average node degree: 5.85
avg. local clustering coefficient: 0.873
expected number of edges: 12
PPI enrichment p-value: 2.01e-09

NCAM1 (UniProt ID: P13591)

>sp|P13591|NCAM1_HUMAN Neural cell adhesion molecule 1 OS=Homo sapiens OX=9606 GN=NCAM1 PE=1 SV=3

MLQTKDLIWTLFFLGTAVSLQVDIVPSQGEISVGESKFFLCQVAGDAKDKDISWFSPNGE
KLTPNQQRISVVWNDDSSSTLTIYNANIDDAGIYKCVVTGEDGSESEATVNVKIFQKLMF
KNAPTPQEFREGEDAVIVCDVVSSLPPTIIWKHKGRDVILKKDVRFIVLSNNYLQIRGIKK
TDEGTYRCEGRILARGEINFKDIQVIVNVPPTIQARQNIVNATANLGQSVTLVCDAEGFPE
PTMSWTKDGEQIEQEEDDEKYIFSDDSSQLTIKKVDKNDEAEYICIAENKAGEQDATIHL
KVFAKPKITYVENQTAMELEEQVTLTCEASGDPIPSITWRTSTRNISSEEKASWTRPEKQE
TLDGHMVVRSHARVSSLTLKSIQYTDAGEYICTASNTIGQDSQSMYLEVQYAPKLQGPV
AVYTWEGNQVNITCEVFAYPSATISWFRDGQLLPSSNYSNIKIYNTPSASYLEVTPDSEN
DFGNYNCTAVNRIGQESLEFILVQADTPSSPSIDQVEPYSSTAQVQFDEPEATGGVPILKY
KAEWRAVGEEVWHSKWYDAKEASMEGIVTIVGLKPETTYAVRLAALNGKGLGEISAA
SEFKTQPVQGEPSAPKLEGQMGEDGNSIKVNLIKQDDGGSPIRHYLVRYRALSSEWKPEI
RLPSGSDHVMLKSLDWNAEYEVYVVAENQQGKSKAAHFVFRTSAQPTAIPANGSPTSG
LSTGAIVGILIVIFVLLLVVVDITCYFLNKCGLFMCIAVNLCGKAGPGAKGKDMEEGKAA
FSKDESKEPIVEVRTEEERTPNHDGGKHTEPNETTPLTEPEKGPVEAKPECQETETKPAPA
EVKTVPNDATQTKENESKA

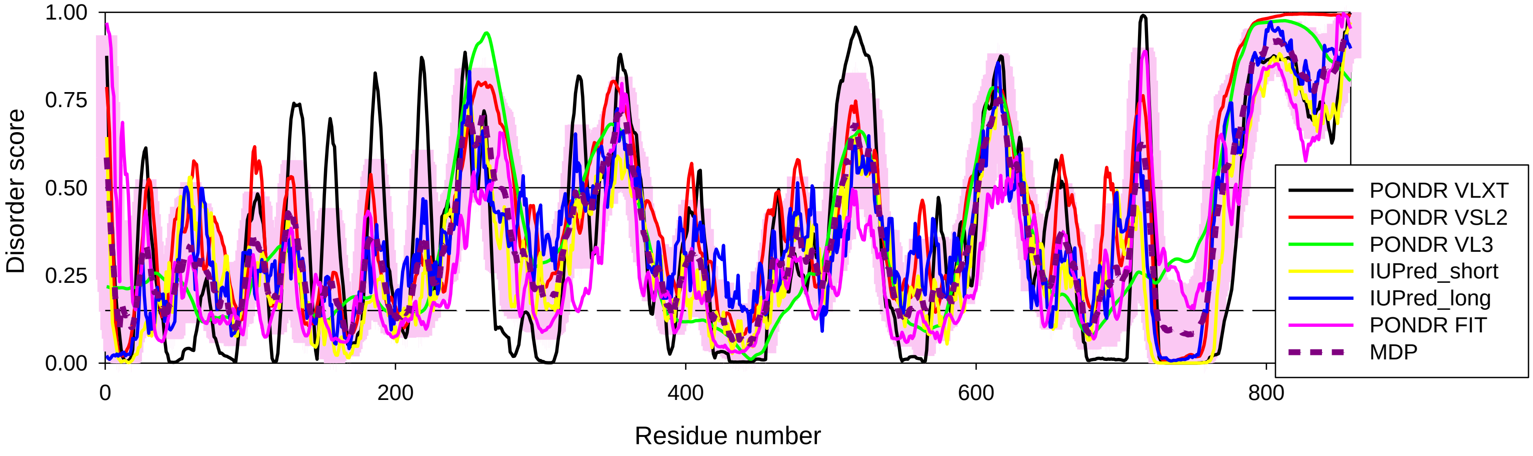

Note: D^2P^2 profile for human NCAM1 is not available.

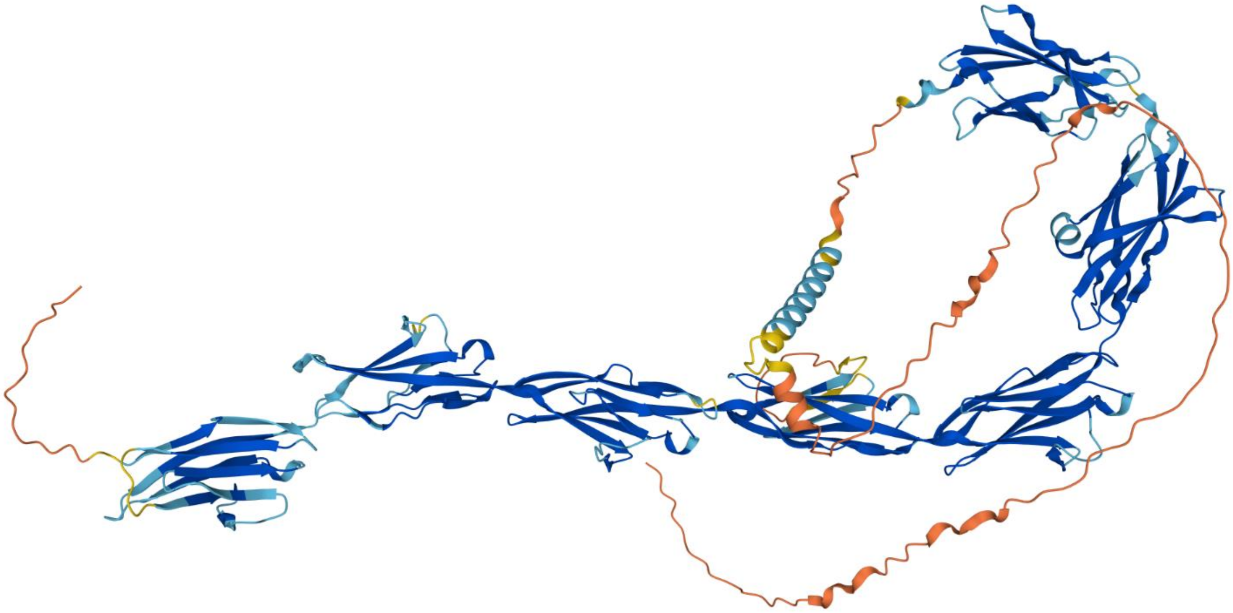

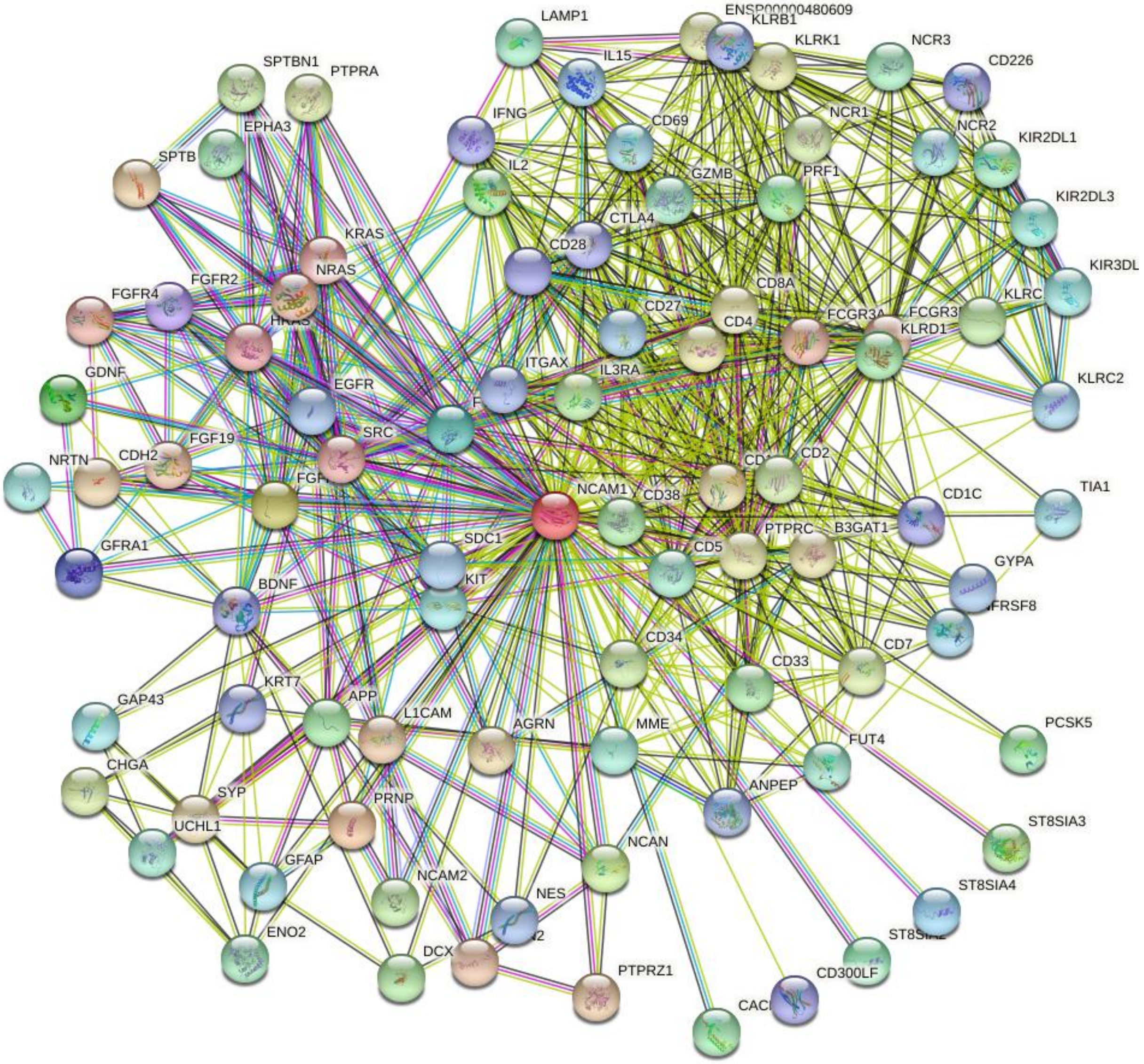

minimum required interaction score: highest confidence (0.900)
number of nodes: 90
number of edges: 673
average node degree: 15
avg. local clustering coefficient: 0.691
expected number of edges: 145
PPI enrichment p-value: < 1.0e-16

p75NTR (UniProt ID: P08138)

>sp|P08138|TNR16_HUMAN Tumor necrosis factor receptor superfamily member 16 OS=Homo sapiens OX=9606 GN=NGFR PE=1 SV=1

MGAGATGRAMDGPRLLLLLLLLGVSLGGAKEACPTGLYTHSGECCKACNLGEGVAQPC
GANQTVCEPCLDSVTFSDVVSATEPCKPCTECVGLQSMSAPCVEADDAVCRCAYGYYQ
DETTGRCEACRVCEAGSGLVFSCQDKQNTVCEECPDGTYSDEANHVDPCLPCTVCEDT
ERQLRECTRWADAECEEIPGRWITRSTPPEGSDSTAPSTQEPEAPPEQDLIASTVAGVVTT
VMGSSQPVVTRGTTDNLIPVYCSILAAVVVGLVAYIAFKRWNSCKQNKQGANSRPVNQ
TPPPEGEKLHSDSGISVDSQSLHDQQPHTQTASGQALKGDGGLYSSLPPAKREEVEKLLN
GSAGDTWRHLAGELGYQPEHIDSFTHEACPVRALLASWATQDSATLDALLAALRRIQR
ADLVESLCSESTATSPV

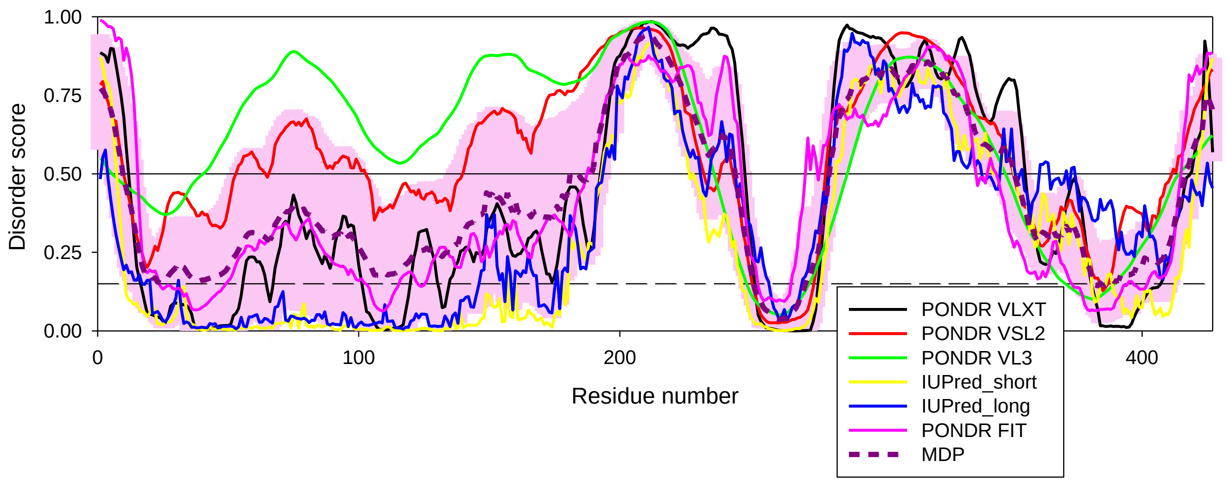

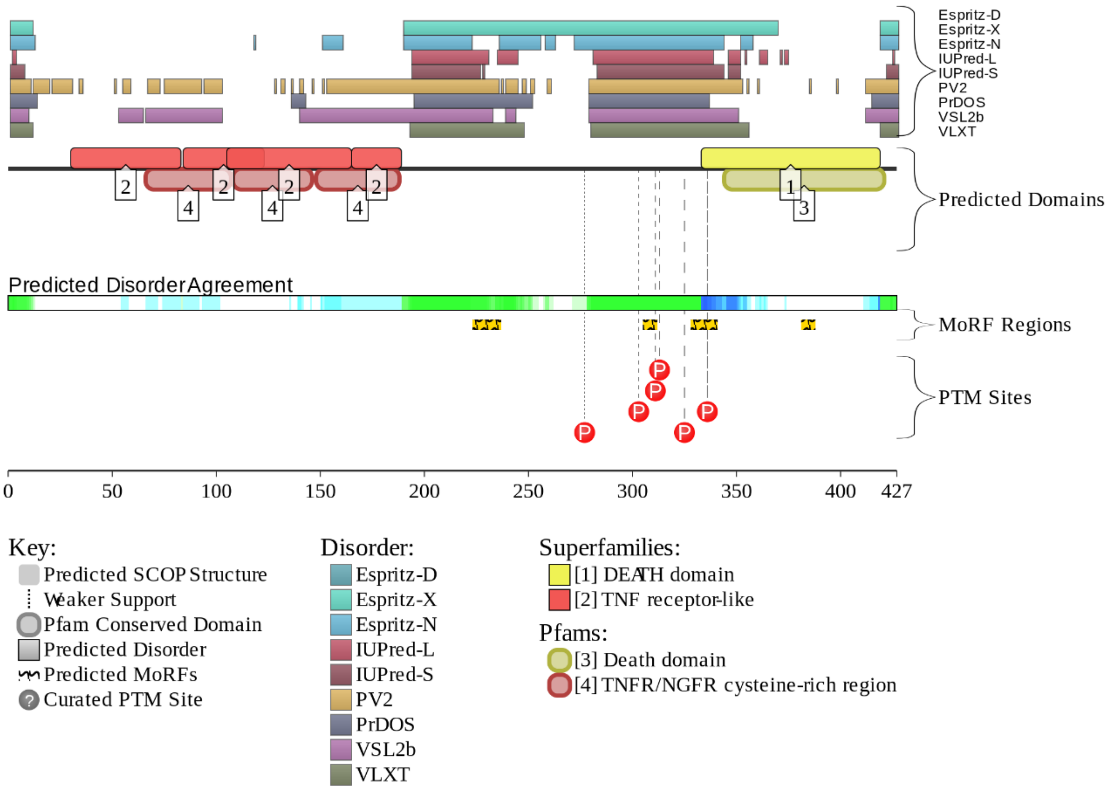
Espritz-D
Espritz-X
Espritz-N
IUPred-L
IUPred-S
PV2
PrDOS
VSL2b
VLXT
Predicted Domains
Predicted Disorder Agreement
MoRF Regions
PTM Sites
0
50
100
150
200
250
300
350
400
427
Key:
Predicted SCOP Structure
Weaker Support
Pfam Conserved Domain
Predicted Disorder
Predicted MoRFs
Curated PTM Site
Disorder:
Espritz-D
Espritz-X
Espritz-N
IUPred-L
IUPred-S
PV2
PrDOS
VSL2b
VLXT
Superfamilies:
[1] DEATH domain
[2] TNF receptor-like
Pfams:
[3] Death domain
[4] TNFR/NGFR cysteine-rich region

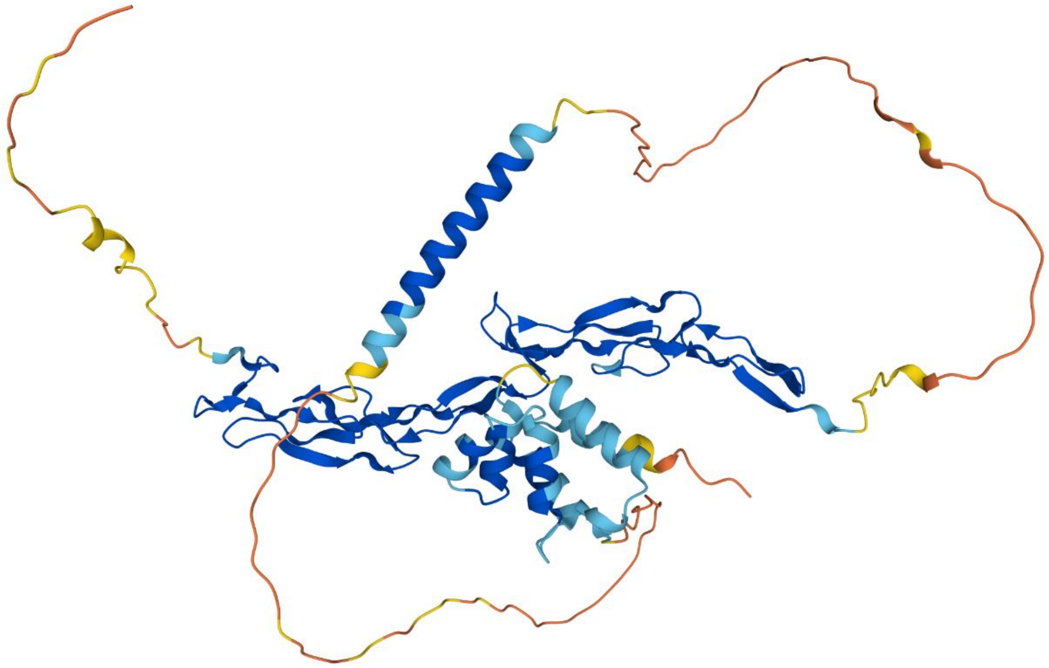

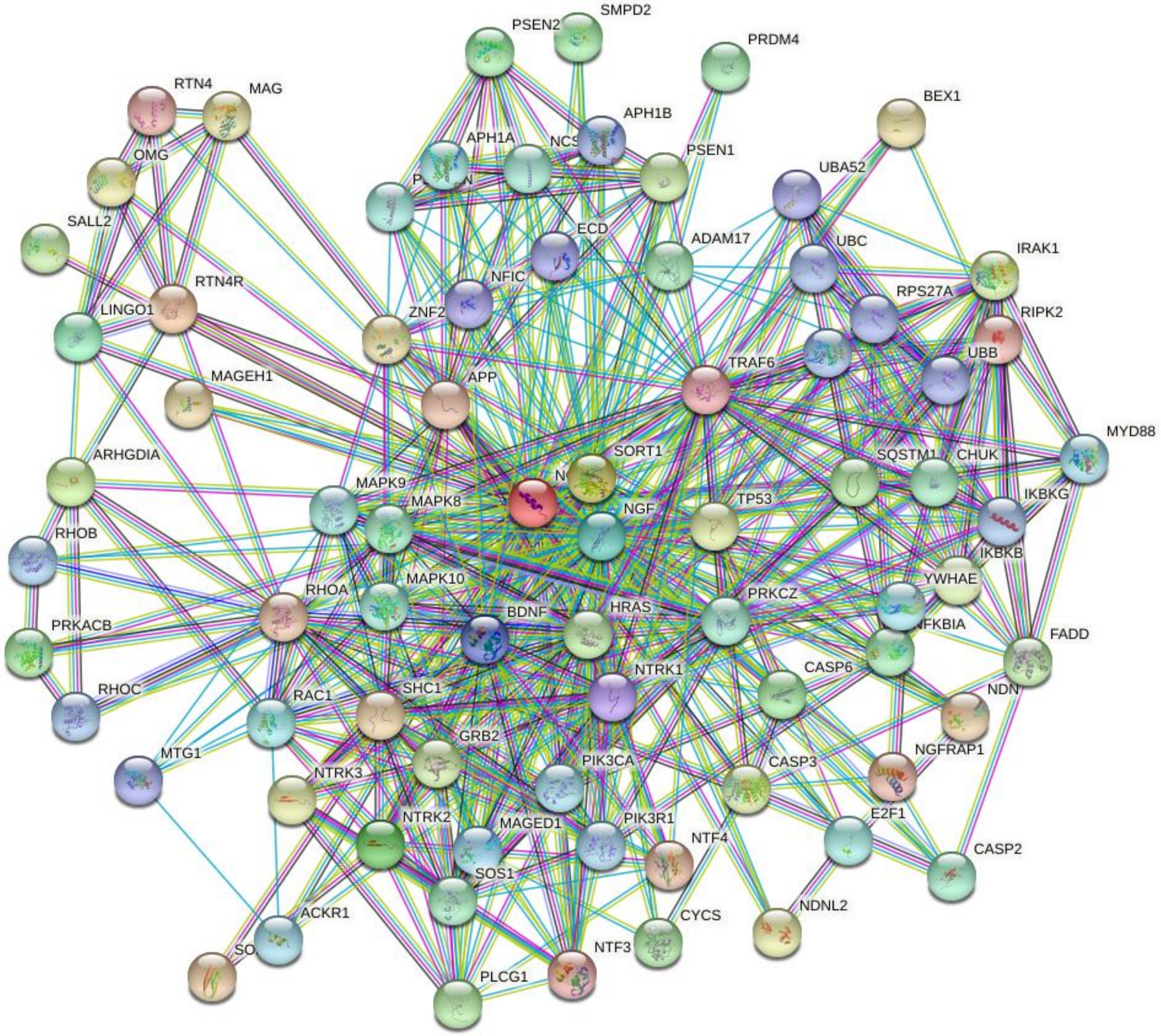

minimum required interaction score: highest confidence (0.900)
number of nodes: 76
number of edges: 498
average node degree: 13.1
avg. local clustering coefficient: 0.731
expected number of edges: 188
PPI enrichment p-value: < 1.0e-16

mGluR2 (UniProt ID: Q14416)

>sp|Q14416|GRM2_HUMAN Metabotropic glutamate receptor 2 OS=Homo sapiens OX=9606 GN=GRM2 PE=1 SV=2

MGSLLALLALLLLWGAVAEGPAKKVLTLEGDLVLGGLFPVHQKGGPAEDCGPVNEHR
GIQRLEAMLFALDRINRDPHLLPGVRLGAHILDSCSKDTHALEQALDFVRASLSRGADG
SRHICPDGSYATHGDAPTAITGVIGGSYSDVSIQVANLLRLFQIPQISYASTSAKLSDKSR
YDYFARTVPPDFFQAKAMAEILRFFNWTYVSTVASEGDYGETGIEAFELEARARNICVA
TSEKVGRAMSRAAFEGVVRALLQKPSARVAVLFTRSEDARELLAASQRLNASFTWVAS
DGWGALESVVAGSEGAAEGAITIELASYPISDFASYFQSLDPWNNSRNPWFREFWEQRF
RCSFRQRDCAAHSLRAVPFEQESKIMFVVNAVYAMAHALHNMHRALCPNTTRLCDAM
RPVNGRRLYKDFVLNVKFDAPFRPADTHNEVRFDRFGDGIGRYNIFTYLRAGSGRYRYQ
KVGYWAEGLTLDTSLIPWASPSAGPLPASRCSEPCLQNEVKSVQPGEVCCWLCIPCQPY
EYRLDEFTCADCGLGYWPNASLTGCFELPQEYIRWGDAWAVGPVTIACLGALATLFVL
GVFVRHNATPVVKASGRELCYILLGGVFLCYCMTFIFIAKPSTAVCTLRRLGLGTAFSVC
YSALLTKTNRIARIFGGAREGAQRPRFISPASQVAICLALISGQLLIVVAWLVVEAPGTGK
ETAPERREVVTLRCNHRDASMLGSLAYNVLLIALCTLYAFKTRKCPENFNEAKFIGFTM
YTTCIIWLAFLPIFYVTSSDYRVQTTTMCVSVSLSGSVVLGCLFAPKLHIILFQPQKNVVS
HRAPTSRFGSAAARASSSLGQGSGSQFVPTVCNGREVVDSTTSSL

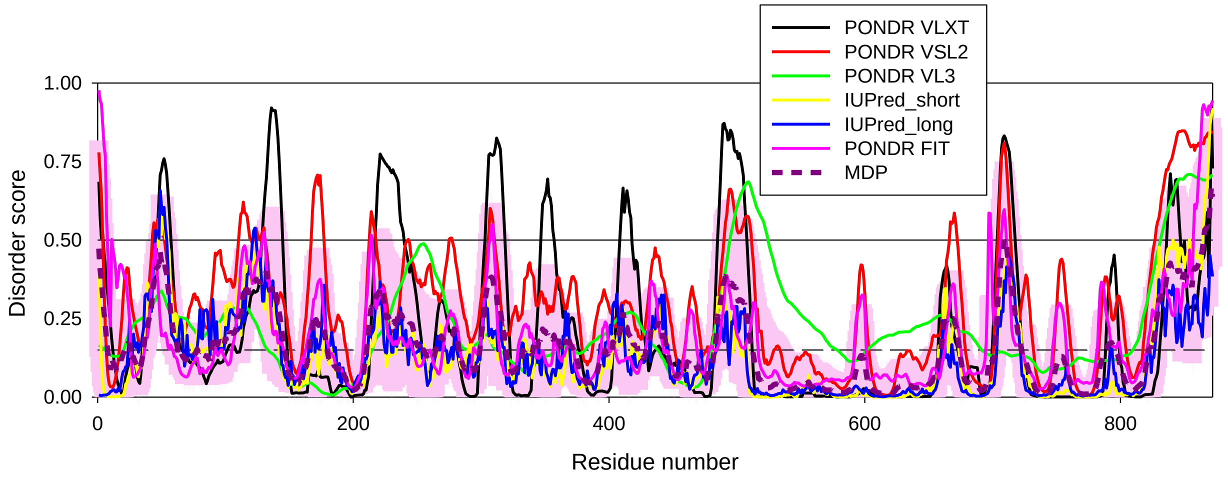

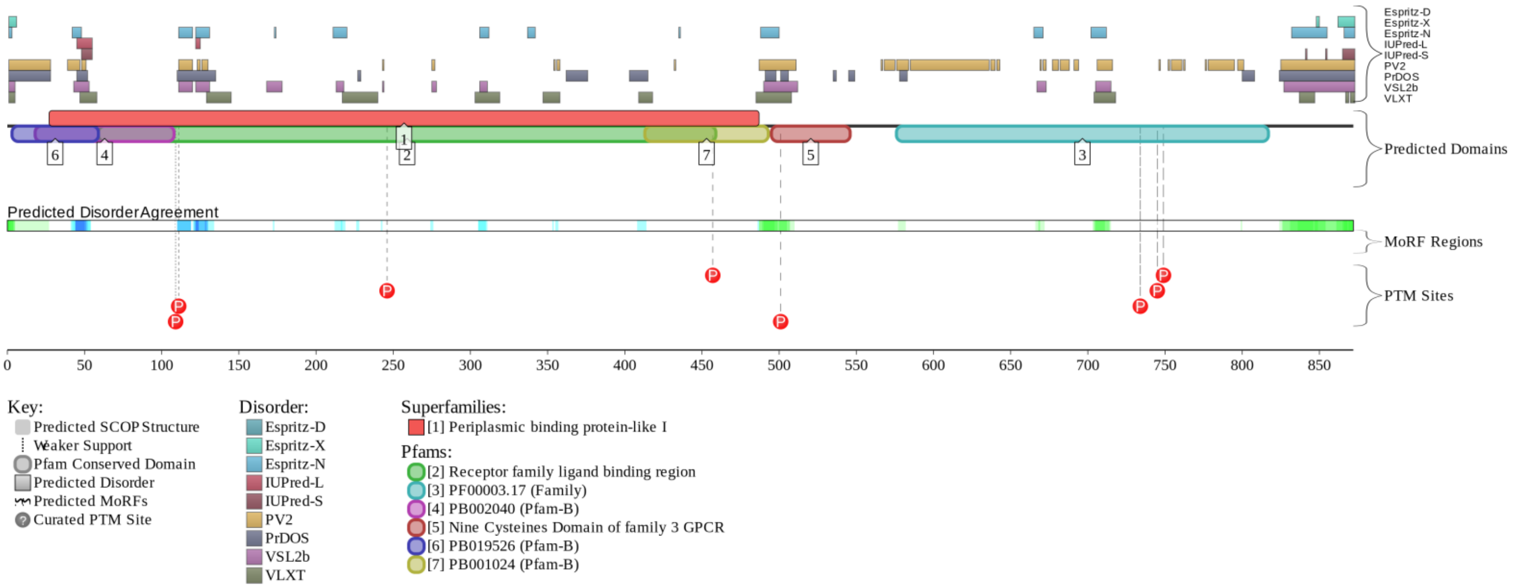
Esprtiz-D
Espritz-X
Espritz-N
IUPred-L
IUPred-S
PV2
PrDOS
VSL2b
VLXT
Predicted Domains
Predicted Disorder Agreement
MoRF Regions
PTM Sites
0 50 100 150 200 250 300 350 400 450 500 550 600 650 700 750 800 850
Key:
Predicted SCOP Structure
Weaker Support
Pfam Conserved Domain
Predicted Disorder
Predicted MoRFs
Curated PTM Site
Disorder:
Espritz-D
Espritz-X
Espritz-N
IUPred-L
IUPred-S
PV2
PrDOS
VSL2b
VLXT
Superfamilies:
[1] Periplasmic binding protein-like I
Pfams:
[2] Receptor family ligand binding region
[3] PF00003.17 (Family)
[4] PB002040 (Pfam-B)
[5] Nine Cysteines Domain of family 3 GPCR
[6] PB019526 (Pfam-B)
[7] PB001024 (Pfam-B)

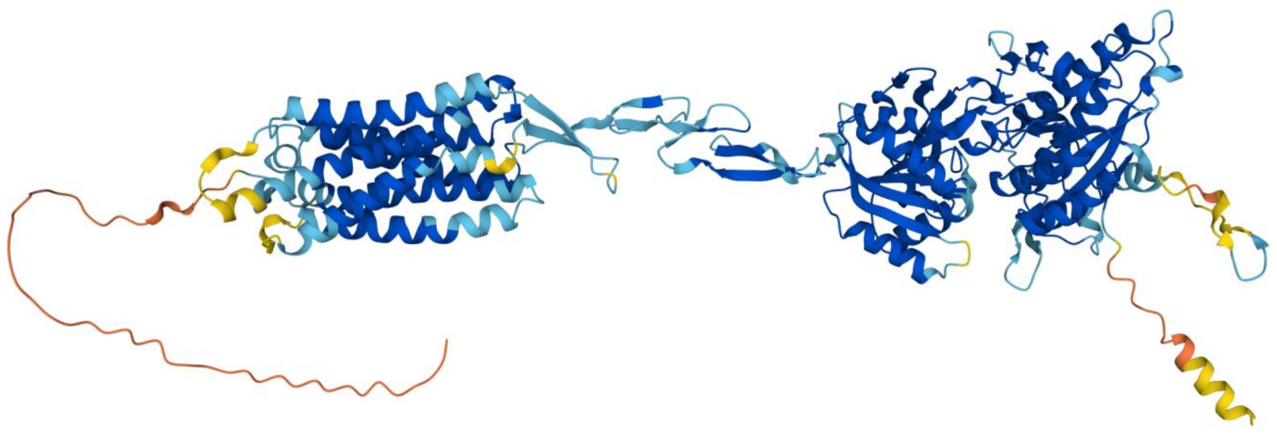

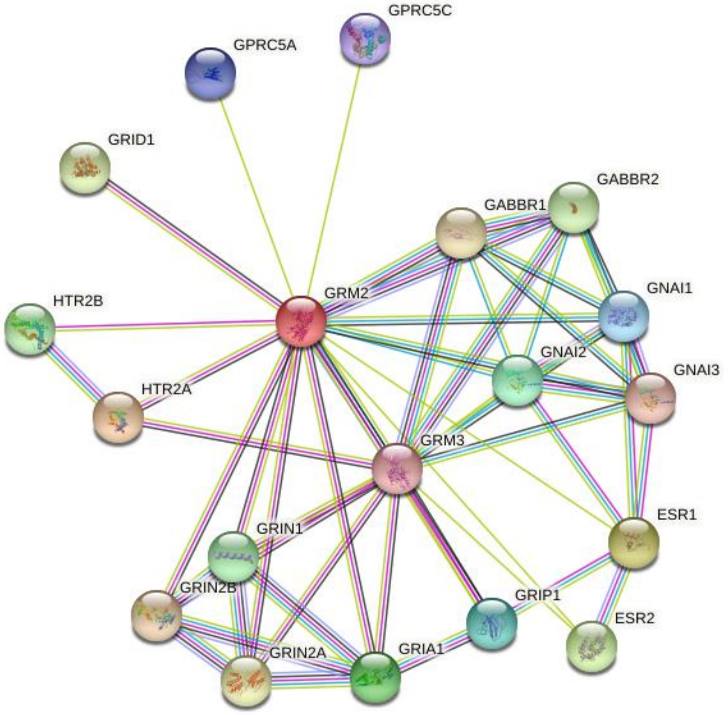

minimum required interaction score: high confidence (0.700)
number of nodes: 19
number of edges: 53
average node degree: 5.58
avg. local clustering coefficient: 0.816
expected number of edges: 20
PPI enrichment p-value: 8.77e-10

MAST1 (UniProt ID: Q9Y2H9)

>sp|Q9Y2H9|MAST1_HUMAN Microtubule-associated serine/threonine-protein kinase 1 OS=Homo sapiens OX=9606 GN=MAST1 PE=1 SV=2

MSDSLWTALSNFSMPSFPGGSMFRRTKSCRTSNRKSLILTSTSPTLPRPHSPLPGHLGSSP
LDSPRNFSPNTPAHFSFASSRRADGRRWSLASLPSSGYGTNTPSSTVSSSCSSQERLHQLP
YQPTVDELHFLSKHFGSTESITDEDGGRRSPAVRPRSRSLSPGRSPSSYDNEIVMMNHVY
KERFPKATAQMEEKLRDFTRAYEPDSVLPLADGVLSFIHHQIIELARDCLTKSRDGLITTV
YFYELQENLEKLLQDAYERSESLEVAFVTQLVKKLLIIISRPARLLECLEFNPEEFYHLLE
AAEGHAKEGHLVKTDIPRYIIRQLGLTRDPFPDVVHLEEQDSGGSNTPEQDDLSEGRSSK
AKKPPGENDFDTIKLISNGAYGAVYLVRHRDTRQRFAMKKINKQNLILRNQIQQAFVER
DILTFAENPFVVGMFCSFETRRHLCMVMEYVEGGDCATLLKNIGALPVEMARMYFAET
VLALEYLHNYGIVHRDLKPDNLLITSMGHIKLTDFGLSKMGLMSLTTNLYEGHIEKDAR
EFLDKQVCGTPEYIAPEVILRQGYGKPVDWWAMGIILYEFLVGCVPFFGDTPEELFGQVI
SDDILWPEGDEALPTEAQLLISSLLQTNPLVRLGAGGAFEVKQHSFFRDLDWTGLLRQK
AEFIPHLESEDDTSYFDTRSDRYHHVNSYDEDDTTEEEPVEIRQFSSCSPRFSKVYSSMEQ
LSQHEPKTPVAAAGSSKREPSTKGPEEKVAGKREGLGGLTLREKTWRGGSPEIKRFSASE
ASFLEGEASPPLGARRRFSALLEPSRFSAPQEDEDEARLRRPPRPSSDPAGSLDARAPKEE
TQGEGTSSAGDSEATDRPRPGDLCPPSKDGDASGPRATNDLVLRRARHQQMSGDVAVE
KRPSRTGGKVIKSASATALSVMIPAVDPHGSSPLASPMSPRSLSSNPSSRDSSPSRDYSPA
VSGLRSPITIQRSGKKYGFTLRAIRVYMGDTDVYSVHHIVWHVEEGGPAQEAGLCAGDL
ITHVNGEPVHGMVHPEVVELILKSGNKVAVTTTPFENTSIRIGPARRSSYKAKMARRNK
RPSAKEGQESKKRSSLFRKITKQSNLLHTSRSLSSLNRSLSSSDSLPGSPTHGLPARSPTHS
YRSTPDSAYLGASSQSSSPASSTPNSPASSASHHIRPSTLHGLSPKLHRQYRSARCKSAGN
IPLSPLAHTPSPTQASPPPLPGHTVGSSHTTQSFPAKLHSSPPVVRPRPKSAEPPRSPLLKR
VQSAEKLGASLSADKKGALRKHSLEVGHPDFRKDFHGELALHSLAESDGETPPVEGLG
APRQVAVRRLGRQESPLSLGADPLLPEGASRPPVSSKEKESPGGAEACTPPRATTPGGRT
LERDVGCTRHQSVQTEDGTGGMARAVAKAALSPVQEHETGRRSSSGEAGTPLVPIVVE
PARPGAKAVVPQPLGADSKGLQEPAPLAPSVPEAPRGRERWVLEVVEERTTLSGPRSKP
ASPKLSPEPQTPSLAPAKCSAPSSAVTPVPPASLLGSGTKPQVGLTSRCPAEAVPPAGLTK
KGVSSPAPPGP

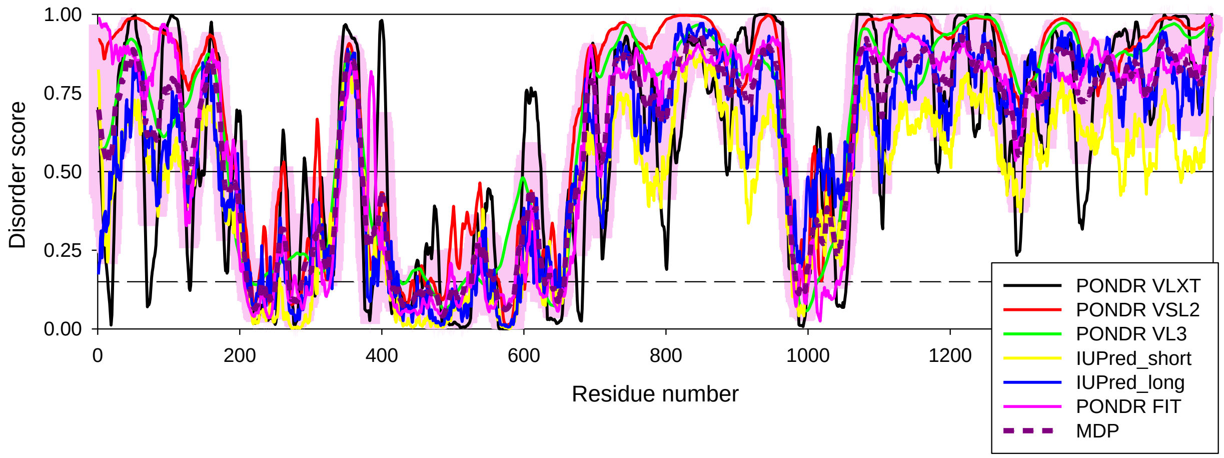

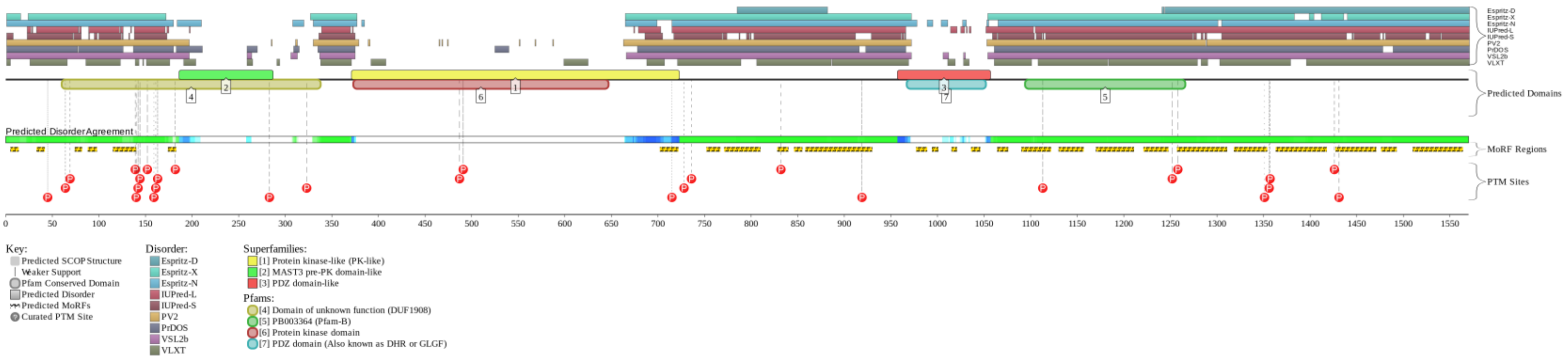
Espritz-D
Espritz-X
Espritz-N
IUPred-L
IUPred-S
PV2
PrDOS
VSL2b
VLXT
Predicted Domains
Predicted Disorder Agreement
MoRF Regions
PTM Sites
Key:
Predicted SCOP Structure
Weaker Support
Pfam Conserved Domain
Predicted Disorder
Predicted MoRFs
Curated PTM Site
Disorder:
Espritz-D
Espritz-X
Espritz-N
IUPred-L
IUPred-S
PV2
PrDOS
VSL2b
VLXT
Superfamilies:
[1] Protein kinase-like (PK-like)
[2] MAST3 pre-PK domain-like
[3] PDZ domain-like
Pfams:
[4] Domain of unknown function (DUF1908)
[5] PB003364 (Pfam-B)
[6] Protein kinase domain
[7] PDZ domain (Also known as DHR or GLGF)

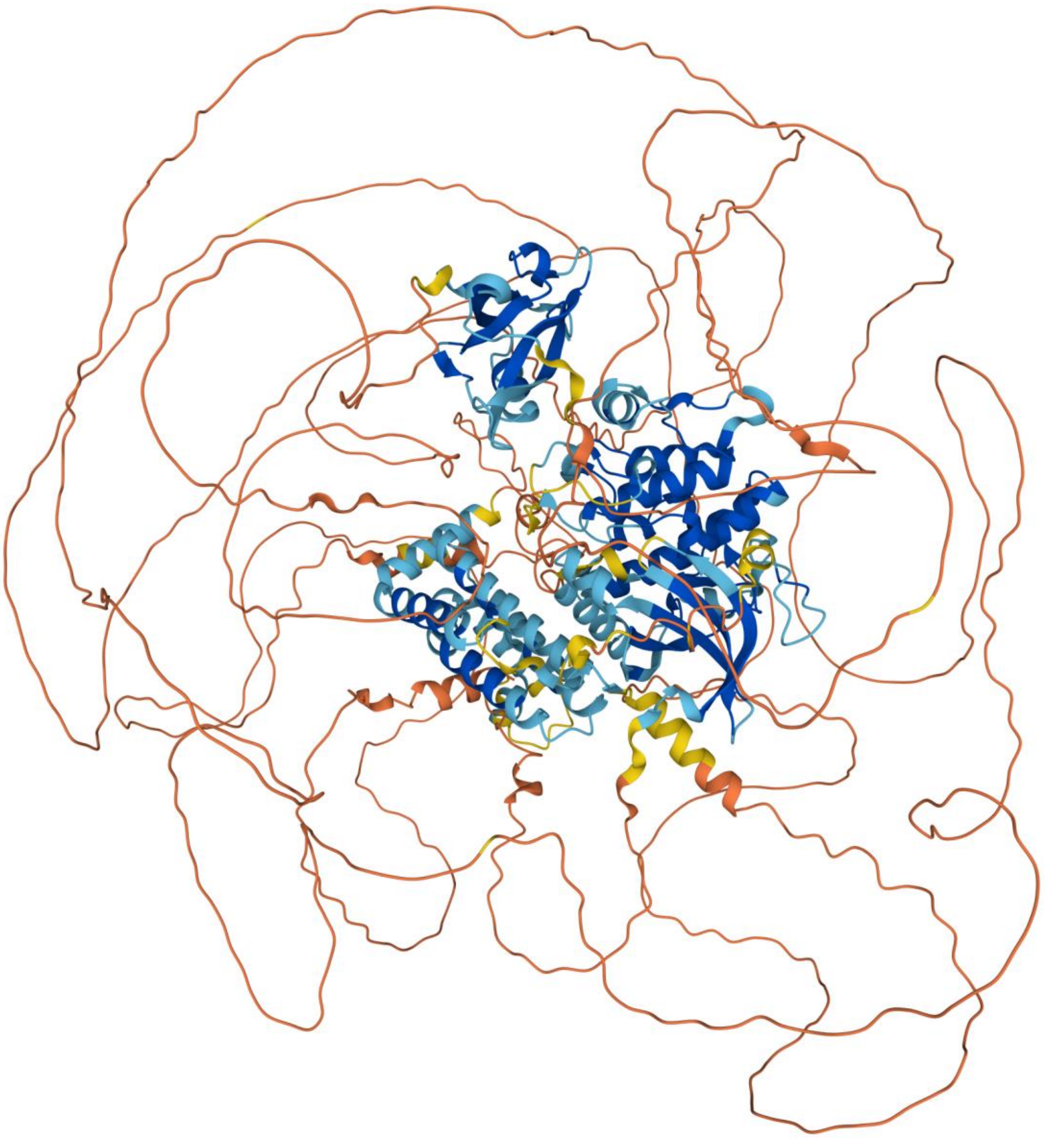

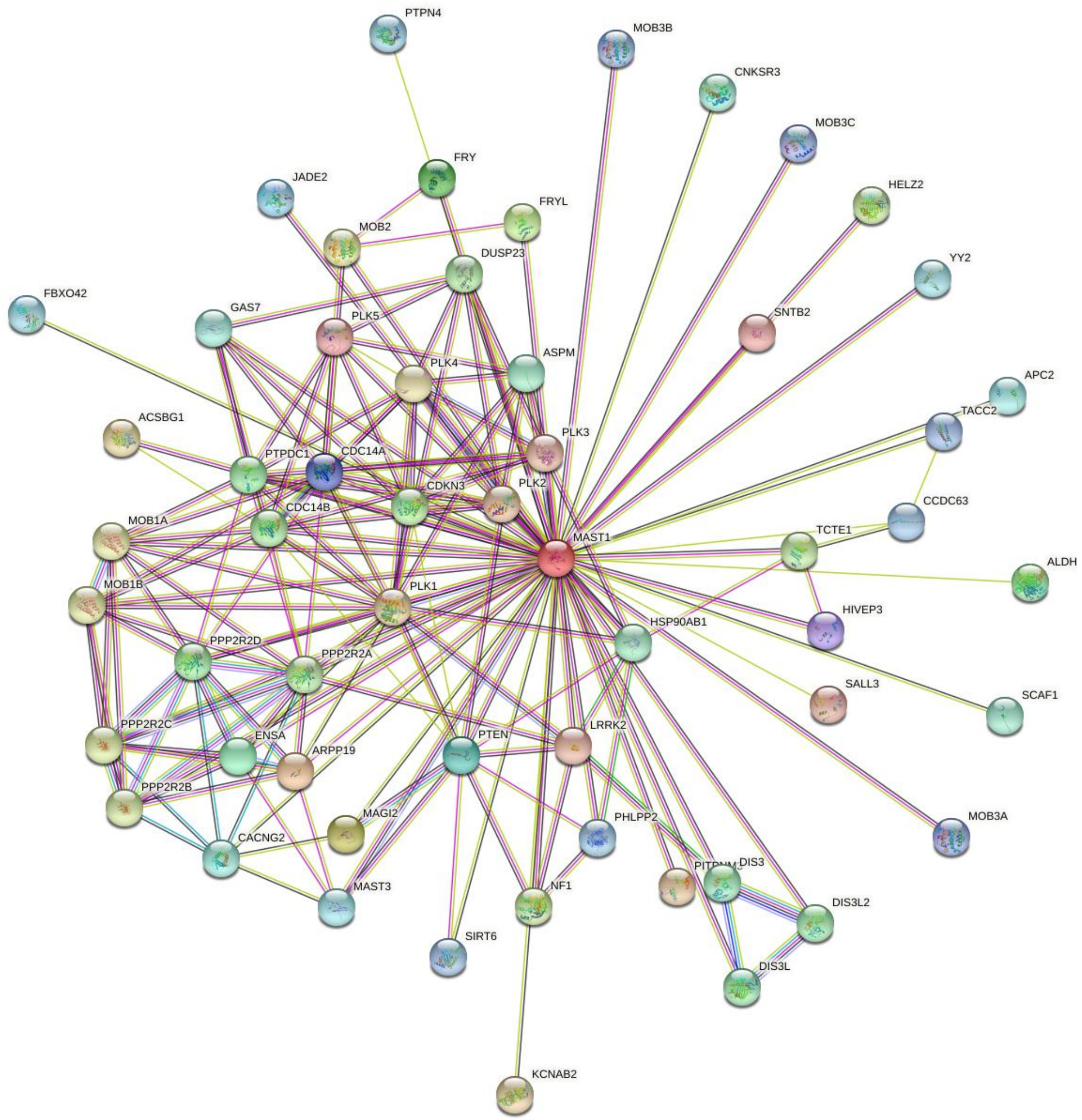

minimum required interaction score: medium confidence (0.400)
number of nodes: 57
number of edges: 178
average node degree: 6.25
avg. local clustering coefficient: 0.751
expected number of edges: 77
PPI enrichment p-value: < 1.0e-16

MAST2 (UniProt ID: Q6P0Q8)

>sp|Q6P0Q8|MAST2_HUMAN Microtubule-associated serine/threonine-protein kinase 2 OS=Homo sapiens OX=9606 GN=MAST2 PE=1 SV=2

MKRSRCRDRPQPPPPDRREDGVQRAAELSQSLPPRRRAPPGRQRLEERTGPAGPEGKEQ
DVVTGVSPLLFRKLSNPDIFSSTGKVKLQRQLSQDDCKLWRGNLASSLSGKQLLPLSSSV
HSSVGQVTWQSSGEASNLVRMRNQSLGQSAPSLTAGLKELSLPRRGSFCRTSNRKSLIV
TSSTSPTLPRPHSPLHGHTGNSPLDSPRNFSPNAPAHFSFVPARRTDGRRWSLASLPSSGY
GTNTPSSTVSSSCSSQEKLHQLPFQPTADELHFLTKHFSTESVPDEEGRQSPAMRPRSRSL
SPGRSPVSFDSEIIMMNHVYKERFPKATAQMEERLAEFISSNTPDSVLPLADGALSFIHHQ
VIEMARDCLDKSRSGLITSQYFYELQDNLEKLLQDAHERSESSEVAFVMQLVKKLMIIIA
RPARLLECLEFDPEEFYHLLEAAEGHAKEGQGIKCDIPRYIVSQLGLTRDPLEEMAQLSS
CDSPDTPETDDSIEGHGASLPSKKTPSEEDFETIKLISNGAYGAVFLVRHKSTRQRFAMK
KINKQNLILRNQIQQAFVERDILTFAENPFVVSMFCSFDTKRHLCMVMEYVEGGDCATL
LKNIGALPVDMVRLYFAETVLALEYLHNYGIVHRDLKPDNLLITSMGHIKLTDFGLSKIG
LMSLTTNLYEGHIEKDAREFLDKQVCGTPEYIAPEVILRQGYGKPVDWWAMGIILYEFL
VGCVPFFGDTPEELFGQVISDEIVWPEGDEALPPDAQDLTSKLLHQNPLERLGTGSAYEV
KQHPFFTGLDWTGLLRQKAEFIPQLESEDDTSYFDTRSERYHHMDSEDEEEVSEDGCLEI
RQFSSCSPRFNKVYSSMERLSLLEERRTPPPTKRSLSEEKEDHSDGLAGLKGRDRSWVIG
SPEILRKRLSVSESSHTESDSSPPMTVRRRCSGLLDAPRFPEGPEEASSTLRRQPQEGIWVL
TPPSGEGVSGPVTEHSGEQRPKLDEEAVGRSSGSSPAMETRGRGTSQLAEGATAKAISDL
AVRRARHRLLSGDSTEKRTARPVNKVIKSASATALSLLIPSEHHTCSPLASPMSPHSQSSN
PSSRDSSPSRDFLPALGSMRPPIIIHRAGKKYGFTLRAIRVYMGDSDVYTVHHMVWHVE
DGGPASEAGLRQGDLITHVNGEPVHGLVHTEVVELILKSGNKVAISTTPLENTSIKVGPA
RKGSYKAKMARRSKRSRGKDGQESRKRSSLFRKITKQASLLHTSRSLSSLNRSLSSGESG
PGSPTHSHSLSPRSPTQGYRVTPDAVHSVGGNSSQSSSPSSSVPSSPAGSGHTRPSSLHGL
APKLQRQYRSPRRKSAGSIPLSPLAHTPSPPPPTASPQRSPSPLSGHVAQAFPTKLHLSPPL
GRQLSRPKSAEPPRSPLLKRVQSAEKLAAALAASEKKLATSRKHSLDLPHSELKKELPPR
EVSPLEVVGARSVLSGKGALPGKGVLQPAPSRALGTLRQDRAERRESLQKQEAIREVDS
SEDDTEEGPENSQGAQELSLAPHPEVSQSVAPKGAGESGEEDPFPSRDPRSLGPMVPSLL
TGITLGPPRMESPSGPHRRLGSPQAIEEAASSSSAGPNLGQSGATDPIPPEGCWKAQHLHT
QALTALSPSTSGLTPTSSCSPPSSTSGKLSMWSWKSLIEGPDRASPSRKATMAGGLANLQ
DLENTTPAQPKNLSPREQGKTQPPSAPRLAHPSYEDPSQGWLWESECAQAVKEDPALSI
TQVPDASGDRRQDVPCRGCPLTQKSEPSLRRGQEPGGHQKHRDLALVPDELLKQT

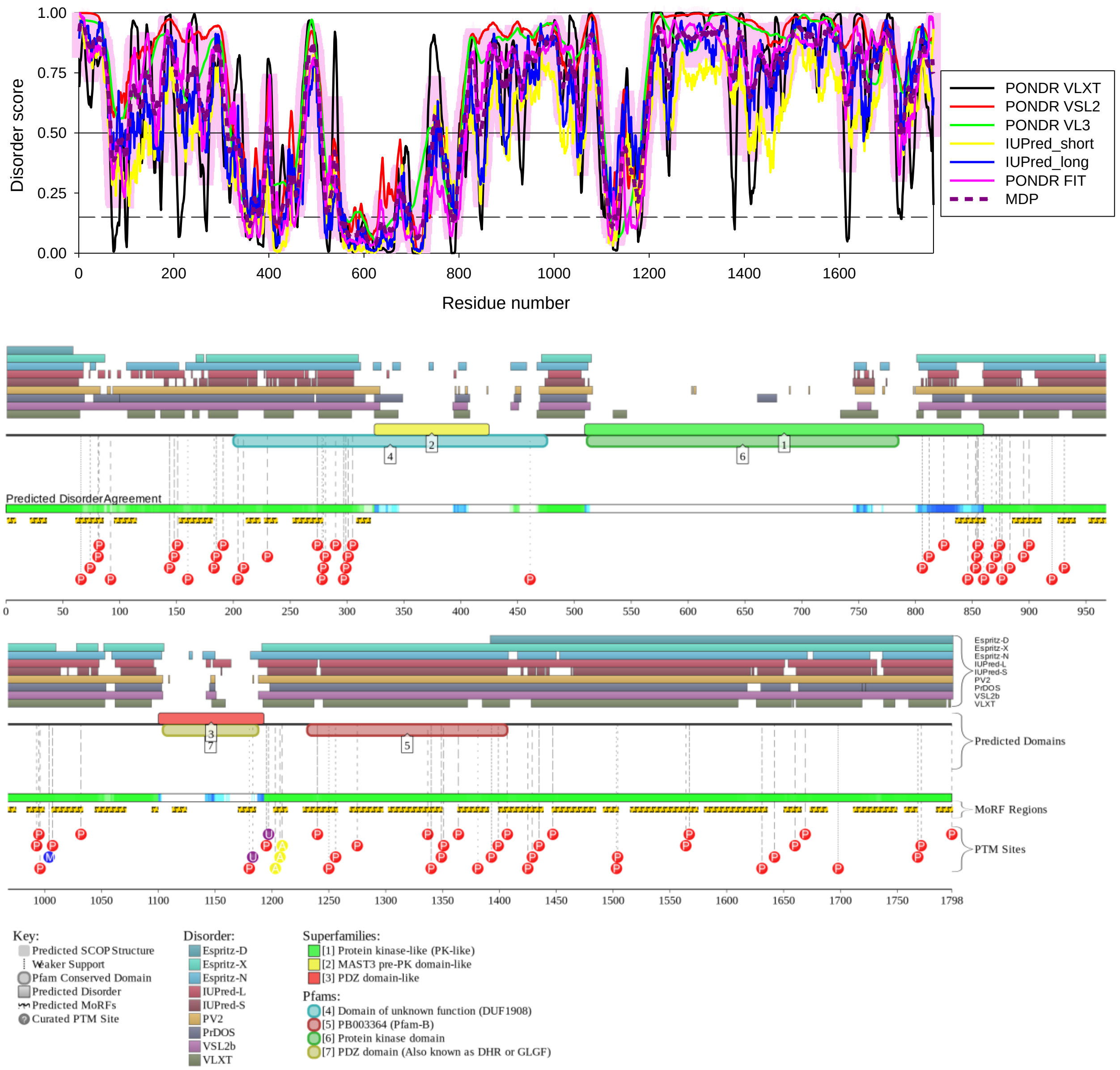

Disorder score
Residue number
PONDR VLXT
PONDR VSL2
PONDR VL3
IUPred_short
IUPred_long
PONDR FIT
MDP
Predicted DisorderAgreement
Espritz-D
Espritz-X
Espritz-N
IUPred-L
IUPred-S
PV2
PrDOS
VSL2b
VLXT
Predicted Domains
MoRF Regions
PTM Sites
Key:
Predicted SCOP Structure
Weaker Support
Pfam Conserved Domain
Predicted Disorder
Predicted MoRFs
Curated PTM Site
Disorder:
Espritz-D
Espritz-X
Espritz-N
IUPred-L
IUPred-S
PV2
PrDOS
VSL2b
VLXT
Superfamilies:
[1] Protein kinase-like (PK-like)
[2] MAST3 pre-PK domain-like
[3] PDZ domain-like
Pfams:
[4] Domain of unknown function (DUF1908)
[5] PB003364 (Pfam-B)
[6] Protein kinase domain
[7] PDZ domain (Also known as DHR or GLGF)

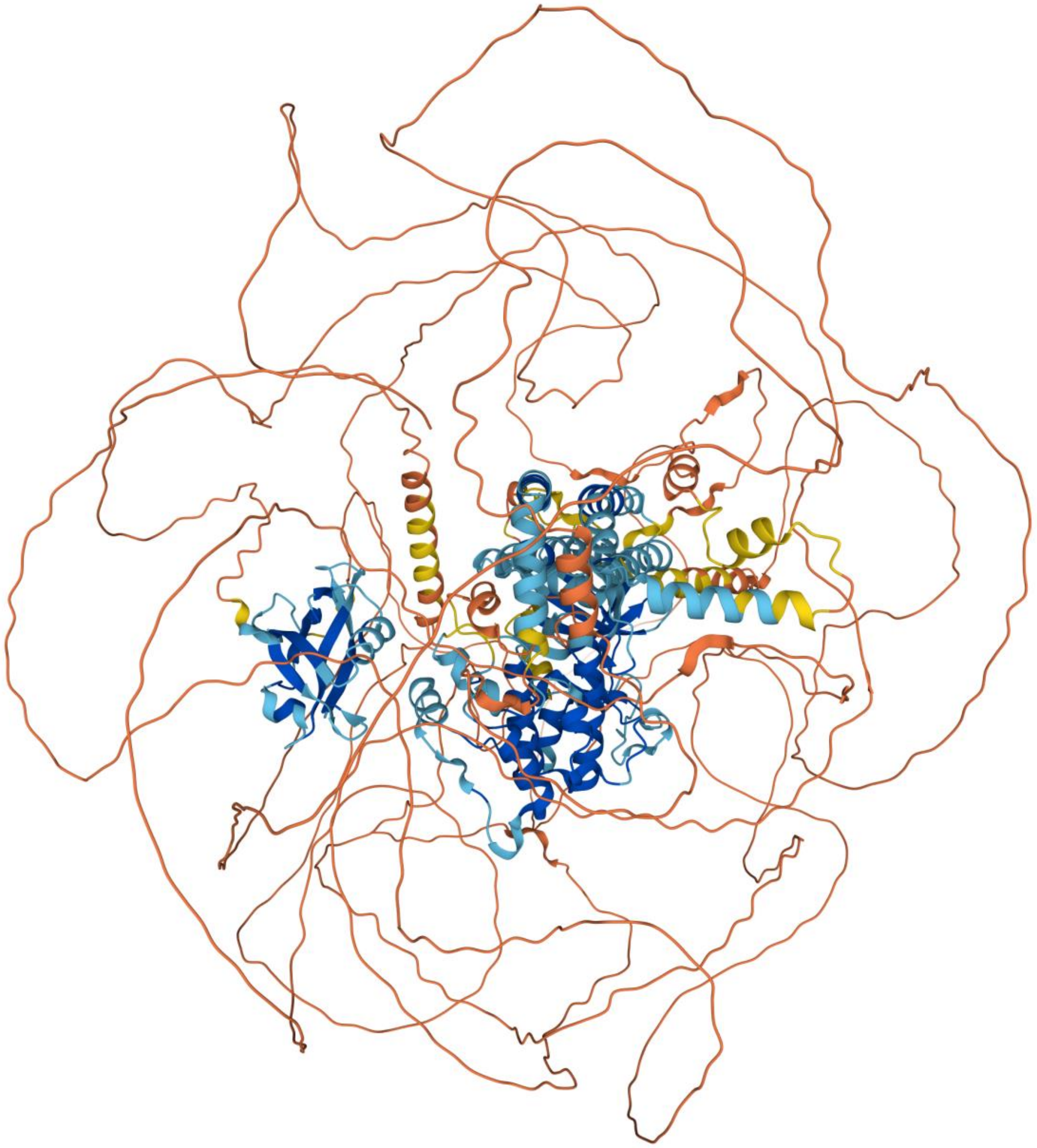

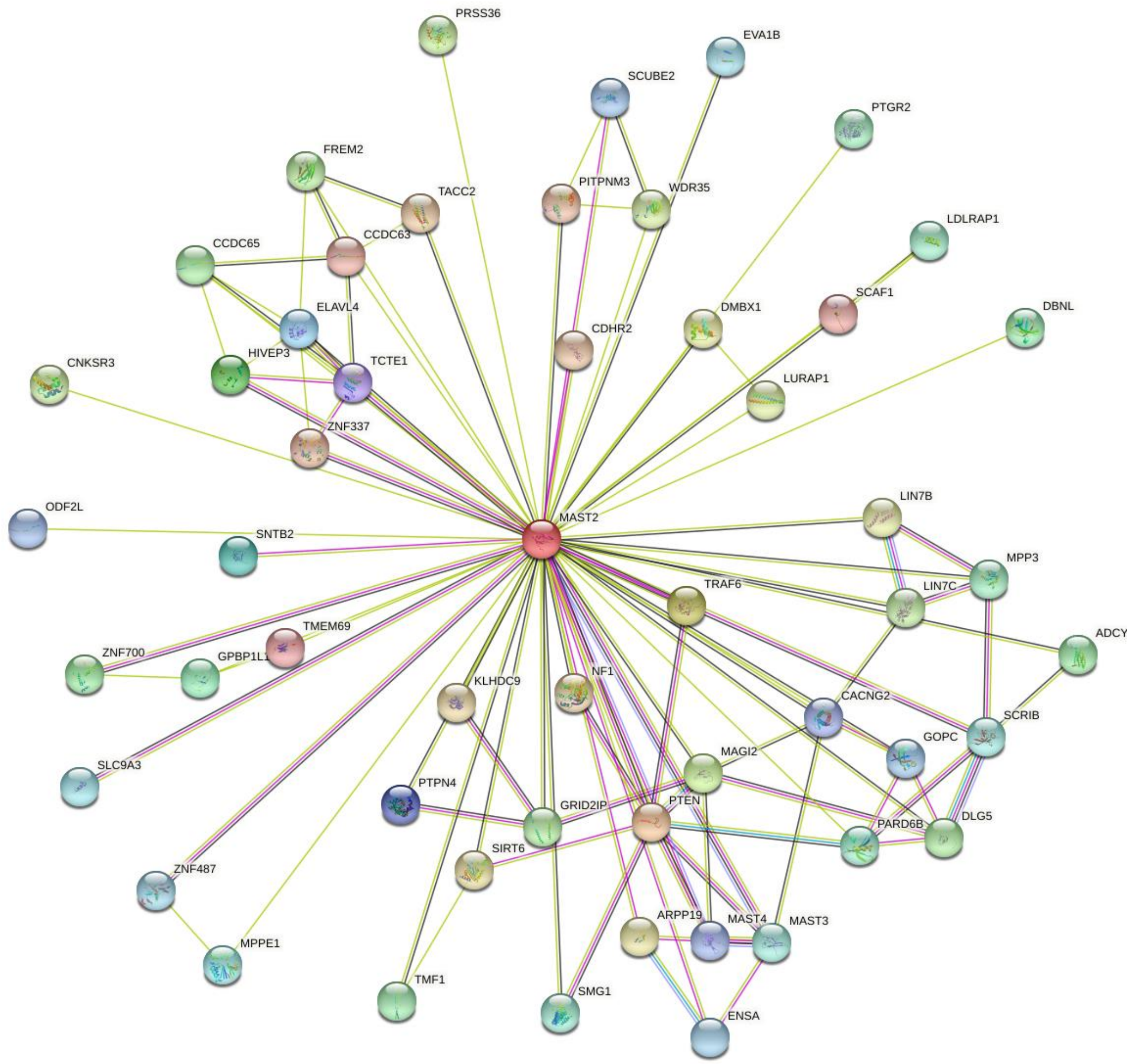

minimum required interaction score: medium confidence (0.400)
number of nodes: 53
number of edges: 106
average node degree: 4
avg. local clustering coefficient: 0.836
expected number of edges: 60
PPI enrichment p-value: 3.95e-08

PTPN4 (UniProt ID: P29074)

>sp|P29074|PTN4_HUMAN Tyrosine-protein phosphatase non-receptor type 4 OS=Homo sapiens OX=9606 GN=PTPN4 PE=1 SV=1

MTSRFRLPAGRTYNVRASELARDRQHTEVVCNILLLDNTVQAFKVNKHDQGQVLLDVV
FKHLDLTEQDYFGLQLADDSTDNPRWLDPNKPIRKQLKRGSPYSLNFRVKFFVSDPNKL
QEEYTRYQYFLQIKQDILTGRLPCPSNTAALLASFAVQSELGDYDQSENLSGYLSDYSFIP
NQPQDFEKEIAKLHQQHIGLSPAEAEFNYLNTARTLELYGVEFHYARDQSNNEIMIGVM
SGGILIYKNRVRMNTFPWLKIVKISFKCKQFFIQLRKELHESRETLLGFNMVNYRACKNL
WKACVEHHTFFRLDRPLPPQKNFFAHYFTLGSKFRYCGRTEVQSVQYGKEKANKDRVF
ARSPSKPLARKLMDWEVVSRNSISDDRLETQSLPSRSPPGTPNHRNSTFTQEGTRLRPSS
VGHLVDHMVHTSPSEVFVNQRSPSSTQANSIVLESSPSQETPGDGKPPALPPKQSKKNSW
NQIHYSHSQQDLESHINETFDIPSSPEKPTPNGGIPHDNLVLIRMKPDENGRFGFNVKGGY
DQKMPVIVSRVAPGTPADLCVPRLNEGDQVVLINGRDIAEHTHDQVVLFIKASCERHSG
ELMLLVRPNAVYDVVEEKLENEPDFQYIPEKAPLDSVHQDDHSLRESMIQLAEGLITGT
VLTQFDQLYRKKPGMTMSCAKLPQNISKNRYRDISPYDATRVILKGNEDYINANYINME
IPSSSIINQYIACQGPLPHTCTDFWQMTWEQGSSMVVMLTTQVERGRVKCHQYWPEPTG
SSSYGCYQVTCHSEEGNTAYIFRKMTLFNQEKNESRPLTQIQYIAWPDHGVPDDSSDFLD
FVCHVRNKRAGKEEPVVVHCSAGIGRTGVLITMETAMCLIECNQPVYPLDIVRTMRDQR
AMMIQTPSQYRFVCEAILKVYEEGFVKPLTTSTNK

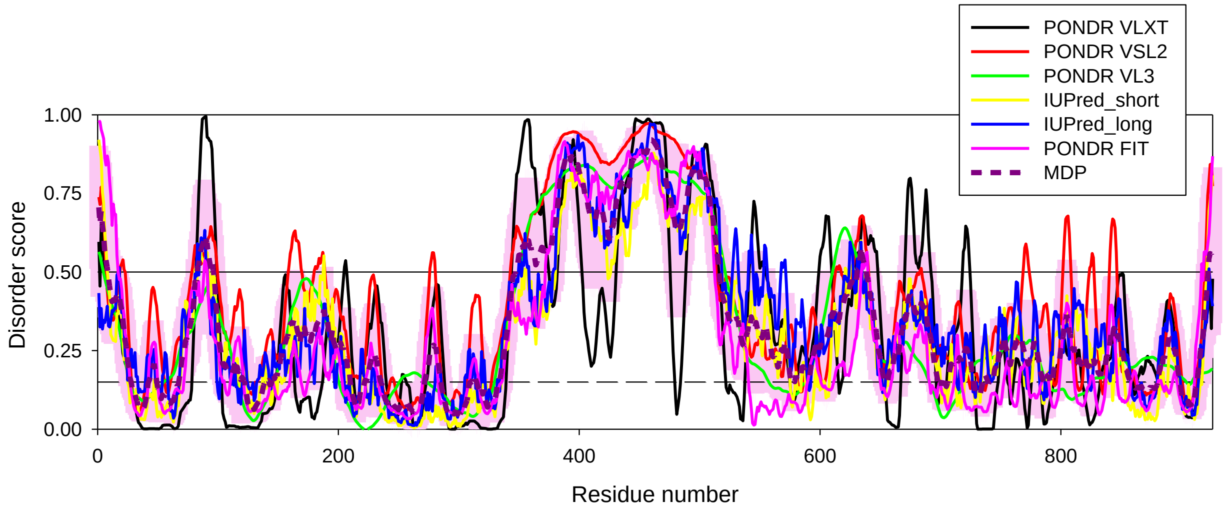

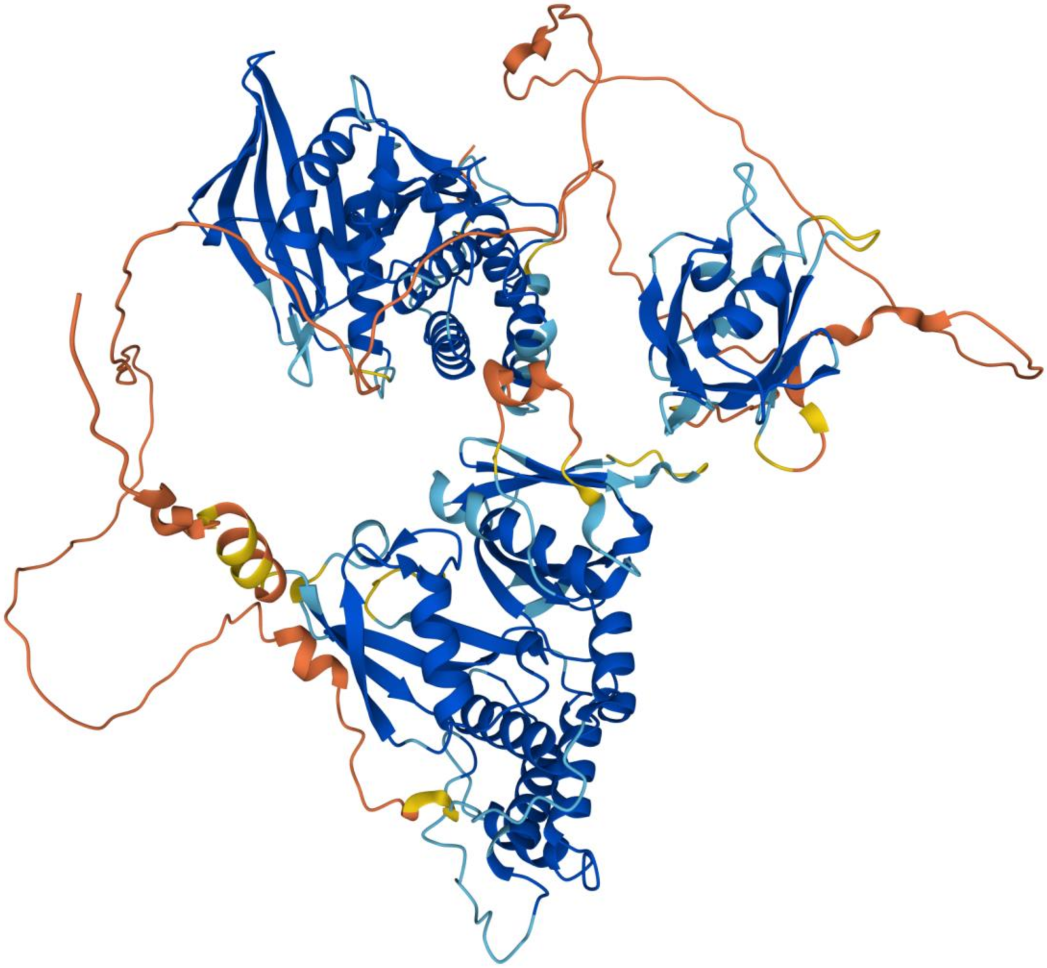

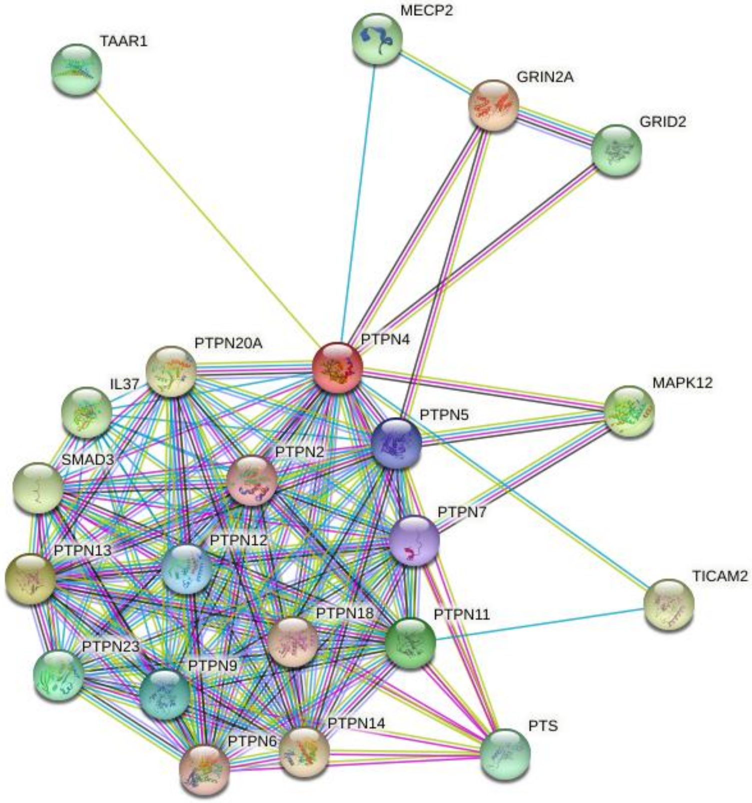

minimum required interaction score: high confidence (0.700)
number of nodes: 22
number of edges: 127
average node degree: 11.5
avg. local clustering coefficient: 0.917
expected number of edges: 23
PPI enrichment p-value: < 1.0e-16

DLG2 (UniProt ID: Q15700)

>sp|Q15700|DLG2_HUMAN Disks large homolog 2 OS=Homo sapiens OX=9606 GN=DLG2 PE=1 SV=3

MFFACYCALRTNVKKYRYQDEDAPHDHSLPRLTHEVRGPELVHVSEKNLSQIENVHGY
VLQSHISPLKASPAPIIVNTDTLDTIPYVNGTEIEYEFEEITLERGNSGLGFSIAGGTDNPHI
GDDPGIFITKIIPGGAAAEDGRLRVNDCILRVNEVDVSEVSHSKAVEALKEAGSIVRLYV
RRRRPILETVVEIKLFKGPKGLGFSIAGGVGNQHIPGDNSIYVTKIIDGGAAQKDGRLQVG
DRLLMVNNYSLEEVTHEEAVAILKNTSEVVYLKVGKPTTIYMTDPYGPPDITHSYSPPM
ENHLLSGNNGTLEYKTSLPPISPGRYSPIPKHMLVDDDYTRPPEPVYSTVNKLCDKPASP
RHYSPVECDKSFLLSAPYSHYHLGLLPDSEMTSHSQHSTATRQPSMTLQRAVSLEGEPR
KVVLHKGSTGLGFNIVGGEDGEGIFVSFILAGGPADLSGELQRGDQILSVNGIDLRGASH
EQAAAALKGAGQTVTIIAQYQPEDYARFEAKIHDLREQMMNHSMSSGSGSLRTNQKRS
LYVRAMFDYDKSKDSGLPSQGLSFKYGDILHVINASDDEWWQARRVMLEGDSEEMGV
IPSKRRVERKERARLKTVKFNAKPGVIDSKGSFNDKRKKSFIFSRKFPFYKNKEQSEQETS
DPERGQEDLILSYEPVTRQEINYTRPVIILGPMKDRINDDLISEFPDKFGSCVPHTTRPKRD
YEVDGRDYHFVISREQMEKDIQEHKFIEAGQYNDNLYGTSVQSVRFVAERGKHCILDVS
GNAIKRLQVAQLYPIAIFIKPRSLEPLMEMNKRLTEEQAKKTYDRAIKLEQEFGEYFTAIV
QGDTLEDIYNQCKLVIEEQSGPFIWIPSKEKL

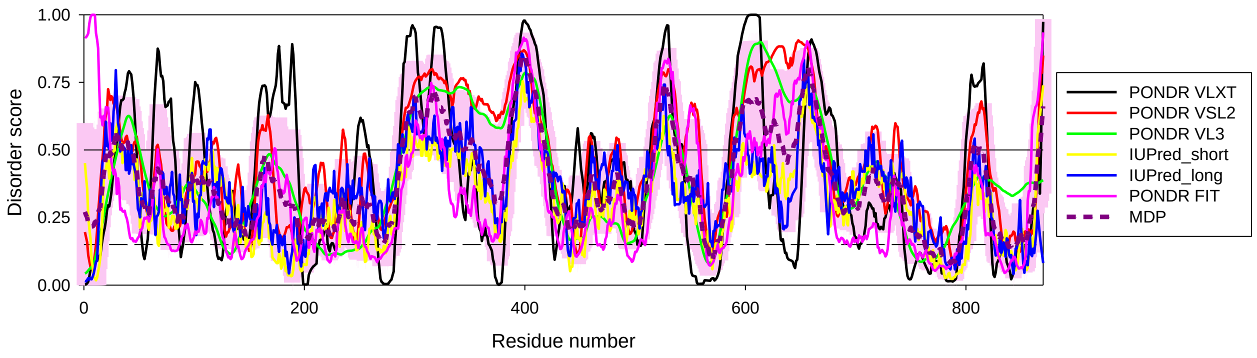

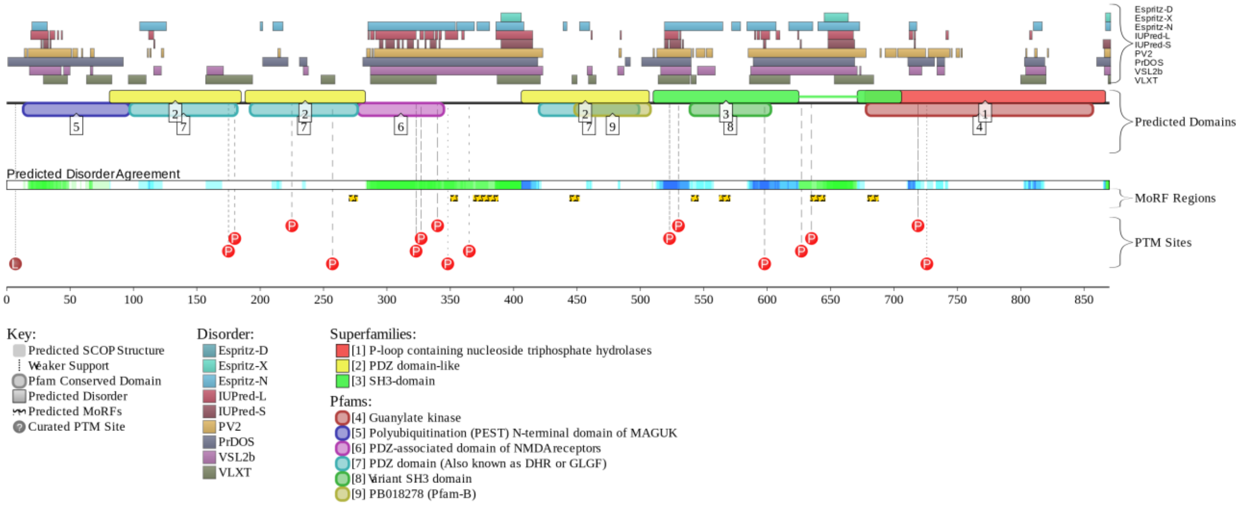

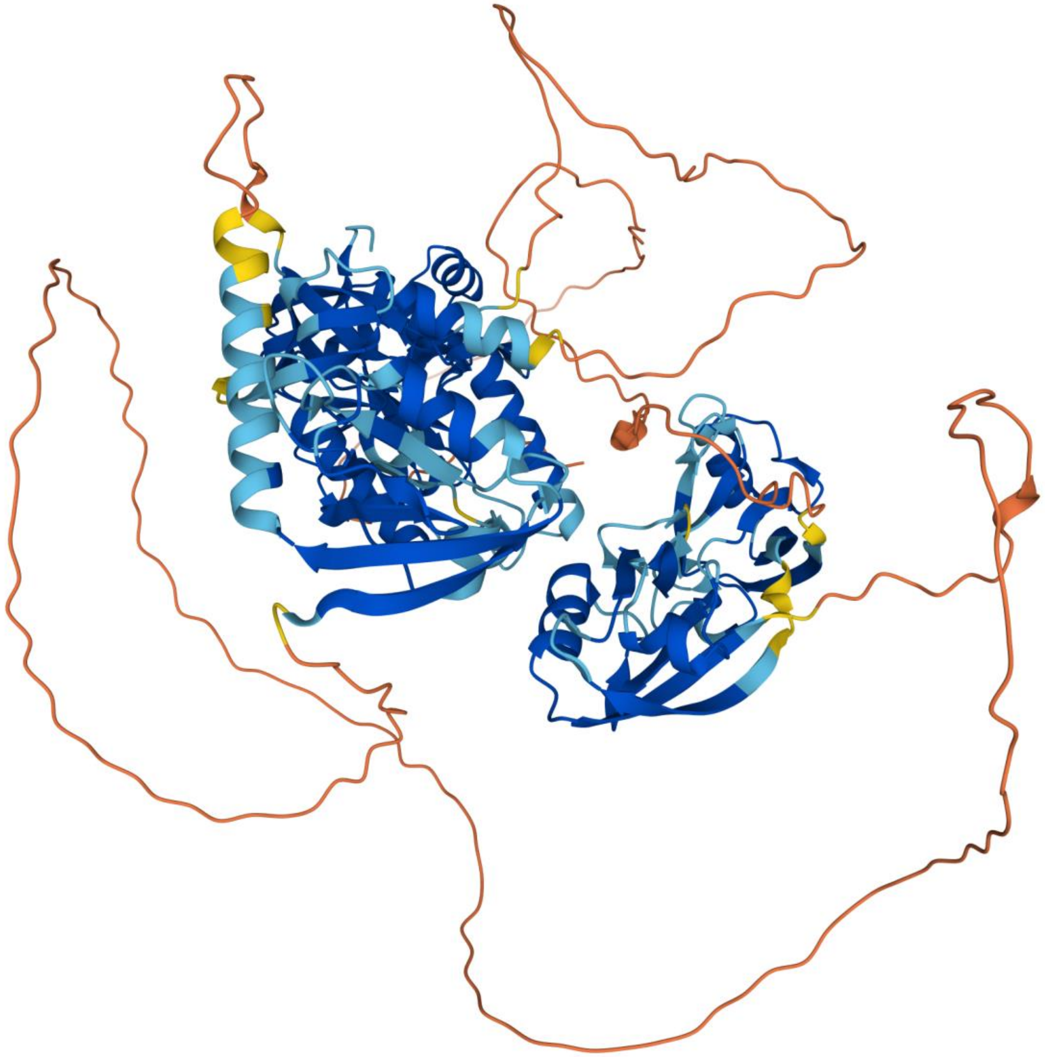

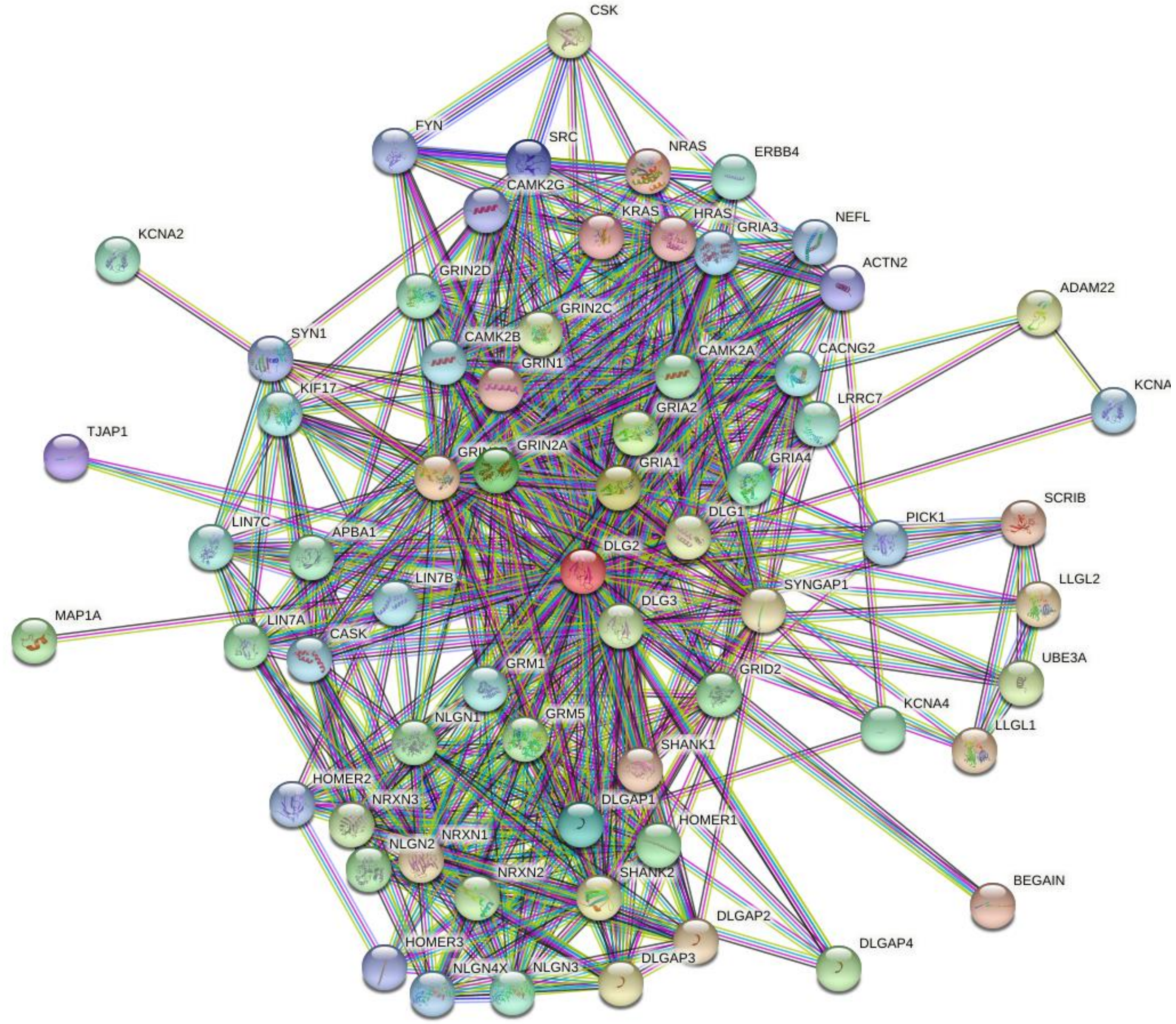

minimum required interaction score: high confidence (0.700)
number of nodes: 65
number of edges: 565
average node degree: 17.4
avg. local clustering coefficient: 0.737
expected number of edges: 87
PPI enrichment p-value: < 1.0e-16

MPDZ (UniProt ID: O75970

>sp|O75970|MPDZ_HUMAN Multiple PDZ domain protein OS=Homo sapiens OX=9606 GN=MPDZ PE=1 SV=2

MLEAIDKNRALHAAERLQTKLRERGDVANEDKLSLLKSVLQSPLFSQILSLQTSVQQLK
DQVNIATSATSNIEYAHVPHLSPAVIPTLQNESFLLSPNNGNLEALTGPGIPHINGKPACD
EFDQLIKNMAQGRHVEVFELLKPPSGGLGFSVVGLRSENRGELGIFVQEIQEGSVAHRD
GRLKETDQILAINGQALDQTITHQQAISILQKAKDTVQLVIARGSLPQLVSPIVSRSPSAAS
TISAHSNPVHWQHMETIELVNDGSGLGFGIIGGKATGVIVKTILPGGVADQHGRLCSGDH
ILKIGDTDLAGMSSEQVAQVLRQCGNRVKLMIARGAIEERTAPTALGITLSSSPTSTPELR
VDASTQKGEESETFDVELTKNVQGLGITIAGYIGDKKLEPSGIFVKSITKSSAVEHDGRIQI
GDQIIAVDGTNLQGFTNQQAVEVLRHTGQTVLLTLMRRGMKQEAELMSREDVTKDAD
LSPVNASIIKENYEKDEDFLSSTRNTNILPTEEEGYPLLSAEIEEIEDAQKQEAALLTKWQR
IMGINYEIVVAHVSKFSENSGLGISLEATVGHHFIRSVLPEGPVGHSGKLFSGDELLEVNG
ITLLGENHQDVVNILKELPIEVTMVCCRRTVPPTTQSELDSLDLCDIELTEKPHVDLGEFI
GSSETEDPVLAMTDAGQSTEEVQAPLAMWEAGIQHIELEKGSKGLGFSILDYQDPIDPAS
TVIIIRSLVPGGIAEKDGRLLPGDRLMFVNDVNLENSSLEEAVEALKGAPSGTVRIGVAKP
LPLSPEEGYVSAKEDSFLYPPHSCEEAGLADKPLFRADLALVGTNDADLVDESTFESPYS
PENDSIYSTQASILSLHGSSCGDGLNYGSSLPSSPPKDVIENSCDPVLDLHMSLEELYTQN
LLQRQDENTPSVDISMGPASGFTINDYTPANAIEQQYECENTIVWTESHLPSEVISSAELP
SVLPDSAGKGSEYLLEQSSLACNAECVMLQNVSKESFERTINIAKGNSSLGMTVSANKD
GLGMIVRSIIHGGAISRDGRIAIGDCILSINEESTISVTNAQARAMLRRHSLIGPDIKITYVP
AEHLEEFKISLGQQSGRVMALDIFSSYTGRDIPELPEREEGEGEESELQNTAYSNWNQPR
RVELWREPSKSLGISIVGGRGMGSRLSNGEVMRGIFIKHVLEDSPAGKNGTLKPGDRIVE
VDGMDLRDASHEQAVEAIRKAGNPVVFMVQSIINRPRKSPLPSLLHNLYPKYNFSSTNPF
ADSLQINADKAPSQSESEPEKAPLCSVPPPPPSAFAEMGSDHTQSSASKISQDVDKEDEFG
YSWKNIRERYGTLTGELHMIELEKGHSGLGLSLAGNKDRSRMSVFIVGIDPNGAAGKDG
RLQIADELLEINGQILYGRSHQNASSIIKCAPSKVKIIFIRNKDAVNQMAVCPGNAVEPLP
SNSENLQNKETEPTVTTSDAAVDLSSFKNVQHLELPKDQGGLGIAISEEDTLSGVIIKSLT
EHGVAATDGRLKVGDQILAVDDEIVVGYPIEKFISLLKTAKMTVKLTIHAENPDSQAVPS
AAGAASGEKKNSSQSLMVPQSGSPEPESIRNTSRSSTPAIFASDPATCPIIPGCETTIEISKG
RTGLGLSIVGGSDTLLGAIIIHEVYEEGAACKDGRLWAGDQILEVNGIDLRKATHDEAIN
VLRQTPQRVRLTLYRDEAPYKEEEVCDTLTIELQKKPGKGLGLSIVGKRNDTGVFVSDIV
KGGIADADGRLMQGDQILMVNGEDVRNATQEAVAALLKCSLGTVTLEVGRIKAGPFHS
ERRPSQSSQVSEGSLSSFTFPLSGSSTSESLESSSKKNALASEIQGLRTVEMKKGPTDSLGI
SIAGGVGSPLGDVPIFIAMMHPTGVAAQTQKLRVGDRIVTICGTSTEGMTHTQAVNLLK
NASGSIEMQVVAGGDVSVVTGHQQEPASSSLSFTGLTSSSIFQDDLGPPQCKSITLERGPD
GLGFSIVGGYGSPHGDLPIYVKTVFAKGAASEDGRLKRGDQIIAVNGQSLEGVTHEEAV
AILKRTKGTVTLMVLS

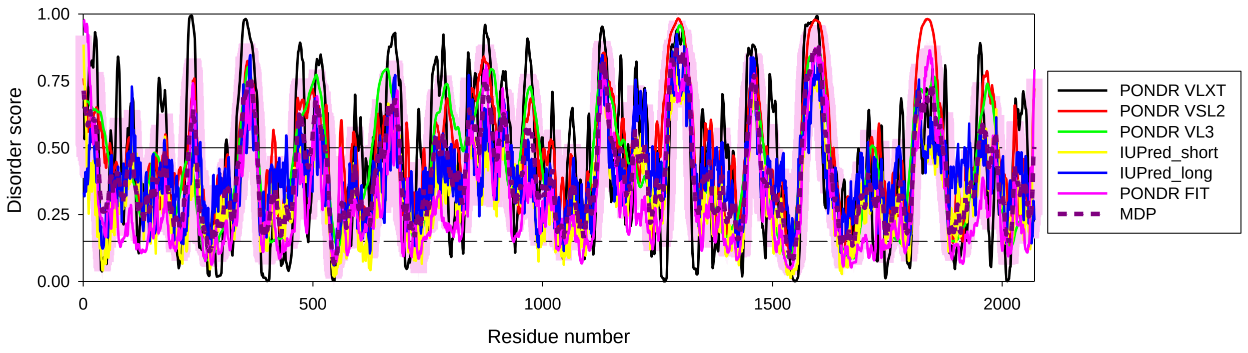
1.00
0.75
0.50
0.25
0.00
Disorder score
0
500
1000
1500
2000
Residue number
PONDR VLXT
PONDR VSL2
PONDR VL3
IUPred_short
IUPred_long
PONDR FIT
MDP

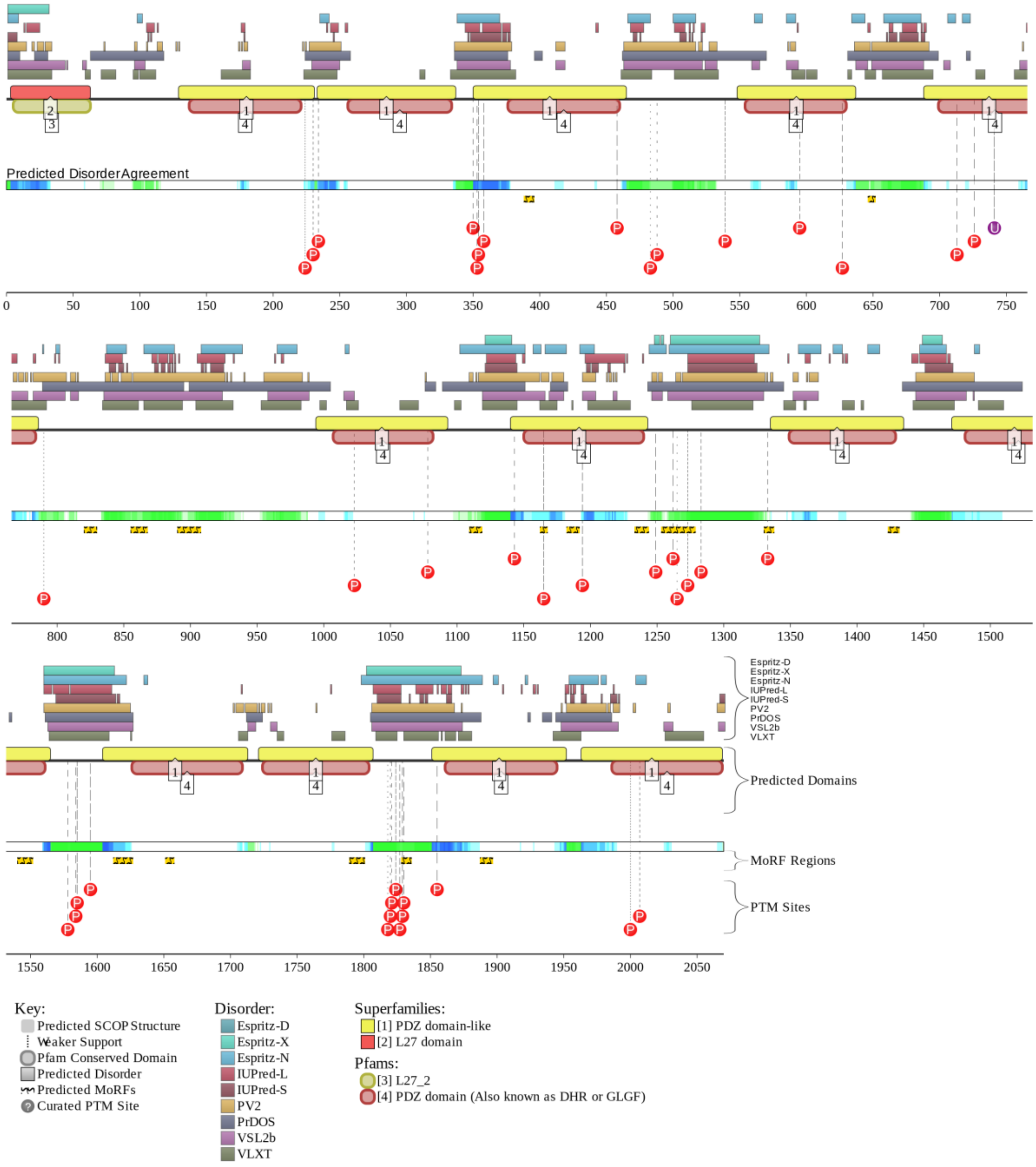
Predicted DisorderAgreement
Espritz-D
Espritz-X
Espritz-N
IUPred-L
IUPred-S
PV2
PrDOS
VSL2b
VLXT
Predicted Domains
MoRF Regions
PTM Sites
Key:
Predicted SCOP Structure
Weaker Support
Pfam Conserved Domain
Predicted Disorder
Predicted MoRFs
Curated PTM Site
Disorder:
Espritz-D
Espritz-X
Espritz-N
IUPred-L
IUPred-S
PV2
PrDOS
VSL2b
VLXT
Superfamilies:
[1] PDZ domain-like
[2] L27 domain
Pfams:
[3] L27_2
[4] PDZ domain (Also known as DHR or GLGF)

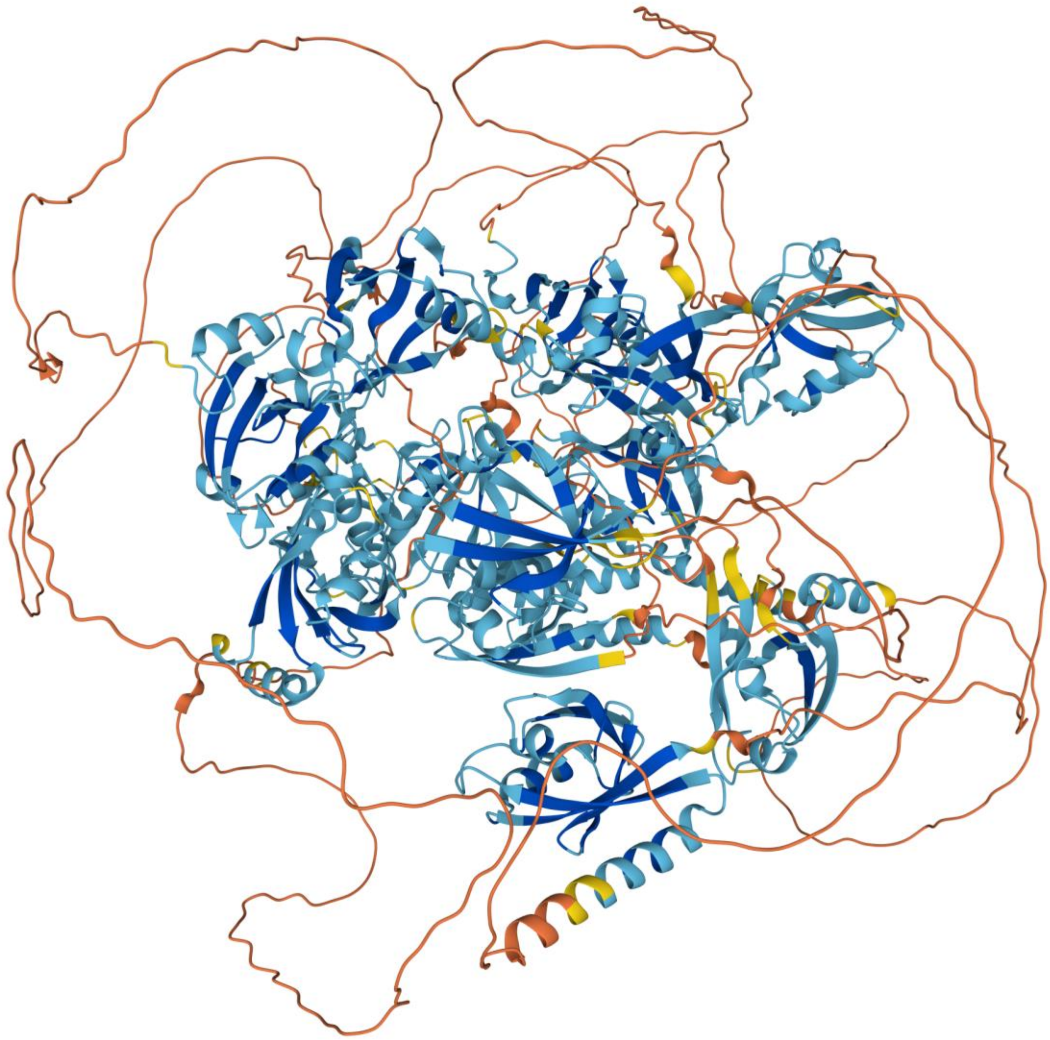

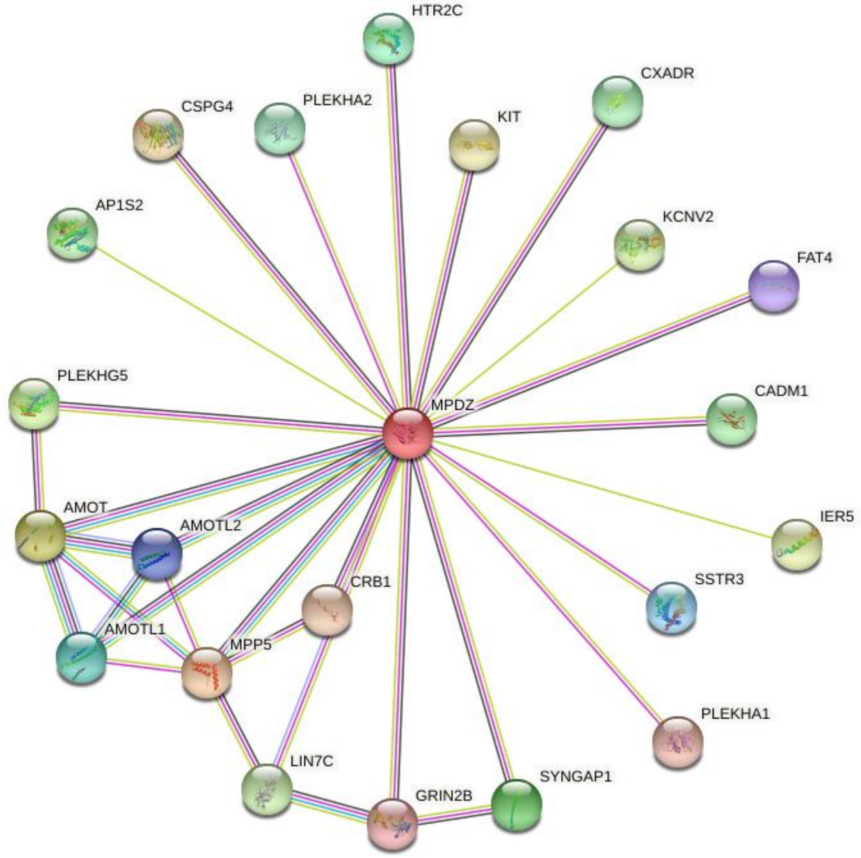

minimum required interaction score: high confidence (0.700)
number of nodes: 22
number of edges: 32
average node degree: 2.91
avg. local clustering coefficient: 0.892
expected number of edges: 21
PPI enrichment p-value: 0.019

SNAP25 (UniProt ID: P60880)

>sp|P60880|SNP25_HUMAN Synaptosomal-associated protein 25 OS=Homo sapiens OX=9606 GN=SNAP25 PE=1 SV=1

MAEDADMRNELEEMQRRADQLADESLESTRRMLQLVEESKDAGIRTLVMLDEQGEQL
ERIEEGMDQINKDMKEAEKNLTDLGKFCGLCVCPCNKLKSSDAYKKAWGNNQDGVVA
SQPARVVDEREQMAISGGFIRRVTNDARENEMDENLEQVSGIIGNLRHMALDMGNEIDT
QNRQIDRIMEKADSNKTRIDEANQRATKMLGSG

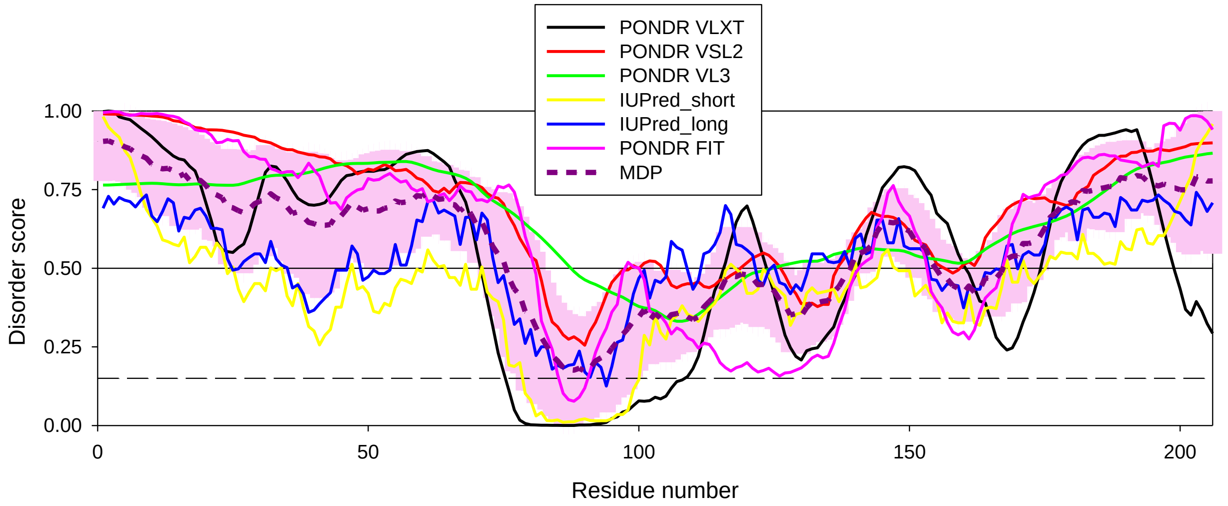

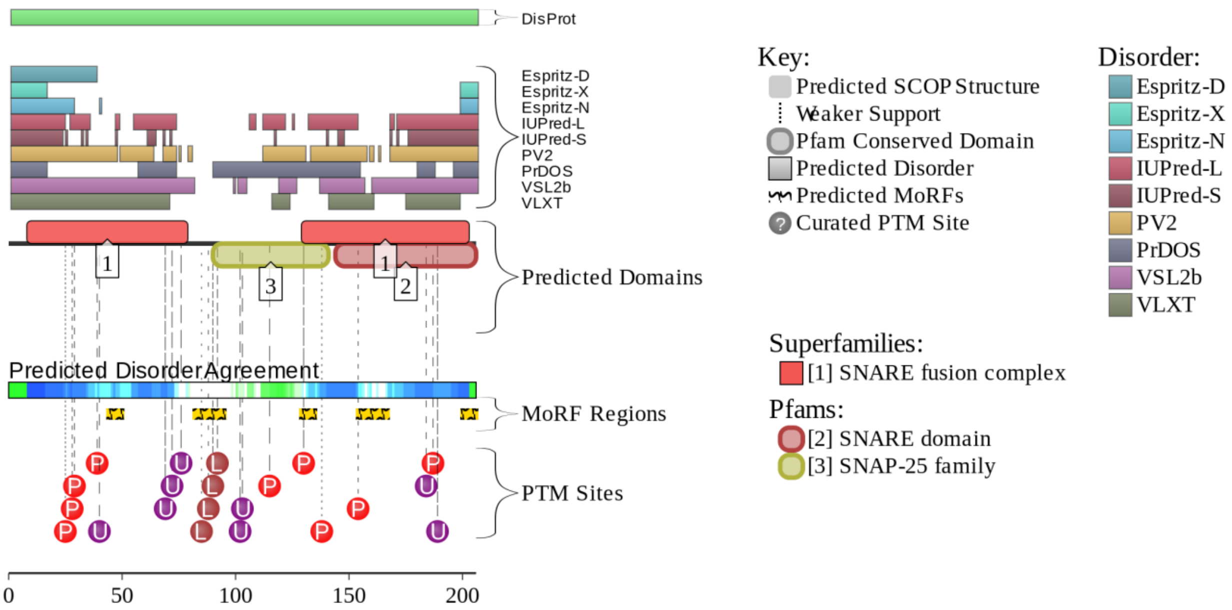

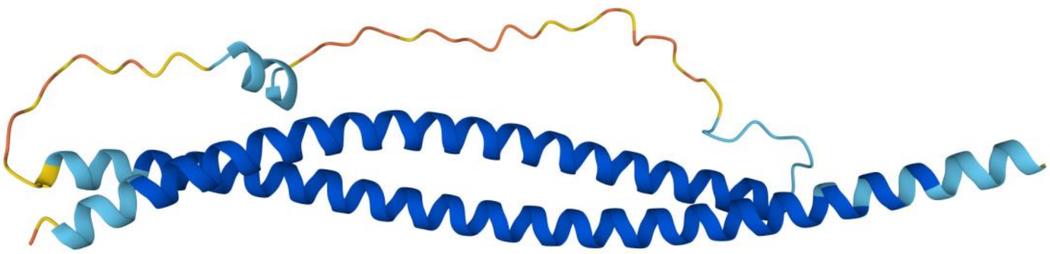

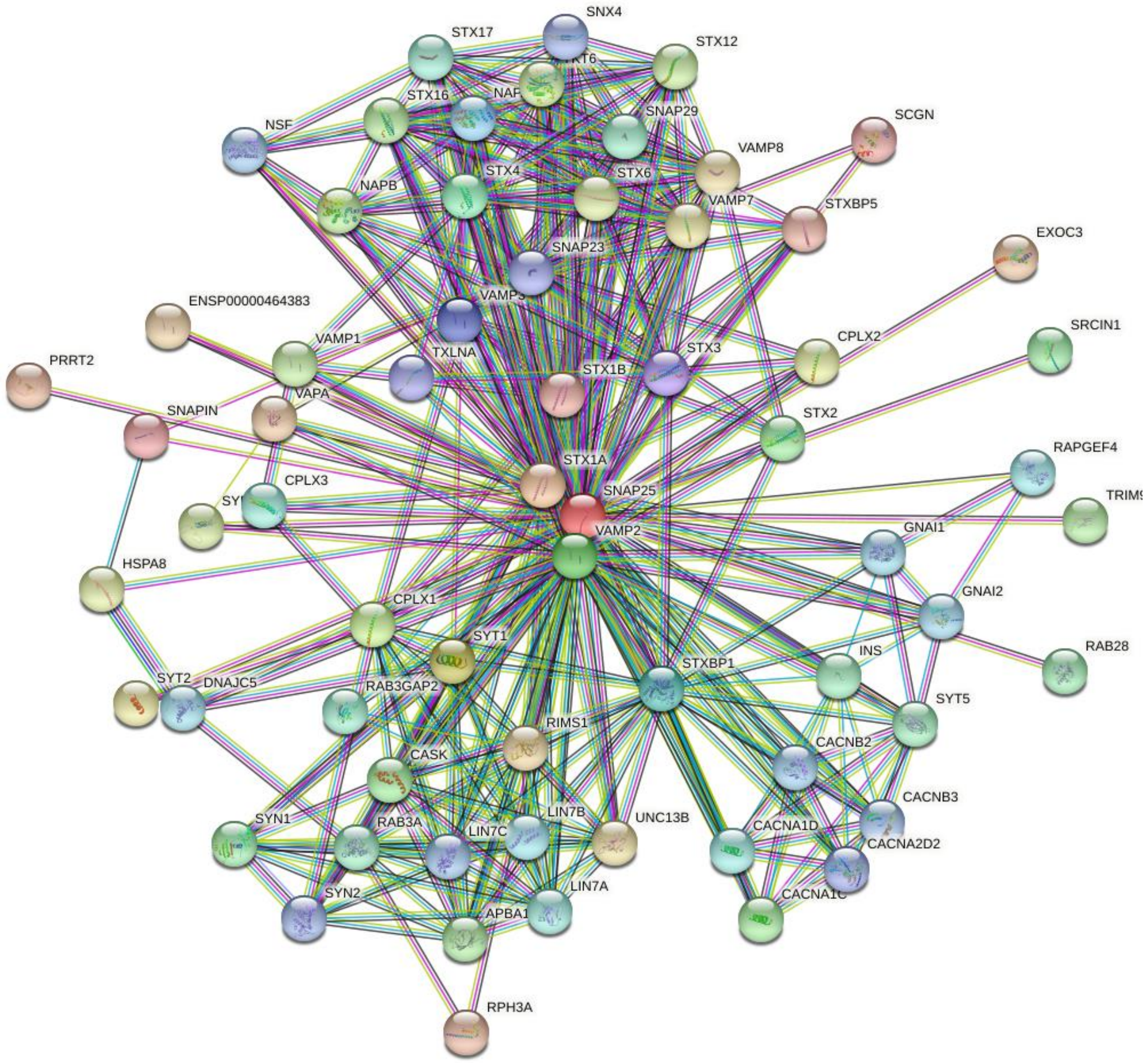

minimum required interaction score: highest confidence (0.900)
number of nodes: 64
number of edges: 393
average node degree: 12.3
avg. local clustering coefficient: 0.817
expected number of edges: 72
PPI enrichment p-value: < 1.0e-16

Supplementary Figure S5. Functional disorder in human proteins interacting with the RABV L-protein. For each protein, an amino acid sequence in FASTA format is shown followed by the disorder profile generated by RIDAO, D^{2}P^2-generated functional disorder profile, modeled 3D structure generated by AlphaFold, and STRING-based protein-protein interaction network.

DYNLL1 (UniProt ID: P63167)

>sp|P63167|DYL1_HUMAN Dynein light chain 1, cytoplasmic OS=Homo sapiens OX=9606 GN=DYNLL1 PE=1 SV=1

MCDRKAVIKNADMSEEMQQDSVECATQALEKYNIEKDIAAHIKKEFDKKYNPTWHCIV GRNFGSYVTHETKHFIYFYLGQVAILLFKSG

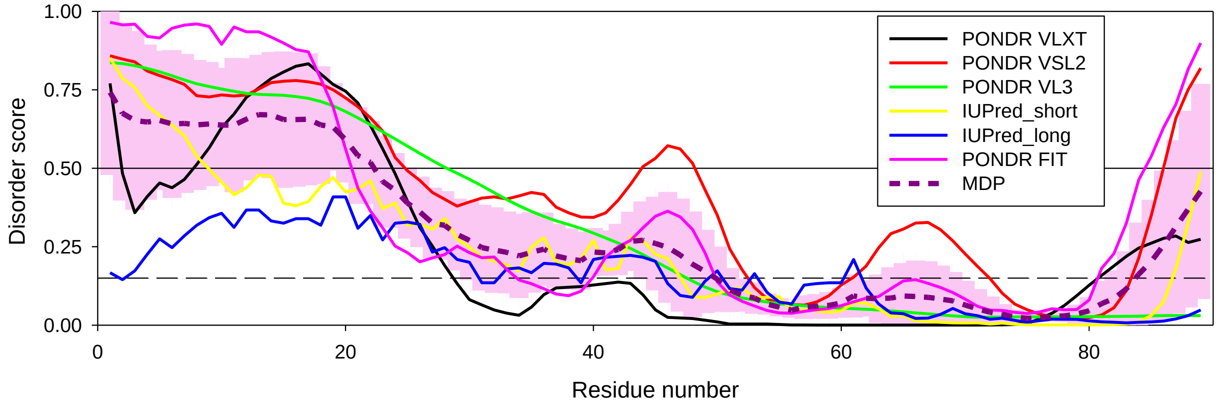

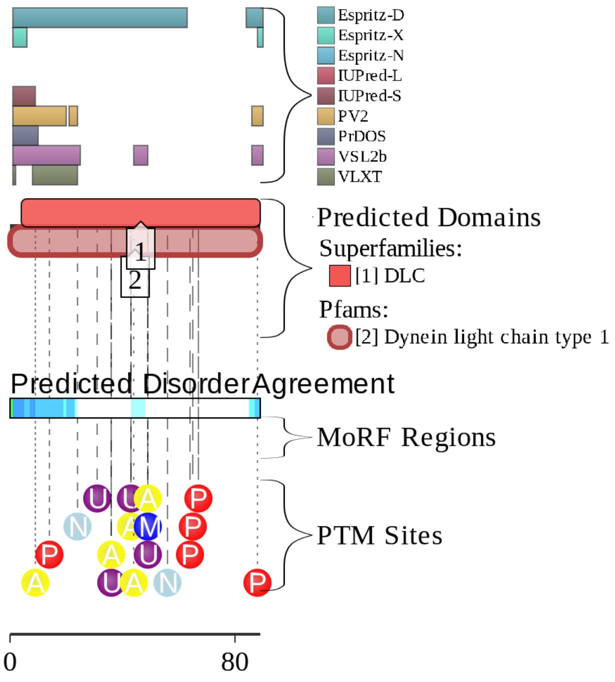

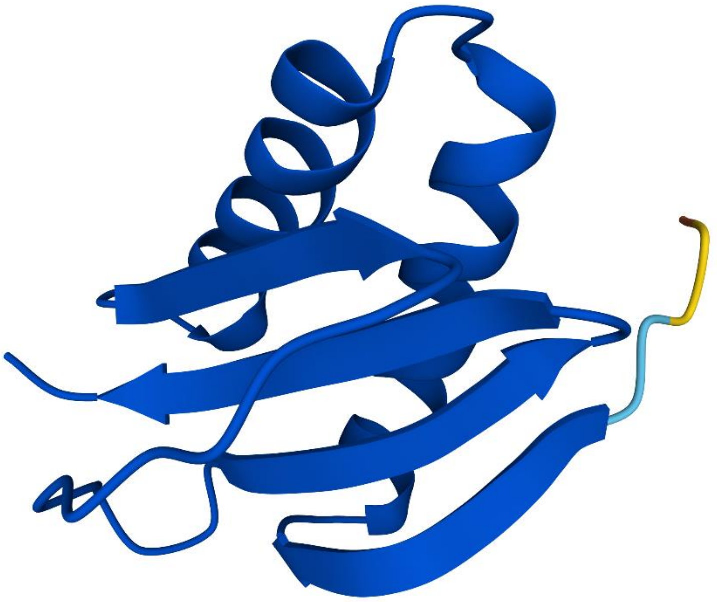

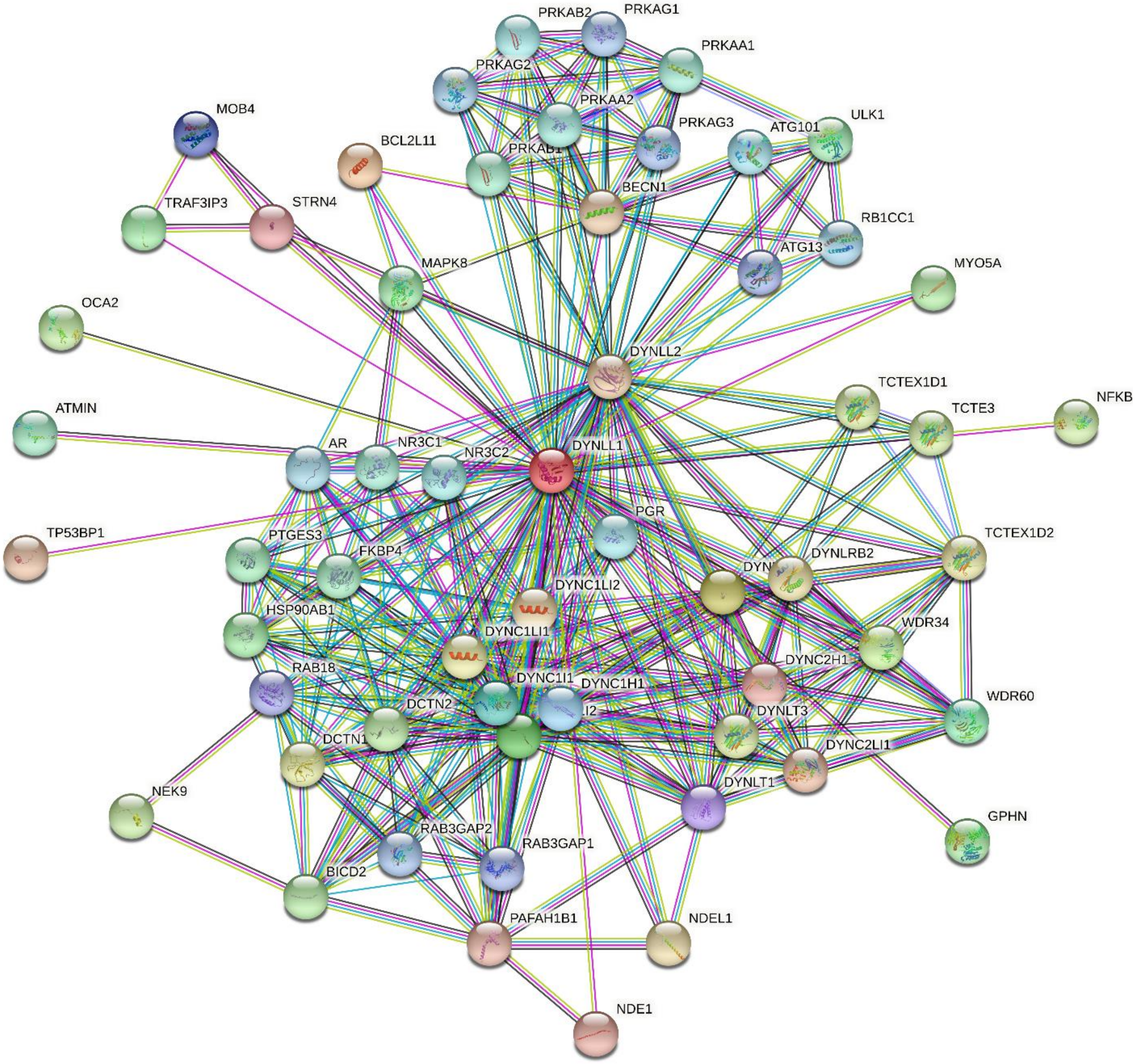

minimum required interaction score: highest confidence (0.900)
number of nodes: 58
number of edges: 338
average node degree: 11.7
avg. local clustering coefficient: 0.828
expected number of edges: 68
PPI enrichment p-value: < 1.0e-16
